# Continuum topological derivative - a novel application tool for denoising CT and MRI medical images

**DOI:** 10.1186/s12880-024-01341-1

**Published:** 2024-07-24

**Authors:** Viswanath Muthukrishnan, Sandeep Jaipurkar, Nedumaran Damodaran

**Affiliations:** 1 Central Instrumentation & Service Laboratory, Guindy Campus, University of Madras, Chennai, India; 2 Image Art, Vijaya Health Centre, Vadapalani, Chennai India

**Keywords:** Denoising, Continuum topological derivative, HRCT images, MRI images, Quantum mottle, Rayleigh noise, Gaussian noise

## Abstract

**Background:**

CT and MRI modalities are important diagnostics tools for exploring the anatomical and tissue properties, respectively of the human beings. Several advancements like HRCT, FLAIR and Propeller have advantages in diagnosing the diseases very accurately, but still have enough space for improvements due to the presence of inherent and instrument noises. In the case of CT and MRI, the quantum mottle and the Gaussian and Rayleigh noises, respectively are still present in their advanced modalities of imaging. This paper addresses the denoising problem with continuum topological derivative technique and proved its trustworthiness based on the comparative study with other traditional filtration methods such as spatial, adaptive, frequency and transformation techniques using measures like visual inspection and performance metrics.

**Methods:**

This research study focuses on identifying a novel method for denoising by testing different filters on HRCT (High-Resolution Computed Tomography) and MR (Magnetic Resonance) images. The images were acquired from the Image Art Radiological Scan Centre using the SOMATOM CT and SIGNA Explorer (operating at 1.5 Tesla) machines. To compare the performance of the proposed CTD (Continuum Topological Derivative) method, various filters were tested on both HRCT and MR images. The filters tested for comparison were Gaussian (2D convolution operator), Wiener (deconvolution operator), Laplacian and Laplacian diagonal (2nd order partial differential operator), Average, Minimum, and Median (ordinary spatial operators), PMAD (Anisotropic diffusion operator), Kuan (statistical operator), Frost (exponential convolution operator), and HAAR Wavelet (time–frequency operator). The purpose of the study was to evaluate the effectiveness of the CTD method in removing noise compared to the other filters. The performance metrics were analyzed to assess the diligence of noise removal achieved by the CTD method. The primary outcome of the study was the removal of quantum mottle noise in HRCT images, while the secondary outcome focused on removing Gaussian (foreground) and Rayleigh (background) noise in MR images. The study aimed to observe the dynamics of noise removal by examining the values of the performance metrics.

In summary, this study aimed to assess the denoising ability of various filters in HRCT and MR images, with the CTD method being the proposed approach. The study evaluated the performance of each filter using specific metrics and compared the results to determine the effectiveness of the CTD method in removing noise from the images.

**Results:**

Based on the calculated performance metric values, it has been observed that the CTD method successfully removed quantum mottle noise in HRCT images and Gaussian as well as Rayleigh noise in MRI. This can be evidenced by the PSNR (Peak Signal-to-Noise Ratio) metric, which consistently exhibited values ranging from 50 to 65 for all the tested images. Additionally, the CTD method demonstrated remarkably low residual values, typically on the order of e^−09^, which is a distinctive characteristic across all the images. Furthermore, the performance metrics of the CTD method consistently outperformed those of the other tested methods. Consequently, the results of this study have significant implications for the quality, structural similarity, and contrast of HRCT and MR images, enabling clinicians to obtain finer details for diagnostic purposes.

**Conclusion:**

Continuum topological derivative algorithm is found to be constructive in removing prominent noises in both CT and MRI images and can serve as a potential tool for recognition of anatomical details in case of diseased and normal ones. The results obtained from this research work are highly inspiring and offer great promise in obtaining accurate diagnostic information for critical cases such as Thoracic Cavity Carina, Brain SPI Globe Lens 4th Ventricle, Brain-Middle Cerebral Artery, Brain-Middle Cerebral Artery and neoplastic lesions. These findings lay the foundation for implementing the proposed CTD technique in routine clinical diagnosis.

**Supplementary Information:**

The online version contains supplementary material available at 10.1186/s12880-024-01341-1.

## Introduction

Medical imaging technology have been grown rapidly from the screen-film to the sophisticated medical images arising from modern modalities wherein digital imaging have been playing a predominant role, in particular CT and MRI. Medical image analysis is aided by various image processing tools like image enhancement, quantification, visualization and computer aided detection [1, 2]. Objective of image restoration is denoising of the degraded medical image to remove the noise generated from non-linearity of sensors, grains, defects in image capturing, erroneous focus of image, distortion due to relative motion of object during imaging, etc.

### Imaging modalities

In this paper, two imaging modalities such as CT and MRI and their denoising characteristics using CTD technique are presented.

### Computer Tomography (CT)

Computer Tomography (CT) has been developed in stages like planar, cross-section and 3-D reconstruction, etc., and has attained the present stage of High Resolution Computed Tomography (HRCT) [3–7]. In HRCT, minimum field of view and optimized resolution of images are the main highlights of HRCT, which proved its ability to give clear images to find lung fibrosis [8], and bronchial tree lesion [9–11]. Further, the image reconstruction algorithms employed in HRCT are capable of extending high spatial-frequency resolution.

Since its introduction in 1972, computed tomography (CT) has evolved significantly with advancements including cardiac gating CT, spectral CT, multidetector CT (MDCT), energy-sensitive photon counting detector CT (PCD-CT), phase contrast CT, spiral CT, sequential CT, coronary CT, triple-phase CT, electron beam CT (EBCT), helical CT, perfusion CT, and high-resolution CT (HRCT), among others. The utilization of SOMATOM HRCT in this study offers several advantages. It allows for low contrast agent dosage, as X-ray tubes can generate high mA at low kV even with reduced contrast agent usage. Additionally, it minimizes the use of rare earth metals and incorporates a high number of detectors, resulting in energy-efficient HRCT machines. This is particularly beneficial for thorax and cardiac procedures, as it facilitates dynamic imaging with excellent temporal resolution, fast scan results, accurate neuro perfusion maps, and reduced metal and motion artifacts. HRCT proves to be highly valuable in the diagnosis of lung disorders and diseases within the thorax region due to its exceptional accuracy.

### Magnetic Resonance Imaging (MRI)

In Magnetic Resonance Imaging (MRI), radio wave is applied on the human beings (excitation or protons) under the influence of strong magnetic field (to align the precision of the proton in a particular direction of the applied magnetic field) and detecting the relaxation times like T1 and T2 of the excited protons forms the basis of the MRI. For image formation in MRI, Radon transform or Fourier Transform was employed [12] to convert the k-space proton relaxation time into pixels. The MRI advanced modalities like Fluid Attenuated Inversion Recovery (FLAIR), Diffusion Weighed Imaging (DWI), and Periodically Rotated Overlapping Parallel Lines with Enhanced Reconstruction (PROPELLER) have helped to overcome the MRI sample *k-space* over a rotatory time period.

In the case of MRI, the three principal nuclei utilized to study metabolism, fluids, and cell membrane composition with pH levels are carbon (C-13), hydrogen, and phosphorus (P31), respectively. For this research, the SIGNA Explore MRI machine was employed, which offers several advantages. It provides very silent scans (< 3 decibels), enables 3D volumetric brain imaging, allows for free-breathing scans, facilitates low sedation scans, distinguishes between calcifications and blood vessels effectively, and provides good proton density sequences. These features enhance the overall imaging experience and contribute to obtaining high-quality results in terms of noise reduction, precise volumetric imaging, and accurate differentiation between different tissues and structures.

### Noises in CT and MR images

In this work, two different types of noises originating from the CT and MRI are attempted using the proposed CTD algorithm.

### Quantum mottle in CT

Quantum mottle is one of the preliminary noises in CT due to loss in photon reaching the detector, which causes image density fluctuation, decreased spatial resolution and poor contrast resolution. This loss in the number of photons is governed by the Poisson distribution on a pixel-to-pixel scale. If $P_{{{{} \mathord{\left/ {\vphantom {{} p}} \right. \kern-0pt} p}}}^{avg}$ is the average number of photons per pixel then, the standard deviation can be defined as $P_{avg}^{SD} = \sqrt {P_{{{{} \mathord{\left/ {\vphantom {{} p}} \right. \kern-0pt} p}}}^{avg} }$. Now the observed quantum mottle noise $Q_{N}^{CT}$ can be equated as

1 $$Q_{N}^{CT} = {\raise0.7ex\hbox{${\sqrt {P_{avg}^{SD} } }$} \!\mathord{\left/ {\vphantom {{\sqrt {P_{avg}^{SD} } } {P_{{{{} \mathord{\left/ {\vphantom {{} p}} \right. \kern-0pt} p}}}^{avg} }}}\right.\kern-0pt} \!\lower0.7ex\hbox{${P_{{{{} \mathord{\left/ {\vphantom {{} p}} \right. \kern-0pt} p}}}^{avg} }$}}$$

In the above Eq. (1), $Q_{N}^{CT}$ is the distributive noise. For quantum noise removal, Filtered Back Projection (FBP) and sinogram affirmed iterative reconstruction (SAFIRE) techniques were used for the estimation of quantum noise in CT images [13–17]. Wavelet transform and its different modes and types were experimented for removing the quantum mottle noise in CT [18–24]. Further, spatio-temporal filtering [25], Bayes filter [26], multineural network filter [27], adaptive multineural network filter [28], stochastic method [29], adaptive statistical iterative reconstruction-V [30], iterative reconstruction algorithm [31–36], deep learning reconstruction algorithm [37, 38], Shearlet transform [39] and neural network [16] are some of the other methods tried to reduce the quantum mottle noise in CT images. The denoising process in the wavelet method involves decomposition of levels, thresholding, and scaling factors. Spatio-temporal filtering, on the other hand, utilizes ordinary mathematical operations followed by basic statistical operations. However, these approaches often struggle to produce crucial diagnostic information due to the averaging of image contents.

In contrast, the Bayes filter employs probability density functions for the filtering process, while neural, multineural, and adaptive multineural methods are based on training datasets. Adaptive statistical iterative reconstruction utilizes statistical and probability distribution functions to perform the filtering operation. The Shearlet transform, on the other hand, utilizes sparse representation to denoise quantum mottle noise.

While these methods often involve averaging existing information through mathematical operations or require time-consuming iterative algorithm testing, the proposed CTD method takes a different approach. It applies a simple perturbation (cost-function) threshold to identify noise information from useful pixel information. This allows for accurate details by discarding the noise information as desired. Moreover, the cost-function can be adjusted to fine-tune the denoising process and obtain precise results [40–51].

### Gaussian and Rayleigh’s noises in MRI

In MRI, k-space variables are used to form the image using Radon transformation. Noise in $k - space$ is characterised by independent and identically distributed random variables called, Gaussian Random variables, which are complex in nature. In MRI, feature extraction and classification are affected by these noises and are additive in nature. Various researchers have tried to reduce the noises in MRI using different filtering techniques [52–55].

The Gaussian noise present in MRI image [56] can be expressed as

2 $$N_{MRI}^{G} = \sqrt {D_{MRI}^{rs} } N_{MRI}^{G1} + N_{MRI}^{G2}$$

In the above Eq. (2), $N_{MRI}^{G}$ represents the Gaussian noise of the MRI image, $N_{MRI}^{G1}$ and $N_{MRI}^{G2}$ are independent zero-means. Wavelet filters [57–61], histogram equalization [62, 63], median filter [64–67] total variation method [55], adaptive wiener filter [68], multiscale enhancement along with Susan edge detector [69], Bayesian method [70], non-local means filter [71, 72], hybrid adaptive algorithm [66], anisotropic diffusion filter [40, 41, 73], bilateral filter [74], convolution neural network [75] and non-local averaging [42] are some of the techniques employed for denoising the Gaussian noise in MRI images. When it comes to Gaussian noise present in MR images, different denoising methods exhibit varying strengths. Histogram equalization primarily enhances contrast information, while the total variation method, multiscale enhancement, and bilateral filters excel in preserving sharp edges. The Bayesian approach can remove additive white noise in MRI, but its effectiveness may be limited. Non-Local Means (NLM) and hybrid adaptive algorithms are often successful in reducing random noise in MRI. On the other hand, the anisotropic diffusion filter and non-local averaging methods can only moderately reduce Gaussian noise in MR images. It is important to note that no single method is capable of reducing Gaussian noise to the desired level perfectly.

Therefore, the proposed CTD method was implemented to address noise reduction in a constructive manner, considering the limitations of other methods. By employing a tailored approach, the CTD method aims to effectively reduce noise while preserving important image details [65, 76–81].

Rayleigh noise concentrates on the magnitude of the image and lies in the background of the MRI image. This background noise removal brings down the lossless compression ratio, which improve the efficiency of image transfer through internet for telemedicine purposes [43]. Rayleigh noise depends on the noise density in the image pixels and is modelled on the Rayleigh curve represented by

3 $$N_{d}^{Ry} \left( {I_{m}^{g} } \right) = \left[ \begin{gathered} \frac{2}{{g_{1} }}\left[ {g - g_{0} } \right]e^{{\frac{{\left( {g - g_{0} } \right)^{2} }}{{g_{1} }}}} \;\;g \ge g_{0} \hfill \\ 0\quad \quad \quad \quad \quad \quad \;\;\,\;g < g_{0} \; \hfill \\ \end{gathered} \right.$$

In Eq. (3), $N_{d}^{Ry} \left( {I_{m}^{g} } \right)$ gives the noise density, $g$ represents the grey level intensity, $g_{0}$ represents the minimum grey level intensity and $g_{1}$ represents the maximum grey level intensity.

Haar wavelet [43], complex diffusion prior [44], partial differential equation [45, 82, 83], Villullas-Martin’s filter [46], anisotropic diffusion [47], adaptive filters [48], discrete complex wavelet [49], Taylor-Krill Herd-based Support Vector Machine [50], Bayes classifier [51], conventional approach [84], maximum-likelihood [85], orthogonal matching pursuit sparsity method [86], genetic programming [87], Markov random fields based maximum a posteriori method [88], non-linear regression models [89], sum of squares reconstruction [90], Bayesian [91], variational model [92], non-local mean filter [72], multi-dilated block network [76] and Nakagami distribution [77] were deployed to remove the Rayleigh noise in MRI images. While these methods were able to reduce Rayleigh noise to a moderate level, the Villullas-Martin’s filter fell short in preserving contour details as expected. The maximum-likelihood method focused solely on removing thermal noise in MRI, and the orthogonal matching pursuit sparsity method only provided a solution for sparse representation.

In order to address the limitations and drawbacks of these existing methods for removing Rayleigh noise in MRI, the proposed CTD method was implemented. The CTD method aims to overcome these limitations by offering improved noise reduction while preserving contour details to the desired level [93–102].

## Methodology

Denoising filters have wide applications in removing the noises present in most of the imaging modalities. They can be categorized as spatial [65, 78–81, 103–105], adaptive [106, 107] and transformation [108] filters. Spatial filters are classified into linear and non-linear types concentrating on neighbourhood operations. Adaptive filters operate on statistical measures like mean and variance, while the transformation filters depend on techniques like Fourier, Curvelet, Contourlet and Wavelet transforms. Filters along with their functions are summarized in Table 1. These noise filters were tested for noise removal in CT and MR images and their performance are summarized in Table 1. These three different categories of traditional filter have been chosen for this work for estimating the performance of the proposed CTD filter by comparing their denoising characteristics using various performance measures. For comparing the performance of the filters, eighteen performance metrics viz., Average Difference (AD), Mean Square Error (MSE), Root Mean Square Error (RMSE), Maximum Difference (MD), Normalized Absolute Error (NAE) and Normalized Mean Square Error (NMSE), Peak Signal to Noise Ratio (PSNR), Structural Content (SC), Correlation Coefficient (CC), Normalized Cross Correlation (NCC), Image Quality Index (IQI) and Structural Similarity Index Map (SSIM), Contrast to Noise Ratio (CNR), Noise Index (NI), Average Signal to Noise Ratio (ASNR), Image Variance (IV), Noise Standard Deviation (NSD), and Equivalent Number of Looks (ENL) were selected and their mathematical forms, definition and range of values are summarized in Table 2 [109].

**Table 1:** List of de-noising filters used

Name	Type	Mathematical function	Resultant Image	Noise Removal
Gaussian	Frequency	Cut off	Smooth	Average
Wiener	Frequency	Low pass	Smooth	Better
Laplacian	Linear	Derivative	Edge detection	Average
Laplacian Diagonal	Linear	Derivative	Sharp	Poor
Average	Spatial (Low pass)	Average	Blurred Edges	Average
Minimum	Non-linear	Minimum	Smooth	Poor
Median	Non-linear	Medium	Smooth	Good
PMAD	Diffusion	Derivative	Smooth	Average
Kuan	Adaptive	Statistical	Smooth	Good
Frost	Adaptive	Statistical	Smooth	Good
HAAR Wavelet	Frequency	Orthogonal	Smooth	Very Good
CTD	Isotropic Conductivity Diffusion	Cost	High quality & contrast denoised image	Superlative

**Table 2:** Performance metrics of the denoising filters

Metrics	Mathematical Expression	Definition	Range
AD	$AD = \frac{1}{mn}\sum\limits_{i = 1}^{m} {\sum\limits_{j = 1}^{n} {\left[ {I_{m} \left( {i,j} \right) - I_{m}^{F} \left( {i,j} \right)} \right]} }$	Mean difference between the original and the denoised image	AD is minimum for high quality denoised images and vice versa *AD* = 0 *to* 255
MSE	$MSE = \frac{1}{mn}\sum\limits_{i = 1}^{m} {\sum\limits_{j = 1}^{n} {\left( {I_{m} \left( {i,j} \right) - I_{m}^{F} \left( {i,j} \right)} \right)} }^{2}$	Average difference between the original and the denoised image	MSE is minimum for high quality denoised images and vice versa *MSE* = 0 *to* 255
RMSE	$RMSE = \sqrt {\frac{1}{mn}\sum\limits_{i = 1}^{m} {\sum\limits_{j = 1}^{n} {\left( {I_{m} \left( {i,j} \right) - I_{m}^{F} \left( {i,j} \right)} \right)} }^{2} }$	Square root of the difference between the square of noised image and the filtered image divided by the size of the original image	RMSE is minimum for high quality denoised images and vice versa
PSNR	$$\begin{gathered} PSNR = 10\log_{10} \frac{{\left( {2^{n} - 1} \right)^{2} }}{MSE} \hfill \\ \quad \quad \;\;{\kern 1pt} = 10\log_{10} \frac{{255^{2} }}{MSE} \hfill \\ \end{gathered}$$	Quantitative measurement of distortion of the signal and a qualitative measurement for comparison of noise	PSNR is maximum for high quality denoised images and vice versa
MD	$MD = \max \left( {\left| {I_{m} \left( {i,j} \right) - I_{m}^{F} \left( {i,j} \right)} \right|} \right)$	Maximum error between the original image and filtered image	MD is minimum for high quality denoised images and vice versa
NAE	$NAE = \frac{{\sum\limits_{i = 1}^{m} {\sum\limits_{j = 1}^{n} {\left| {\left[ {I_{m} \left( {i,j} \right) - I_{m}^{F} \left( {i,j} \right)} \right]} \right|} } }}{{\sum\limits_{i = 1}^{m} {\sum\limits_{j = 1}^{n} {\left| {\left[ {I_{m} \left( {i,j} \right)} \right]} \right|} } }}$	Numerical variance of the filtered image w.r.t the original image	NAE is minimum for high quality denoised images and vice versa NAE = 0 ~ 1
NMSE	$NMSE = \frac{{\sum\limits_{i = 1}^{m} {\sum\limits_{j = 1}^{n} {\left[ {I_{m} \left( {i,j} \right) - I_{m}^{F} \left( {i,j} \right)} \right]^{2} } } }}{{\sum\limits_{i = 1}^{m} {\sum\limits_{j = 1}^{n} {\left[ {I_{m} \left( {i,j} \right)} \right]^{2} } } }}$	Measurement of the variation of MSE	NMSE is minimum for high quality denoised images and vice versa
SC	$SC = \frac{{\sum\limits_{i = 1}^{m} {\sum\limits_{j = 1}^{n} {\left[ {I_{m} \left( {i,j} \right)} \right]^{2} } } }}{{\sum\limits_{i = 1}^{m} {\sum\limits_{j = 1}^{n} {\left[ {I_{m}^{F} \left( {i,j} \right)} \right]^{2} } } }}$	Qualitative representation in terms of the correlation function and quantitative measurement for comparison of similarity between the original image and filtered image	$$\begin{array}{c}SC=1\Rightarrow \\ {I}_{m}\left(i,j\right)={I}_{m}^{F}\left(i,j\right)\end{array}$$
CC	$CC = \frac{{\sum\limits_{i = 1}^{m} {\sum\limits_{j = 1}^{n} {\left[ {\left[ {I_{m} \left( {i,j} \right) - \overline{I}_{m} \left( {i,j} \right)} \right]\left[ {I_{m}^{F} \left( {i,j} \right) - \overline{I}_{m}^{F} \left( {i,j} \right)} \right]} \right]} } }}{{\sqrt {\sum\limits_{i = 1}^{m} {\sum\limits_{j = 1}^{n} {\left[ {I_{m} \left( {i,j} \right) - \overline{I}_{m} \left( {i,j} \right)} \right]^{2} } } } \sqrt {\sum\limits_{i = 1}^{m} {\sum\limits_{j = 1}^{n} {\left[ {I_{m}^{F} \left( {i,j} \right) - \overline{I}_{m}^{F} \left( {i,j} \right)} \right]^{2} } } } }}$	Inter-relationship between original and denoised image in terms of edge preservation	$\text{CC}=0\sim 1$ $$\begin{array}{c}CC=1\Rightarrow \\ {I}_{m}\left(i,j\right)={I}_{m}^{F}\left(i,j\right)\end{array}$$
NCC	$NCC = \frac{{\sum\limits_{i = 1}^{m} {\sum\limits_{j = 1}^{n} {I_{m} \left( {i,j} \right) \cdot I_{m}^{F} \left( {i,j} \right)} } }}{{\sum\limits_{i = 1}^{m} {\sum\limits_{j = 1}^{n} {\left[ {I_{m} \left( {i,j} \right)} \right]^{2} } } }}$	Establishes degree of similarity between original and filtered images	$NCC=-1\sim 1$
IQI	$IQI=\frac{4{\sigma }_{{I}_{m}{I}_{m}^{F}}{\overline{I} }_{m}{\overline{I} }_{m}^{F}}{\left({{{\sigma }^{2}}_{I}}_{m}+{{\sigma }^{2}}_{{I}_{m}^{F}}\right)\left({\overline{I} }_{m}^{2}+{\overline{I} }_{m}^{2_{F}}\right)}$	Deformation in terms of loss of correlation, mean distortion and variance distortion	$IQI=-1\sim 1$
SSIM	$SSIM=\frac{\left(2{\overline{I} }_{m}{\overline{I} }_{m}^{F}+{c}_{1}\right)\left(2{\sigma }_{{I}_{m}{I}_{m}^{F}}+{c}_{2}\right)}{\left({\overline{I} }_{m}^{2}+{\overline{I} }_{m}^{2_{F}}+{c}_{1}\right)\left({{{\sigma }^{2}}_{I}}_{m}+{{\sigma }^{2}}_{{I}_{m}^{F}}+{c}_{2}\right)}$	Evaluates the degradation of the image and is considered as comparison metric for structure, contrast as well as luminance between the original image and the filtered image	$\text{SSIM}=0\sim 1$ $$\begin{array}{c}SSIM=1\Rightarrow \\ {I}_{m}\left(i,j\right)={I}_{m}^{F}\left(i,j\right)\end{array}$$
CNR	$CNR = \frac{{\left| {\overline{I}_{m} - \overline{I}_{m}^{F} } \right|}}{{\sqrt {\sigma^{2}_{{I_{m} }} + \sigma^{2}_{{I_{m}^{F} }} } }}$	Qualitative metric for identifying differences between two ROI	CNR is maximum for high quality denoised images and vice versa
NI	$NI = \frac{{\sigma_{{I_{m}^{F} }} }}{{\overline{I}_{m}^{F} }}$	An inherent property of imaging instrument and measurement of noise removal	NI is minimum for high quality denoised images and vice versa
ASNR	$ASNR = \frac{{\overline{I}_{m}^{F} }}{{\sigma_{{I_{m}^{F} }} }}$	Ratio of the mean value of the standard deviation and variation of noise to the mean value	ASNR is maximum for high quality denoised images and vice versa
IV	$IV = \frac{1}{mn}\sum\limits_{i = 1}^{m} {\sum\limits_{j = 1}^{n} {\left[ {I_{m}^{F} \left( {i,j} \right) - \overline{I}_{m}^{F} \left( {i,j} \right)} \right]} }^{2}$	Quantitative metric for the description of factors present in noise and is independent of intensity	IV is minimum for high quality denoised images and vice versa
NSD	$NSD = \sqrt {\frac{1}{mn}\sum\limits_{i = 1}^{m} {\sum\limits_{j = 1}^{n} {\left[ {I_{m}^{F} \left( {i,j} \right) - I_{m}^{Fn} \left( {i,j} \right)} \right]} }^{2} }$	Quantitative metric that describes noise reduction in images and narrates the constituents of noise present in the image	NSD is minimum for high quality denoised images and vice versa
ENL	$ENL = \frac{{\left( {I_{m}^{Fn} \left( {i,j} \right)} \right)^{2} }}{{NSD^{2} }}$	Quantitative metric for the estimation of noise present in images and a pivotal tool used for the statistical modelling of images	ENL is maximum for high quality denoised images and vice versa

The proposed CTD filter and its mathematical definition are described in the following section.

### Continuum Topological Derivative (CTD)

The Topological Derivative (TD) concept was originally conceived from structural mechanics to solve shape optimization and topology optimization problems. Later on, it was utilized to solve image processing problems. In this research work, Continuum Topological Derivative (CTD) was proposed for solving discontinuities in the domain, boundaries and improving the shape sensitivity of CT and MR images [110].

Further, the CTD can be effective in solving the changes occurred during processing of images. All these features of the CTD impressed us to choose for denoising the CT and MR images. The main advantage of CTD lies that even after the object got distorted by an external factor, it is still possible to study the properties of the object with respect to the original one [111]. This concept further influences the proposed denoising studies carried out in CT and MR images [112]. The topological derivative can be expressed as,

4 $$D_{T} (\widehat{x}) = \mathop {\lim }\limits_{\varepsilon \to \infty } \frac{{\Psi (\Omega_{\varepsilon } ) - \Psi (\Omega )}}{f(\varepsilon )}$$

where $\Psi (\Omega_{\varepsilon } )$ is the cost function of the perturbed domain, $\Psi (\Omega )$ is the cost function of the original domain, $f(\varepsilon )$ is the monotone function, $D_{T} (\widehat{x})$ is the topological derivative for a perturbation factor $\varepsilon$. Further, TD with advanced techniques like wavelet transform, Discrete Filter Bank (DFB), and Contourlet Transform (CLT) have been experimented to solve the perturbed domain issues of extracting contours, boundaries and edges in biomedical images [93]. The TD, CLT and interpolation methods help to resolve the edges in the organs of the biomedical images [94]. The CLT with Pyramidal Directional Filter Bank (PDFB) acts as a tool for flexibility of decomposition at the sub-band level [95]. The TD can produce enhanced efficiency in the segmentation of medical images with the introduction of diffusion concept [96]. Moreover, the Topological derivative could be applied for getting resolved images in Electrical Impedance Tomography (EIT) [97]. The TD with functional analysis techniques can provide emphasized results in delineation attributes of the medical images [98]. In the case of Continuum Topological Derivative (CTD), the features like shape functional, asymptotic expansion, sensitivity, and boundary and edge functional play a pivotal role in CTD while arriving at denoising results of CT and MRI medical images. The fundamental expression for CTD is given by,

5 $$\Psi \left[ {\Omega_{\varepsilon } } \right] - \Psi \left[ \Omega \right] = f_{\varepsilon } D_{C}^{T} \left( {\hat{x}} \right)$$

The Eq. (5) lays the foundation stone for CTD expressed in terms of cost function of original and perturbed domains along with monotonic function. Treating the state equation as the domain, which represents a specific region in the human body or an specific organ in the human body, the shape of it will be represented as $\Psi \left[ \Omega \right]$, where $\Psi$ is the state function and the $\Omega$ is the domain that is characteristic of the state equation. Here, $\Psi \left[ \Omega \right]$ handles the original domain in an undisturbed state. Introduction of a perturbation into this domain will induce topological variation in the state of the domain. In such a case, $\Psi \left[ \Omega \right]$ is defined by the approximate solution. Obtaining approximate solution to linear system in the case of sparse system provides a perceptual way to solve problems [99]. Further, approximate solutions are of great use when handling noisy data [100]. As a result, the approximate solutions for the sparse systems which handle rank of the matrix provide a neat frame work for tomographic study on a quantum scale [101].

Shape sensitivity, parameterized domain, edge detection technique, elliptical boundary variational problems, adjoint method, mapping procedure, constraints on the domain, and gradient method aids in boundary studies along with numerical results are some of the vital applications of CTD [102, 113–115]. Study of elliptical boundary problems is important in finding the fundamental solutions, positive solutions and non-linear boundary value problem [116–122]. Treating mesh as equivalent to the arrangement of pixels in the medical image, one can evaluate the difference approximation for the elliptical boundary value problems [123]. Also, the elliptical boundary problem $\Psi \left[ \Omega \right]$ is based on the solution of the state function $\Psi$, i.e., the shape functional. With this background the CTD can also be expressed as

$$\begin{gathered} D_{T} \left( {\hat{x}} \right) = \, Asymptotic \, analysis \, of \, solutions \, to \, elliptic\;Boundary \, value \, problems \, in \, singularly\ perturbed \, domain \ + Asymptotic \, analysis \, of \, Shape \, functional. \hfill \\ \end{gathered}$$

The CTD was used to solve the denoising problems in CT and MR images and the algorithm for the same was tested for different images in the clinical examples as illustrated in the following sections.

### Specification of tools

The proposed CTD and other tranditional denoising algorithms were tested in various CT and MR images, The image specification, hardware and the software details are given below. The CT and MR images in the Digital imaging and communication system (DICOM) format, which enables user-friendly interaction between Picture Archiving and Communication System (PACS) and Radiology Information Systems (RIS), were acquired from the scan facility available at Image Art, Vijaya Health Centre, Vadapalani, Chennai. The HRCT images were taken from SOMATOM dual-source CT, which is the fastest CT scan machine in the present scenario. All the HRCT scans were taken at 1.00 mm thick, 40 mm in beam length, and 400 mm in beam width (or Window Width). The images were acquired in $0^{ \circ }$ angle tilt or gantry tilt and 30.3 cm Display Field of View (DFOV). The operating power inputs of HRCT are 120 kV and 178 mA. The kernel used for soft tissue reconstruction is Br40f/3. The resolution of the images acquired is 480 × 340 (width x height) with a bit depth of 32 bits. Brain HRCT images are also collected from the same scan centre in spiral (SPI) mode. The brain MR images were acquired from the GE Healthcare SIGNA™ Explorer machine operating at 1.5 Tesla magnetic fields. We have collected and tested more than 300 HRCT and MR images for studying the denoising effect of the proposed algorithm. Of which, the results of five HRCT and MR images are given here for comparison. The algorithms were tested on an HP laptop with AMD Ryzen5 3500U processor, Radeon Vega mobile Gfx graphics card, 8 GB RAM, and MATLAB 2018a on a 64-bit Windows 10 system.

### Clinical examples

The proposed CTD and traditional filters were tested on a HRCT image (Thoracic Cavity Carina, Head CT (Globe Lens 4th Ventricle), and nine MR images (six Middle Cerebral Artery territory of DWI, FLAIR T2, and PROP T2), and three neoplastic lesions (two PROP and FLAIR T1). Quality metrics were computed using denoised and original images to compare the efficiency of filters in handling quantum mottle and Gaussian/Rayleigh noises in CT and MR images respectively. A radiographic expert assessed image quality through visual inspection. Clinical example images, estimated quality metrics, and histogram plots are presented in Figs. 1, 3, 5, 7, 9, 11, 13, 15, 17, 19, 21, Tables 3, 4, 5, 6, 7, 8, 9, 10, 11, 12 and 13, and histogram plots in Figs. 2, 4, 6, 8, 10, 12, 14, 16, 18, 20, 22 respectively.

**Fig. 1:**
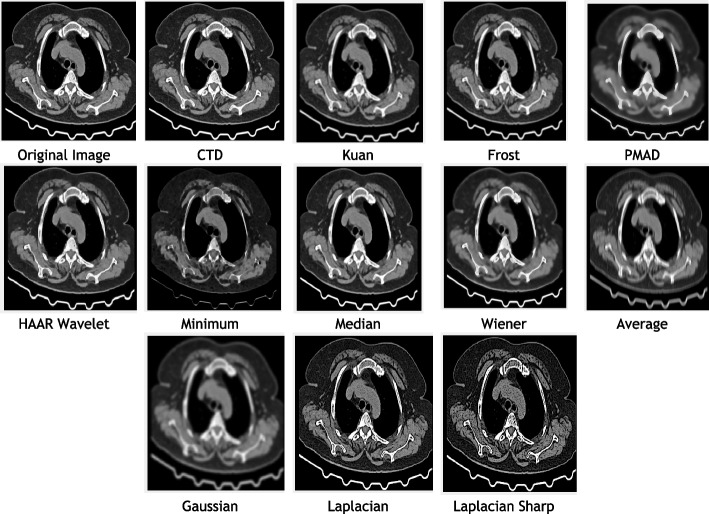
Original and denoised Images of Carina

**Table 3:** Quality metric for Carina

Metrics	Continuum TD	Kuan Filter	Frost Filter	PMAD Filter (15 itrs)	Wavelet	Ordinary Filter Min	Median Filter	Wiener Filter	Average Filter 7 × 7	Gaussian Filter	Laplacian Filter	Laplacian Filter Sharp
AD	0.0991	3.31	2.83	5.40	1.71	18.40	1.66	3.06	7.56	6.96	7.07	11.40
MSE	0.3853	26.61	23.07	44.10	14.11	95.88	13.66	30.75	40.56	41.75	51.81	65.84
RMSE	0.6207	5.15	4.80	6.64	3.75	9.79	3.69	5.54	6.36	6.46	7.19	8.11
PSNR	52.27	33.87	34.49	31.68	36.63	28.31	36.77	33.25	32.04	31.92	30.98	29.94
MD	34	116	111	97	21	255	181	72	205	154	180	220
NAE	0.0018	0.0628	0.0539	0.1026	3.26E-02	0.3494	0.0315	0.0581	0.1436	0.1322	0.1342	0.2165
NMSE	0.0024	0.1717	0.1492	0.2822	9.07E-02	0.6186	0.0885	0.1974	0.2607	0.2681	0.3360	0.4262
SC	1	0.9621	0.9695	0.9170	0.9903	1.08	0.9990	0.9534	0.9521	0.9005	1.17	1.39
CC	1	0.9816	0.9850	0.9667	0.9967	0.8535	0.9940	0.9883	0.8882	0.9268	0.9672	0.9274
NCC	1	1.0069	1.0067	1.0063	1.0017	0.9427	1.0005	1.0052	1.0042	1.0083	0.8850	0.7452
IQI	0.9986	0.9528	0.9560	0.8567	0.9364	0.7459	0.9851	0.8846	0.8906	0.8622	0.9646	0.9178
SSIM	0.9982	0.9041	0.9243	0.6925	0.9440	0.6465	0.9437	0.8353	0.7003	0.6655	0.8849	0.7985
CNR	1.50E-06	1.56E-04	8.05E-04	2.32E-04	2.97E-05	0.2311	0.0042	0.0037	0.0138	2.26E-04	0.0773	0.1246
NI	1.88E-05	1.75E-05	1.77E-05	1.69E-05	1.85E-05	2.05E-05	1.86E-05	1.77E-05	1.69E-05	1.57E-05	2.16E-05	2.38E-05
ASNR	5.32E + 04	5.71E + 04	5.64E + 04	5.91E + 04	5.42E + 04	4.87E + 04	5.37E + 04	5.63E + 04	5.90E + 04	6.37E + 04	4.64E + 04	4.19E + 04
IV	4.21E + 03	3.65E + 03	3.75E + 03	3.40E + 03	4.05E + 03	2.12E + 03	4.08E + 03	3.80E + 03	3.27E + 03	2.93E + 03	4.15E + 03	4.15E + 03
NSD	1.82E + 08	1.82E + 08	1.82E + 08	1.82E + 08	1.82E + 08	7.69E + 07	1.79E + 08	1.84E + 08	1.74E + 08	1.82E + 08	1.36E + 08	1.12E + 08
ENL	8.40E-14	8.40E-14	8.37E-14	8.40E-14	8.40E-14	1.98E-13	8.52E-14	8.29E-14	8.79E-14	8.40E-14	1.12E-13	1.37E-13

**Table 4:** Quality metric for globe lens 4th ventricle

Metrics	Continuum TD	Kuan Filter	Frost Filter	PMAD Filter(15 itrs)	Haar Wavelet	Ordinary Filter Min	Median Filter	Wiener Filter	Average Filter 7 × 7	Gaussian Filter	Laplacian Filter	Laplacian Filter Sharp
AD	9.00E-04	2.57	2.14	4.78	0.0095	17.002	0.9130	2.58	7.15	6.42	4.48	8.77
MSE	0.0035	22.09	18.30	43.75	0.0095	7.89E + 01	7.005	26.11	41.96	43.76	32.03	43.07
RMSE	0.0598	4.70	4.27	6.61	0.0976	8.883875	2.64	5.10	6.47	6.61	5.66	6.56
PSNR	72.58	34.68	35.50	31.72	68.33	29.15	39.67	33.96	31.90	31.71	33.07	31.78
MD	11	130	124	136	1	255	163	74	192	176	168	209
NAE	1.24E-05	0.0353	0.0294	0.0657	1.31E-04	0.2334	0.0125	0.0354	0.0982	0.0881	0.0615	0.1204
NMSE	3.13E-05	0.1629	0.1348	0.3270	6.78E-05	0.5939	0.0502	0.1940	0.3162	0.3271	0.2479	0.3377
SC	1	0.9431	0.9525	0.8708	1	1.15	1	0.9193	0.9297	0.8718	1.06	1.14
CC	1	1	1	0.9856	1	0.9187	1	1	0.9545	0.9655	1	0.9627
NCC	1	1.0126	1.0119	1.0137	1	0.8954	1	1.01	1.006	1.01	0.9438	0.8858
IQI	1	0.9816	0.9534	0.8939	1	0.7445	0.9705	0.9146	1.011	0.9263	0.9705	0.9500
SSIM	1	0.9485	0.9611	0.7866	1	0.7096	0.9760	0.9005	0.7800	0.7667	0.9405	0.8516
CNR	3.65E-07	1.74E-05	4.98E-04	1.20E-04	1.97E-05	0.1443	6.76E-04	2.56E-04	0.0137	3.97E-05	0.0358	0.0700
NI	1.86E-05	1.80E-05	1.81E-05	1.75E-05	1.86E-05	2.11E-05	1.86E-05	1.80E-05	1.78E-05	1.69E-05	1.97E-05	2.10E-05
ASNR	5.38E + 04	5.57E + 04	5.54E + 04	5.70E + 04	5.38E + 04	4.73E + 04	5.38E + 04	5.55E + 04	5.63E + 04	5.92E + 04	5.07E + 04	4.75E + 04
IV	7.88E + 03	7.35E + 03	7.45E + 03	7.01E + 03	7.88E + 03	5.98E + 03	7.85E + 03	7.40E + 03	6.86E + 03	6.50E + 03	7.80E + 03	7.80E + 03
NSD	3.48E + 08	3.48E + 08	3.48E + 08	3.48E + 08	3.48E + 08	2.04E + 08	3.47E + 08	3.48E + 08	3.32E + 08	3.48E + 08	3.06E + 08	2.69E + 08
ENL	4.39E-14	4.39E-14	4.38E-14	4.39E-14	4.39E-14	7.47E-14	4.40E-14	4.39E-14	4.60E-14	4.39E-14	4.98E-14	5.67E-14

**Table 5:** Quality metric for MCA DWI (B 800) MRI

Metrics	Continuum TD	Kuan Filter	Frost Filter	PMAD Filter(15 itrs)	Haar Wavelet	Ordinary Filter Min	Median Filter	Wiener Filter	Average Filter 7 × 7	Gaussian Filter	Laplacian Filter	Laplacian Filter Sharp
AD	1.10E-01	0.8712	0.6221	3.43	1.40	9.56	0.4974	1.48	4.06	2.63	2.55	6.16
MSE	0.1417	3.25	1.83	33.11	7.39	8.33E + 01	1.74	9.00	33.20	24.00	21.71	52.10
RMSE	3.77E-01	1.80	1.35	5.75	2.71	9.12	1.32	3.00	5.76	4.90	4.66	7.21
PSNR	56.61	43.00	45.50	32.93	39.44	28.92	45.71	38.58	32.91	34.32	34.76	30.96
MD	6	17	14	49	16	88	19	25	86	43	74	125
NAE	1.38E-03	0.0109	0.0078	0.0431	1.76E-02	0.1201	0.0062	0.0186	0.0509	0.0331	0.0320	0.0773
NMSE	6.36E-04	0.0182	0.0102	0.1884	4.13E-02	0.4745	0.0097	0.0509	0.1942	0.1354	0.1232	0.2995
SC	1	1	1	0.9796	1	1.08	1	1	1.02	0.9846	1.04	1.08
CC	1	1	1	0.9895	1	0.9876	1	1	0.9883	1	1	0.9859
NCC	1	1	1	1.0094	1	0.9553	1	1	0.9888	1.006	0.9719	0.9316
IQI	0.9837	0.9652	0.9820	0.8440	0.9071	0.8886	0.9677	0.9063	0.8497	0.8627	0.9675	0.9211
SSIM	1	0.9808	0.9882	0.8012	0.9429	0.8641	0.9865	0.9305	0.8513	0.8774	0.9557	0.8396
CNR	2.30E-05	8.24E-05	1.19E-03	6.86E-04	1.43E-04	0.0991	9.49E-05	4.77E-04	0.0185	2.73E-05	0.0256	0.0621
NI	1.35E-05	1.34E-05	1.34E-05	1.29E-05	1.34E-05	1.44E-05	1.34E-05	1.33E-05	1.36E-05	1.31E-05	1.39E-05	1.46E-05
ASNR	7.42E + 04	7.48E + 04	7.48E + 04	7.73E + 04	7.46E + 04	6.95E + 04	7.44E + 04	7.51E + 04	7.35E + 04	7.66E + 04	7.18E + 04	6.87E + 04
IV	4.95E + 03	4.86E + 03	4.88E + 03	4.55E + 03	4.90E + 03	4.36E + 03	4.92E + 03	4.82E + 03	4.81E + 03	4.64E + 03	4.96E + 03	4.92E + 03
NSD	4.16E + 08	4.16E + 08	4.17E + 08	4.15E + 08	4.16E + 08	3.22E + 08	4.16E + 08	4.15E + 08	3.97E + 08	4.16E + 08	3.90E + 08	3.54E + 08
ENL	3.67E-14	3.67E-14	3.66E-14	3.68E-14	3.67E-14	4.74E-14	3.67E-14	3.67E-14	3.84E-14	3.67E-14	3.92E-14	4.31E-14

**Table 6:** Quality metric for MCA FLAIR T2

Metrics	Continuum TD	Kuan Filter	Frost Filter	PMAD Filter (15 itrs)	Haar Wavelet	Ordinary Filter Min	Median Filter	Wiener Filter	Average Filter 7 × 7	Gaussian Filter	Laplacian Filter	Laplacian Filter Sharp
AD	9.16E-02	1.44	1.09	4.42	1.35	11.89	0.7067	1.79	4.89	3.98	3.43	6.82
MSE	0.1395	10.98	7.30	36.46	7.53	8.12E + 01	4.34	14.68	35.23	31.87	31.64	55.42
RMSE	3.74E-01	3.31	2.70	6.03	2.74	9.009	2.08	3.83	5.93	5.64	5.62	7.44
PSNR	56.68	37.72	39.49	32.51	39.36	29.03	41.75	36.46	32.66	33.09	33.12	30.69
MD	8	45	35	92	16	144	54	33	117	87	86	167
NAE	1.46E-03	0.0229	0.0174	0.0704	2.15E-02	0.1891	0.0112	0.0285	0.0777	0.0633	0.0545	0.1085
NMSE	6.58E-04	0.0557	0.0373	0.1987	4.05E-02	0.4380	0.0218	0.0811	0.1987	0.1693	0.1712	0.3114
SC	1	0.9816	0.9837	0.9534	1	1.07	1	0.9752	1	0.9629	1.07	1.15
CC	1	1	1	0.9587	1	0.9354	1	1	0.9406	0.9629	1	0.9662
NCC	1	1	1	1.02	1.002	0.9667	1.002	1.01	1.003	1.021	0.9408	0.8806
IQI	0.9547	0.9205	0.9276	1.11	0.8621	0.6844	0.9293	0.8569	0.8298	0.8931	0.9754	0.9485
SSIM	1	0.9693	0.9796	0.7504	0.9532	0.7988	0.9824	0.9162	0.8089	0.8151	0.9460	0.8299
CNR	1.15E-04	1.20E-04	1.60E-03	4.16E-04	8.22E-05	0.1740	1.54E-03	8.72E-04	0.0206	1.45E-04	0.0472	0.0937
NI	1.23E-05	1.19E-05	1.20E-05	1.10E-05	1.22E-05	1.37E-05	1.22E-05	1.19E-05	1.20E-05	1.11E-05	1.33E-05	1.42E-05
ASNR	8.10E + 04	8.40E + 04	8.35E + 04	9.09E + 04	8.22E + 04	7.33E + 04	8.19E + 04	8.43E + 04	8.36E + 04	9.04E + 04	7.53E + 04	7.05E + 04
IV	2.59E + 03	2.41E + 03	2.45E + 03	2.06E + 03	2.52E + 03	2.08E + 03	2.52E + 03	2.40E + 03	2.32E + 03	2.08E + 03	2.68E + 03	2.72E + 03
NSD	2.59E + 08	2.59E + 08	2.60E + 08	2.59E + 08	2.59E + 08	1.71E + 08	2.59E + 08	2.60E + 08	2.48E + 08	2.59E + 08	2.32E + 08	2.06E + 08
ENL	5.88E-14	5.88E-14	5.86E-14	5.89E-14	5.88E-14	8.94E-14	5.90E-14	5.87E-14	6.16E-14	5.88E-14	6.58E-14	7.40E-14

**Table 7:** Quality metric for MCA PROP T2

Metrics	Continuum TD	Kuan Filter	Frost Filter	PMAD Filter (15 itrs)	Haar Wavelet	Ordinary Filter Min	Median Filter	Wiener Filter	Average Filter 7 × 7	Gaussian Filter	Laplacian Filter (Lap,0,replicate)	Laplacian Filter Sharp (1 1 1; 1 -8 1; 1 1 1)
AD	1.16E-01	1.70	1.36	4.21	1.34	3.76	1.02	1.74	4.68	3.76	4.68	10.22
MSE	0.2539	14.11	10.90	35.23	7.68	3.01E + 01	7.66	13.92	34.28	30.08	39.05	63.84
RMSE	5.04E-01	3.75	3.30	5.93	2.77	5.48	2.76	3.73	5.85	5.48	6.24	7.99
PSNR	54.08	36.63	37.75	32.66	39.27	33.34	39.28	36.69	32.78	33.34	32.21	30.07
MD	24	51	44	82	16	97	56	41	127	97	122	163
NAE	1.71E-03	0.0251	0.020	0.0619	1.97E-02	0.0553	0.0151	0.0255	0.0687	0.0553	0.0687	0.1501
NMSE	1.23E-03	0.0812	0.0625	0.2060	4.41E-02	0.1759	0.0433	0.0788	0.2061	0.1759	0.2282	0.3769
SC	1	0.98	0.9868	0.9634	1	0.9703	1	0.9776	1	0.9703	1.06	1.13
CC	1	1	1	0.9807	1	0.9811	1	1	0.9762	0.9811	0.9885	0.9494
NCC	1	1.006	1.006	1.014	1.00	1.01	1	1	1.001	1.01	0.9543	0.8963
IQI	0.9384	0.8961	0.9128	0.7689	0.8288	0.8151	0.9126	0.8220	0.7995	0.8151	0.9530	0.9242
SSIM	1	0.9528	0.9665	0.7629	0.9546	0.8125	0.9728	0.9142	0.8083	0.8125	0.9152	0.7683
CNR	1.64E-05	1.11E-04	1.28E-03	5.46E-04	3.80E-04	3.78E-05	2.94E-03	1.62E-03	0.0165	3.78E-05	0.0490	0.1073
NI	1.52E-05	1.49E-05	1.49E-05	1.44E-05	1.51E-05	1.44E-05	1.51E-05	1.49E-05	1.50E-05	1.44E-05	1.62E-05	1.76E-05
ASNR	6.58E + 04	6.70E + 04	6.69E + 04	6.94E + 04	6.63E + 04	6.92E + 04	6.62E + 04	6.69E + 04	6.65E + 04	6.92E + 04	6.18E + 04	5.67E + 04
IV	4.61E + 03	4.43E + 03	1.49E-05	4.13E + 03	4.54E + 03	4.16E + 03	4.51E + 03	4.47E + 03	4.30E + 03	4.16E + 03	4.52E + 03	4.48E + 03
NSD	3.04E + 08	3.04E + 08	3.05E + 08	3.04E + 08	3.04E + 08	3.04E + 08	3.01E + 08	3.05E + 08	2.90E + 08	3.04E + 08	2.64E + 08	2.20E + 08
ENL	5.02E-14	5.02E-14	5.00E-14	5.03E-14	5.01E-14	5.02E-14	5.06E-14	5.00E-14	5.26E-14	5.02E-14	5.79E-14	6.95E-14

**Table 8:** Quality metric for acute infarct

Metrics	Continuum TD	Kuan Filter	Frost Filter	PMAD Filter (15 itrs)	Wavelet	Ordinary Filter Min	Median Filter	Wiener Filter	Average Filter 7 × 7	Gaussian Filter	Laplacian Filter	Laplacian Filter Sharp
AD	0.0214	0.5456	0.2986	1.95	0.5211	5.60	0.2165	0.7652	2.67	1.41	1.33	3.29
MSE	0.0228	1.65	0.6689	16.61	1.12	43.44	0.4633	2.86	18.87	10.02	8.76	30.58
RMSE	0.1512	1.28	0.8179	4.07	1.06	6.59	0.6806	1.69	4.34	3.16	2.96	5.53
PSNR	64.54	45.94	49.87	35.92	47.60	31.75	51.47	43.56	35.37	38.12	38.70	33.27
MD	3	107	14	37	6	107	9	14	92	48	45	82
NAE	2.95E-04	0.0075	0.0041	0.0269	0.0072	0.0771	0.0030	0.0105	0.0368	0.0195	0.0183	0.0453
NMSE	1.12E-04	0.0103	0.0040	0.1050	0.0069	0.2775	0.0028	0.0181	0.1242	0.0614	0.0538	0.1980
SC	1	1	0.9965	0.9845	1	1.06	1	0.9975	1.01	0.9906	1.02	1.05
CC	1	1	1	0.9972	1	0.9933	1	1	0.9941	0.9980	1	0.9960
NCC	1	0.9973	1	1	1	0.9635	1	1	0.9912	1	0.9842	0.9581
IQI	0.9891	0.9667	0.9773	0.8180	0.9213	0.8731	0.9726	0.9289	0.8767	0.9113	0.9823	0.9121
SSIM	1	0.9868	0.9938	0.8896	0.9835	0.9159	0.9941	0.9672	0.9264	0.9378	0.9738	0.8898
CNR	2.13E-05	0.0011	8.67E-04	8.90E-04	7.45E-05	0.0518	1.32E-04	1.74E-04	0.0153	1.05E-04	0.0121	0.0301
NI	1.64E-05	1.64E-05	1.64E-05	1.61E-05	1.64E-05	1.72E-05	1.64E-05	1.64E-05	1.66E-05	1.62E-05	1.67E-05	1.70E-05
ASNR	6.08E + 04	6.09E + 04	6.10E + 04	6.19E + 04	6.10E + 04	5.80E + 04	6.09E + 04	6.10E + 04	6.00E + 04	6.17E + 04	5.98E + 04	5.85E + 04
IV	6.02E + 03	5.98E + 03	5.99E + 03	5.80E + 03	6.01E + 03	5.62E + 03	6.01E + 03	5.97E + 03	5.92E + 03	5.85E + 03	5.99E + 03	5.92E + 03
NSD	3.43E + 08	3.42E + 08	3.43E + 08	3.42E + 08	3.42E + 08	2.92E + 08	3.42E + 08	3.42E + 08	3.27E + 08	3.42E + 08	3.30E + 08	3.12E + 08
ENL	4.48E-14	4.50E-14	4.47E-14	4.45E-14	4.48E-14	5.26E-14	4.48E-14	4.48E-14	4.70E-14	4.48E-14	4.65E-14	4.92E-14

**Table 9:** Quality metrics of ischemic demyelination disease

Metrics	Continuum TD	Kuan Filter	Frost Filter	PMAD Filter (15 itrs)	Wavelet	Ordinary Filter Min	Median Filter	Wiener Filter	Average Filter 7 × 7	Gaussian Filter	Laplacian Filter	Laplacian Filter Sharp
AD	0.0353	0.7771	0.4725	2.25	0.8091	5.90	0.4055	0.9487	2.29	1.75	1.91	4.43
MSE	0.0437	3.18	1.64	20.57	2.99	47.85	1.41	4.82	16.07	14.41	15.66	37.11
RMSE	0.2091	1.78	1.28	4.53	1.72	6.91	1.18	2.19	4.01	3.79	3.95	6.09
PSNR	61.72	43.10	45.96	34.99	43.37	31.33	46.62	41.29	36.06	36.54	36.18	32.43
MD	5	138	16	57	11	138	26	19	118	58	64	101
NAE	6.91E-04	0.0152	0.0092	0.0441	0.0158	0.1156	0.0079	0.0185	0.0449	0.0343	0.0374	0.0867
NMSE	2.31E-04	0.0238	0.0122	0.1591	0.0223	0.3724	0.0106	0.0360	0.1288	0.1101	0.1213	0.2936
SC	1	0.9939	0.9887	0.9599	0.9948	1.12	0.9992	0.9821	1	0.9668	1.05	1.09
CC	1	0.9974	0.9995	0.9921	1	0.9885	1	0.9988	0.9915	0.9943	0.9977	0.9868
NCC	1	1	1	1.01	1	0.9388	0.9993	1	0.9923	1.01	0.9657	0.9261
IQI	.99	0.9397	0.9493	0.9737	0.9095	0.6706	0.9380	0.8278	0.8357	0.8664	0.9450	0.8979
SSIM	1	0.9766	0.9873	0.8457	0.9640	0.8839	0.9869	0.9443	0.9131	0.8973	0.9557	0.8546
CNR	2.46E-05	0.0016	0.0012	0.0010	2.68E-04	0.0710	4.81E-04	2.18E-05	0.0138	8.6E-05	0.0222	0.0521
NI	1.83E-05	1.83E-05	1.82E-05	1.78E-05	1.82E-05	1.92E-05	1.83E-05	1.82E-05	1.85E-05	1.79E-05	1.88E-05	1.95E-05
ASNR	5.45E + 04	5.47E + 04	5.48E + 04	5.59E + 04	5.46E + 04	5.18E + 04	5.46E + 04	5.48E + 04	5.39E + 04	5.56E + 04	5.30E + 04	5.12E + 04
IV	3.72E + 03	3.67E + 03	3.68E + 03	3.51E + 03	3.69E + 03	3.21E + 03	3.69E + 03	3.67E + 03	3.62E + 03	3.56E + 03	3.64E + 03	3.50E + 03
NSD	1.69E + 08	1.69E + 08	1.70E + 08	1.69E + 08	1.70E + 08	1.32E + 08	1.69E + 08	1.69E + 08	1.62E + 08	1.69E + 08	1.57E + 08	1.42E + 08
ENL	9.05E-14	9.10E-14	9.01E-14	9.08E-14	9.04E-14	1.15E-13	9.06E-14	9.05E-14	9.48E-14	9.05E-14	9.76E-14	1.09E-13

**Table 10:** Quality metrics of cerebral infarct

Metrics	Continuum TD	Kuan Filter	Frost Filter	PMAD Filter (15 itrs)	Wavelet	Ordinary Filter Min	Median Filter	Wiener Filter	Average Filter 7 × 7	Gaussian Filter	Laplacian Filter	Laplacian Filter Sharp
AD	0.0134	0.3700	0.2385	1.81	0.4802	4.52	0.1733	0.6146	2.10	1.21	1.22	2.97
MSE	0.0137	0.8863	0.4715	14.71	0.9813	36.69	0.3560	1.89	15.07	8.07	6.81	26.14
RMSE	0.1171	0.9414	0.6867	3.83	0.9906	6.05	0.5967	1.37	3.88	2.84	2.61	5.11
PSNR	66.75	48.65	51.39	36.45	48.21	32.48	52.61	45.35	36.35	39.05	39.79	33.95
MD	3	15	12	44	6	79	12	14	90	37	57	87
NAE	2.68E-04	0.0074	0.0048	0.0361	0.0096	0.0904	0.0035	0.0123	0.0420	0.0241	0.0244	0.0595
NMSE	6.04E-05	0.0053	0.0028	0.0913	0.0061	0.2283	0.0021	0.0120	0.0981	0.0486	0.0410	0.1674
SC	1	0.9959	0.9942	0.9689	1	1.04	1	0.9921	1.01	0.9818	1.02	1.06
CC	1	0.9997	0.9998	0.9941	0.9997	0.9925	1	0.9993	0.9924	0.9969	0.9989	0.9934
NCC	1	1	1	1.01	0.9997	0.9754	0.9997	1	0.9915	1	0.9850	0.9567
IQI	0.9805	0.9390	0.9355	0.7695	0.8484	0.8259	0.9490	0.8457	0.8403	0.8396	0.9133	0.8523
SSIM	1	0.9912	0.9947	0.8979	0.9847	0.9353	0.9957	0.9759	0.9299	0.9485	0.9742	0.8962
CNR	1.84E-06	1.35E-04	0.0015	7.06E-04	2.37E-04	0.0620	1.10E-04	3.72E-05	0.0155	5.81E-05	0.0163	0.0399
NI	1.63E-05	1.62E-05	1.62E-05	1.58E-05	1.63E-05	1.69E-05	1.63E-05	1.62E-05	1.65E-05	1.60E-05	1.66E-05	1.71E-05
ASNR	6.13E + 04	6.15E + 04	6.16E + 04	6.30E + 04	6.14E + 04	5.90E + 04	6.14E + 04	6.16E + 04	6.05E + 04	6.25E + 04	6.00E + 04	5.84E + 04
IV	2.82E + 03	2.79E + 03	2.80E + 03	2.66E + 03	2.81E + 03	2.51E + 03	2.81E + 03	2.78E + 03	2.75E + 03	2.71E + 03	2.80E + 03	2.75E + 03
NSD	1.63E + 08	1.63E + 08	1.63E + 08	1.63E + 08	1.63E + 08	1.34E + 08	1.63E + 08	1.63E + 08	1.55E + 08	1.63E + 08	1.55E + 08	1.44E + 08
ENL	9.44E-14	9.44E-14	9.39E-14	9.45E-14	9.43E-14	1.14E-13	9.44E-14	9.43E-14	9.89E-14	9.44E-14	9.91E-14	1.07E-13

**Table 11:** Quality metrics of neoplastic lesion 1 glioma

Metrics	Continuum TD	Kuan Filter	Frost Filter	PMAD Filter(15 itrs)	Haar Wavelet	Ordinary Filter Min	Median Filter	Wiener Filter	Average Filter 7 × 7	Gaussian Filter	Laplacian Filter	Laplacian Filter Sharp
AD	0.1539	1.71	1.42	4.06	1.37	10.67	1.19	1.65	4.39	3.52	4.49	8.69
MSE	0.3562	14.01	11.27	32.37	8.04	75.72	9.09	13.20	33.17	27.92	38.51	63.25
RMSE	0.5968	3.74	3.35	5.69	2.83	8.70	3.01	3.63	5.76	5.28	6.20	7.95
PSNR	52.61	36.66	37.61	33.03	39.07	29.33	38.54	36.92	32.92	33.67	32.27	30.12
MD	23	53	45	104	16	154	57	38	101	97	101	175
NAE	0.0032	0.0360	0.0298	0.0855	0.0288	0.2246	0.0250	0.0348	0.0924	0.0742	0.0945	0.1828
NMSE	0.0017	0.0764	0.0612	0.1816	0.0452	0.4241	0.0506	0.0762	0.1931	0.1550	0.2216	0.3710
SC	1	0.9794	0.9814	0.9177	0.9913	1.11	0.9957	0.9693	0.9839	0.9370	1.07	1.17
CC	1	0.9914	0.9936	0.9592	0.9964	0.9332	0.9955	0.9936	0.9444	0.9640	0.9770	0.9342
NCC	1	1	1	1.02	1	0.9416	1	1	1	1.02	0.9469	0.8718
IQI	0.9557	0.9246	0.9150	0.9204	0.8644	0.6707	0.9365	0.8660	0.8622	0.8589	0.9456	0.9352
SSIM	1	0.9411	0.9564	0.7368	0.9480	0.7241	0.9634	0.9084	0.7876	0.7993	0.8918	0.7414
CNR	2.15E-06	1.52E-04	0.0018	0.0010	5.31E-04	0.1783	0.0071	0.0030	0.0175	7.15E-05	0.0698	0.1348
NI	1.47E-05	1.42E-05	1.42E-05	1.31E-05	1.45E-05	1.62E-05	1.45E-05	1.42E-05	1.42E-05	1.33E-05	1.62E-05	1.80E-05
ASNR	6.78E + 04	7.03E + 04	7.00E + 04	7.60E + 04	6.88E + 04	6.17E + 04	6.86E + 04	7.03E + 04	7.04E + 04	7.50E + 04	6.15E + 04	5.53E + 04
IV	2.07E + 03	1.93E + 03	1.95E + 03	1.64E + 03	2.01E + 03	1.51E + 03	1.98E + 03	1.94E + 03	1.84E + 03	1.69E + 03	2.07E + 03	2.08E + 03
NSD	1.47E + 08	1.47E + 08	1.47E + 08	1.47E + 08	1.47E + 08	8.83E + 07	1.44E + 08	1.48E + 08	1.40E + 08	1.47E + 08	1.20E + 08	9.81E + 07
ENL	1.04E-13	1.04E-13	1.04E-13	1.04E-13	1.04E-13	1.74E-13	1.06E-13	1.03E-13	1.09E-13	1.04E-13	1.27E-13	1.56E-13

**Table 12:** Quality Metrics for neoplastic lesion 2—4th ventricle tumour

Metrics	Continuum TD	Kuan Filter	Frost Filter	PMAD Filter(15 itrs)	Haar Wavelet	Ordinary Filter Min	Median Filter	Wiener Filter	Average Filter 7 × 7	Gaussian Filter	Laplacian Filter	Laplacian Filter Sharp
AD	0.1211	1.69	1.37	4.14	1.37	10.77	1.11	1.67	4.11	3.59	4.39	9.12
MSE	0.2573	13.60	10.75	32.61	8.16	74.80	8.42	13.42	30.89	28.06	36.91	62.32
RMSE	0.5072	3.68	3.27	5.71	2.85	8.64	2.90	3.66	5.55	5.30	6.07	7.89
PSNR	54.03	36.79	37.81	32.99	39.01	29.39	38.87	36.85	33.23	33.65	32.46	30.18
MD	14	57	52	88	16	183	81	35	123	109	110	159
NAE	0.0023	0.0328	0.0264	0.0799	0.0264	0.2079	0.0213	0.0322	0.0794	0.0693	0.0847	0.1760
NMSE	0.0013	0.0716	0.0562	0.1798	0.0456	0.4178	0.0456	0.07780	0.1769	0.1519	0.2094	0.3619
SC	1	0.9773	0.9802	0.9323	0.9911	1.09	0.9950	0.9733	0.9862	0.9429	1.05	1.14
CC	1	0.9916	0.9940	0.9583	0.9966	0.9301	0.9961	0.9939	0.9567	0.9618	0.9798	0.9256
NCC	1	1	1	1.02	1	0.9459	1	1	1	1.02	0.9606	0.8931
IQI	0.9396	0.8977	0.9120	0.7645	0.8399	0.7145	0.9128	0.8243	0.8212	0.8086	0.9242	0.8703
SSIM	1	0.9471	0.9628	0.7397	0.9496	0.7405	0.9704	0.9097	0.8095	0.8013	0.9002	0.7386
CNR	1.81E-05	2.83E-04	0.0019	0.0012	2.99E-04	0.1757	0.0062	0.0026	0.0185	3.48E-05	0.0665	0.1379
NI	1.39E-05	1.34E-05	1.34E-05	1.27E-05	1.36E-05	1.48E-05	1.37E-05	1.34E-05	1.34E-05	1.26E-05	1.50E-05	1.68E-05
ASNR	7.19E + 04	7.47E + 04	7.44E + 04	8.02E + 04	7.31E + 04	6.73E + 04	7.30E + 04	7.43E + 04	7.41E + 04	7.93E + 04	6.63E + 04	5.94E + 04
IV	2.19E + 03	2.03E + 03	2.06E + 03	1.76E + 03	2.12E + 03	1.57E + 03	2.09E + 03	2.06E + 03	1.97E + 03	1.80E + 03	2.15E + 03	2.18E + 03
NSD	1.74E + 08	1.74E + 08	1.75E + 08	1.74E + 08	1.75E + 08	1.09E + 08	1.72E + 08	1.75E + 08	1.67E + 08	1.75E + 08	1.46E + 08	1.18E + 08
ENL	8.80E-14	8.81E-14	8.76E-14	8.82E-14	8.80E-14	1.40E-13	8.94E-14	8.75E-14	9.22E-14	8.80E-14	1.05E-13	1.29E-13

**Table 13:** Quality metrics for neoplastic lesion Ax T1 FLAIR glioma

Metrics	Continuum TD	Kuan Filter	Frost Filter	PMAD Filter(15 itrs)	Haar Wavelet	Ordinary Filter Min	Median Filter	Wiener Filter	Average Filter 7 × 7	Gaussian Filter	Laplacian Filter	Laplacian Filter Sharp
AD	0.0907	1.01	0.7424	3.24	0.9452	7.62	0.5592	1.20	3.59	2.36	3.03	6.76
MSE	0.1522	5.48	3.58	28.48	3.48	63.03	2.71	6.01	26.69	20.25	26.32	55.34
RMSE	0.3902	2.34	1.89	5.33	1.87	7.93	1.64	2.45	5.16	4.50	5.13	7.43
PSNR	56.30	40.73	42.58	33.58	42.70	30.13	43.80	40.34	33.86	35.06	33.92	30.70
MD	12	56	45	109	11	114	70	22	115	106	67	118
NAE	0.0014	0.0156	0.0115	0.0501	0.0146	0.1178	0.0086	0.0186	0.0555	0.0365	0.0467	0.1045
NMSE	7.55E-04	0.0298	0.0196	0.1462	0.0185	0.3283	0.0147	0.0309	0.1482	0.1045	0.1385	0.2937
SC	1	0.9921	0.9906	0.9622	0.9955	1.05	1	0.9870	1	0.9715	1.05	1.12
CC	1	0.9974	0.9984	0.9788	0.9985	0.9758	0.9987	0.9974	0.9726	0.9865	0.9927	0.9676
NCC	1	1	1	1.02	1	0.9762	1	1	0.9988	1.01	0.9629	0.9046
IQI	0.9410	0.9235	0.9109	0.7746	0.8514	0.0828	0.9198	0.7457	0.8106	0.8740	0.9093	0.5315
SSIM	0.9977	0.9694	0.9809	0.8012	0.9675	0.8551	0.9821	0.9459	0.8413	0.8732	0.9309	0.7741
CNR	1.24E-05	3.10E-04	0.0018	0.0022	2.52E-04	0.1142	8.96E-04	1.11E-04	0.0221	2.76E-06	0.0441	0.0979
NI	1.14E-05	1.13E-05	1.13E-05	1.08E-05	1.13E-05	1.24E-05	1.14E-05	1.13E-05	1.15E-05	1.10E-05	1.21E-05	1.31E-05
ASNR	8.73E + 04	8.84E + 04	8.84E + 04	9.18E + 04	8.78E + 04	8.05E + 04	8.76E + 04	8.85E + 04	8.69E + 04	9.09E + 04	8.21E + 04	7.62E + 04
IV	2.33E + 03	2.26E + 03	2.27E + 03	2.09E + 03	2.30E + 03	2.12E + 03	2.30E + 03	2.26E + 03	2.24E + 03	2.14E + 03	2.39E + 03	2.44E + 03
NSD	2.72E + 08	2.72E + 08	2.73E + 08	2.71E + 08	2.72E + 08	2.12E + 08	2.72E + 08	2.72E + 08	2.60E + 08	2.72E + 08	2.47E + 08	2.18E + 08
ENL	5.64E-14	5.64E-14	5.62E-14	5.67E-14	5.64E-14	7.25E-14	5.65E-14	5.64E-14	5.91E-14	5.64E-14	6.21E-14	7.03E-14

**Fig. 2:**
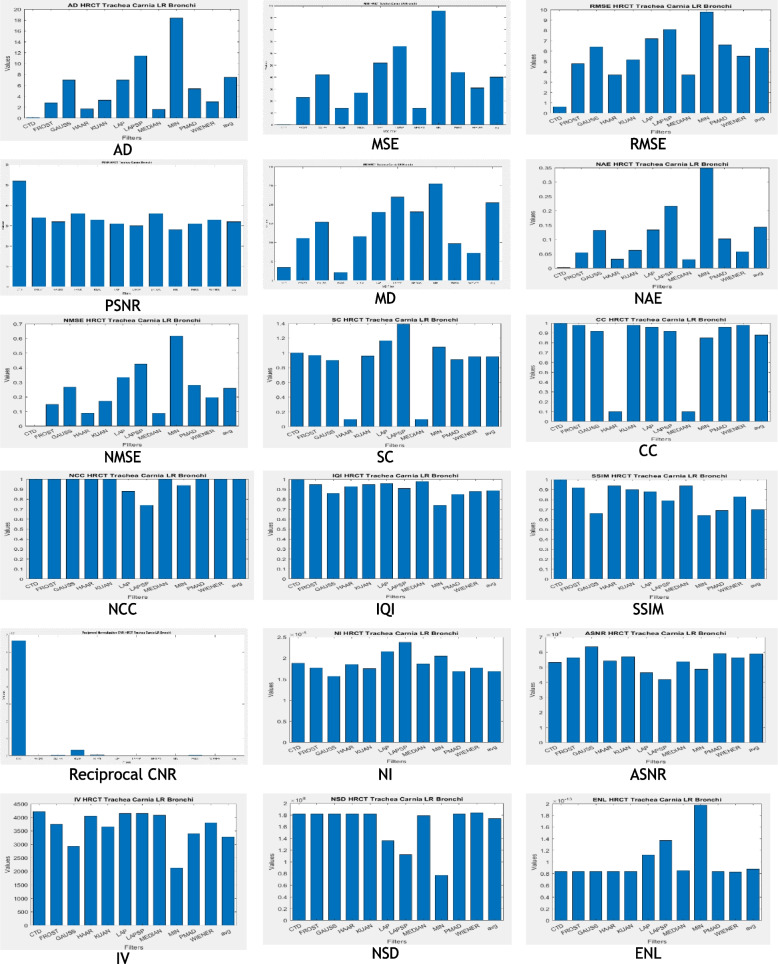
Histogram plots of the quality metrics of Carina

### Clinical example 1: HRCT thoracic cavity carina

The study focuses on the role of Carina in chest CT imaging for detecting tracheobronchial carcinoid tumors [124]. Quantum mottle noise in CT images often obscures the carina morphology due to vascular matter in the lungs. The proposed denoising technique (CTD) effectively addresses this issue, producing clear and high-resolution images. Inferences from HRCT carina filtered images, metrics, and histogram plots (Fig. 1, Table 3 and Fig. 2) are summarized as follows:

- CTD exhibited significantly lower values in AD, MSE, RMSE, MD, NAE, and NMSE compared to traditional filters, showcasing exceptional denoising capability, particularly in reducing quantum mottle noise. Radiographers noted the CTD denoised image’s ability to delineate the carina point with complete opacity on both sides, aiding surgeons and anesthesiologists in accurate findings for post-pneumonectomy-like syndrome severity.
- SC, CC, NCC, IQI, and SSIM returned unity values for CTD, retaining all structural information in carina HRCT, unlike other filters. The cartilaginous ridge shape of the carina and the surrounding lungs cavity were exceptionally retained in the denoised CTD image.
- PSNR recorded a 30% higher value for CTD, attesting to its ability to produce a high-quality, quantum mottle-free carina image. CNR, NI, ASNR, IV, NSD, and ENL scores were notable for CTD, highlighting its fine contrast, noise-free, and radiologically preserved carina image.
- Ultimately, the CTD denoised image accurately retained radiological features of the carina while eliminating quantum mottle noise, as confirmed by visual inspection from a radiological expert.

### Clinical example 2: Ocular globe-lens 4th ventricle head CT

**Fig. 3:**
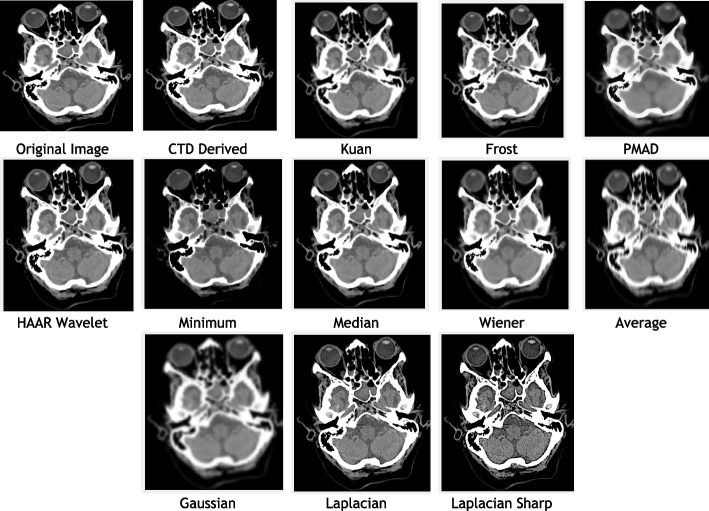
Original and denoised Images of Globe Lens 4th Ventricle

The cerebral ventricular system, a protective unit filled with cerebrospinal fluid, indicates brain health. Deformities in this system signal potential brain diseases [125]. The system comprises four ventricles, with the fourth ventricle located between the cerebellum and pons/medulla. In CT imaging, an irregularly shaped fourth ventricle may indicate meningioma tumours [126, 127]. The ocular globe’s structure is crucial in cases like orbital trauma and retinoblastoma [128, 129]. A CTD denoised image impressed a radiographer, showcasing improved visualization of the pyramidal structure of the fourth ventricle and enhanced details of the ocular globe, including its fibrous, vascular, and neural layers. The radiologist anticipates accurate detection of deformities and diseases by addressing hidden quantum mottle noise. The study focuses on CT images of the fourth ventricle, globe, and lens. Denoised images, including CTD and traditional filters, are presented in Fig. 3. The structurally preserved image will greatly assist ophthalmic surgeons and neuro-physicians in clinical treatment decisions, whether through surgery or non-invasive methods.

**Fig. 4:**
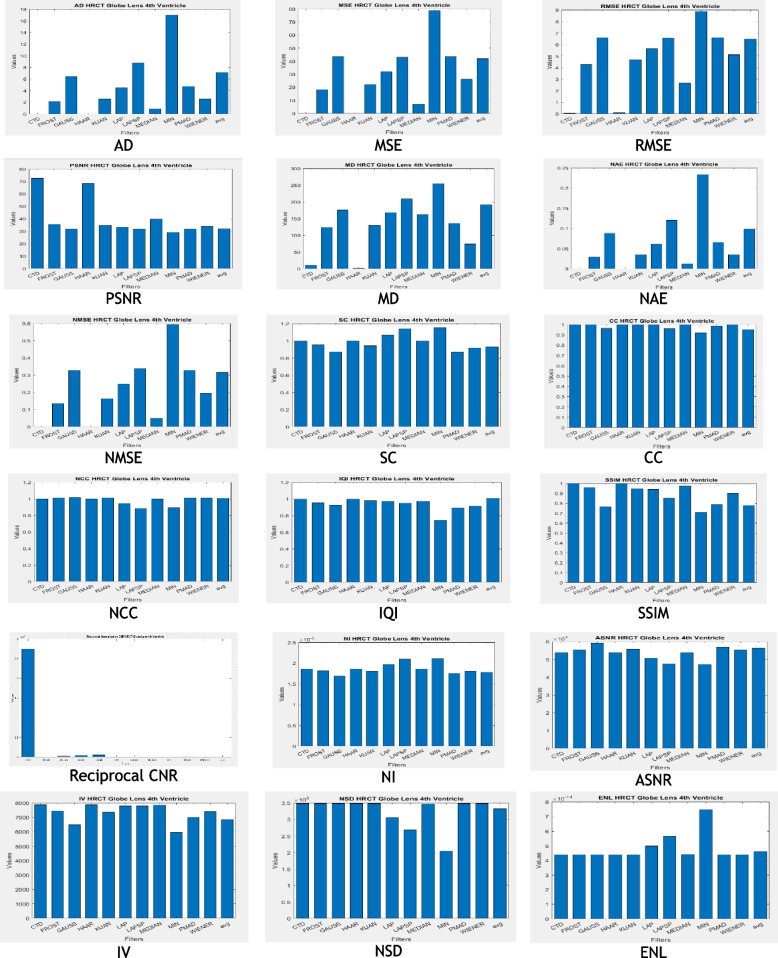
Histogram plots of the performance metrics of Globe Lens 4th Ventricle

Observations from brain HRCT Globe Lens 4th Ventricle quality metrics and histogram plots (Table 4 and Fig. 4):

- CTD filter yielded minimum values in AD, MSE, RMSE, MD, NAE, and NMSE, generating a high-quality denoised image useful for precise location identification and deformation needs in the globe, lens, and 4th ventricle, due to the selection efficiency of appropriate cost-function and subsequent computation of topological derivative.
- Quality metrics SC, CC, NCC, IQI, and SSIM, indicators of shape and structure similarity, reached unity for CTD, highlighting its merit in retaining original shape and preserving structural details of aperture nature, unlike other traditional filters.
- PSNR recorded a 50% higher value for CTD, demonstrating superior denoising ability over other filters, except Haar wavelet.
- CNR, NI, ASNR, IV, NSD, and ENL values for CTD-filtered image surpassed those of other filters, ensuring fine contrast, noise-free, and radiologically preserved images for Globe-Lens 4th Ventricle.

### Clinical example 3: MRI brain-middle cerebral artery territory DWI (B 800)

The Middle Cerebral Artery (MCA), the largest terminal branch of the internal carotid artery, supplies blood to the brain, traversing through distinct segments (M1 to M4). CT images reveal MCA segment discontinuity, while MR images disclose Circle of Willis anomalies. Radiologically, Moyamoya syndrome, aneurysm formation, intracranial hemorrhage, and vascular variations differentiate rare rete MCA anomalies. Gaussian and Rayleigh noises in images may lead to misdiagnosis [130]. Besides, void signals in T2-MRI wittnessed in twig like MCA (T-MCA) for diagnoising hemodynmic delay, intracranial aneursym, internal carotid artery and trandsural anastomosis conditions in MCA is very difficult due to the presence of Gaussian and Raleigh’s noises [131–137].

T-MCA anomalies are challenging due to void signals in T2-MRI, making Diffusion-weighted imaging (DWI) and Fluid-attenuated inversion recovery (FLAIR) essential. PROPELLER MRI reduces motion artifacts [138–140]. Despite advanced MRI modes, Gaussian and Rayleigh noises persist, necessitating denoising for accurate MCA diagnosis.

**Fig. 5:**
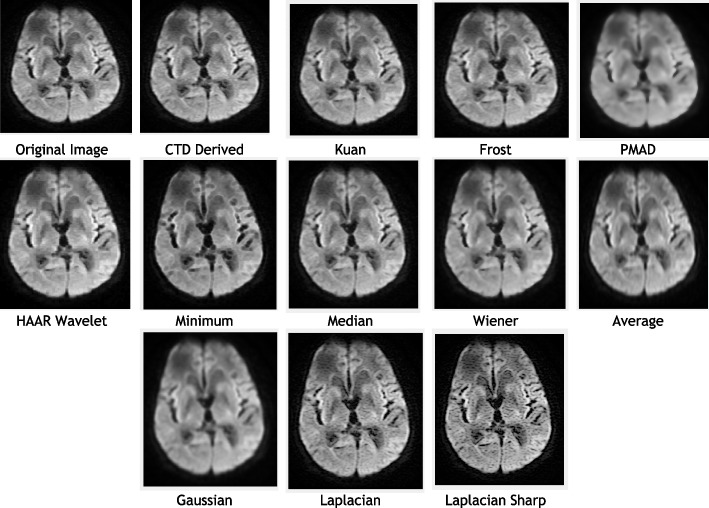
Original and denoised Images of MCA DWI (B 800) MRI

**Fig. 6:**
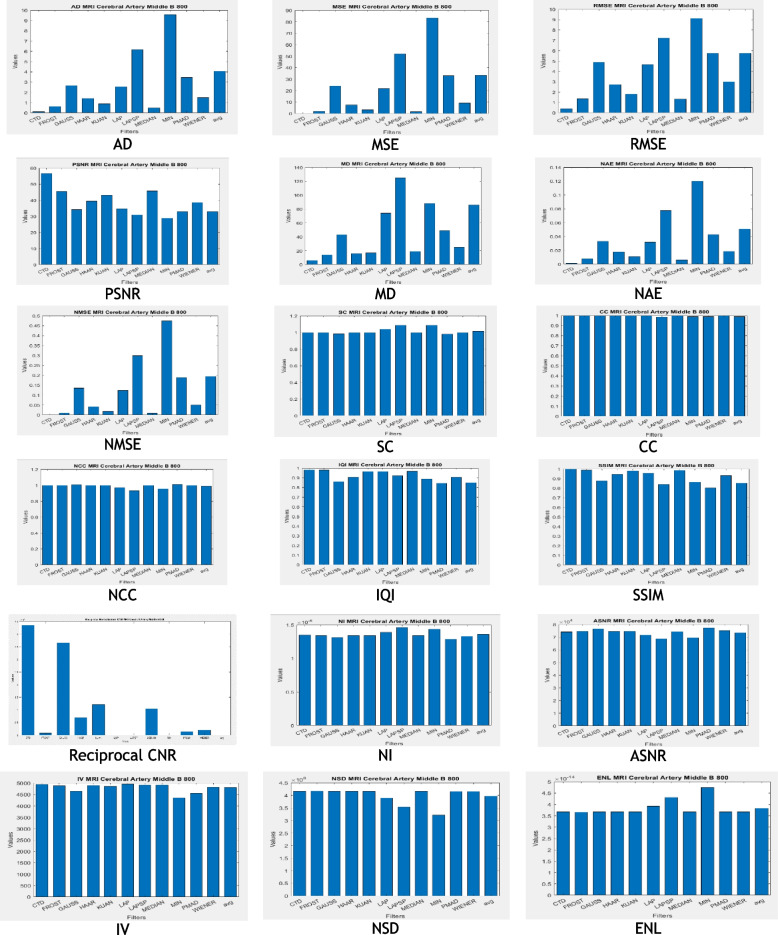
Histogram plots of the quality metrics for MCA DWI (B 800) MRI image

For Brain-MCA territory DWI (B 800) MRI, CTD and traditional filters were applied, displaying outputs in Fig. 5. Quality metrics and histogram plots are in Table 5 and Fig. 6. Radiologists noted CTD’s distinguishable features in MCA territory, aiding easy detection of irregularities. Shape derivatives from CTD support neurologists in clinically and surgically assessing MCA abnormalities and severity.

### Clinical example 4: MRI brain—middle cerebral artery territory FLAIR T2

**Fig. 7:**
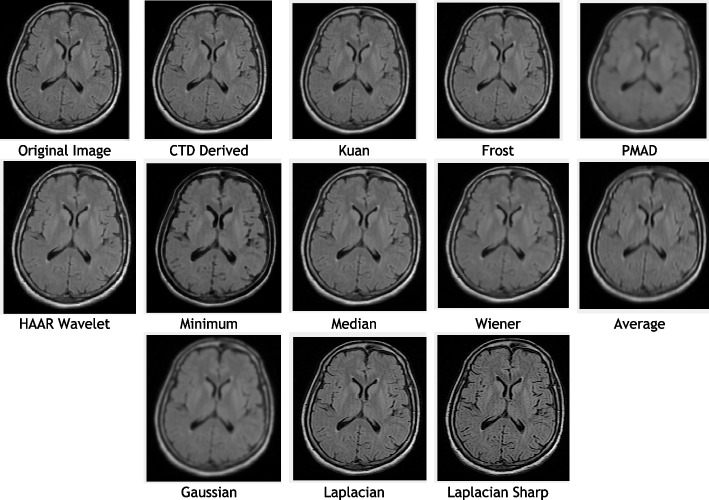
Original and Denoised images of MRI MCA FLAIR T2

**Fig. 8:**
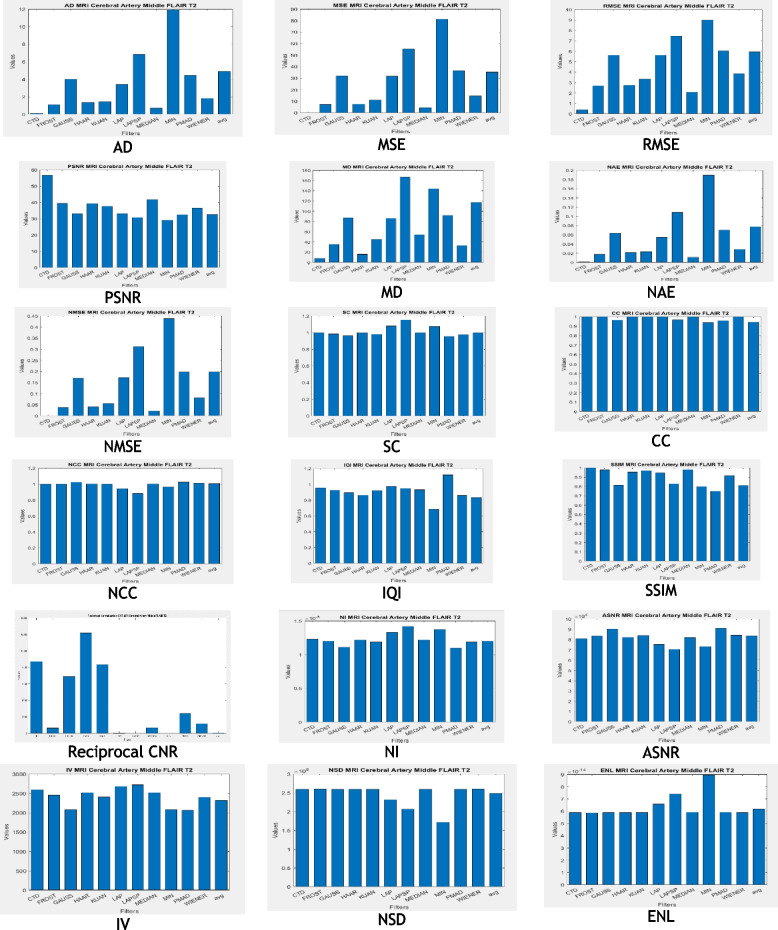
Histogram plots of the performance metrics for brain MCA FLAIR T2 MR Image

Similarly, the Brain-MCA territory FLAIR T2 MR territory image underwent testing with both the proposed and traditional algorithms, as illustrated in Fig. 7. A summary of the quality metrics computed for all denoising algorithms is provided in Table 6, with their corresponding histogram plots displayed in Fig. 8. These results consistently demonstrate the effectiveness of the proposed CTD techniques in denoising.

### Clinical example 5: MRI brain—middle cerebral artery territory PROP T2

**Fig. 9:**
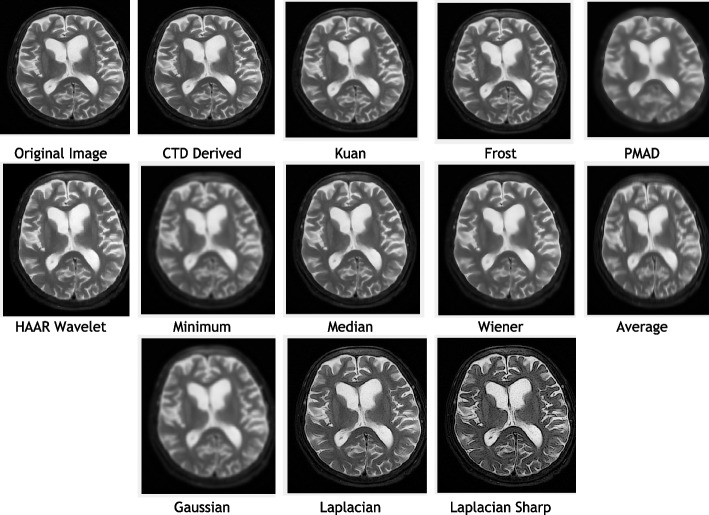
Original and denoised images of MRI MCA PROP T2

**Fig. 10:**
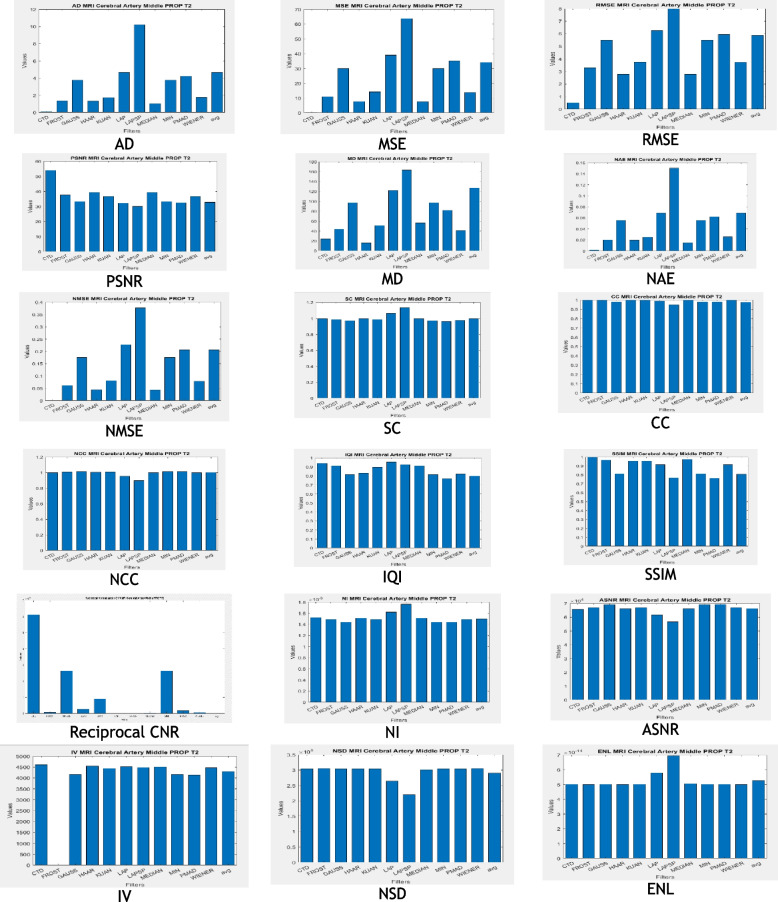
Histogram plots of the performance metrics of brain MCA PROP T2

Similarly, the Brain-MCA territory PROP T2 MR territory image underwent testing with various denoising algorithms, and the outcomes are illustrated in Fig. 9. Corresponding performance metrics and their histogram plots are detailed in Table 7 and Fig. 10, respectively. Altogether, following conclusions have been drawn for all of MCA territory MR images i.e., DWI (B 800), FLAIR T2 and PROP T2:

- The CTD method, indicated by low scores in AD, MSE, RMSE, MD, NAE, and NMSE, strategically tuned the cost-function, effectively removing Gaussian and Rayleigh noises from Brain MCA territory DWI (B 800), FLAIR T2, and PROP T2 MR images. This denoising capability of the proposed CTD technique produces high-quality images crucial for assessing subtle changes in the MCA territory, quantifying injury severity accurately.
- SC, CC, NCC, IQI, and SSIM, critical factors for similarity indices, consistently reached unity for CTD, indicating full retention of MCA territory after denoising. Deviations from the original MCA territory serve as strong indicators for acute stroke, accurately identified in CTD-filtered images, aiding physicians in detecting metastatic brain conditions in MCA regions.
- PSNR returned noteworthy values for CTD, justifying its effectiveness in removing Gaussian and Rayleigh noise in MCA territory DWI (B 800), FLAIR T2 and PROP T2 MR images.
- CNR, NI, ASNR, IV, NSD, and ENL metrics recorded noteworthy values for CTD, resulting in improved contrast, noise-free, and distinctly preserved territorial regions of MCA territory DWI (B 800), FLAIR T2, and PROP T2 MR images.

In all clinical examples (1–5), the relative residual value is extremely low, indicating that the CTD algorithm converges with a minimum number of iterations, generating negligible errors, and ensuring very good quality in denoised images. Further analysis of the CTD algorithm for diseased regions of MCA territory will be presented in clinical examples 6, 7, and 8 given below.

### Clinical Examples 6, 7 and 8: Infarct and Demyelination

**Fig. 11:**
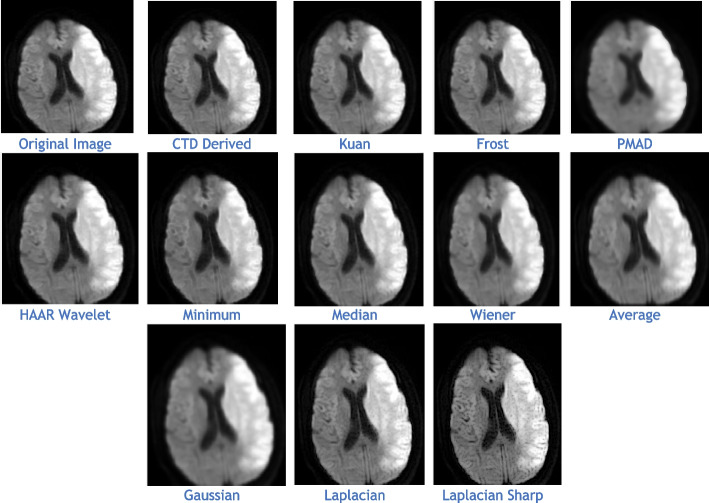
Examination of Acute Infarct images obtained by application of various denoising filters

**Fig. 12:**
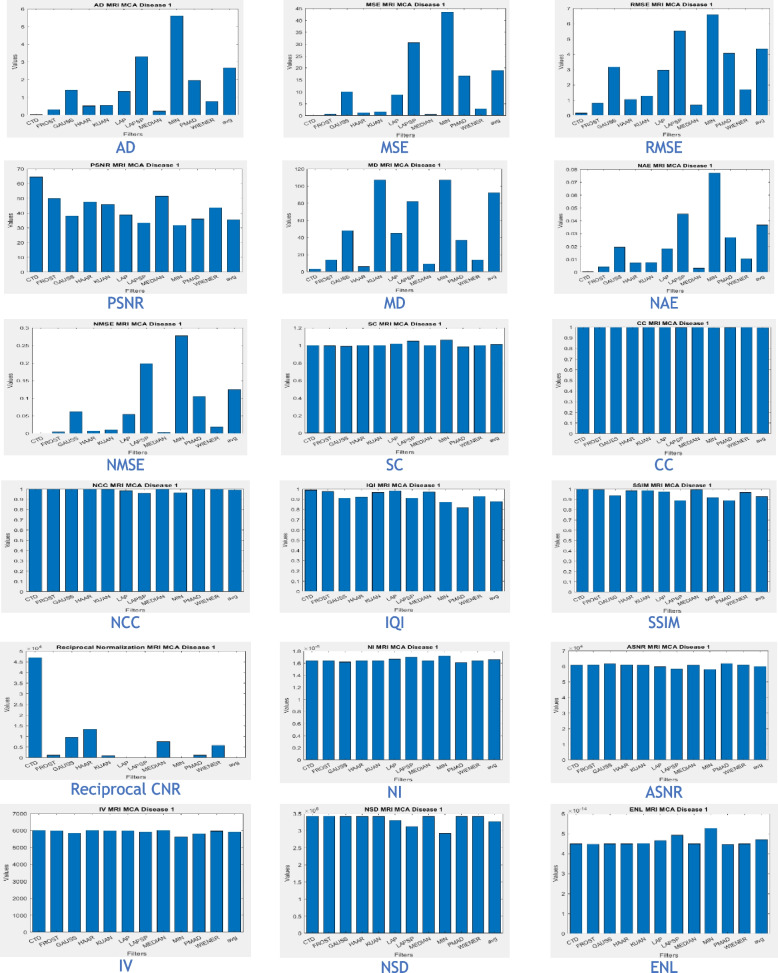
Histogram plots of the performance metrics of acute cerebral infarct

**Fig. 13:**
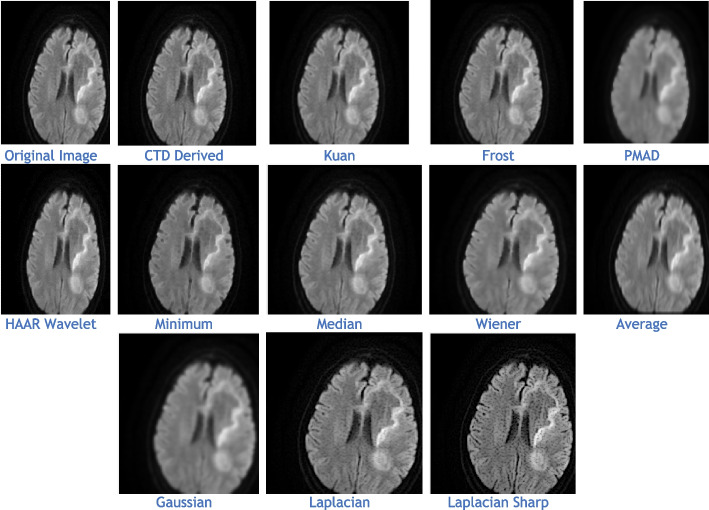
Denoised images of the brain MRI of Ischemic Demyelination disease for various filters and the proposed CTD filter

**Fig. 14:**
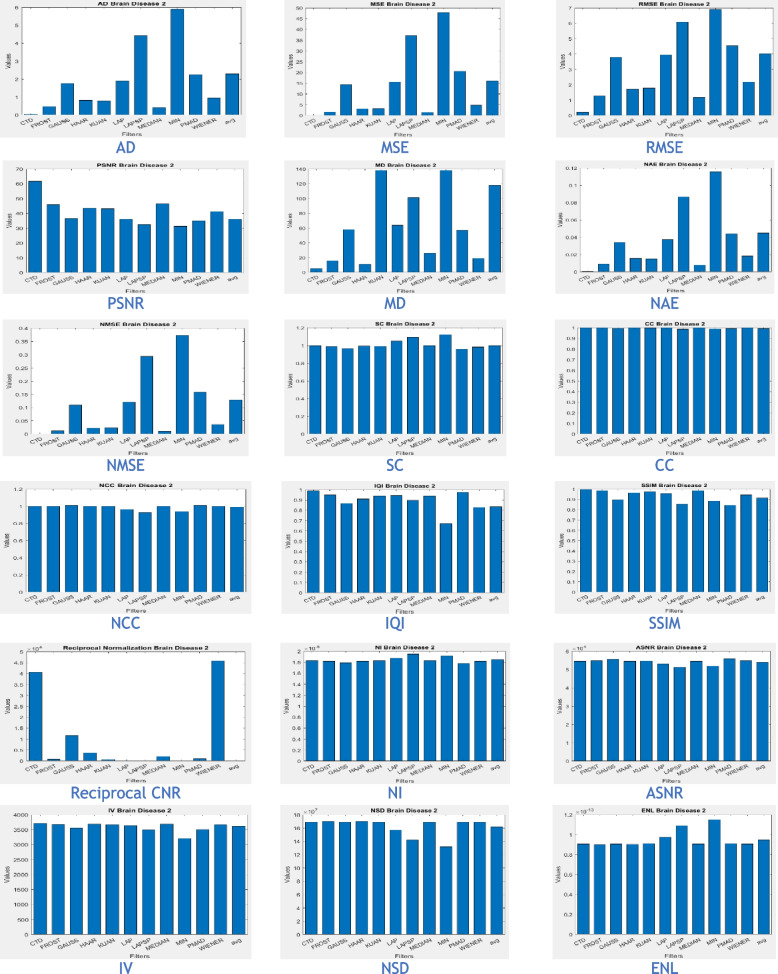
Histogram plots of the performance metrics of Ischemic Demyelination Disease

**Fig. 15:**
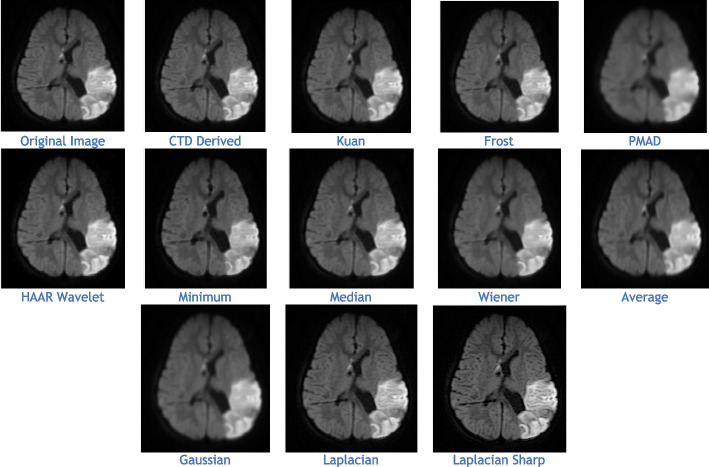
Denoised output images of cerebral infarct disease of different types of filters and the CTD filter

**Fig. 16:**
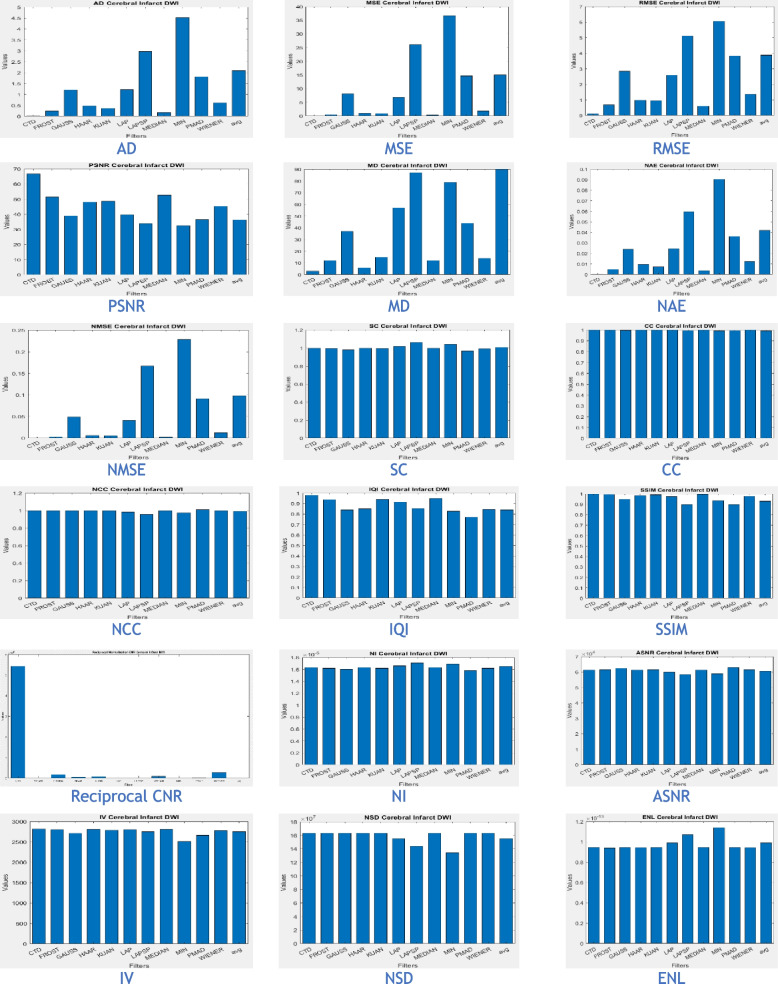
Histogram plots of the performance metrics of cerebral infarct

This section presents a detailed analysis of brain regions using CTD and other denoising filters (Figs. 11, 13, and 15). Performance metrics from these filters are reported in Tables 8, 9, and 10, with their distribution visualized in histogram plots (Figs. 12, 14, and 16). The results offer insights into the filters’ effectiveness in improving image quality and detecting brain diseased region.

Figure 11 presents MR images obtained through DWI, known for its ability to provide excellent lesion contrast and differentiate strokes from conditions mimicking strokes. DWI identifies diffusion restriction, indicating acute infarct presence, and effectively visualizes cerebral infarction volume. It consistently delivers excellent results from early lesion stages to full infarction, aiding prognosis in stroke patients’ follow-up scans.

DWI also detects vasogenic edema and acute lesions in chronic ischemic cases, offering insights into brain physiology. Notably, the CTD-derived acute infarct DWI image displayed exceptional quality, similarity, and noise metrics (Table 8, Fig. 12), emphasizing enhanced contrast and diffusion aspects. Thus, neuro physicians and radiologists can rely on CTD-enhanced images for accurate stroke detection and brain damage assessment.

The denoised images illustrate MR images obtained using the DWI technique, with various applied filters (Fig. 13). While DWI is commonly used for infarct cases, it plays a crucial role in identifying Ischemic Demyelination cases, distinguishing between vascular ischemia (restricted diffusion) and demyelination (facilitated diffusion). This sequence proves advantageous in characterizing tissue and histopathology in cases of ischemic demyelination disease. In this rare case, CTD demonstrated its uniqueness through performance values (Table 9) and histogram plots (Fig. 14). CTD-derived images excel in producing enhanced representations of demyelination diseases, showing a high degree of structural similarity (unit values for SC, CC, NCC, IQI, and SSIM). This characteristic is critical when analyzing images with restricted diffusion.

Figure 15 displays MR images obtained using the DWI technique, processed with various denoising filters. DWI identifies ischemic changes in brain tissue, represented as hyperintensity in MR DWI images. Cerebral Small Vessel Disease (SVD), characterized by nil restricted diffusion, is an indicator for cerebral infarct. The CTD cerebral infarct image in Fig. 15 stands out for exceptional denoising quality, enhancing hyperintensity regions significantly, as indicated by the outstanding PSNR value. Minimal values in both quality and noise metrics (Table 10) and corresponding histogram plots (Fig. 16) affirm the effectiveness of this denoising approach. Additionally, the hyperintensity signals in this CTD image exhibit no noticeable distortion, underscoring the reliability and accuracy of the denoising process employed in generating the enhanced image.

Inferences from filtered images, metrics, and histogram plots for infarct and Ischemic Demyelination cases are outlined below:

- Acute and Cerebral Infarct DWI:
  a AD, MSE, RMSE, MD, NAE, and NMSE demonstrate remarkably low values compared to other filters, indicating effective denoising of Gaussian and Rayleigh noises. The CTD technique enhances hyperintense areas, delivering high-quality images for accurate brain infarct identification, showcasing superior denoising capabilities.
  b SC, CC, NCC, and SSIM yield unity for CTD, with IQI slightly below unity, a unique characteristic. Other filters show metric values either lower or higher than unity, indicating that CTD retains similarity in infarct brain MRI images with high accuracy after denoising.
  c The PSNR value for CTD surpasses other filters, showcasing its effectiveness in removing both foreground and background noise from diseased brain images.
  d CNR, NI, ASNR, IV, NSD, and ENL metrics exhibit favorable values for CTD, ensuring enhanced contrast, noise-free results, and well-preserved radiological features in brain infarct MRI images.
- Ischemic Demyelination Disease:
  a AD, MSE, RMSE, MD, NAE, and NMSE show notably low values compared to other filters, indicating effective denoising of Gaussian and Rayleigh noises in the brain affected by Ischemic Demyelination Disease. The CTD-derived MRI image differentiates hypo and hyper intense areas excellently, providing crucial information about demyelination and ischemic changes in the diseased brain.
  b SC, CC, NCC, IQI, and SSIM all achieve unity for the CTD filter, while other filters show values greater than or less than unity. The CTD denoised image returns a highly structured image with zero error, enabling easy observation of subtle shifts in ischemic demyelination within the brain tissue.
  c The PSNR returns an excellent value for the CTD filter, affirming its ability to address both foreground and background noise in diseased brain tissues effectively.
  d The CNR, NI, ASNR, IV, NSD, and ENL metrics demonstrate outstanding values for the CTD-derived image, leading to improved contrast and radiologically preserved features in MR images of diseased brain tissues.

In conclusion, the CTD-filtered ischemic demyelination Brain MR image yields exceptional results, shedding further light on pathogenesis and enabling further evaluation of white matter lesions. This is evident through visual inspection and the estimated performance metrics.

### Clinical Examples 9, 10 and 11: Neoplastic lesions

To comprehensively evaluate the efficacy of CTD and denoising filters, our study expands to encompass cases of cerebral neoplasms. The brain exhibits various neoplastic lesions, including gliomas, glioblastoma, choroid plexus tumors, anaplastic astrocytoma, fibrillary astrocytoma, meningioma, cerebellar medulloblastoma, oligodendroglioma, craniopharyngioma, pituitary adenomas, brain metastases, and CNS lymphomas [141]. Accurate detection and differentiation of neoplastic lesions, crucial in neuroscience research, also include Neurofibromatosis type 1 [142] and multiple ring-enhancing lesions [143]. Dynamic contrast-enhanced MRI effectively distinguishes neoplastic lesions [144]. Gliomas, predominantly found in the central nervous system, often necessitate biopsy for verification, with altered metabolic activity and SSADH expression significantly influencing their growth [145, 146]. Note that gliomas are considered malignant [147].

4th ventricular tumors, strongly associated with spine metastases (SM), play a vital role in cerebral neoplasm identification [148]. Leptomeningeal disease (LMD) is common in cerebellar medulloblastomas, a specific neoplasm [149]. Rare cerebral neoplasms like Rosette-forming glioneuronal tumors (RGNT) occur in the 4th ventricle [150]. Treatment options for RGNT include gamma knife radiosurgery [151], azacytidine [152, 153], staged open cranial surgery [154], and tumor resection [155, 156], effectively managing and treating cerebral neoplasms.

Glioma lesions pose challenges for clinical diagnosis and imaging interpretation due to their neoplastic nature. This study highlights the use of MRI PROP mode for identifying neoplastic glioma lesions, chosen for its exceptional imaging capabilities crucial for tackling such lesions. Distinguishing between neoplasms and other conditions in MRI can be daunting, but PROP MRI, with glioma lesions manifesting as abnormal or high-intensity signals, aids in differentiation and contributes significantly to histopathological studies.

**Fig. 17:**
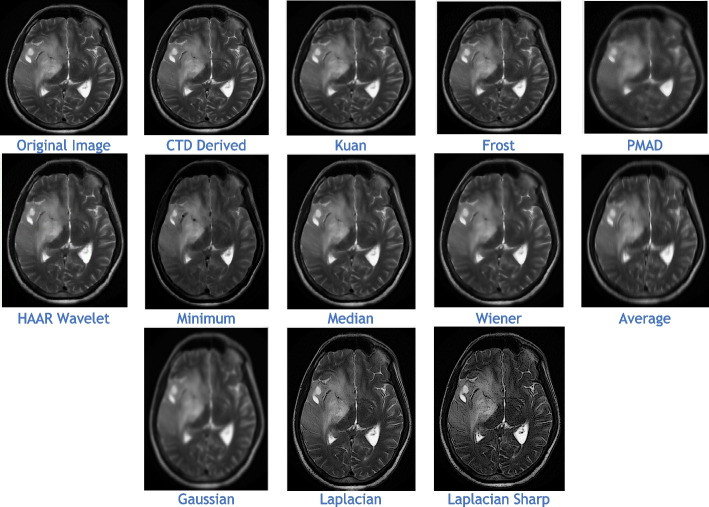
Denoised images of glioma neoplastic lesion as observed in the upper left corner

**Fig. 18:**
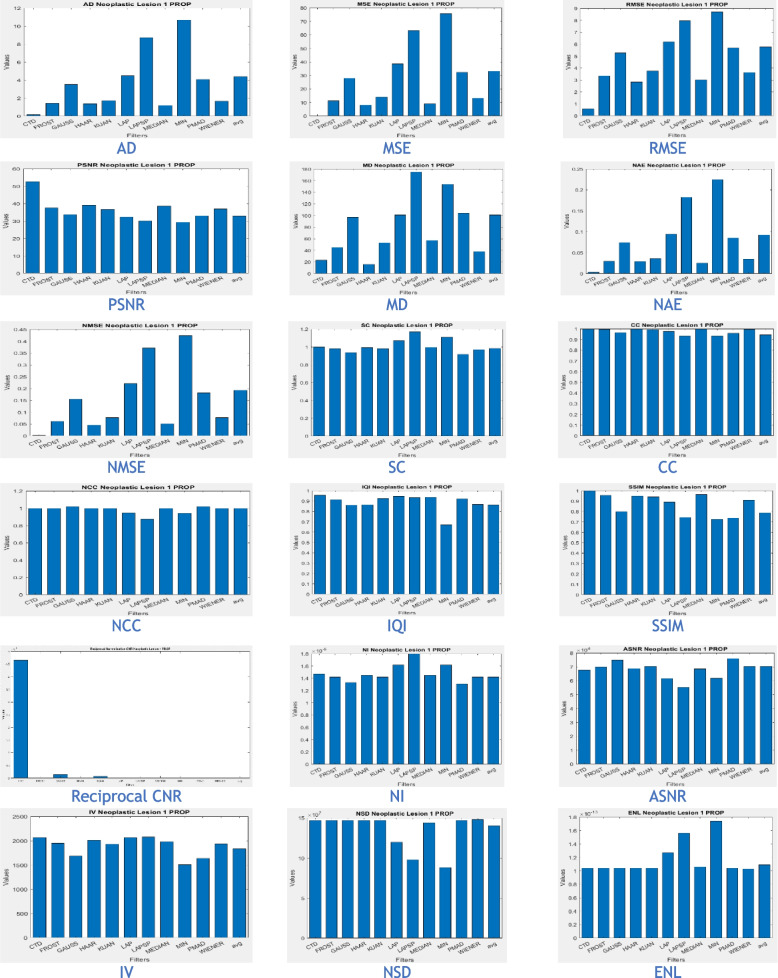
Histogram plots of the performance metrics of Neoplastic Lesion 1 glioma

The CTD-derived image (Fig. 17) proves highly beneficial in glioma analysis, offering high denoising value while genuinely preserving the original image signal and enhancing its quality. Quality and similarity metrics scores for the CTD image are impressive, as seen in metric values (Table 11) and corresponding histogram plots (Fig. 18). The nature and structural preservation in CTD images remain consistently striking for all glioma grades.

The combination of MRI PROP mode and CTD-derived images demonstrates a promising approach for accurate glioma diagnosis, supporting improved patient care and outcomes. CTD’s crystal-clear imaging and denoising advantages provide valuable support to clinicians and radiologists in detecting and characterizing glioma neoplastic lesions effectively.

Once again, we’ve selected an MR PROPELLER image, emphasizing PROP mode’s exceptional ability to display high-quality images crucial for neoplastic cases. The focus is on creating a clear MRI environment for comparing different neoplastic lesion cases, particularly challenging cases like 4th ventricular tumors. These intraventricular tumors, situated within the ventricular cavity, pose a difficulty in accurate detection.

**Fig. 19:**
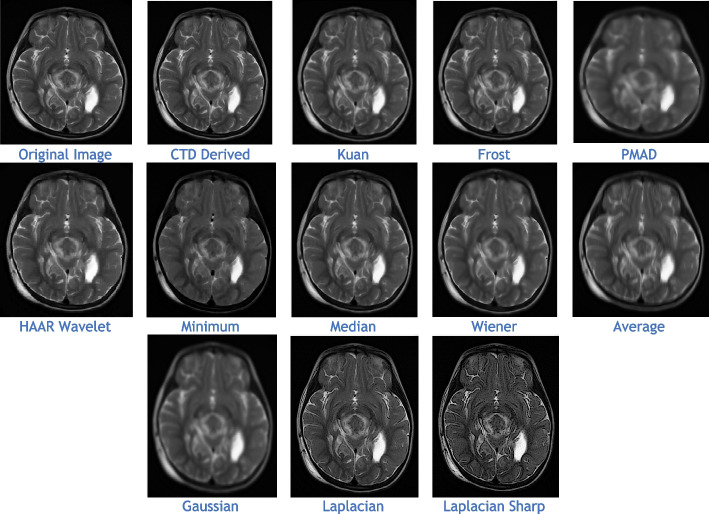
Denoised images of 4th ventricle tumour, a rare case of neoplastic lesion

This study presents two distinct neoplastic tumor cases in PROP mode (Figs. 17 and 19). Applying the CTD algorithm to these already clear images returns unparalleled results, showcasing its exceptional performance across various diseased cases. Despite a slight decrease in PSNR compared to glioma lesions, this is valuable as radiological signatures can be weak for 4th ventricular neoplastic tumors.

**Fig. 20:**
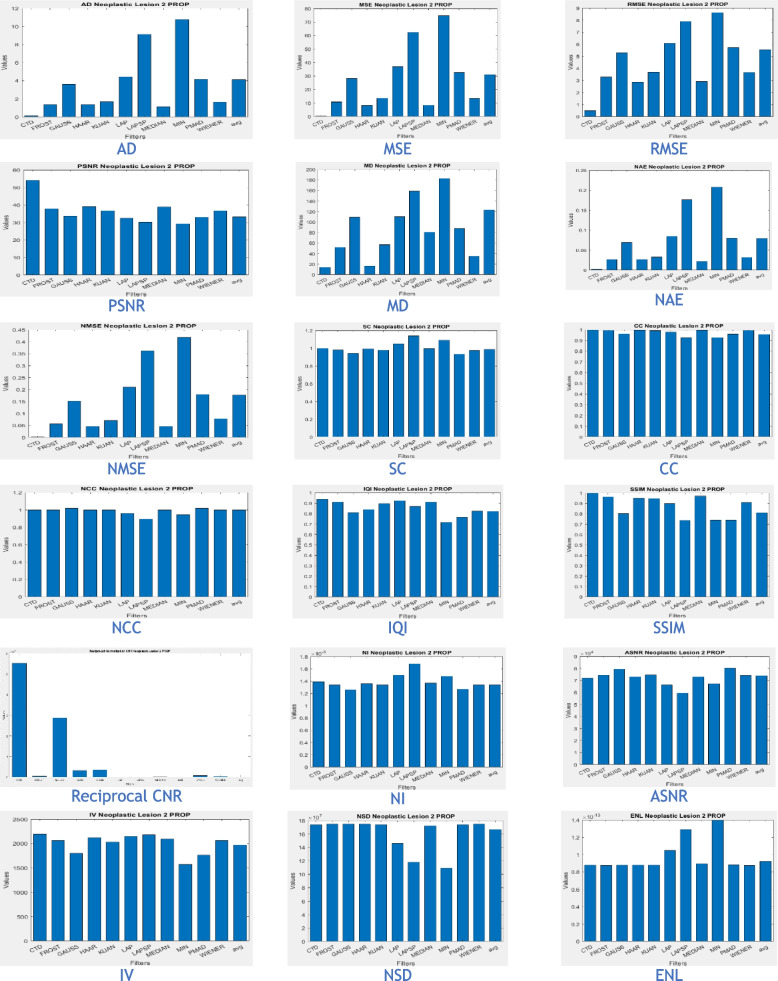
Histogram plots of the performance metrics of Neoplastic Lesion 2—4th ventricle tumour

Comparatively, quality and similarity metrics in CTD images closely align with glioma neoplastic lesions, evident in quality metric values (Table 12) and histogram plots (Fig. 20). Like in other cases, the CTD image preserves structural similarity, consistently producing enhanced radiological images.

In summary, the combination of MR PROPELLER and the CTD algorithm proves a powerful and reliable approach for neoplastic lesion imaging. Providing high-quality and detailed images, this combination aids radiologists in effectively detecting and analyzing neoplastic lesions, especially challenging 4th ventricular tumors within the ventricular cavity. The CTD algorithm’s exceptional performance solidifies its position as a valuable tool for enhancing radiological images across various diseased conditions.

In this study, FLAIR MRI is chosen for its ability to delineate gliomas, offering excellent multiplanar structural information and enhanced tissue characterization. A comparison between FLAIR and PROP mode reveals distinct strengths in detecting neoplastic lesions. FLAIR MRI excels in providing valuable structural information for gliomas, contributing to their diagnosis and prognosis.

**Fig. 21:**
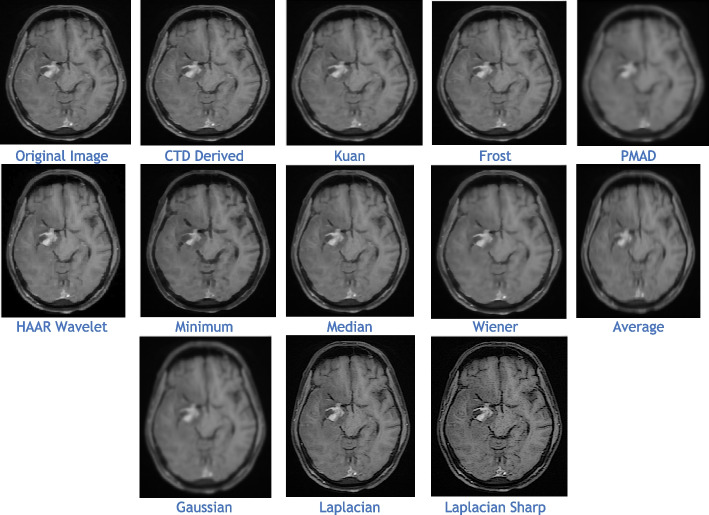
Denoised images of glioma lesion seen in middle left region of brain

Practical analysis highlights clear differences in CTD-derived images from PROP (Fig. 17) and FLAIR (Fig. 21) sequences for gliomas. Notably, PROP displays higher signal intensity features, while FLAIR exhibits diminished features. Despite variations, the CTD-derived FLAIR image demonstrates commendable denoising, ensuring a distortion-free image, supported by the PSNR value.

**Fig. 22:**
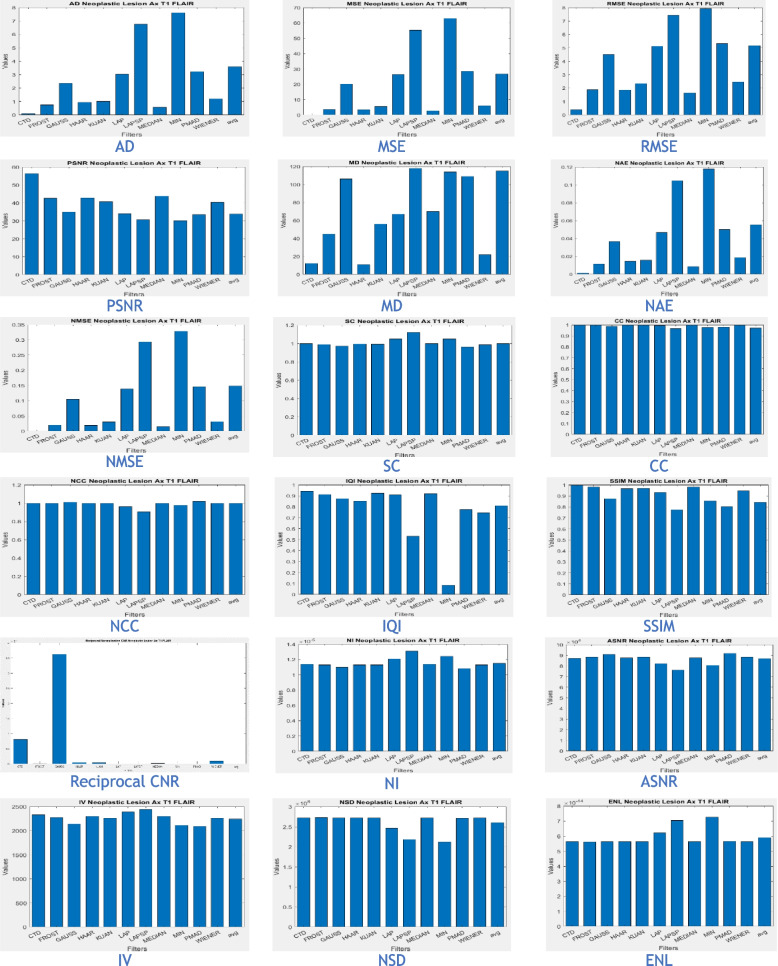
Histogram plots of the performance metrics of Neoplastic Lesion Ax T1 FLAIR glioma

Structural similarity (Table 13) and noise metrics histogram plots (Fig. 22), yield good values, though not as high as PROP, affirming radiologically well-preserved and error-free characteristics in the CTD FLAIR image. Both FLAIR and PROP MRI sequences prove valuable for glioma detection, with the CTD FLAIR image remaining a robust resource for accurate evaluation and analysis despite slight metric differences between the two sequences.

Based on the analysis of neoplastic lesion cases, the following conclusions were drawn:

- The CTD algorithm effectively removed Gaussian and Rayleigh noises, resulting in superior denoised images crucial for identifying cerebral edema in neoplastic brain lesions.
- Metrics such as SC, CC, NCC, and SSIM consistently showed a unity value for the CTD filter, maintaining striking similarity in neoplastic brain MR images. Even with greater denoising accuracy, the CTD image retained similarity, especially in cases involving blood vessel infiltration, compression, or vasospasm associated with cerebral neoplasms.
- The PSNR value for the CTD filter exceeded expectations, confirming its remarkable noise removal capability in neoplastic brain MR images.
- Metrics like CNR, NI, ASNR, IV, NSD, and ENL demonstrated commendable values for the CTD image, resulting in exceptional neoplastic brain MR images with enhanced contrast and well-preserved radiological features. This contributes to improved clinical diagnosis in various areas.
- Overall, the CTD filtered neoplastic brain MRI provided noise-free images with maintained structural similarity, as confirmed through visual inspection and estimated performance metrics.

## Results

The proposed CTD filter and other filters were assessed on various CT and MR images, and key inferences were drawn:

- Gaussian filter, while moderate in denoising, lacks efficient edge and contour preservation.
- Wiener denoising filter, despite suppressing frequency components, falls short in efficient edge and contour filtering.
- Laplacian filter, a derivative-based method, excels in preserving edge and contour details but results in loss of tissue-based information.
- Laplacian Sharp filter, another derivative-based method, preserves edge and contour details but lacks expected reliability in contrast details.
- Average filter, a denoising technique, yields poor results while preserving edges and contours.
- Minimum filter, a poor denoising filter, struggles to preserve edges and contours.
- Median filter, an average denoising filter, moderately preserves edges and contours.
- PMAD, a diffusion-based filter, performs moderately but shows poor denoising at edges and contours without loss in details.
- Kuan filter, a statistical-based technique, achieves modest performance in denoising and contrast enhancement.
- Frost filter, an exponential-based method, performs at a medium level in improving denoising and contrast.
- Haar, a wavelet-based filter, offers optimum performance in denoising, edge and contour preservation, and contrast enhancement.
- The proposed CTD filter is validated as the best choice for denoising, showcasing commendable performance in denoising, structural preservation of edges and contours, and contrast enhancement. Further, the visual inspection and estimated performance metrics affirm the denoising abilities of the proposed CTD filter substantiated by quality improvements across all CT and MR test images (clinical examples 1–11) in this research study.

## Discussion

Biomedical imaging modalities like CT and MRI are prone to noise and artifacts, which impacting image quality. Our research focused on the CTD technique, addressing quantum mottle noise in CT and Gaussian/Rayleigh noises in MR images. CTD excelled by achieving infinitesimal residual values with fewer iterations and shape functionality, outperforming traditional filters. Comparative studies, case analyses, visual inspections, and metrics affirmed CTD’s superiority, making it a valuable tool for future medical imaging assessments.

Furthermore, the CTD algorithm proved to be a robust computational technique for addressing critical visual aspects, such as similarity checks, contrast issues, and delineation of boundaries in both normal and pathological CT and MR images. Although the noise metrics scored similarly with respect to other filters, the CTD technique consistently achieved better values, thus demonstrating its overall superiority. Altogether CTD provided a clear picture of ridge shape, articulate globe lens with excellent clarity, distinct contrast of MCA territory, access the damage caused in MCA territory under various diseased conditions, improved resolution over hypo and hyper intense regions in all the cases of HRCT, CT and MR images including the diseased ones. The algorithm proved robust, addressing visual aspects and providing clarity in both normal and pathological images. CTD consistently surpassed other techniques in noise metrics, offering enhanced resolution in HRCT, CT, and MR images. Despite its longer execution time (5–7 min), CTD’s clinical validity and lack of major drawbacks reinforce its potential for high-quality image delivery.

## Conclusion

CT and MRI are standard imaging modalities but struggle with inherent noises, like quantum mottle in CT and Rayleigh, Rician, and Gaussian noises in MRI, impacting diagnostic clarity. Existing techniques fall short in fully addressing these issues. This research introduces the CTD framework, effectively mitigating noise problems in both CT and MRI. The outcomes of this research are profoundly encouraging and hold significant potential for acquiring precise diagnostic insights, especially in critical cases like Thoracic Cavity Carina, Head CT Globe Lens 4th Ventricle, Brain-Middle Cerebral Artery Territory, and neoplastic lesions. These discoveries establish the groundwork for integrating the proposed CTD technique into standard clinical diagnostic practices.

Comparative studies, clinical examples, visual inspections, and performance metrics confirm CTD’s superiority over traditional filters. CTD holds promise for Computer-Aided Diagnosis (CAD) post field trials. The results inspire confidence in accurate diagnostics for critical cases, setting the stage for routine clinical use. However, CTD’s complex nature hinders real-time implementation, prompting future exploration of hardware deployment in FPGA/Raspberry Pi/Arduino for thorough testing and validation in real-world scenarios.

### Supplementary Information

Supplementary Material 1.

Supplementary Material 2.

Supplementary Material 3.

Supplementary Material 4.

Supplementary Material 5.

Supplementary Material 6.

Supplementary Material 7.

Supplementary Material 8.

Supplementary Material 9.

Supplementary Material 10.

Supplementary Material 11.

Supplementary Material 12.

Supplementary Material 13.

Supplementary Material 14.

## Data Availability

The medical image data required for this research work on CT and MRI images was generously provided by Image Art, Vijaya Health Centre, located in Vadapalani, Chennai. We express our gratitude for their invaluable contribution. It is important to note that patient data will only be shared on an individual request basis, after ensuring compliance with the ethical conditions set by the data provider. The datasets utilized in this research article, along with the supplementary files, are included and made available for reference.

## Abbreviations

CT: Computer Tomography
HRCT: High Resolution Computed Tomography
MRI: Magnetic Resonance Imaging
T1, T2: Relaxation times
FLAIR: Fluid Attenuated Inversion Recovery
DWI: Diffusion Weighed Imaging
PROPELLER: Periodically Rotated Overlapping Parallel Lines with Enhanced Reconstruction
FBP: Filtered Back Projection
SAFIRE: Sinogram Affirmed Iterative Reconstruction
AD: Average Difference
MSE: Mean Square Error
RMSE: Root Mean Square Error
MD: Maximum Difference
NAE: Normalized Absolute Error
NMSE: Normalized Mean Square Error
PSNR: Peak Signal to Noise Ratio
SC: Structural Content
CC: Correlation Coefficient
NCC: Normalized Cross Correlation
IQI: Image Quality Index
SSIM: Structural Similarity Index Map
CNR: Contrast to Noise Ratio
NI: Noise Index
ASNR: Average Signal to Noise Ratio
IV: Image Variance
NSD: Noise Standard Deviation
ENL: Equivalent Number of Looks
TD: Topological Derivative
CTD: Continuum Topological Derivative
DFB: Discrete Filter Bank
CLT: Contourlet Transform
PDFB: Pyramidal Directional Filter Bank
EIT: Electrical Impedance Tomography
DICOM: Digital imaging and communication system
PACS: Picture Archiving and Communication System
RIS: Radiology Information Systems
DFOV: Display Field of View
SPI: Spiral mode
MCA: Middle Cerebral Artery
T-MCA: Twig like MCA
CAD: Computer Aided Diagnosis
SSADH: Succinic Semialdehyde Dehydrogenase
SM: Spine Metastases
LMD: Leptomeningeal Disease
RGNT: Rosette-forming glioneuronal Tumor
TIA: Transient Ischemic Attack
CADASIL: Cerebral Autosomal Dominant Arteriopathy with Subcortical Infarcts and Leukoencephalopathy
SVD: Small Vessel Disease
