# Supplementary material for: Continuum topological derivative - a novel application tool for denoising CT and MRI medical images
Source: BMC Med Imaging. 2024 Jul 24;24:182. doi: 10.1186/s12880-024-01341-1 (PMC11267933; doi:10.1186/s12880-024-01341-1)
Supplement: Supplementary file 1 — Supplementary Material 1. [file 12880_2024_1341_MOESM1_ESM.docx]

Clinical Example CS: Complex Cranial Base

| 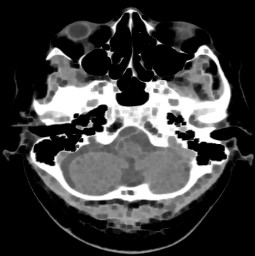 | 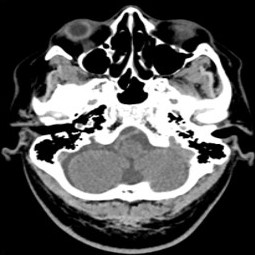 | 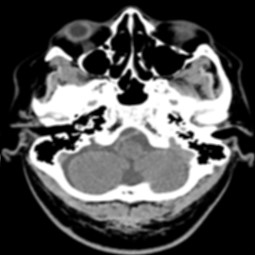 | 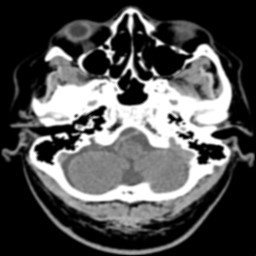 | 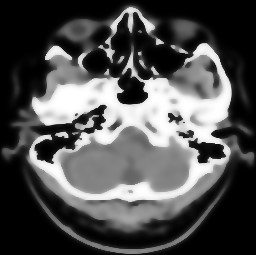 |
| --- | --- | --- | --- | --- |
| Original Image | CTD Derived | Kuan | Frost | PMAD |
| 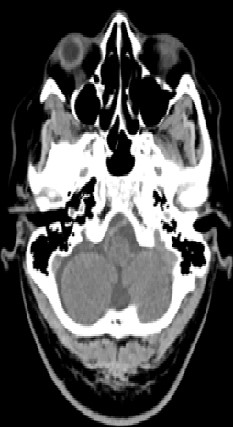 | 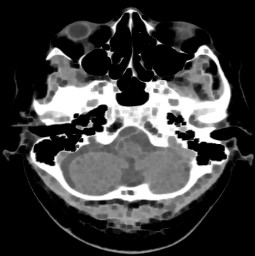 | 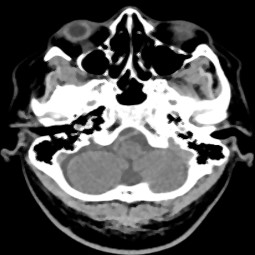 | 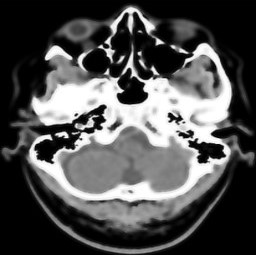 | 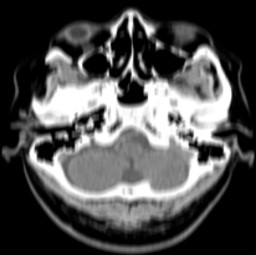 |
| HAAR Wavelet | Minimum | Median | Wiener | Average |
|  | 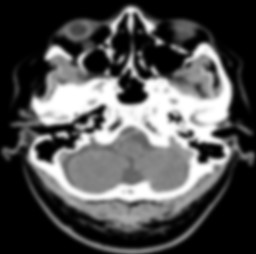 | 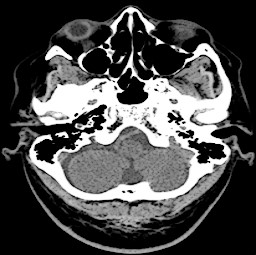 | 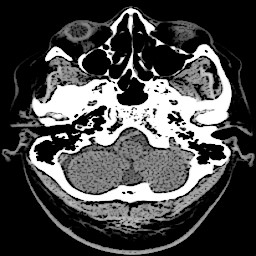 |  |
|  | Gaussian | Laplacian | Laplacian Sharp |  |
| **Figure CS1** Original and Denoised images of complex cranial base axial | | | | |

**Table CS1** Quality Metrics for complex cranial base axial

| Metrics | Continuum TD | Kuan Filter | Frost Filter | PMAD Filter(15 itrs) | Haar Wavelet | Ordinary Filter Min | Median Filter | Wiener Filter | Average Filter 7x7 | Gaussian Filter | Laplacian Filter | Laplacian Filter Sharp |
| --- | --- | --- | --- | --- | --- | --- | --- | --- | --- | --- | --- | --- |
| AD | 0.0051 | 2.75 | 2.24 | 5.37 | 0.5119 | 18.97 | 1.11 | 2.95 | 8.35 | 7.11 | 4.69 | 8.61 |
| MSE | 0.0172 | 24.67 | 20.17 | 48.86 | 1.26 | 89.17 | 8.57 | 30.76 | 47.39 | 48.17 | 33.21 | 42.05 |
| RMSE | 0.1311 | 4.96 | 4.49 | 6.99 | 1.12 | 9.44 | 2.92 | 5.54 | 6.88 | 6.94 | 5.76 | 6.48 |
| PSNR | 65.77 | 34.20 | 35.08 | 31.24 | 47.09 | 28.62 | 38.79 | 33.25 | 31.37 | 31.30 | 32.91 | 31.89 |
| MD | 19 | 107 | 92 | 119 | 5 | 234 | 136 | 105 | 173 | 175 | 170 | 209 |
| NAE | 6.31e-05 | 0.0344 | 0.0281 | 0.0672 | 0.0064 | 0.2374 | 0.0138 | 0.0370 | 0.1046 | 0.0889 | 0.0587 | 0.1077 |
| NMSE | 1.40e-04 | 0.1791 | 0.1472 | 0.3550 | 0.0091 | 0.6505 | 0.0606 | 0.2208 | 0.3496 | 0.3502 | 0.2464 | 0.3152 |
| SC | 1 | 0.9371 | 0.94 | 0.83 | 1 | 1.19 | 1 | 0.89 | 0.90 | 0.84 | 1.09 | 1.17 |
| CC | 1 | 0.99 | 0.99 | 0.98 | 1 | 0.92 | 1 | 0.99 | 0.95 | 0.96 | 0.98 | 0.96 |
| NCC | 1 | 1.01 | 1.01 | 1.02 | 1 | 0.88 | 0.99 | 1.01 | 1.01 | 1.02 | 0.92 | 0.86 |
| IQI | 1 | 0.96 | 0.96 | 0.90 | 0.96 | 0.75 | 1 | 0.90 | 0.91 | 0.8770 | 0.95 | 0.94 |
| SSIM | 1 | 0.94 | 0.96 | 0.75 | 0.99 | 0.66 | 0.97 | 0.88 | 0.73 | 0.7318 | 0.93 | 0.85 |
| CNR | 6.86e-07 | 2.94e-05 | 4.41e-04 | 3.12e-04 | 6.86e-05 | 0.1499 | 0.0013 | 8.57e-04 | 0.0142 | 1.86e-04 | 0.0352 | 0.0645 |
| NI | 1.79e-05 | 1.73e-05 | 1.75e-05 | 1.69e-05 | 1.79e-05 | 2.11e-05 | 1.79e-05 | 1.73e-05 | 1.71e-05 | 1.63e-05 | 1.91e-05 | 2.02e-05 |
| ASNR | 5.55e+04 | 5.74e+04 | 5.71e+04 | 5.89e+04 | 5.57e+04 | 4.73e+04 | 5.55e+04 | 5.75e+04 | 5.82e+04 | 6.10e+04 | 5.22e+04 | 4.94e+04 |
| IV | 8.89e+03 | 8.30e+03 | 8.42e+03 | 7.89e+03 | 8.83e+03 | 7.13e+03 | 8.84e+03 | 8.29e+03 | 7.71e+03 | 7.34e+03 | 8.91e+03 | 8.94e+03 |
| NSD | 4.18e+08 | 4.18e+08 | 4.19e+08 | 4.18e+08 | 4.18e+08 | 2.43e+08 | 4.17e+08 | 4.20e+08 | 3.99e+08 | 4.18e+08 | 3.71e+08 | 3.33e+08 |
| ENL | 3.64e-14 | 3.64e-14 | 3.63e-14 | 3.64e-14 | 3.64e-14 | 6.26e-14 | 3.65e-14 | 3.63e-14 | 3.81e-14 | 3.64e-14 | 4.11e-14 | 4.57e-14 |

| 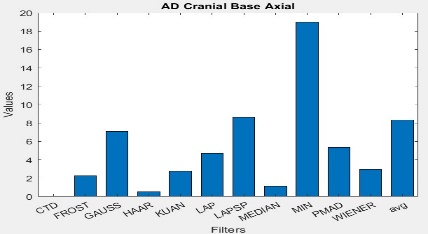  AD | 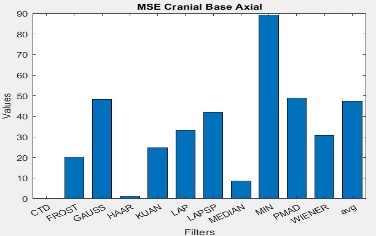  MSE | 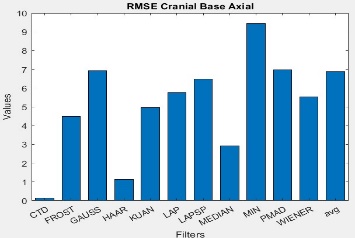  RMSE |
| --- | --- | --- |
| 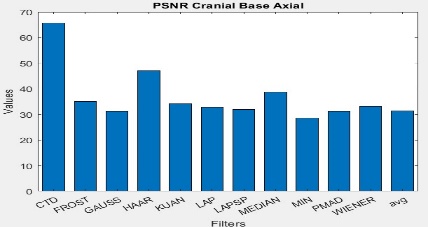  PSNR | 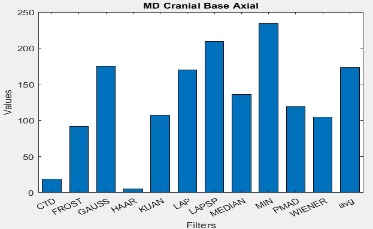  MD | 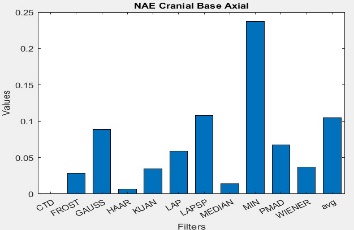  NAE |
| 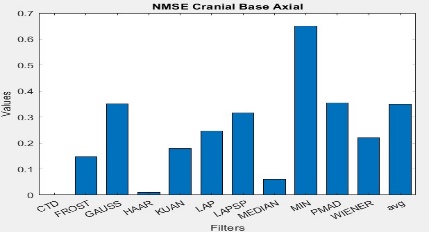  NMSE | 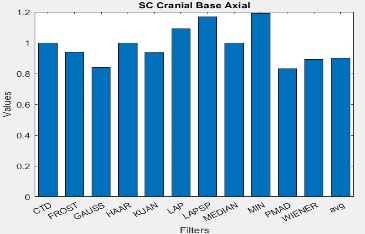  SC | 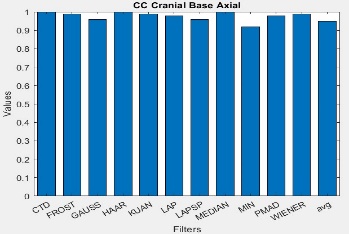  CC |
| 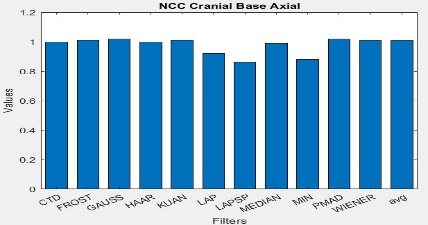  NCC | 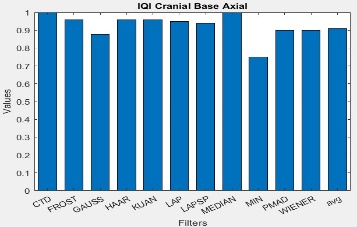  IQI | 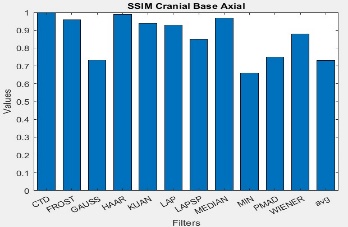  SSIM |
| 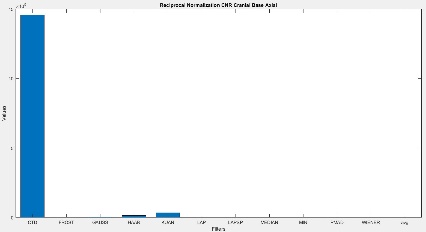  Reciprocal CNR | 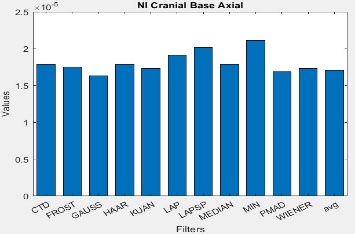  NI | 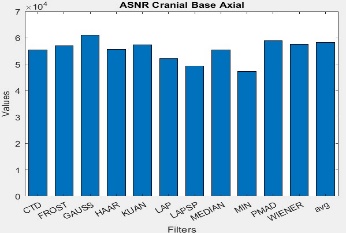  ASNR |
| 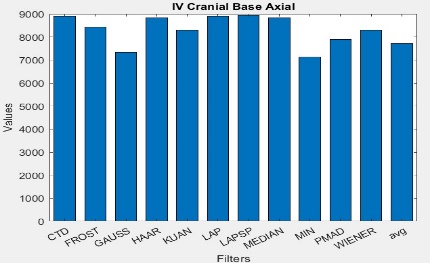  IV | 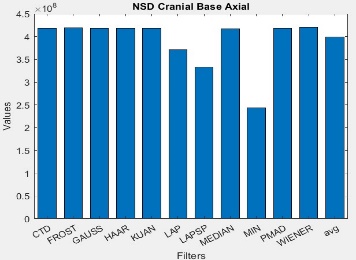  NSD | 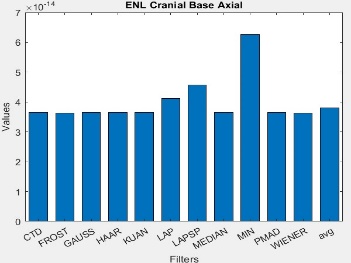  ENL |

**Figure CS2** Histogram plots of the performance metrics of Extensive Intracranial Hemorrhage

Cranial Skull Base Axial:

In analyzing the filtered images, metrics, and histogram plots for infarct and the axial CT head image of the cranial skull base—a preferred sequence for optimal viewing—we draw the following conclusions:

1. AD, MSE, RMSE, MD, NAE, and NMSE exhibited extremely low values compared to other filters, achieving exceptional denoising of quantum mottle throughout the cranial skull base. Consequently, the CTD denoised image perfectly reconstructed complex structures such as the zygomatic arch, sphenoid bone, temporal bone, occipital bone, nasal and vomer bone, foramen magnum, and suboccipital muscles.
2. SC, CC, NCC, IQI, and SSIM consistently resulted in a unity value for the CTD filter, showcasing perfect preservation of similarity in nasal cavity, temporal process of the zygomatic bone, zygomatic process of the temporal bone, nasopharynx, mastoid process, cerebellum, cavities, bones, and muscles within this cranial floor. In contrast, unity value deviations were observed for other filters, highlighting the supremacy of CTD in structural resemblance.
3. PSNR for the CTD filter returned an excellent value, effectively eliminating noise in all regions of interest within the cranial skull base.
4. CNR, NI, ASNR, IV, NSD, and ENL metrics yielded appreciable values for the CTD image, resulting in a noise-free representation with well-preserved radiological features in muscles, tissues, orifices, processes, and bony regions of this complex cranial base.
5. Overall, the CTD-filtered cranial skull base image produced a highly standardized radiological image, showcasing its effectiveness in another unique case.
