# Supplementary material for: Continuum topological derivative - a novel application tool for denoising CT and MRI medical images
Source: BMC Med Imaging. 2024 Jul 24;24:182. doi: 10.1186/s12880-024-01341-1 (PMC11267933; doi:10.1186/s12880-024-01341-1)
Supplement: Supplementary file 2 — Supplementary Material 2. [file 12880_2024_1341_MOESM2_ESM.docx]

# **Algorithm for Restoration using CTD**

- Obtain the Gray-scale CT or MR image
- Resize the image into $256\times256$resolution
- Apply Isotropic Diffusion method for measuring the conductivity of diffusion in pixels
- Fix the initial conductivity value $K_{I_{m}}^{a}$
- $K_{I_{m}}^{a}$ is the perturbed value in the topological derivative problem
- Only $\left|K_{I_{m}}^{a}\right|$ will be used for solving the problem
- Fix the penetration of conduction over perturbed location $K_{p}^{c}$
- Value of $K_{p}^{c}$ will be infinitesimal compared to $K_{I_{m}}^{a}$
- Choose the percentage of pixels for applying diffusion $pc\left(K_{I_{m}}^{a}\right)$
- Reshape the original image as a single column $I_{m}^{T}$
- Create a diffusion tensor of $1 and 0$
- Represent this diffusion tensor by$K_{d}^{I_{m}}\left(\left|K_{I_{m}}^{a}\right|\right)$
- Create a Symbolic matrix $S_{i*j}^{id}$
- Introduce diffusivity $K_{d}^{I_{m}}\left(\left|K_{I_{m}}^{a}\right|\right)$ into the problem
- Obtain two global matrices $k=D_{G}^{S_{i*j}^{id}}\left(K_{d}^{I_{m}}\left(\left|K_{I_{m}}^{a}\right|\right)\right)$ and $l=E_{G}\left(S_{i*j}^{id}\right)$

$D_{G}^{S_{i*j}^{id}}$ and $E_{G}$ are diffusion coefficients

- Both $k$ and $l$ are sparse matrices
- Calculate $k_{p}^{S_{i*j}^{id}}=k\times I_{m}^{T}$ and $l_{p}^{S_{i*j}^{id}}=l\times I_{m}^{T}$
- Find $kt=k+l$
- Fix the tolerance value and number of iterations
- Equate $ktX_{a}=X_{a}l_{p}^{S_{i*j}^{id}}$
- Solve for $X_{a}$ using conjugate gradient method
- Solve for $ktX_{b}=X_{b}k_{p}^{S_{i*j}^{id}}$
- Solve for $X_{b}$ using conjugate gradient method
- Compute $X_{A}=X_{a}^{i,j}\left(S_{i*j}^{id}\right)$ and $X_{B}=X_{b}^{i,j}\left(S_{i*j}^{id}\right)$
- Create a tensor $X_{TD}^{i,j}=tsr\left([X_{A,}X_{A}^{T},X_{B,}X_{B}^{T}]\right)$//Here *tsr* denotes a tensor//
- $X_{TD}^{i,j}$ is basically a gradient norm function
- Compute the eigenvalues of $X_{TD}^{i,j}$
- Denote it by$\lambda_{TD}^{i,j}$
- Functionally it can be written as $\lambda_{TD}^{i,j}\left(X_{TD}^{i,j}\right)$
- Topological derivative $TD$ is arrived by computing $\lambda_{TD}^{i,j}\left(X_{TD}^{i,j}\right)$
- Apply $TD$ to all pixels in the image
- Compute new diffusivity tensor $K_{D}^{TD}$ after applying $TD$
- Functionally it can be written as $K_{D}^{TD}\left(K_{d}^{I_{m}}\left(\left|K_{I_{m}}^{a}\right|\right)\right)$
- Obtain another global matrix $k_{D}^{TD}=K_{D}^{TD}\left(K_{d}^{I_{m}}\left(\left|K_{I_{m}}^{a}\right|\right)\right)$
- Calculate $k_{1}t=k_{D}^{TD}+l$
- Fix different tolerance and iteration values
- Equate $k_{1}tX_{c}=X_{c}l_{p}^{S_{i*j}^{id}}$
- Solve for $X_{c}$ using conjugate gradient method
- Reshape the noised image with $X_{c}$
- It gives the denoised image obtained with topological derivative method
