# Supplementary material for: Continuum topological derivative - a novel application tool for denoising CT and MRI medical images
Source: BMC Med Imaging. 2024 Jul 24;24:182. doi: 10.1186/s12880-024-01341-1 (PMC11267933; doi:10.1186/s12880-024-01341-1)
Supplement: Supplementary file 3 — Supplementary Material 3. [file 12880_2024_1341_MOESM3_ESM.docx]

Clinical Example AS: Extensive Intracranial Haemorrhage

| 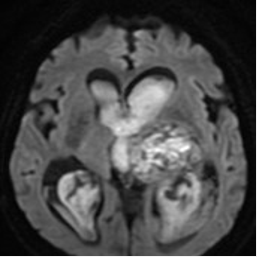 | 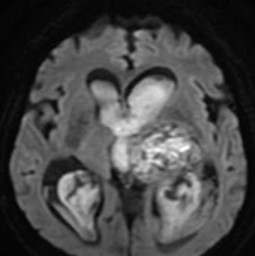 | 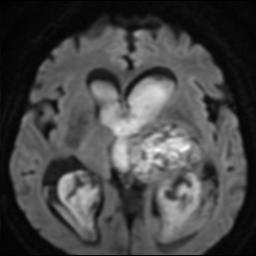 | 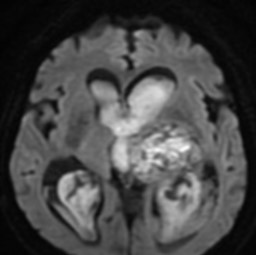 | 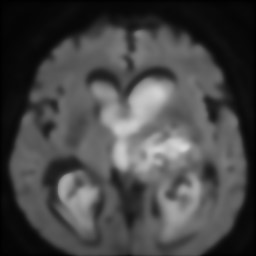 |
| --- | --- | --- | --- | --- |
| Original Image | CTD Derived | Kuan | Frost | PMAD |
| 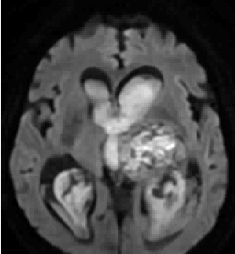 | 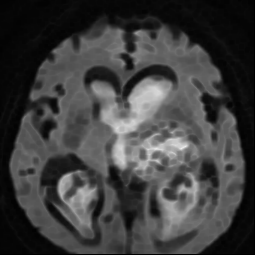 | 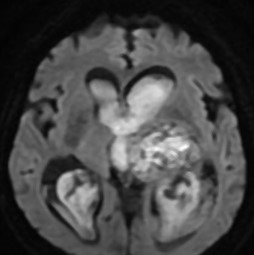 | 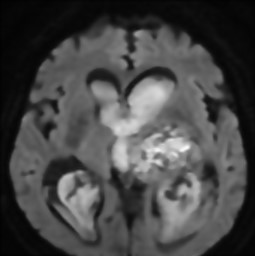 | 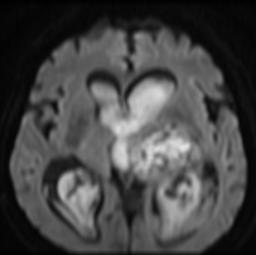 |
| HAAR Wavelet | Minimum | Median | Wiener | Average |
|  | 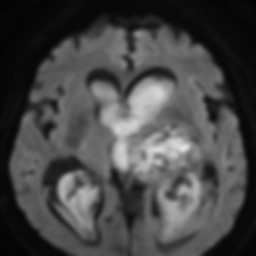 | 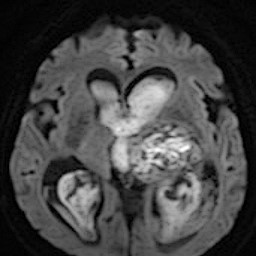 | 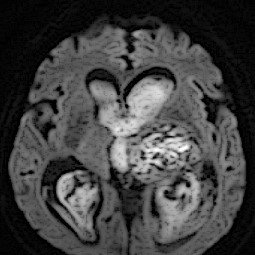 |  |
|  | Gaussian | Laplacian | Laplacian Sharp |  |
| **Figure AS1** Original and Denoised images of extensive intracranial haemorrhage | | | | |

**Table AS1** Quality Metrics for extensive intracranial haemorrhage

| Metrics | Continuum TD | Kuan Filter | Frost Filter | PMAD Filter(15 itrs) | Haar Wavelet | Ordinary Filter Min | Median Filter | Wiener Filter | Average Filter 7x7 | Gaussian Filter | Laplacian Filter | Laplacian Filter Sharp |
| --- | --- | --- | --- | --- | --- | --- | --- | --- | --- | --- | --- | --- |
| AD | 0.0927 | 0.8763 | 0.4787 | 3.54 | 1.04 | 8.41 | 0.3578 | 1.24 | 3.60 | 2.26 | 2.18 | 5.55 |
| MSE | 0.1090 | 2.95 | 1.21 | 33.55 | 3.84 | 76.70 | 1.11 | 5.90 | 29.19 | 18.99 | 17.37 | 49.80 |
| RMSE | 0.3301 | 1.72 | 1.10 | 5.79 | 1.96 | 8.75 | 1.05 | 2.43 | 5.40 | 4.35 | 4.16 | 7.05 |
| PSNR | 57.76 | 43.42 | 47.30 | 32.87 | 42.28 | 29.28 | 47.65 | 40.41 | 33.48 | 35.34 | 35.73 | 31.15 |
| MD | 6 | 101 | 13 | 55 | 11 | 103 | 19 | 22 | 88 | 44 | 47 | 114 |
| NAE | 0.0013 | 0.0124 | 0.0068 | 0.0502 | 0.0147 | 0.1191 | 0.0051 | 0.0176 | 0.0511 | 0.032 | 0.0310 | 0.0786 |
| NMSE | 4.82e-04 | 0.0147 | 0.0060 | 0.1622 | 0.0186 | 0.3760 | 0.0055 | 0.0284 | 0.1469 | 0.0925 | 0.0851 | 0.2429 |
| SC | 1 | 0.99 | 0.99 | 0.98 | 1 | 1.06 | 1 | 0.99 | 1.01 | 0.98 | 1.03 | 1.08 |
| CC | 1 | 0.99 | 1 | 0.98 | 1 | 0.9838 | 1 | 0.99 | 0.98 | 0.99 | 0.99 | 0.98 |
| NCC | 1 | 0.99 | 1 | 1 | 1 | 0.9651 | 1 | 1 | 0.99 | 1 | 0.97 | 0.93 |
| IQI | 1 | 0.9770 | 0.9881 | 1.04 | 0.9470 | 0.8948 | 0.99 | 0.9706 | 0.90 | 0.92 | 0.98 | 0.92 |
| SSIM | 1 | 0.9758 | 0.99 | 0.8107 | 0.9653 | 0.8850 | 0.99 | 0.9552 | 0.87 | 0.90 | 0.96 | 0.84 |
| CNR | 1.04e-06 | 0.0025 | 0.0017 | 0.0044 | 1.83e-04 | 0.1178 | 2.03e-05 | 0.0012 | 0.02 | 1.73e-05 | 0.03 | 0.0748 |
| NI | 1.12e-05 | 1.11e-05 | 1.11e-05 | 1.06e-05 | 1.11e-05 | 1.20e-05 | 1.11e-05 | 1.10e-05 | 1.13e-05 | 1.07e-05 | 1.16e-05 | 1.24e-05 |
| ASNR | 8.90e+04 | 8.95e+04 | 8.99e+04 | 9.36e+04 | 8.95e+04 | 8.31e+04 | 8.93e+04 | 9.03e+04 | 8.82e+04 | 9.26e+04 | 8.56e+04 | 8.05e+04 |
| IV | 2.70e+03 | 2.66e+03 | 2.65e+03 | 2.42e+03 | 2.67e+03 | 2.40e+03 | 2.67e+03 | 2.61e+03 | 2.62e+03 | 2.49e+03 | 2.73e+03 | 2.80e+03 |
| NSD | 3.27e+08 | 3.25e+08 | 3.27e+08 | 3.23e+08 | 3.26e+08 | 2.53e+08 | 3.26e+08 | 3.25e+08 | 3.11e+08 | 3.27e+08 | 3.06e+08 | 2.77e+08 |
| ENL | 4.67e-14 | 4.69e-14 | 4.65e-14 | 4.71e-14 | 4.70e-14 | 6.01e-14 | 4.67e-14 | 4.68e-14 | 4.89e-14 | 4.67e-14 | 4.97e-14 | 5.50e-14 |

| 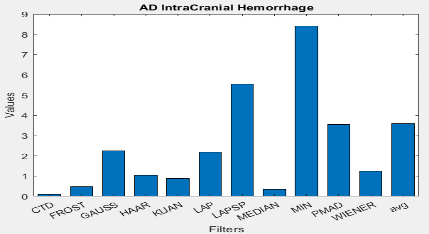  AD | 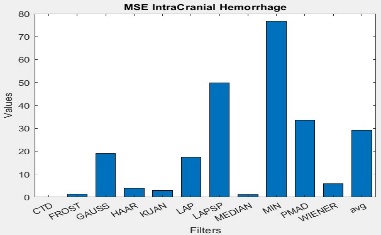  MSE | 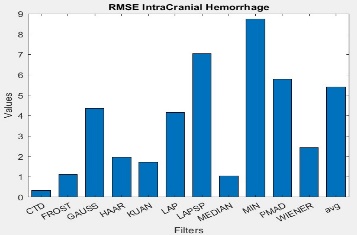  RMSE |
| --- | --- | --- |
| 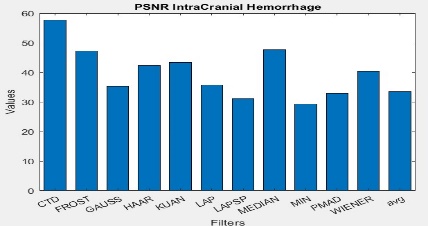  PSNR | 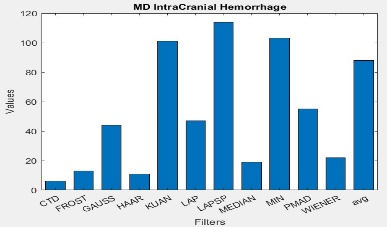  MD | 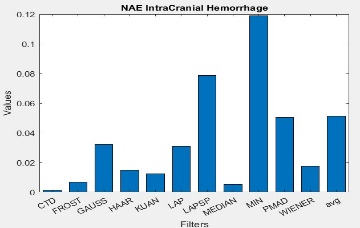  NAE |
| 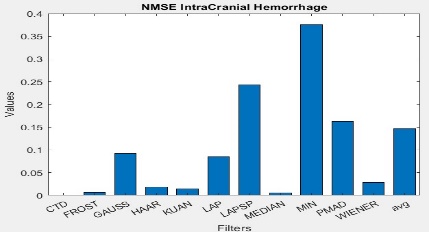  NMSE | 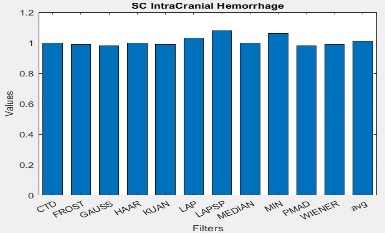  SC | 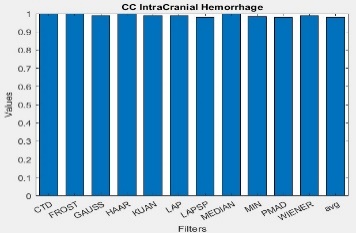  CC |
| 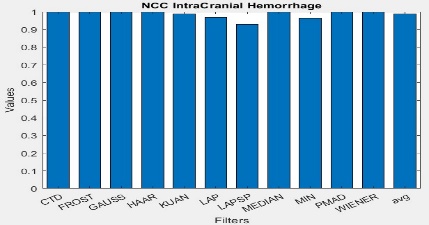  NCC | 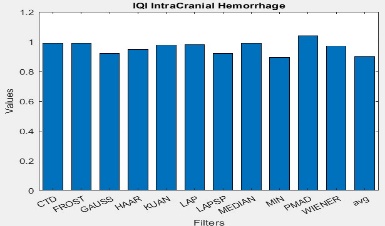  IQI | 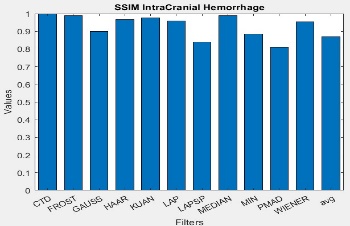  SSIM |
| 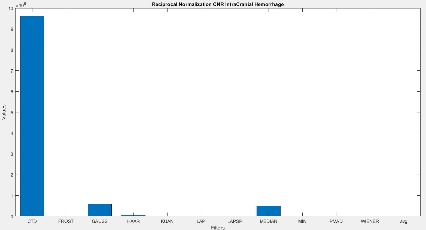  Reciprocal CNR | 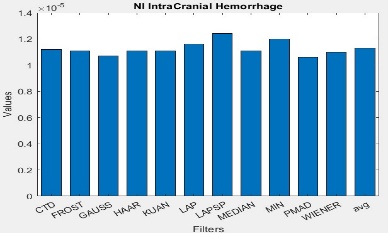  NI | 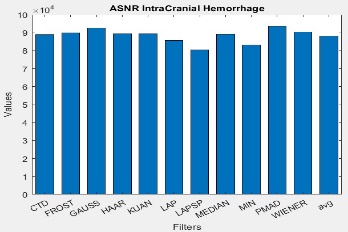  ASNR |
| 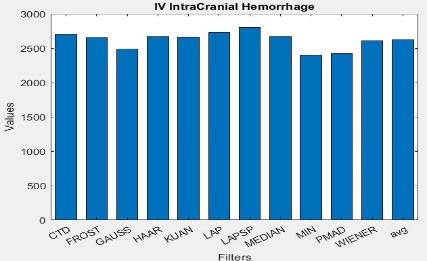  IV | 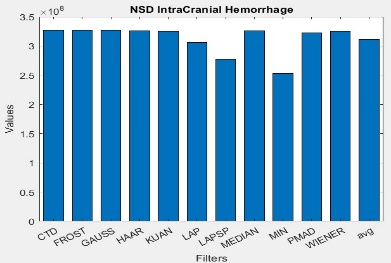  NSD | 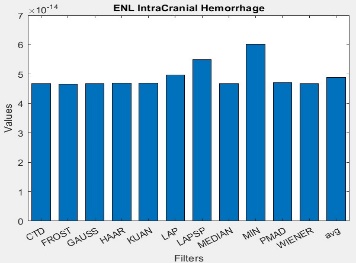  ENL |

**Figure AS2** Histogram plots of the performance metrics of Extensive Intracranial Hemorrhage

Extensive Intracranial Hemorrhage:

In the analysis of filtered images, metrics, and histogram plots for infarct and extensive intracranial hemorrhage, the focus is on presenting FLAIR T2 MRI of an extensive intracranial hemorrhage case with secondary intraventricular and intracerebral bleeding.

1. AD, MSE, RMSE, MD, NAE, and NMSE yielded the lowest values among all filters, signifying exceptional denoising of Gaussian and Rayleigh noises in extensive intracranial hemorrhage. Consequently, the CTD denoised image provided additional insights into hyperintense areas, enhancing the visualization of cerebral bleed areas.
2. SC, CC, NCC, IQI, and SSIM consistently returned a unity value for the CTD filter. In contrast, other filters either showed unity for some metrics or values lower/higher than unity. Thus, the CTD-filtered image maintained a high degree of structural similarity, especially in cerebral bleed areas.
3. PSNR for the CTD filter achieved a commendable value, effectively removing both foreground and background noise from cerebral bleed, brain tissue, and ventricles.
4. CNR, NI, ASNR, IV, NSD, and ENL metrics demonstrated good values for the CTD image, resulting in a noise-free representation with improved contrast and diagnostically appreciable radiological features in extensive intracranial hemorrhage.
5. Ultimately, the CTD-filtered hemorrhage image exhibited high quality with no structural deformation, preserving radiological characteristics even in cerebral bleed areas for this intracranial hemorrhage case.
