# Supplementary material for: Continuum topological derivative - a novel application tool for denoising CT and MRI medical images
Source: BMC Med Imaging. 2024 Jul 24;24:182. doi: 10.1186/s12880-024-01341-1 (PMC11267933; doi:10.1186/s12880-024-01341-1)
Supplement: Supplementary file 4 — Supplementary Material 4. [file 12880_2024_1341_MOESM4_ESM.docx]

| 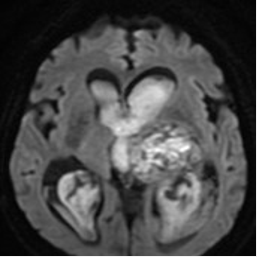 | 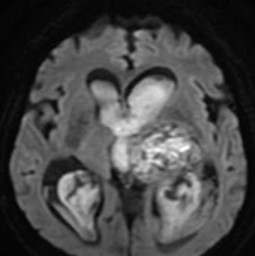 | 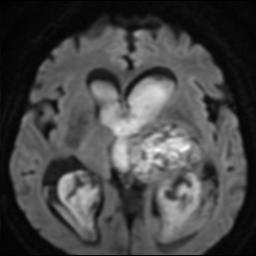 | 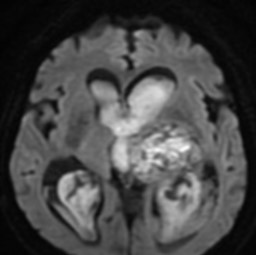 | 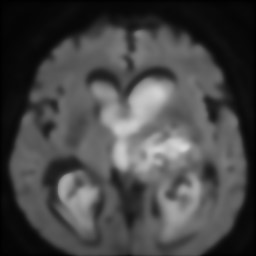 |
| --- | --- | --- | --- | --- |
| Original Image | CTD Derived | Kuan | Frost | PMAD |
| 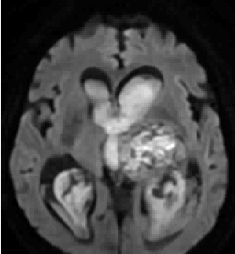 | 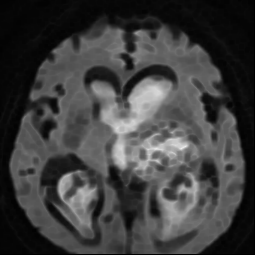 | 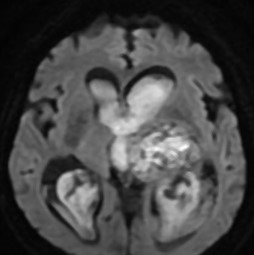 | 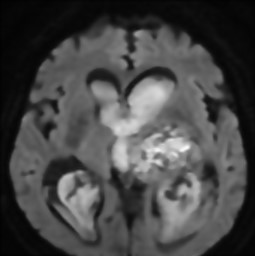 | 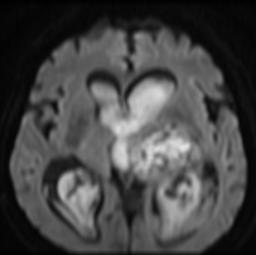 |
| HAAR Wavelet | Minimum | Median | Wiener | Average |
|  | 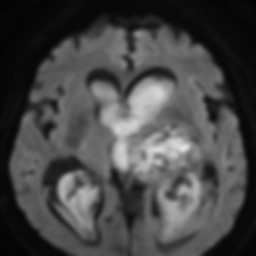 | 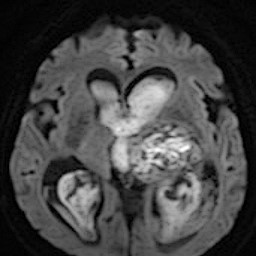 | 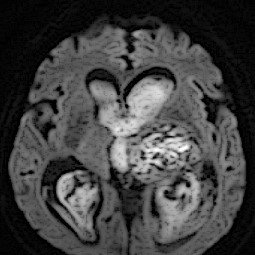 |  |
|  | Gaussian | Laplacian | Laplacian Sharp |  |
| **Figure AS1** Denoised images of extensive intracranial hemorrhage | | | | |
