# Supplementary material for: Continuum topological derivative - a novel application tool for denoising CT and MRI medical images
Source: BMC Med Imaging. 2024 Jul 24;24:182. doi: 10.1186/s12880-024-01341-1 (PMC11267933; doi:10.1186/s12880-024-01341-1)
Supplement: Supplementary file 9 — Supplementary Material 9. [file 12880_2024_1341_MOESM9_ESM.docx]

| 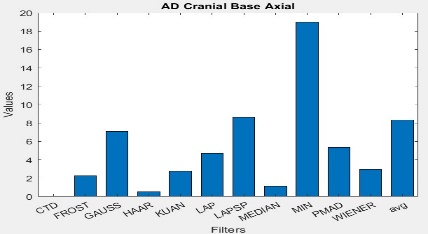  AD | 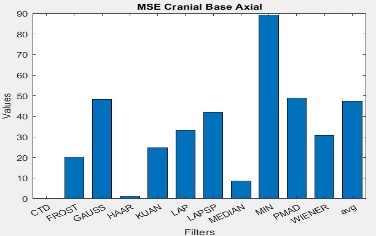  MSE | 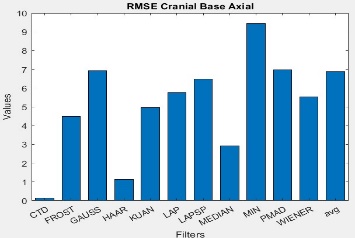  RMSE |
| --- | --- | --- |
| 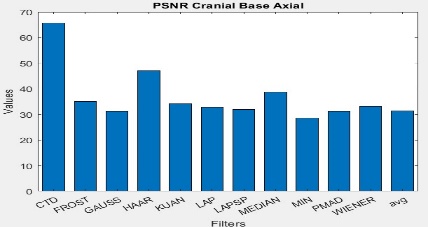  PSNR | 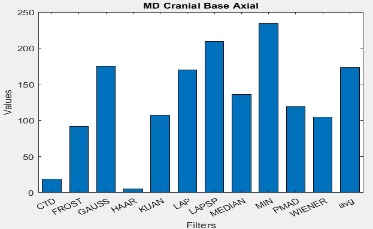  MD | 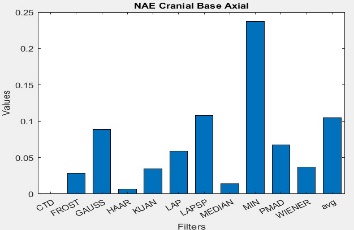  NAE |
| 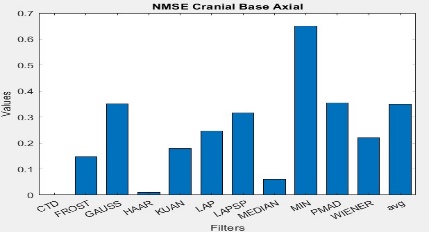  NMSE | 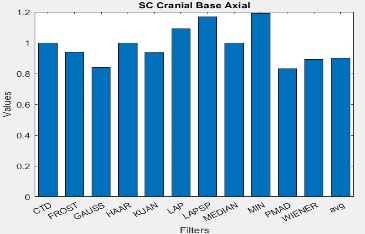  SC | 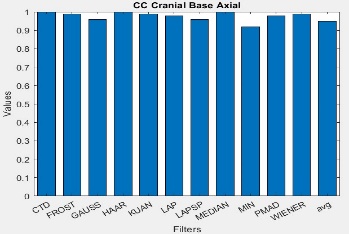  CC |
| 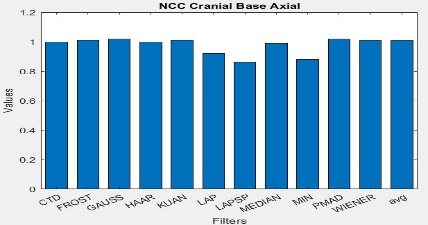  NCC | 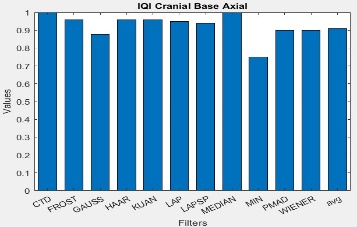  IQI | 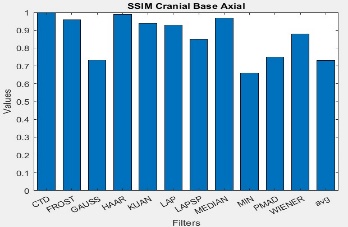  SSIM |
| 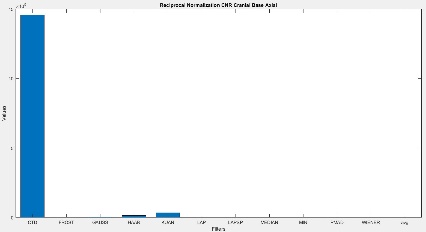  Reciprocal CNR | 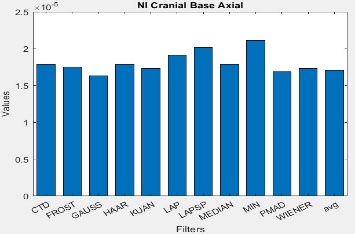  NI | 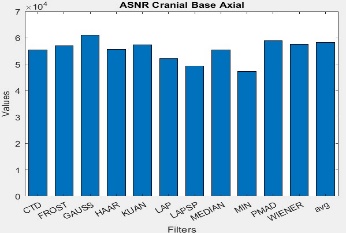  ASNR |
| 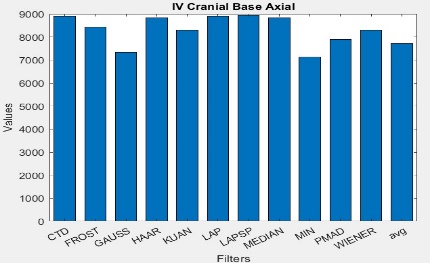  IV | 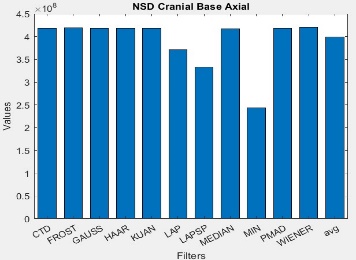  NSD | 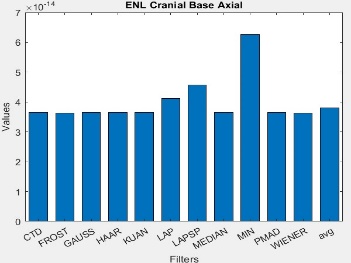  ENL |

**Figure CS2** Histogram plots of the performance metrics of Extensive Intracranial Hemorrhage
