# Supplementary material for: Continuum topological derivative - a novel application tool for denoising CT and MRI medical images
Source: BMC Med Imaging. 2024 Jul 24;24:182. doi: 10.1186/s12880-024-01341-1 (PMC11267933; doi:10.1186/s12880-024-01341-1)
Supplement: Supplementary file 10 — Supplementary Material 10. [file 12880_2024_1341_MOESM10_ESM.docx]

Clinical Example BS: Fractured Knee

| 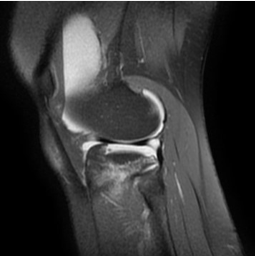 | 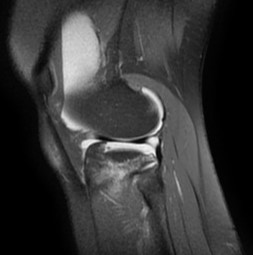 | 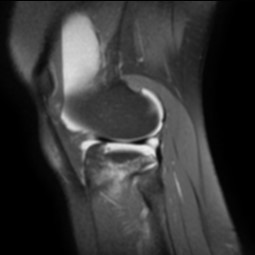 | 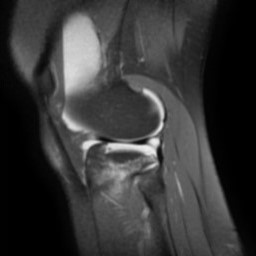 | 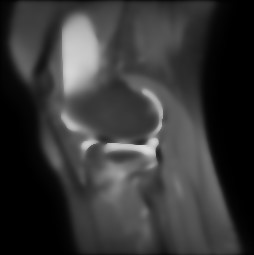 |
| --- | --- | --- | --- | --- |
| Original Image | CTD Derived | Kuan | Frost | PMAD |
| 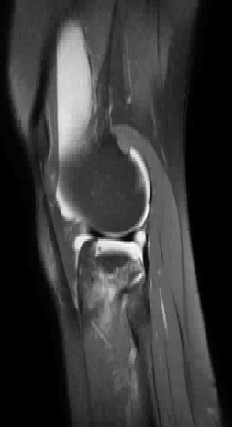 | 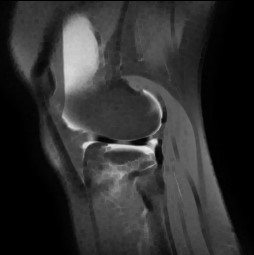 | 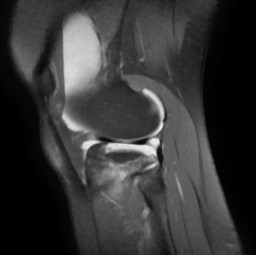 | 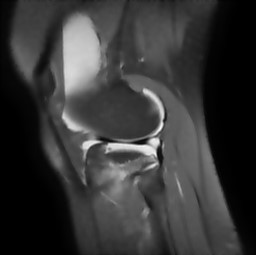 | 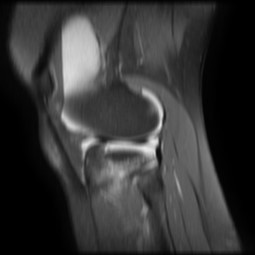 |
| HAAR Wavelet | Minimum | Median | Wiener | Average |
|  | 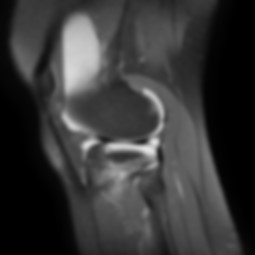 | 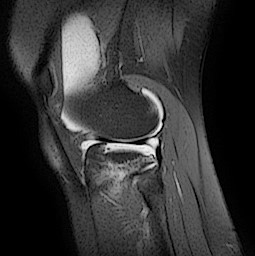 | 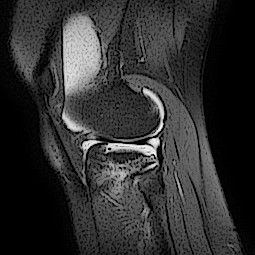 |  |
|  | Gaussian | Laplacian | Laplacian Sharp |  |
| **Figure BS1** Original and Denoised images of fractured left knee | | | | |

**Table BS1** Quality Metrics for fractured left knee

| Metrics | Continuum TD | Kuan Filter | Frost Filter | PMAD Filter(15 itrs) | Haar Wavelet | Ordinary Filter Min | Median Filter | Wiener Filter | Average Filter 7x7 | Gaussian Filter | Laplacian Filter | Laplacian Filter Sharp |
| --- | --- | --- | --- | --- | --- | --- | --- | --- | --- | --- | --- | --- |
| AD | 0.063 | 1.19 | 0.7204 | 2.67 | 0.8762 | 6.90 | 0.6247 | 1.17 | 2.73 | 1.98 | 2.99 | 7.07 |
| MSE | 0.1000 | 6.70 | 3.55 | 23.05 | 3.34 | 55.65 | 3.18 | 6.55 | 21.36 | 15.28 | 26.26 | 56.52 |
| RMSE | 0.3162 | 2.59 | 1.88 | 4.80 | 1.82 | 7.45 | 1.78 | 2.56 | 4.62 | 3.90 | 5.12 | 7.51 |
| PSNR | 58.13 | 39.86 | 42.62 | 34.50 | 42.89 | 30.67 | 43.09 | 39.96 | 34.83 | 36.28 | 33.93 | 30.60 |
| MD | 10 | 73 | 38 | 77 | 11 | 147 | 41 | 25 | 88 | 90 | 100 | 157 |
| NAE | 0.0012 | 0.0220 | 0.0132 | 0.0491 | 0.0161 | 0.1267 | 0.0115 | 0.0216 | 0.0502 | 0.0364 | 0.0549 | 0.1300 |
| NMSE | 5.62e-04 | 0.0381 | 0.0210 | 0.1273 | 0.0185 | 0.3099 | 0.0187 | 0.0372 | 0.1220 | 0.0869 | 0.1501 | 0.3188 |
| SC | 1 | 1 | 1 | 0.98 | 1 | 1.03 | 1 | 0.99 | 1 | 0.98 | 1.02 | 1.08 |
| CC | 1 | 1 | 1 | 0.98 | 1 | 0.97 | 1 | 1 | 0.98 | 0.98 | 1 | 0.9564 |
| NCC | 1 | 0.99 | 1 | 1 | 1 | 0.98 | 1 | 1 | 1 | 1 | 0.98 | 0.9421 |
| IQI | 1 | 0.98 | 0.98 | 0.94 | 0.95 | 0.91 | 0.98 | 1 | 0.96 | 0.96 | 0.96 | 0.9228 |
| SSIM | 1 | 0.95 | 0.97 | 0.82 | 0.96 | 0.86 | 0.97 | 0.92 | 0.89 | 0.87 | 0.91 | 0.7439 |
| CNR | 6.65e-07 | 0.0040 | 0.0014 | 0.0055 | 1.39e-04 | 0.1052 | 0.0012 | 4.89e-04 | 0.0183 | 5.76e-05 | 0.0436 | 0.1032 |
| NI | 1.36e-05 | 1.35e-05 | 1.34e-05 | 1.31e-05 | 1.35e-05 | 1.41e-05 | 1.35e-05 | 1.34e-05 | 1.37e-05 | 1.31e-05 | 1.42e-05 | 1.55e-05 |
| ASNR | 7.33e+04 | 7.37e+04 | 7.42e+04 | 7.62e+04 | 7.37e+04 | 7.06e+04 | 7.37e+04 | 7.41e+04 | 7.31e+04 | 7.62e+04 | 6.99e+04 | 6.42e+04 |
| IV | 2.36e+03 | 2.31e+03 | 2.31e+03 | 2.16e+03 | 2.34e+03 | 1.94e+03 | 2.33e+03 | 2.31e+03 | 2.27e+03 | 2.18e+03 | 2.32e+03 | 2.33e+03 |
| NSD | 1.94e+08 | 1.92e+08 | 1.95e+08 | 1.91e+08 | 1.94e+08 | 1.48e+08 | 1.93e+08 | 1.94e+08 | 1.85e+08 | 1.94e+08 | 1.73e+08 | 1.47e+08 |
| ENL | 7.85e-14 | 7.93e-14 | 7.82e-14 | 7.96e-14 | 7.85e-14 | 1.03e-13 | 7.87e-14 | 7.86e-14 | 8.22e-14 | 7.85e-14 | 8.79e-14 | 1.03e-13 |

| 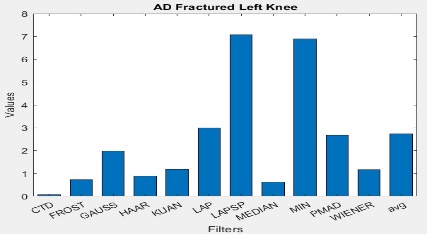  AD | 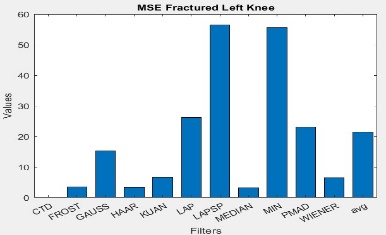  MSE | 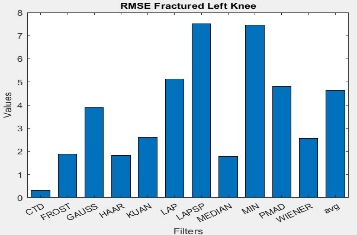  RMSE |
| --- | --- | --- |
| 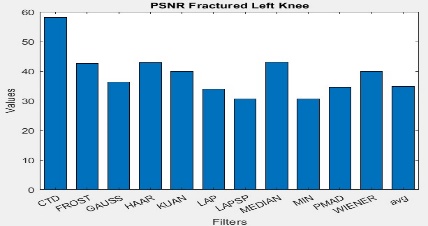  PSNR | 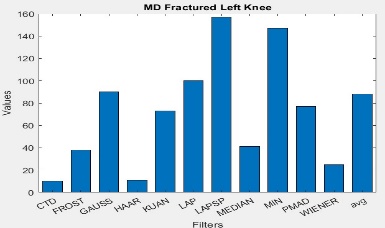  MD | 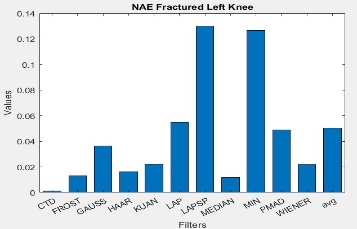  NAE |
| 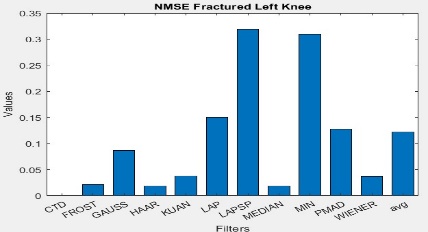  NMSE | 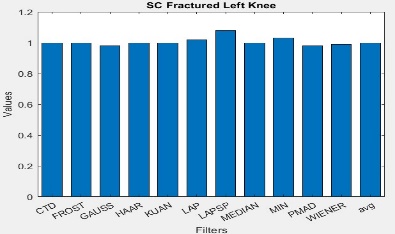  SC | 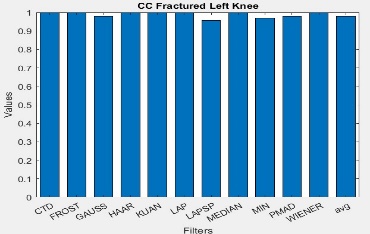  CC |
| 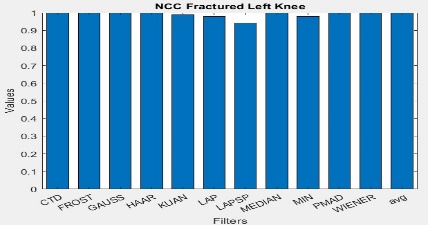  NCC | 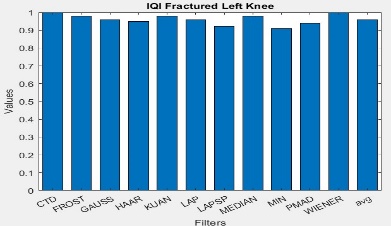  IQI | 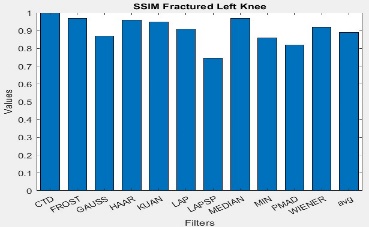  SSIM |
| 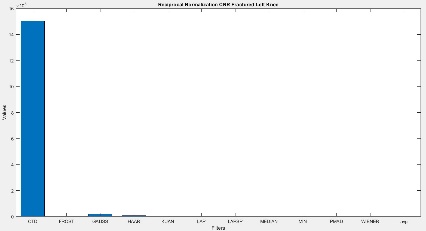  Reciprocal CNR | 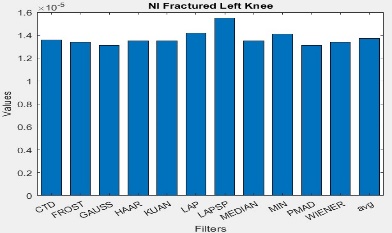  NI | 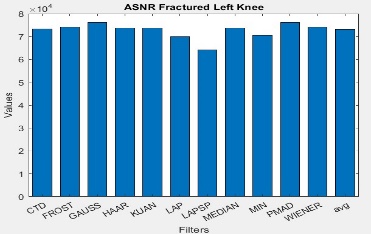  ASNR |
| 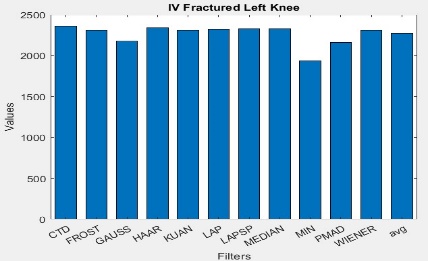  IV | 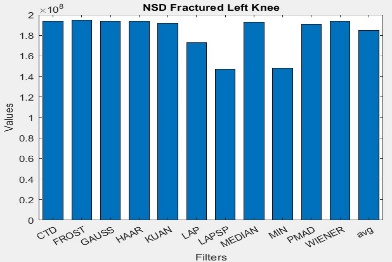  NSD | 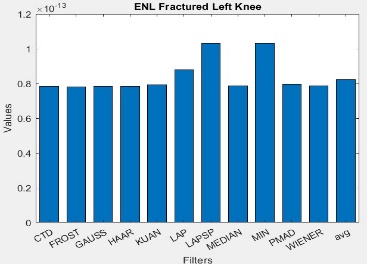  ENL |

**Figure BS2** Histogram plots of the performance metrics of Extensive Intracranial Hemorrhage

Fractured Left Knee: Sagittal MRI Fat Suppressed (FS) Sequence with Bone Edema

In this clinical example, we showcase a sagittal MRI Fat Suppressed (FS) sequence of a fractured left knee also diagnosed with bone edema. The derived conclusions based on filtered images, metrics, and histogram plots for both infarct and extensive intracranial hemorrhage are outlined below:

1. AD, MSE, RMSE, MD, NAE, and NMSE yielded exceptionally small values compared to other filters, contributing to a high-quality denoised MRI image of the fractured knee. This CTD denoised image provides additional details over the bone, fat, cartilage, and meniscus regions, proving crucial for comprehensive diagnostic insights into the fractured knee with bone edema.
2. SC, CC, NCC, IQI, and SSIM consistently resulted in a unity value for the CTD filter. Despite the challenges posed by edema and intricate bone and muscle structures, the CTD denoised fractured knee image maintained a high degree of similarity with zero error.
3. The PSNR for the CTD filter demonstrated an excellent denoised value, affirming the removal of both Gaussian and Rayleigh noise to a greater extent compared to other filters.
4. CNR, NI, ASNR, IV, NSD, and ENL metrics exhibited commendable values for the CTD image, resulting in a noise-free representation with excellent contrast and enhanced radiological features in the bone edema fractured knee MR image.
5. Ultimately, the CTD denoised image showcased outstanding image quality, even in the case of fat-suppressed bone edema.
