# Supplementary material for: Continuum topological derivative - a novel application tool for denoising CT and MRI medical images
Source: BMC Med Imaging. 2024 Jul 24;24:182. doi: 10.1186/s12880-024-01341-1 (PMC11267933; doi:10.1186/s12880-024-01341-1)
Supplement: Supplementary file 11 — Supplementary Material 11. [file 12880_2024_1341_MOESM11_ESM.pdf]

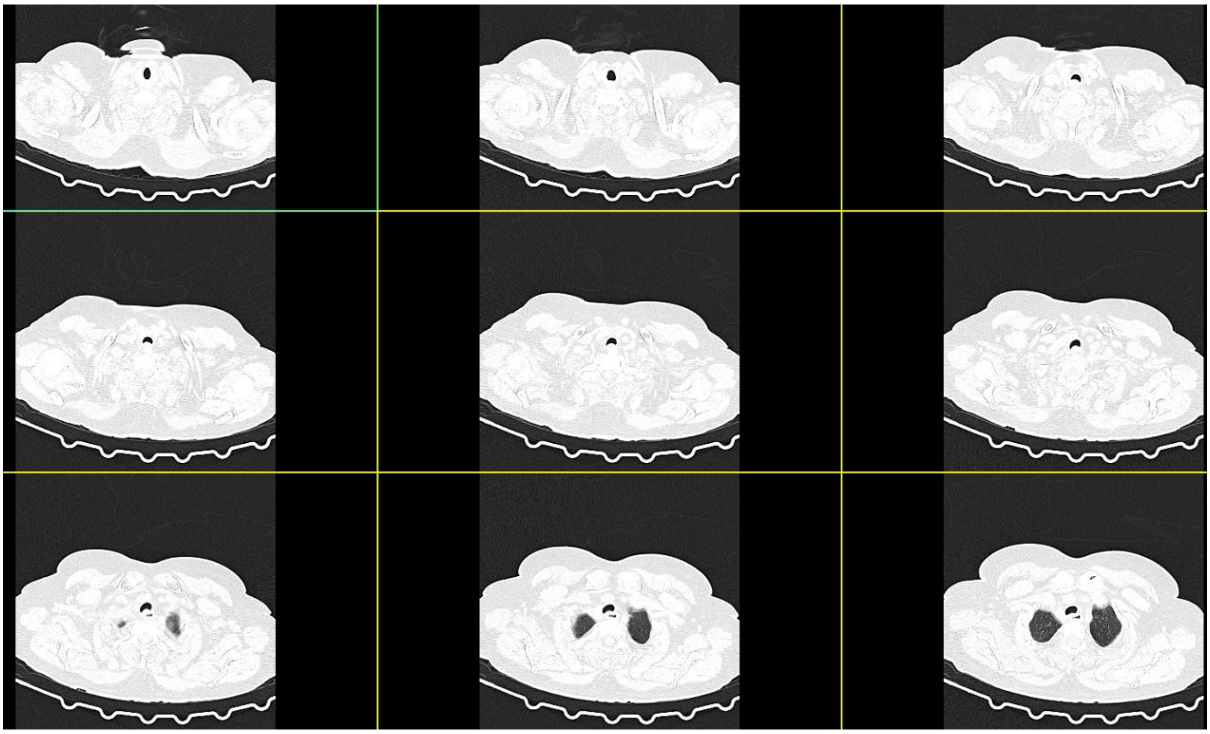

Figure A1 High Resolution Computer Tomography Images (3x3 display) of thoracic cavity - Region of Interest is Lungs

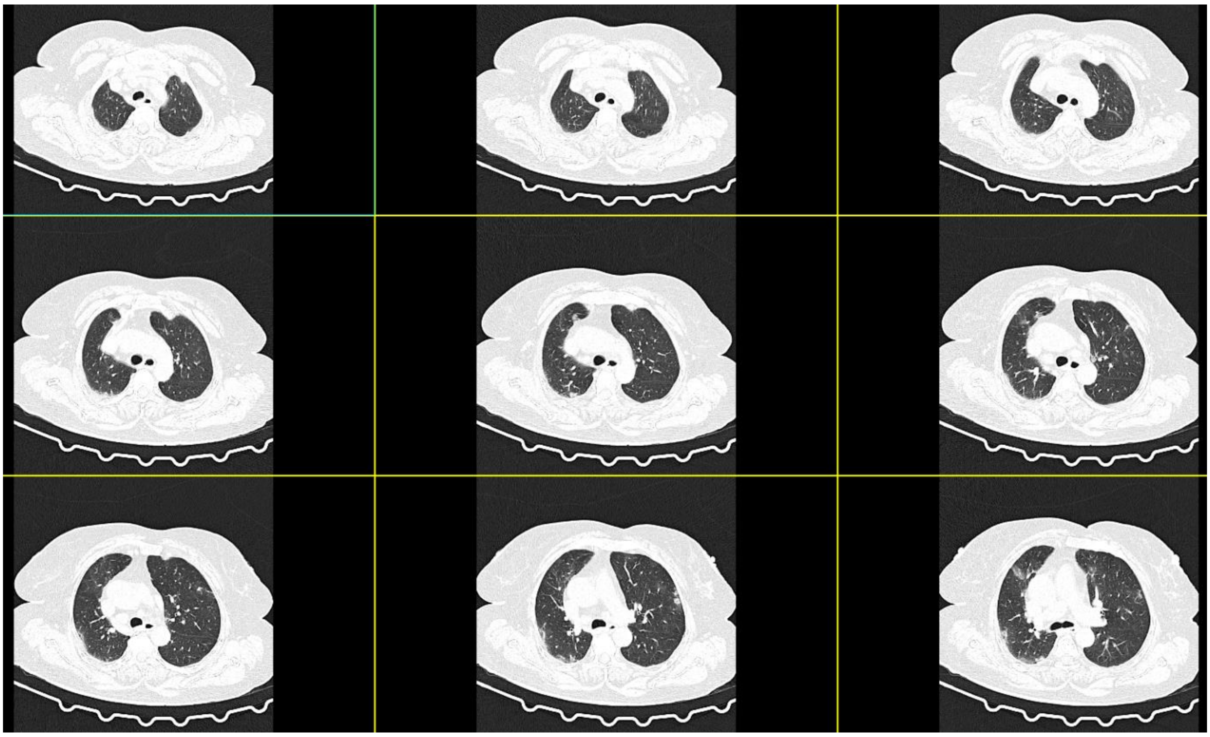

Figure A2 High Resolution Computer Tomography Images (3x3 display) of thoracic cavity - Region of Interest is Lungs

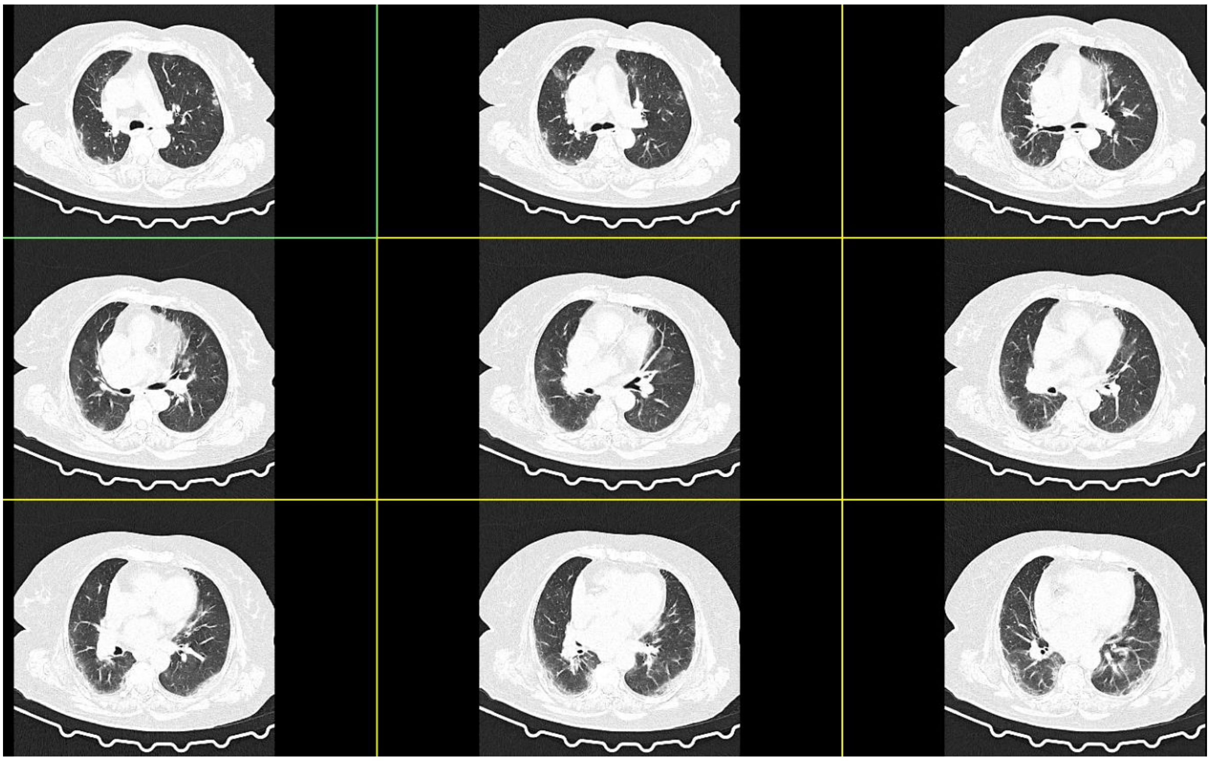

Figure A3 High Resolution Computer Tomography Images (3x3 display) of thoracic cavity - Region of Interest is Lungs

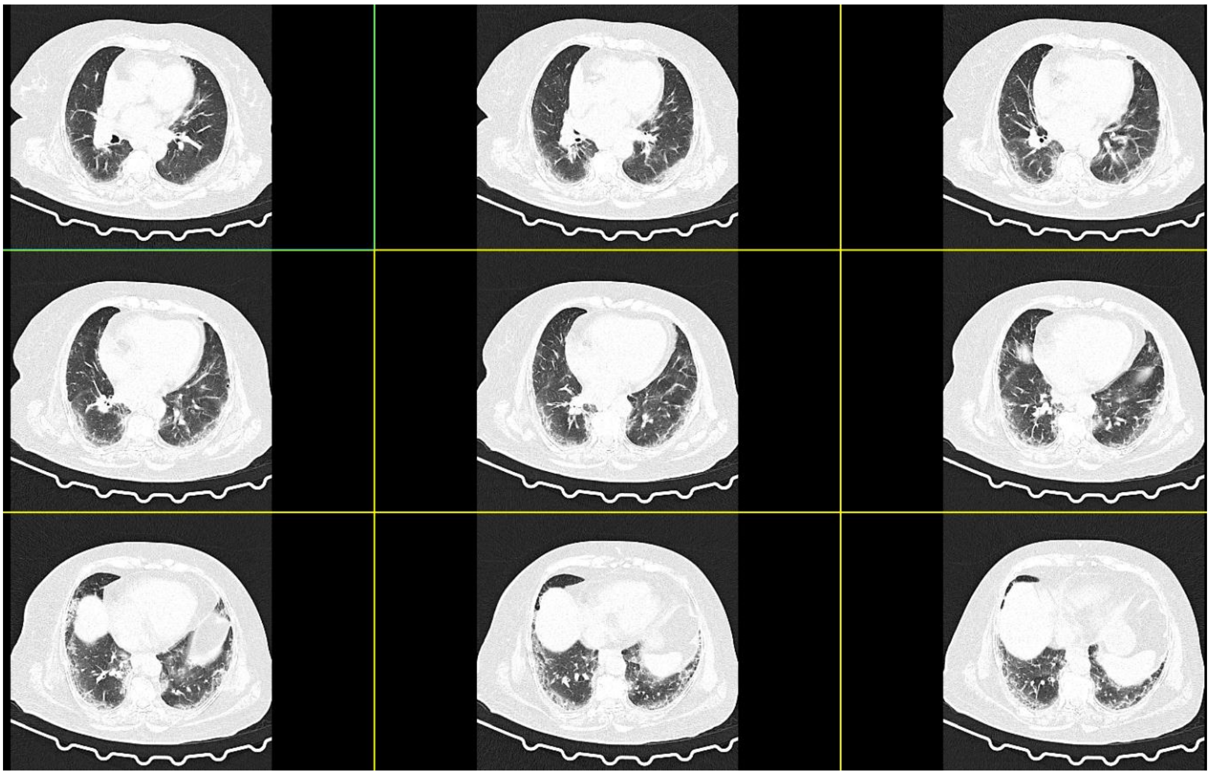

Figure A4 High Resolution Computer Tomography Images (3x3 display) of thoracic cavity - Region of Interest is Lungs

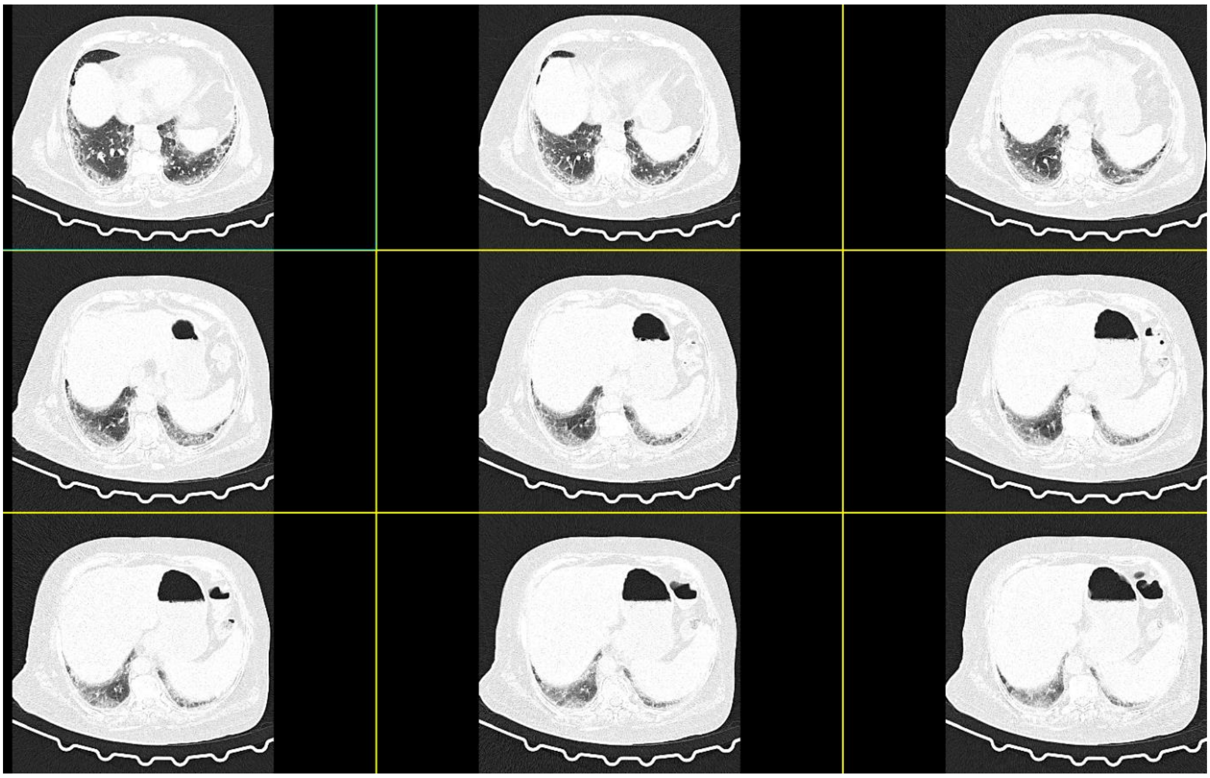

Figure A5 High Resolution Computer Tomography Images (3x3 display) of thoracic cavity - Region of Interest is Lungs

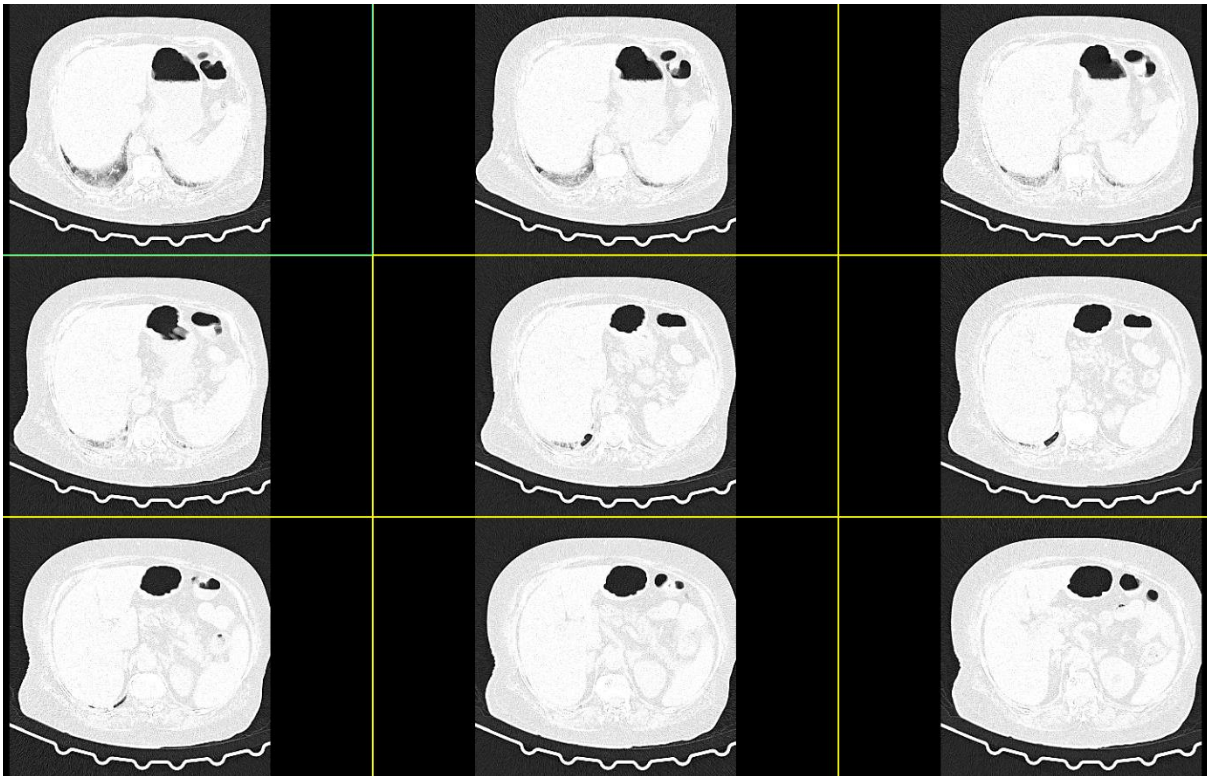

Figure A6 High Resolution Computer Tomography Images (3x3 display) of thoracic cavity - Region of Interest is Lungs

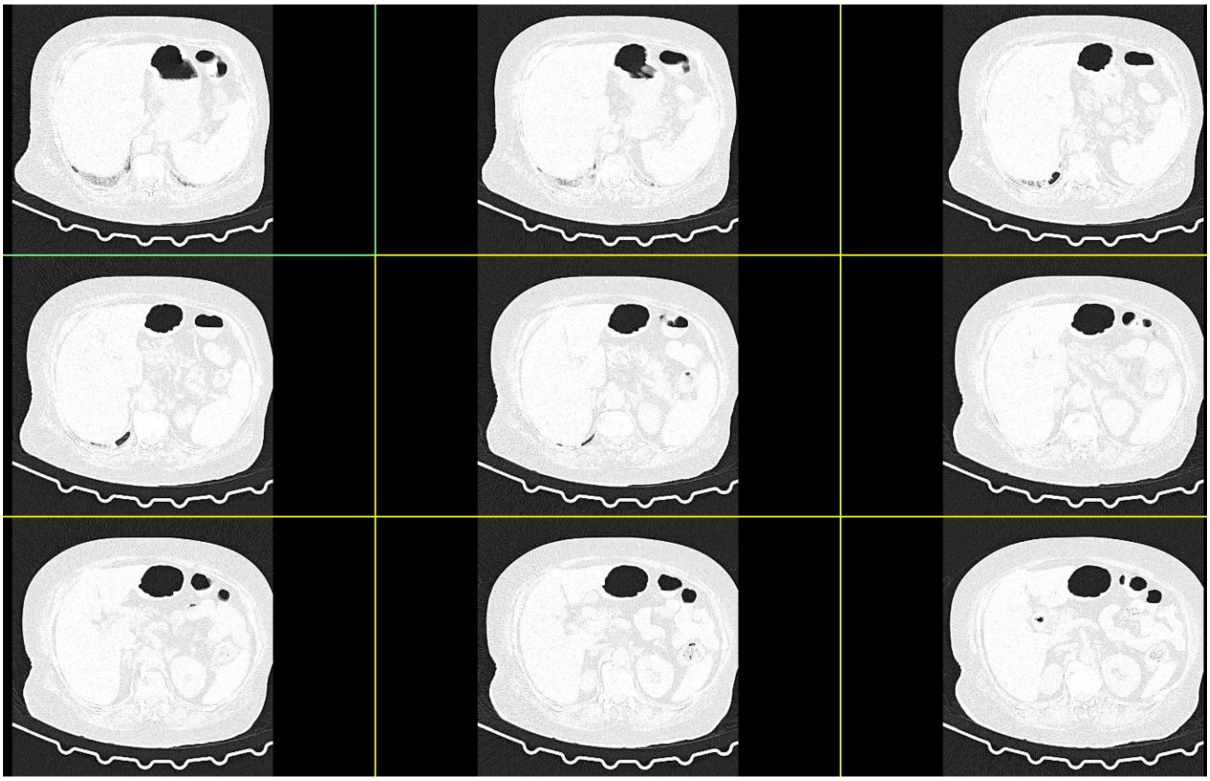

Figure A7 High Resolution Computer Tomography Images (3x3 display) of thoracic cavity - Region of Interest is Lungs

All the above figures of Thoracic cavity (figure A1-A7), are HRCT images acquired at improved low contrast resolution for better image impression

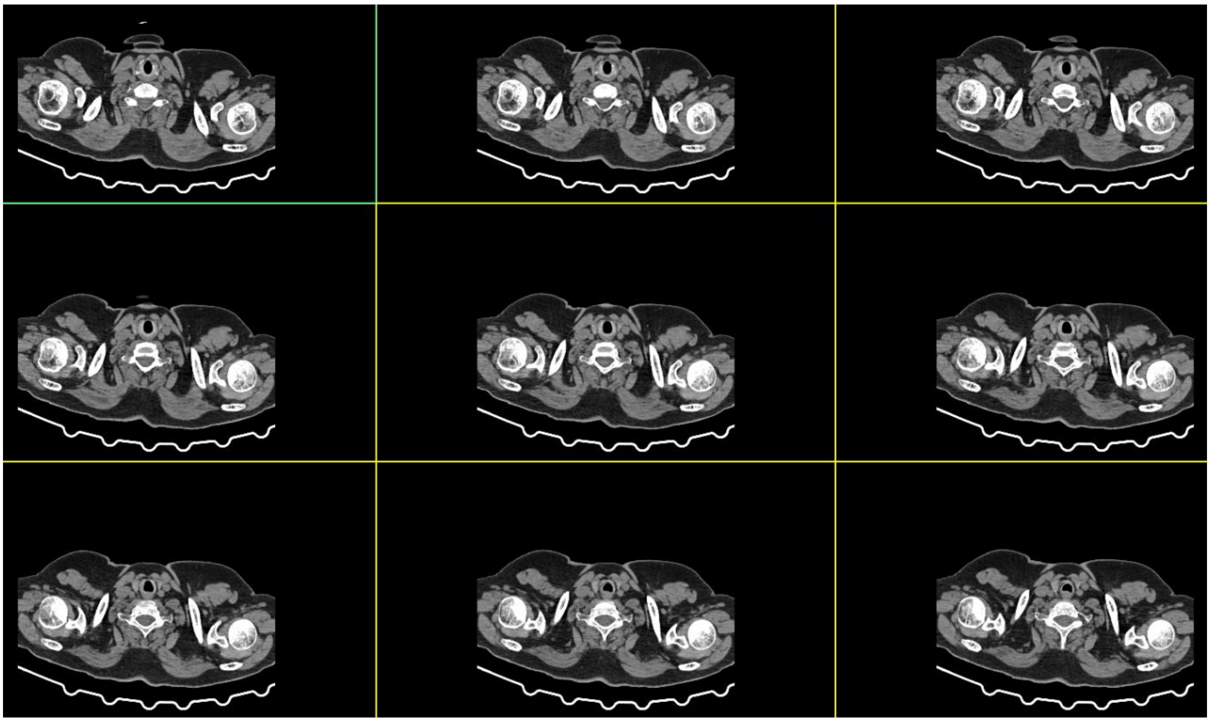

Figure B1 High Resolution Computer Tomography Images (3x3 display) of thoracic cavity - Region of Interest is Trachea and Carina

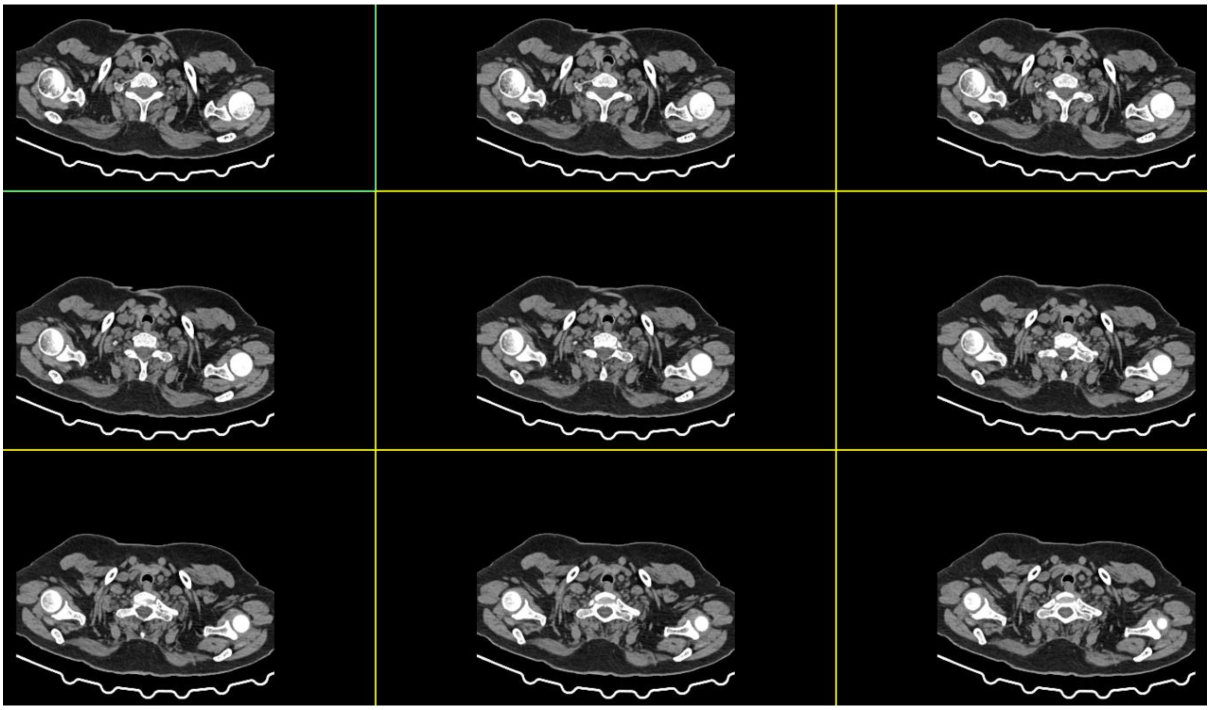

Figure B2 High Resolution Computer Tomography Images (3x3 display) of thoracic cavity - Region of Interest is Trachea and Carina

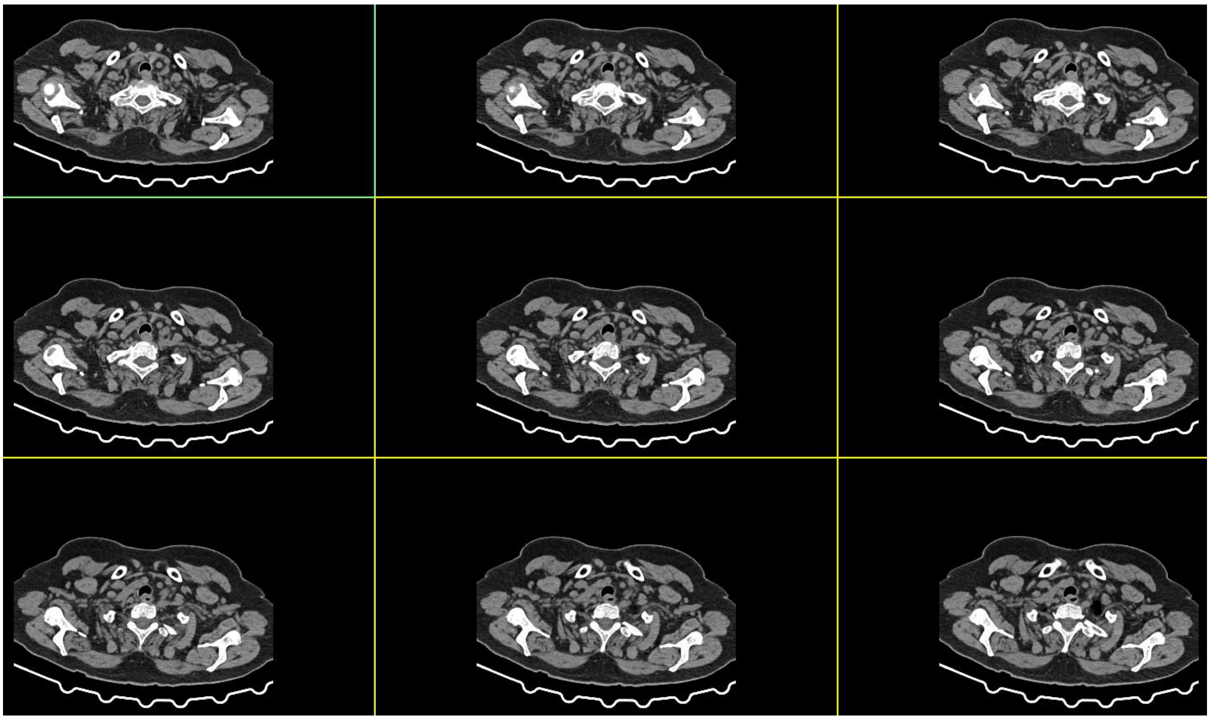

Figure B3 High Resolution Computer Tomography Images (3x3 display) of thoracic cavity - Region of Interest is Trachea and Carina

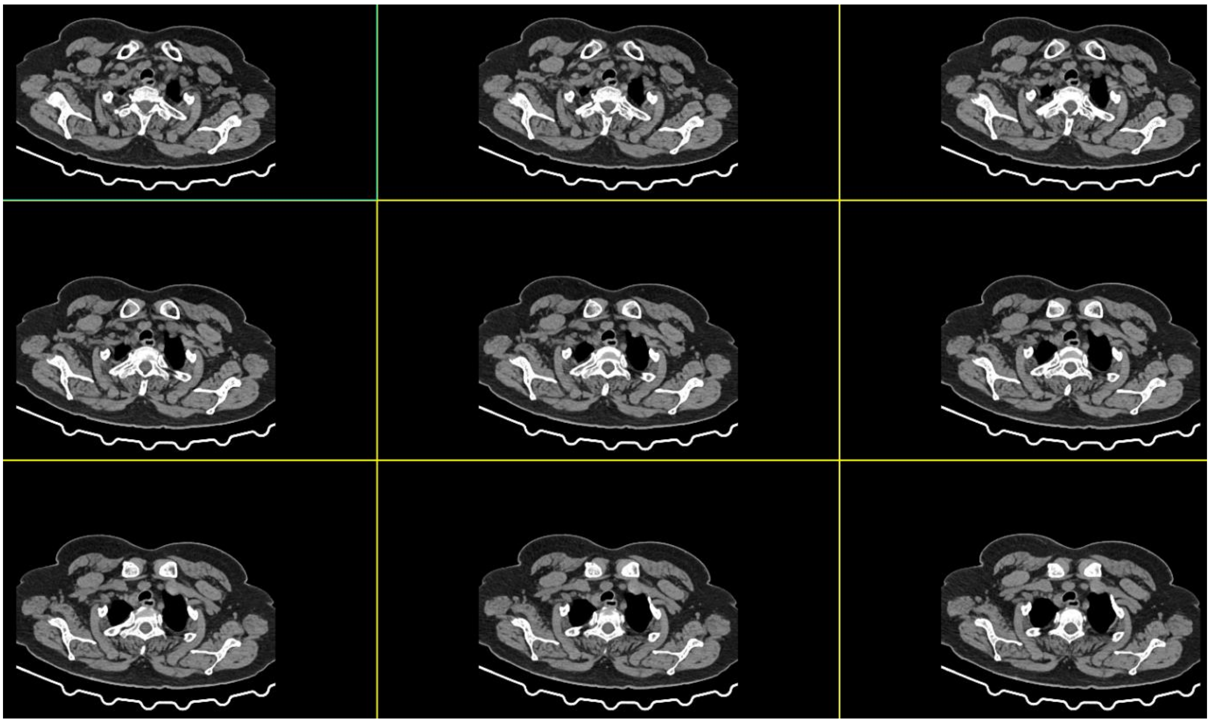

Figure B4 High Resolution Computer Tomography Images (3x3 display) of thoracic cavity - Region of Interest is Trachea and Carina

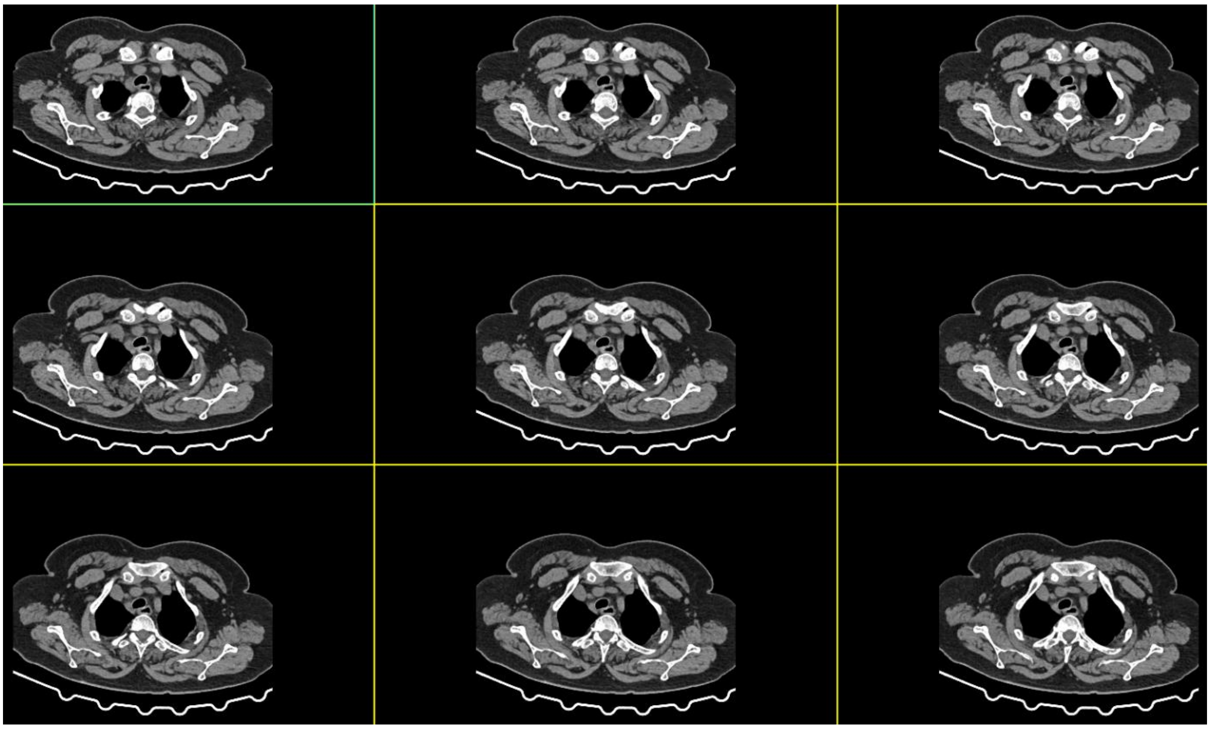

Figure B5 High Resolution Computer Tomography Images (3x3 display) of thoracic cavity - Region of Interest is Trachea and Carina

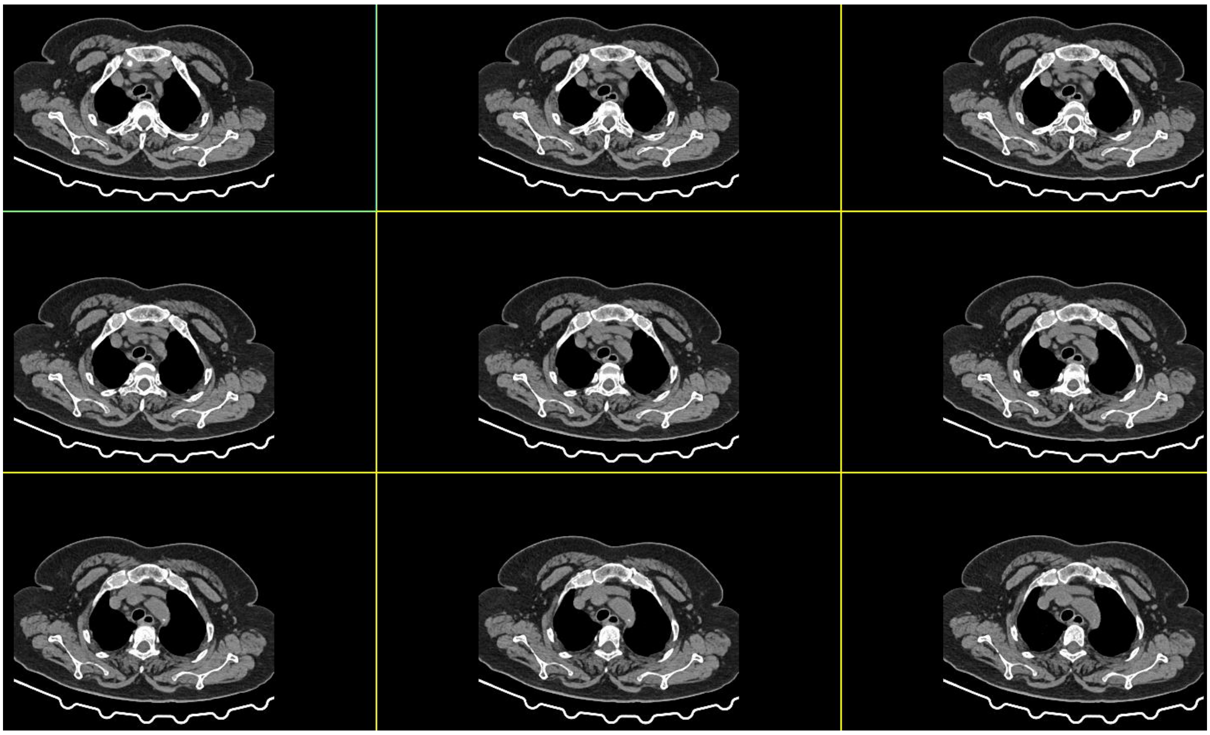

Figure B6 High Resolution Computer Tomography Images (3x3 display) of thoracic cavity - Region of Interest is Trachea and Carina

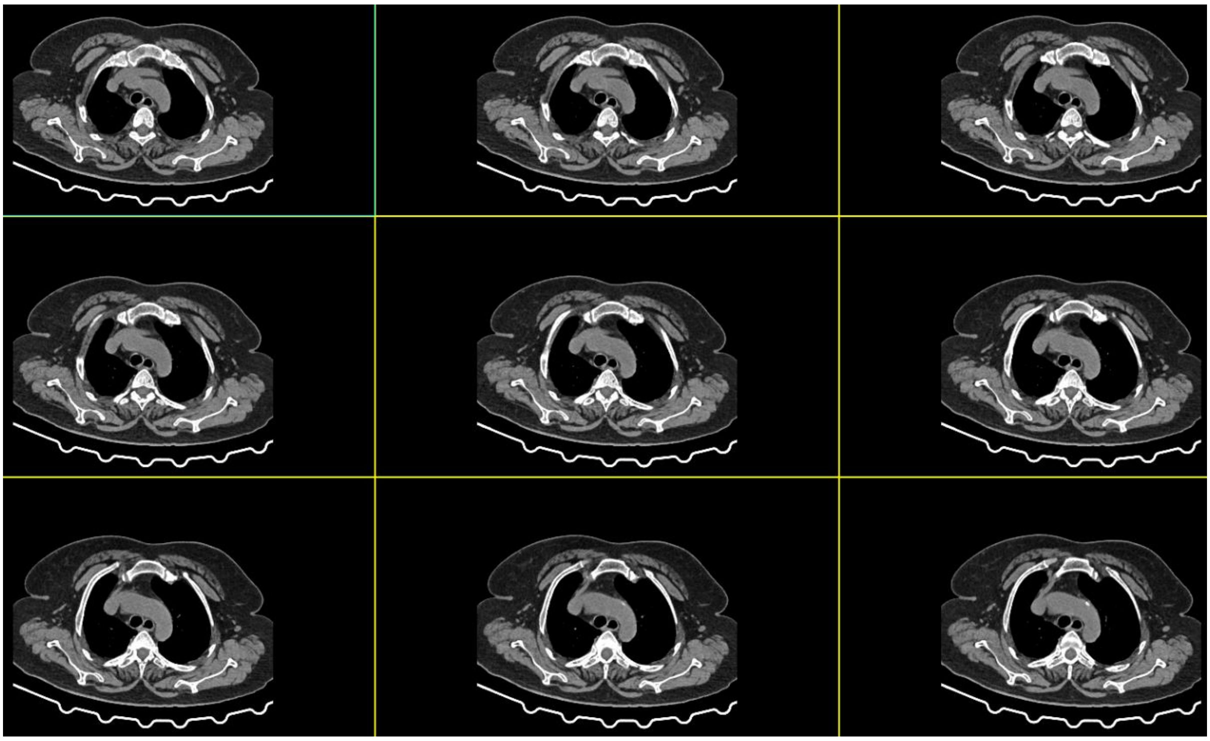

Figure B7 High Resolution Computer Tomography Images (3x3 display) of thoracic cavity - Region of Interest is Trachea and Carina

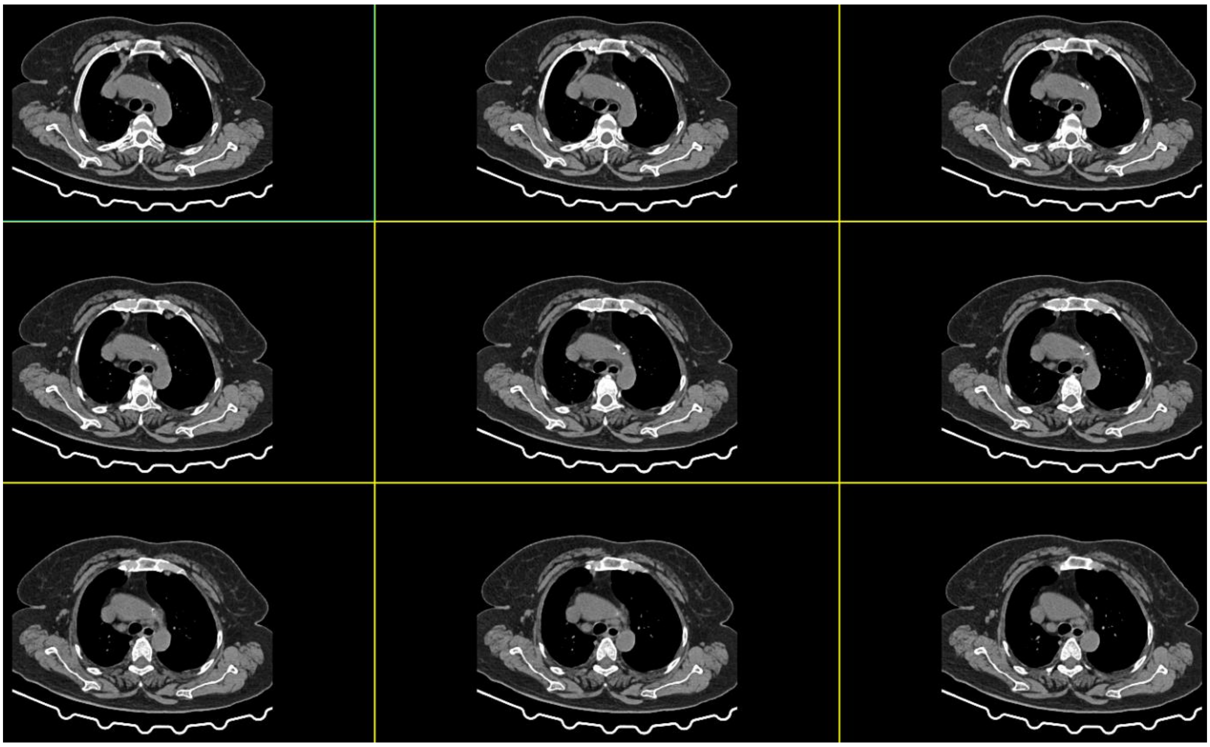

Figure B8 High Resolution Computer Tomography Images (3x3 display) of thoracic cavity - Region of Interest is Trachea and Carina

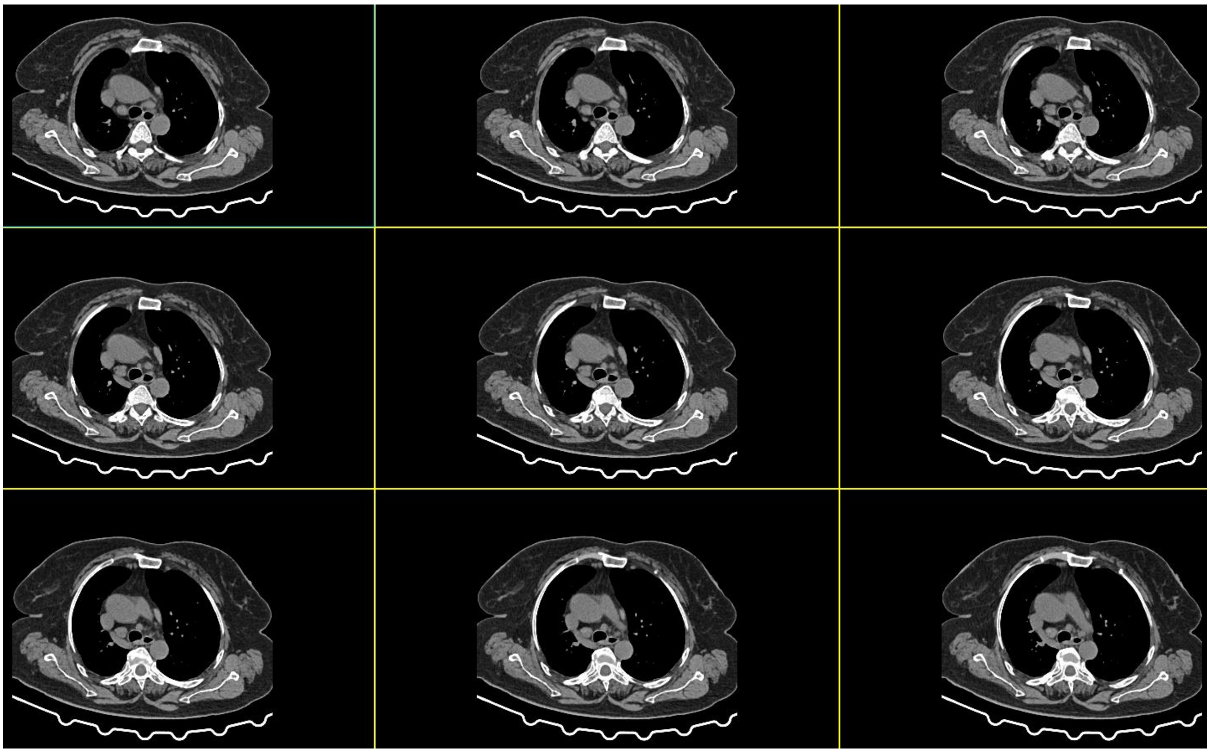

Figure B9 High Resolution Computer Tomography Images (3x3 display) of thoracic cavity - Region of Interest is Trachea and Carina

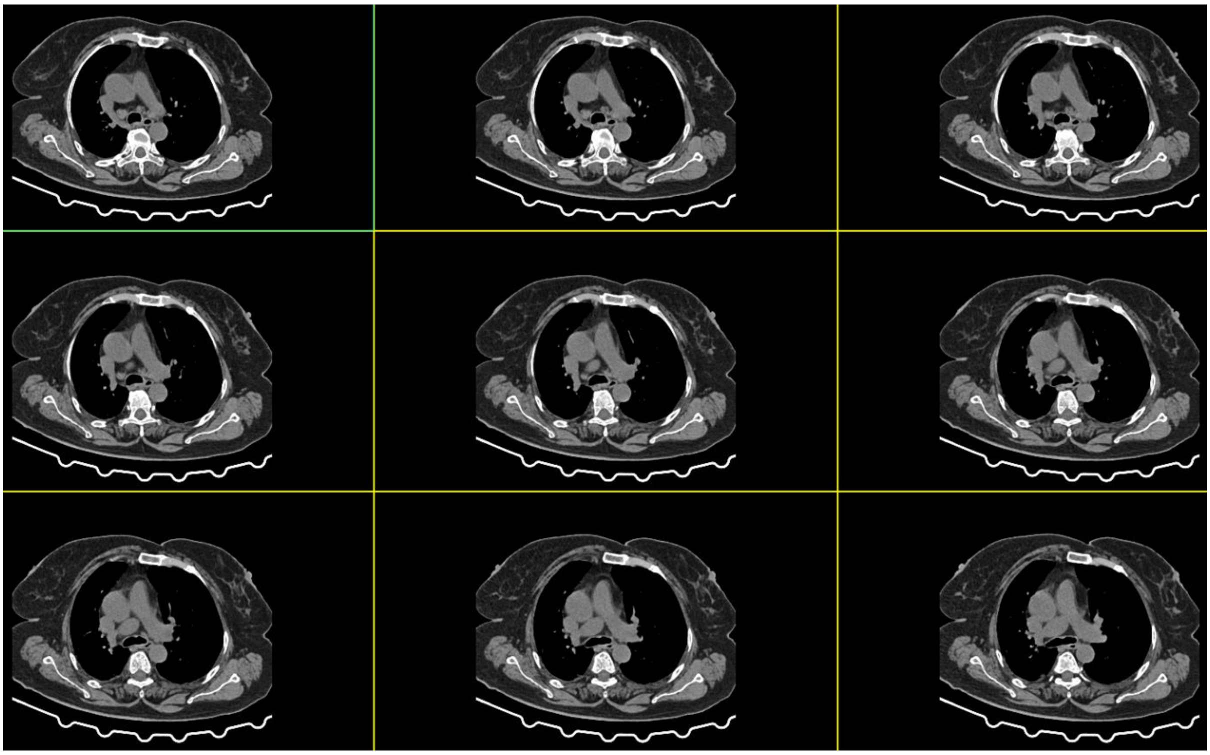

Figure B10 High Resolution Computer Tomography Images (3x3 display) of thoracic cavity - Region of Interest is Trachea and Carina

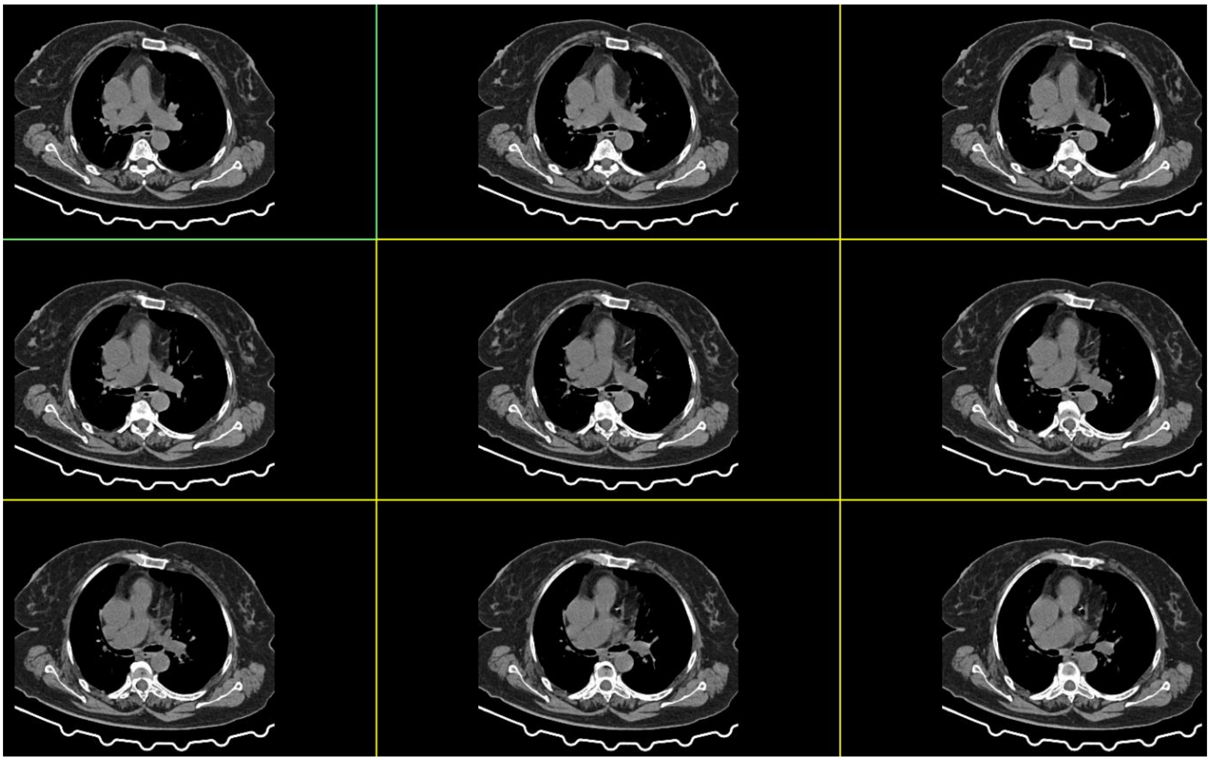

Figure B11 High Resolution Computer Tomography Images (3x3 display) of thoracic cavity - Region of Interest is Trachea and Carina, Lungs

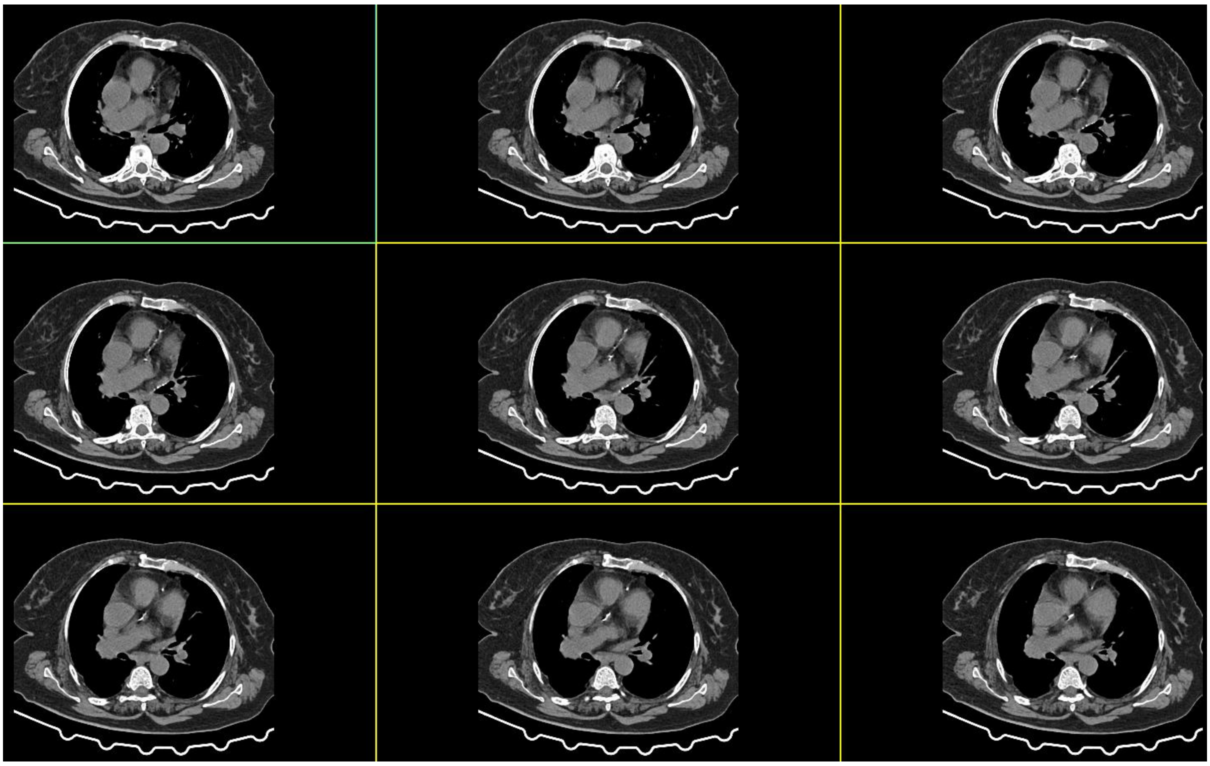

Figure B12 High Resolution Computer Tomography Images (3x3 display) of thoracic cavity - Region of Interest is Lungs

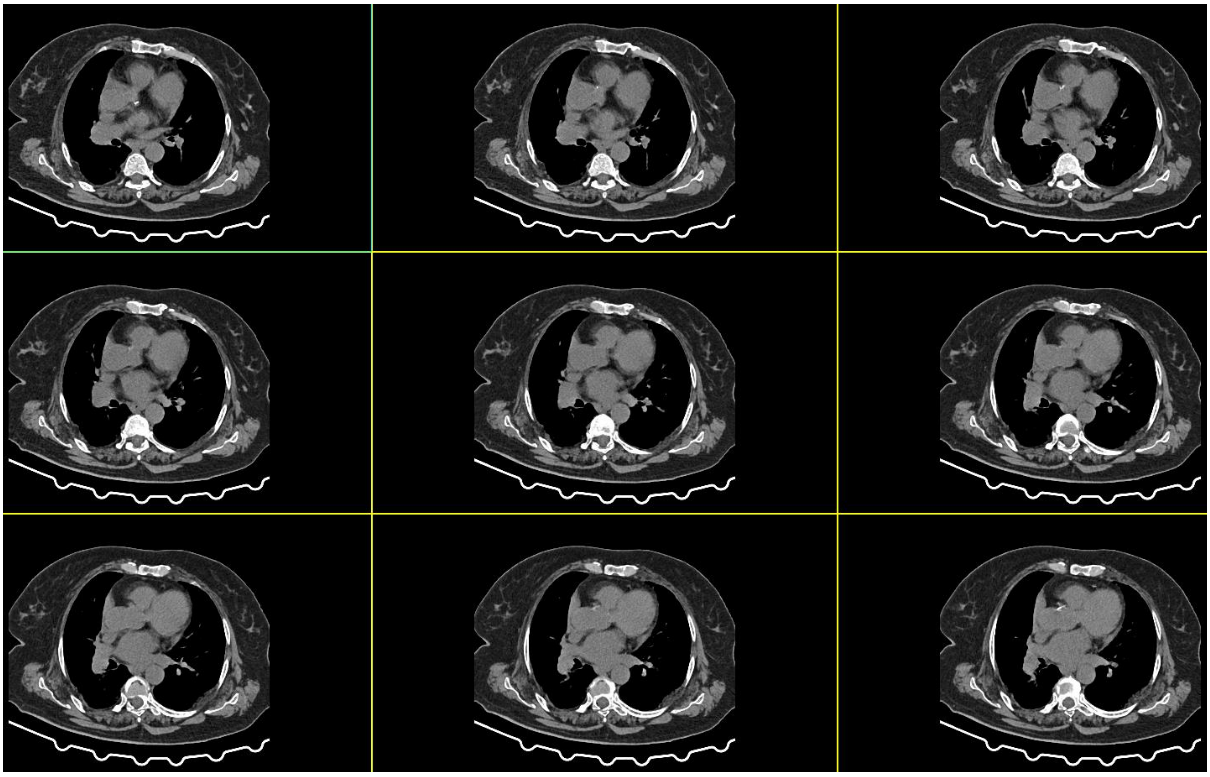

Figure B13 High Resolution Computer Tomography Images (3x3 display) of thoracic cavity - Region of Interest is Lungs

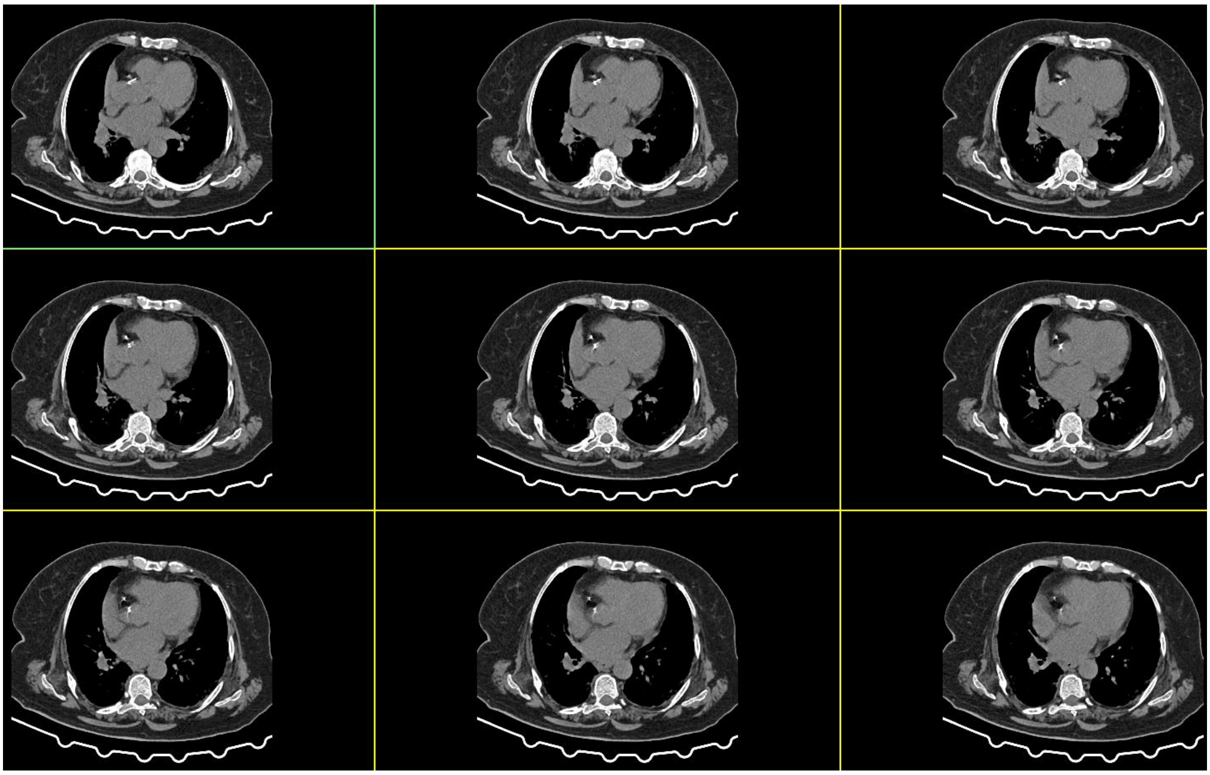

Figure B14 High Resolution Computer Tomography Images (3x3 display) of thoracic cavity - Region of Interest is Lungs

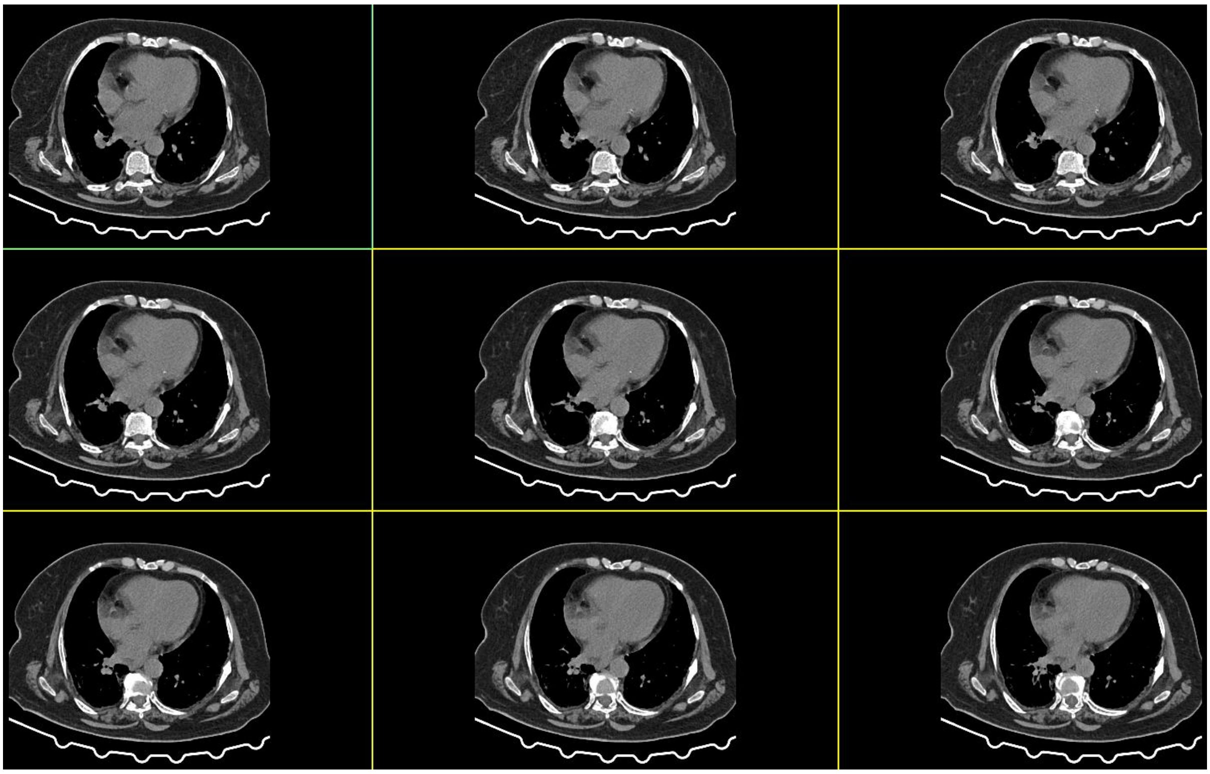

Figure B15 High Resolution Computer Tomography Images 3x3 display, (3x3 display) of thoracic cavity - Region of Interest is Lungs

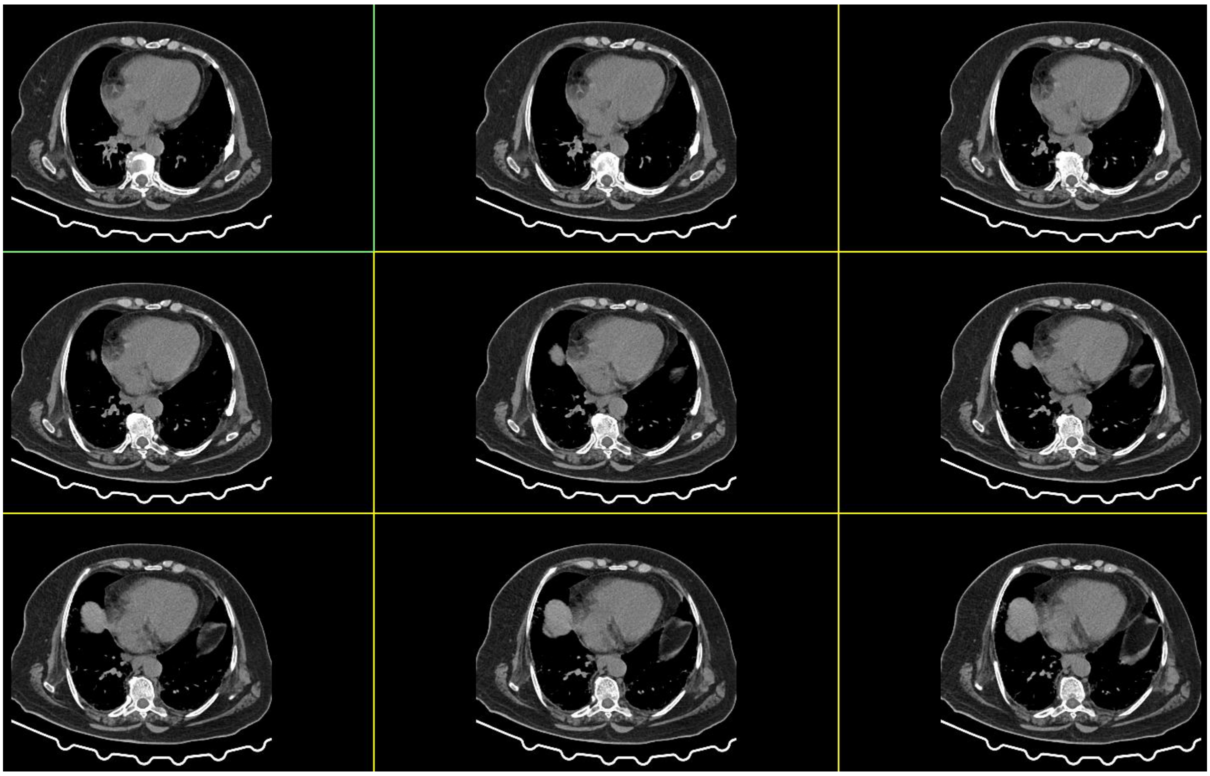

Figure B16 High Resolution Computer Tomography Images (3x3 display) of thoracic cavity - Region of Interest is Lungs

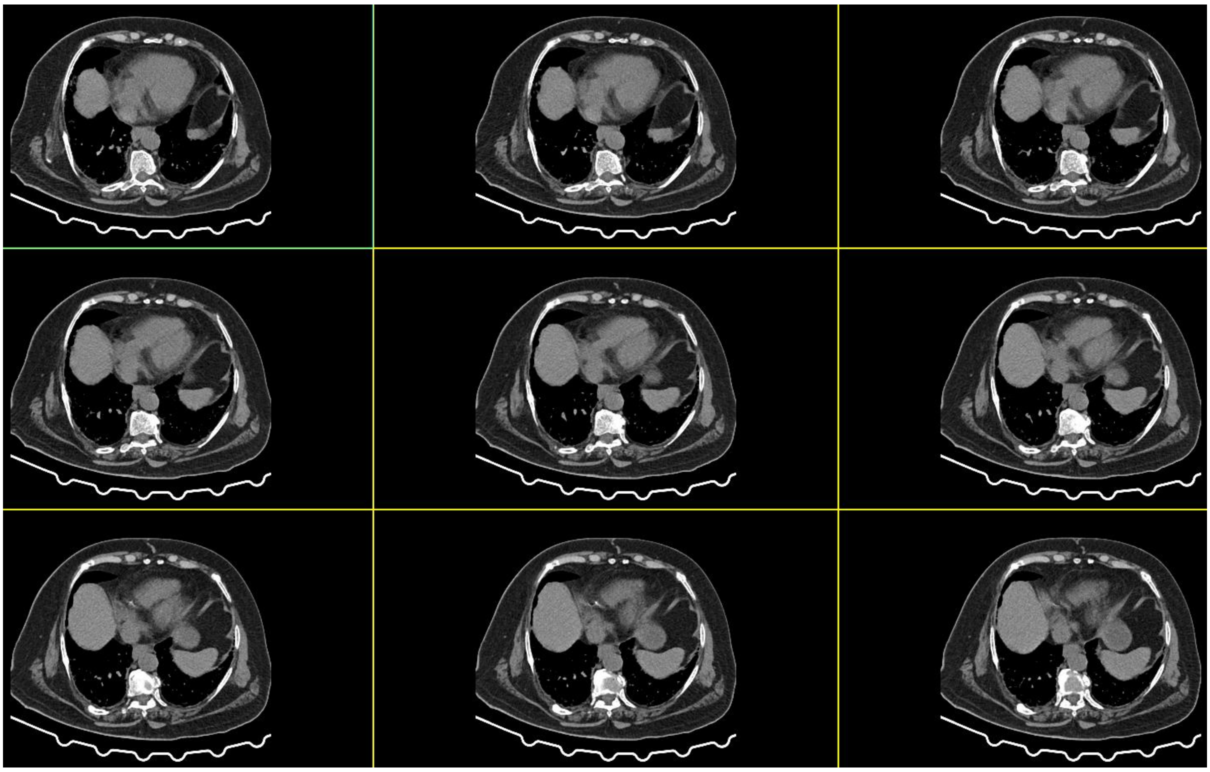

Figure B17 High Resolution Computer Tomography Images (3x3 display) of thoracic cavity - Region of Interest is Lungs

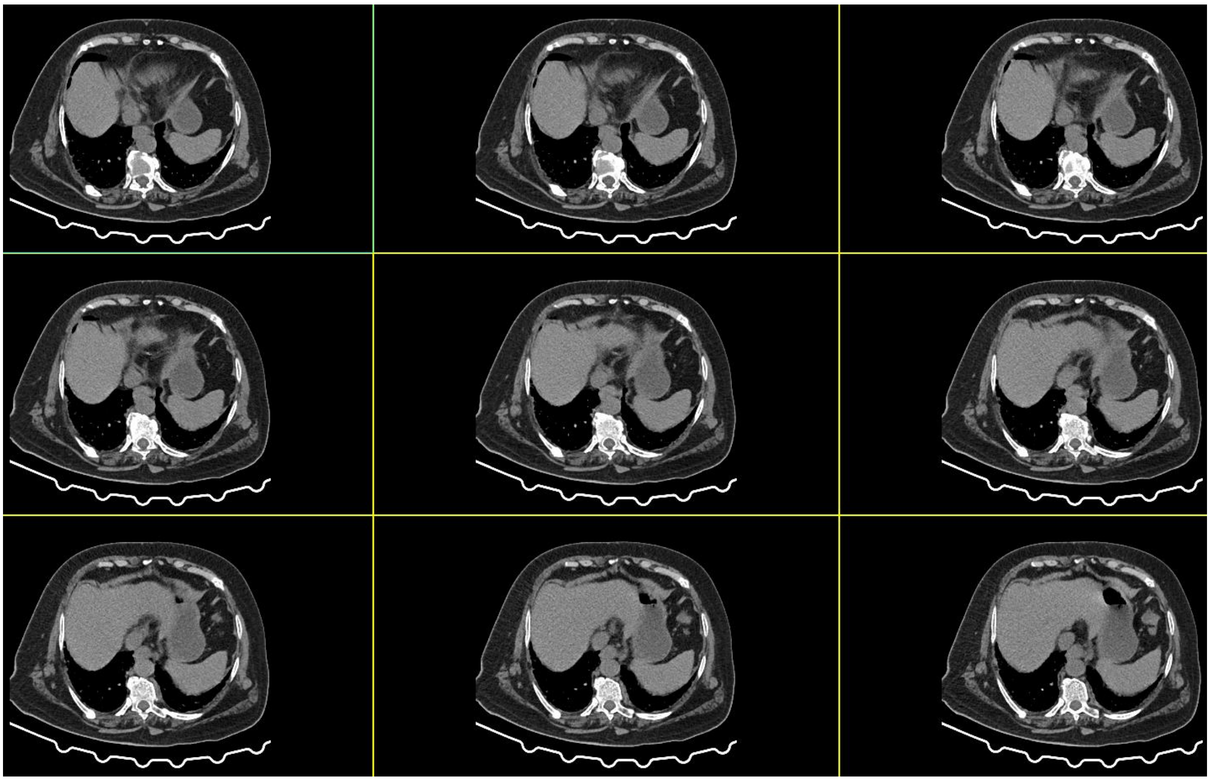

Figure B18 High Resolution Computer Tomography Images (3x3 display) of thoracic cavity - Region of Interest is Lungs and Vertebral column

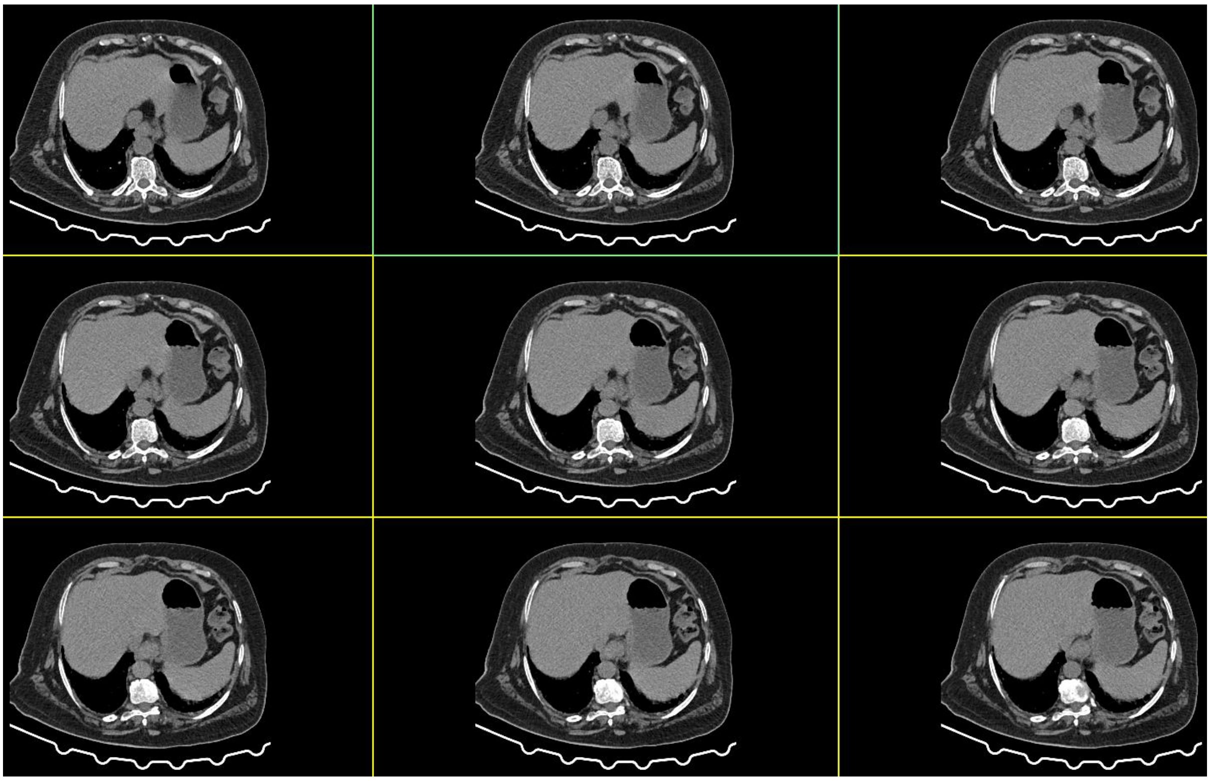

Figure B19 High Resolution Computer Tomography Images (3x3 display) of thoracic cavity - Region of Interest is Vertebral column

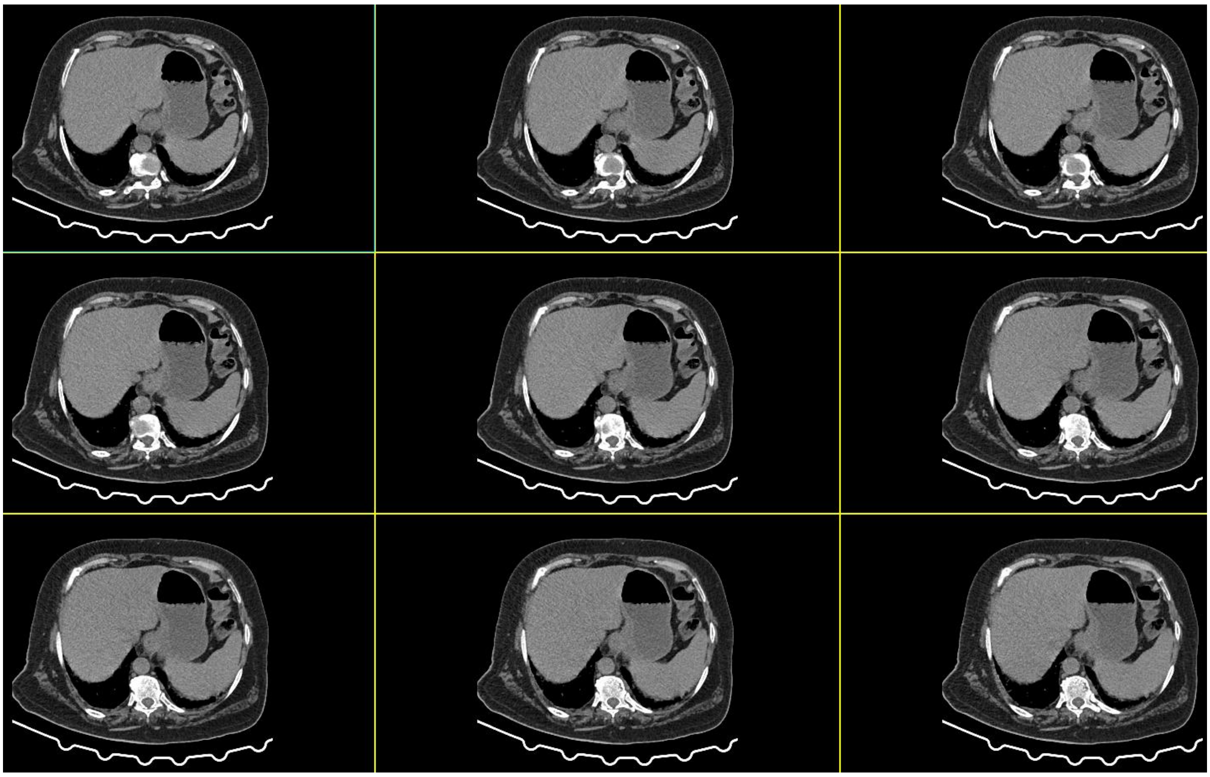

Figure B20 High Resolution Computer Tomography Images (3x3 display) of thoracic cavity - Region of Interest is Vertebral column

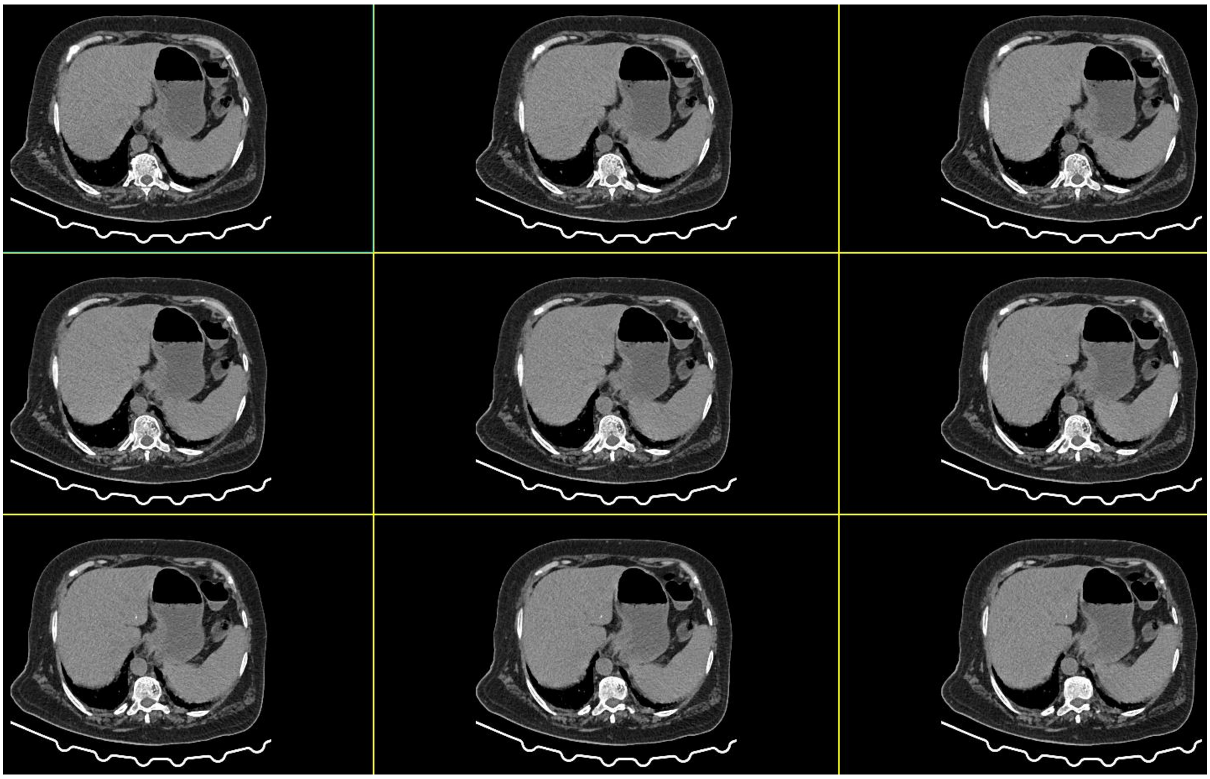

Figure B21 High Resolution Computer Tomography Images (3x3 display) of thoracic cavity - Region of Interest is Vertebral column

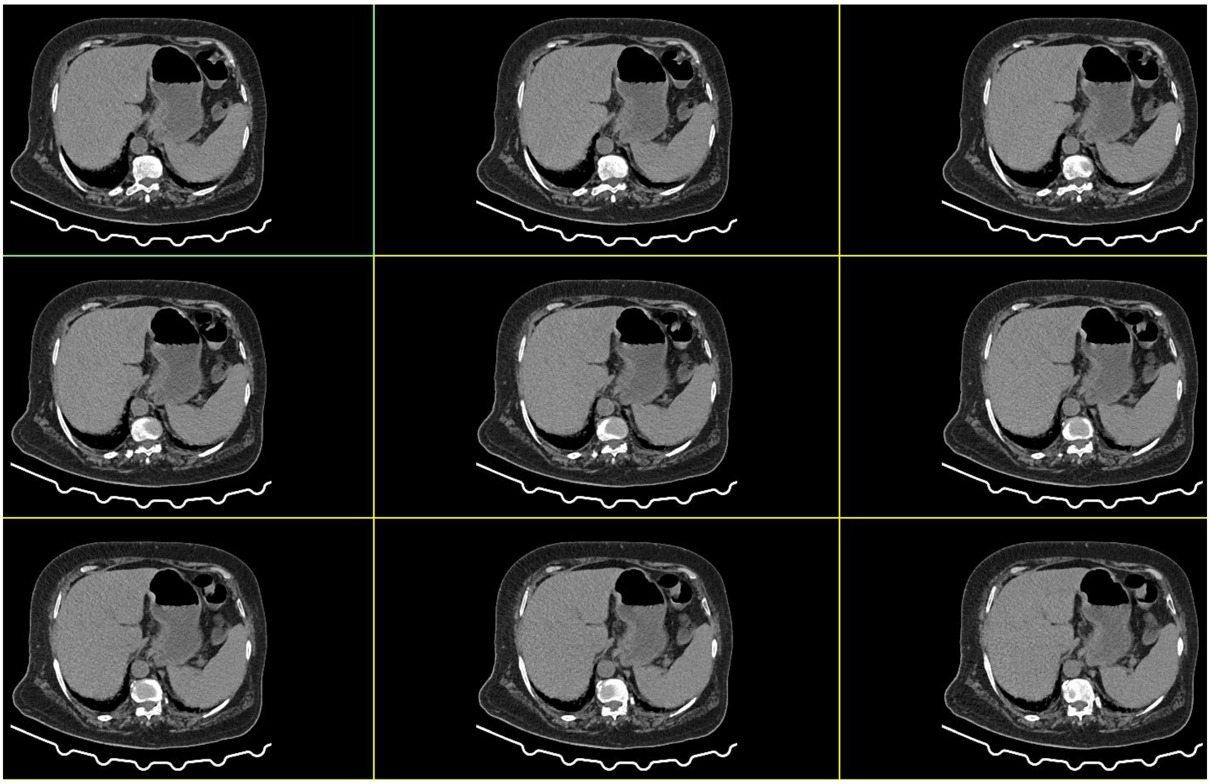

Figure B22 High Resolution Computer Tomography Images (3x3 display) of thoracic cavity - Region of Interest is Vertebral column

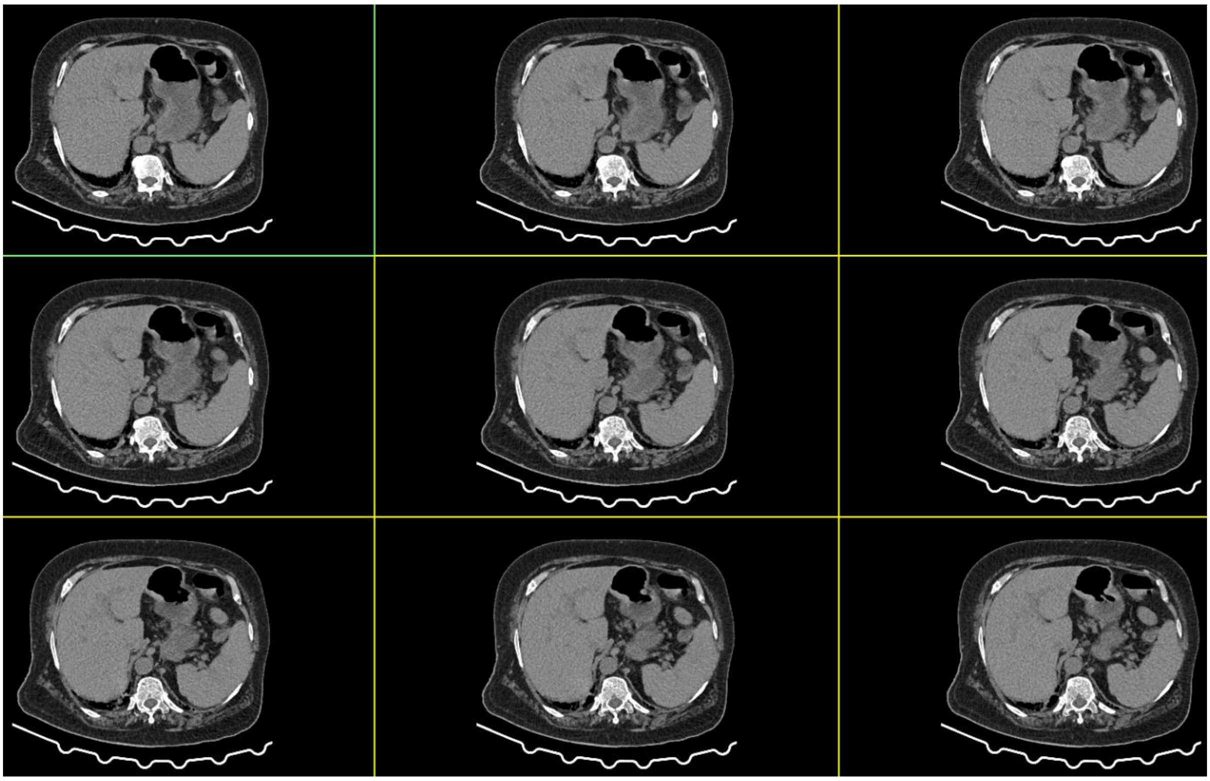

Figure B23 High Resolution Computer Tomography Images (3x3 display) of thoracic cavity - Region of Interest is Vertebral column

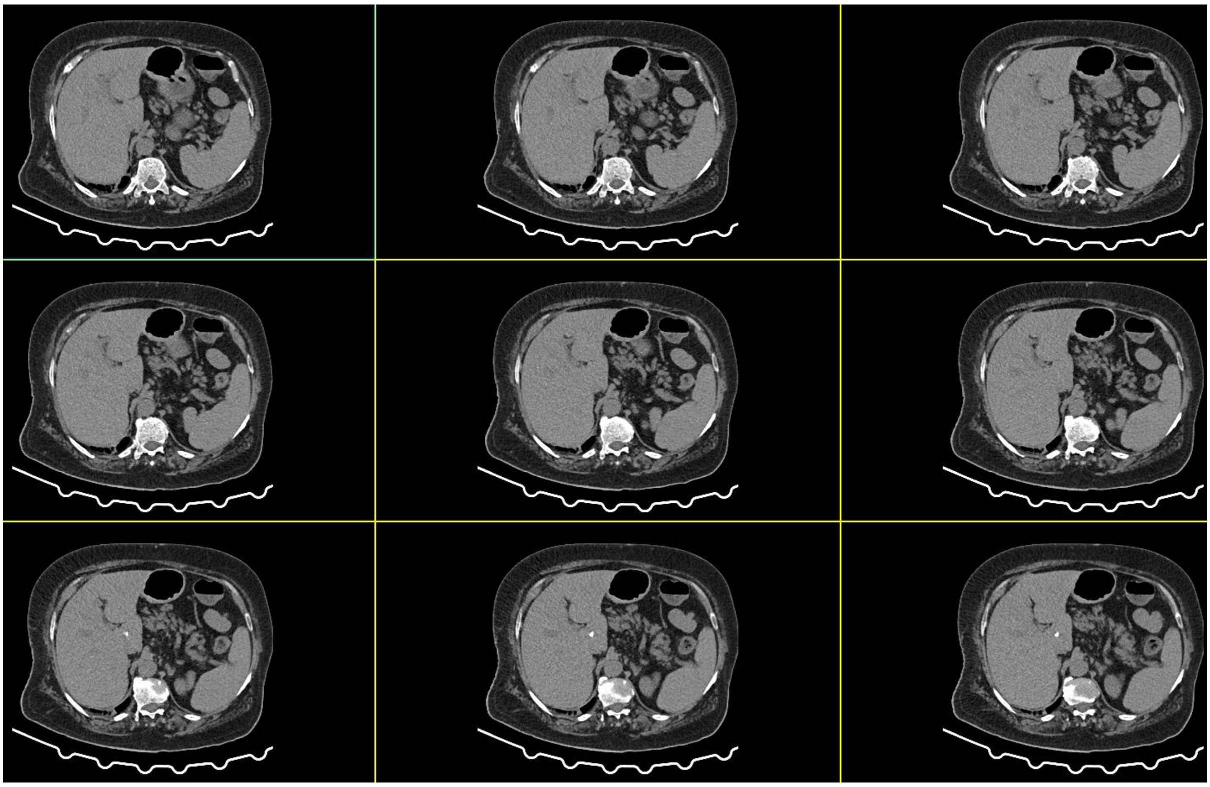

Figure B24 High Resolution Computer Tomography Images (3x3 display) of thoracic cavity - Region of Interest is Vertebral column

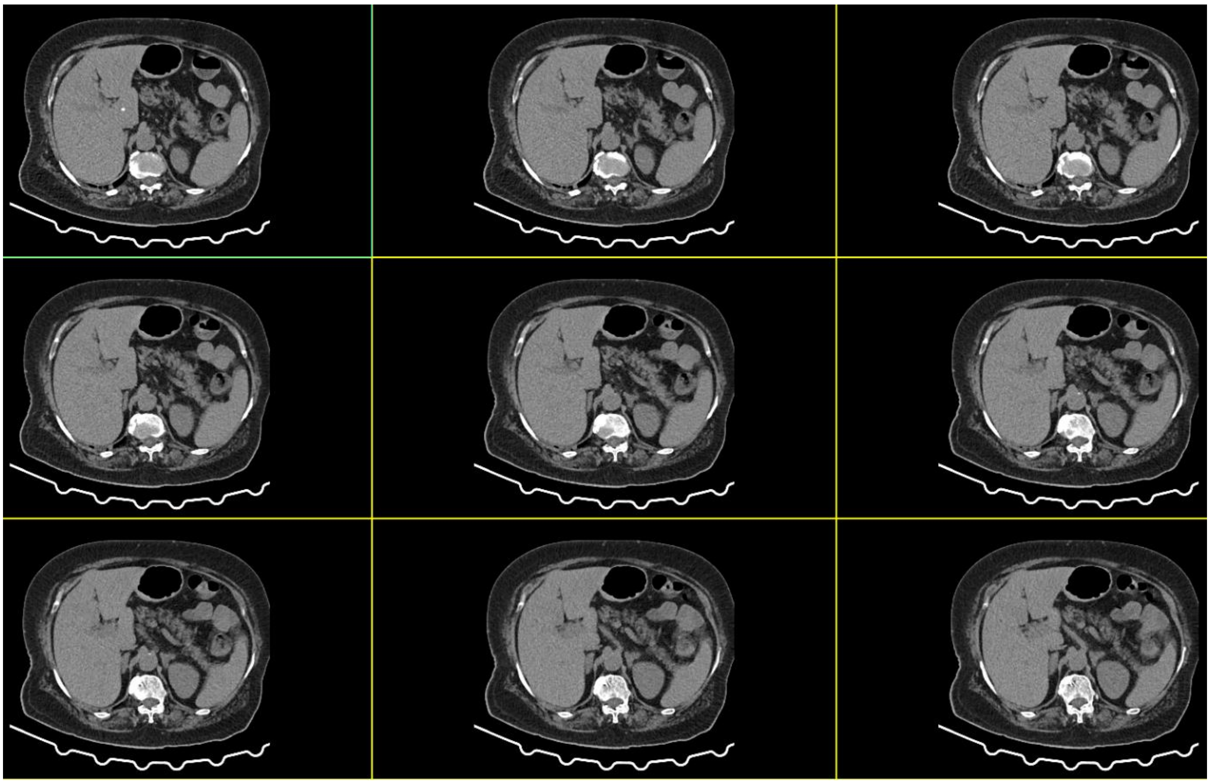

Figure B25 High Resolution Computer Tomography Images (3x3 display) of thoracic cavity - Region of Interest is Vertebral column

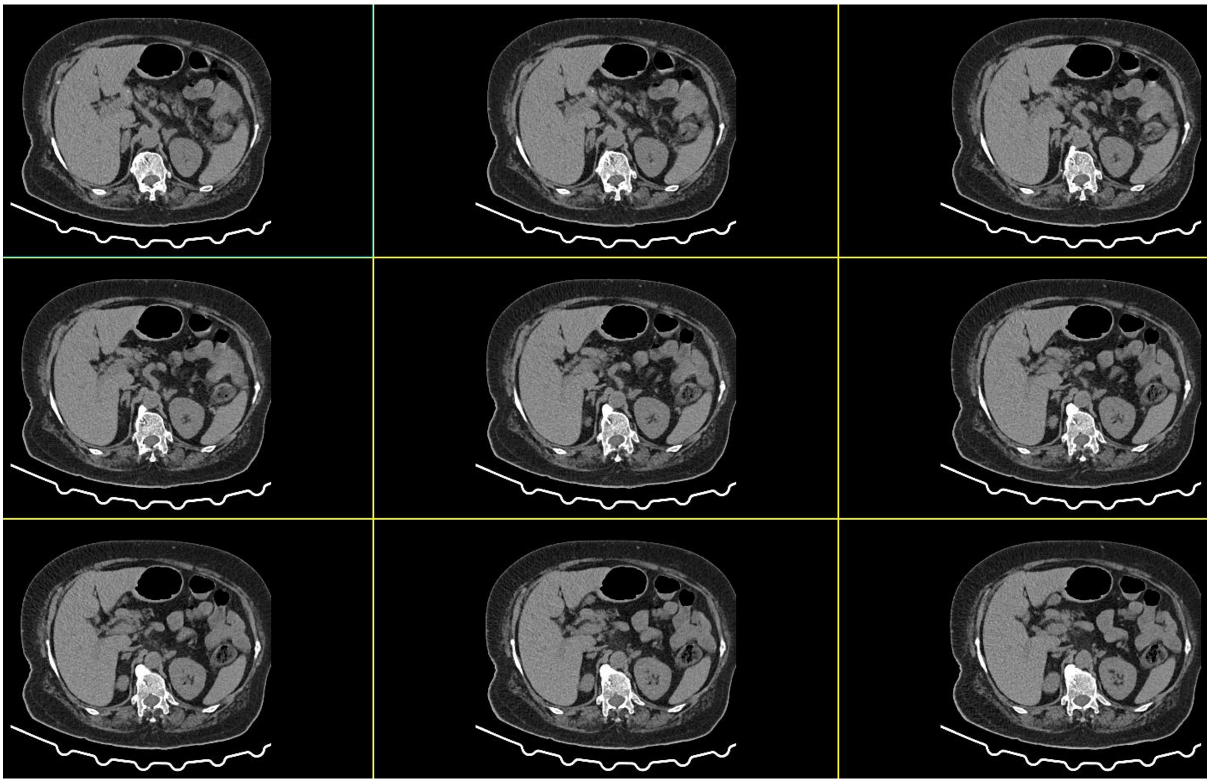

Figure B26 High Resolution Computer Tomography Images (3x3 display) of thoracic cavity - Region of Interest is Vertebral column

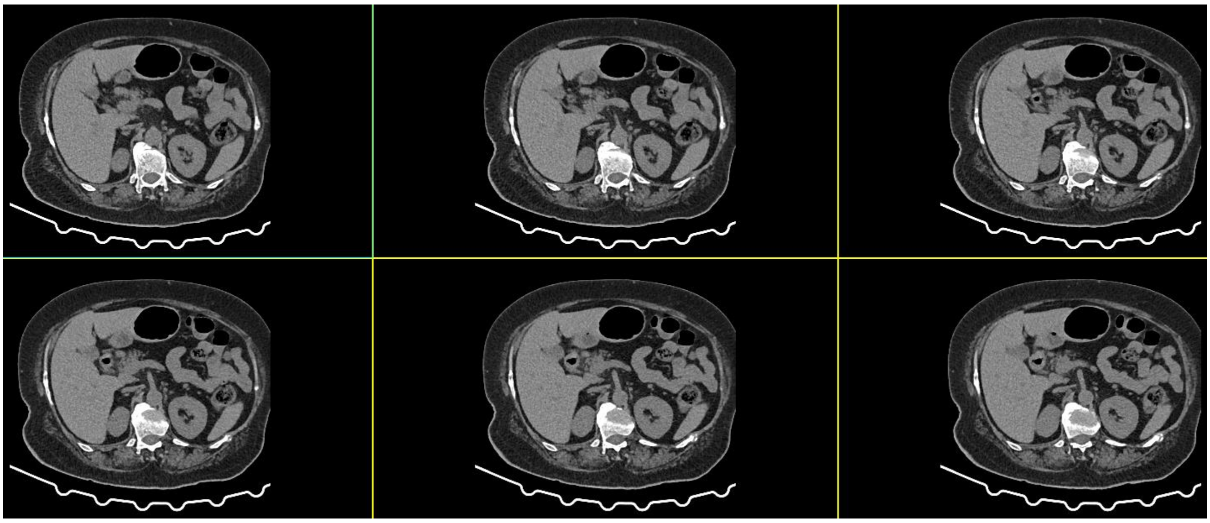

Figure B27 High Resolution Computer Tomography Images (3x3 display) of thoracic cavity - Region of Interest is Vertebral column

All the above figures of Thoracic cavity (figure B1-B27), are HRCT images acquired at improved low contrast resolution for better image impression

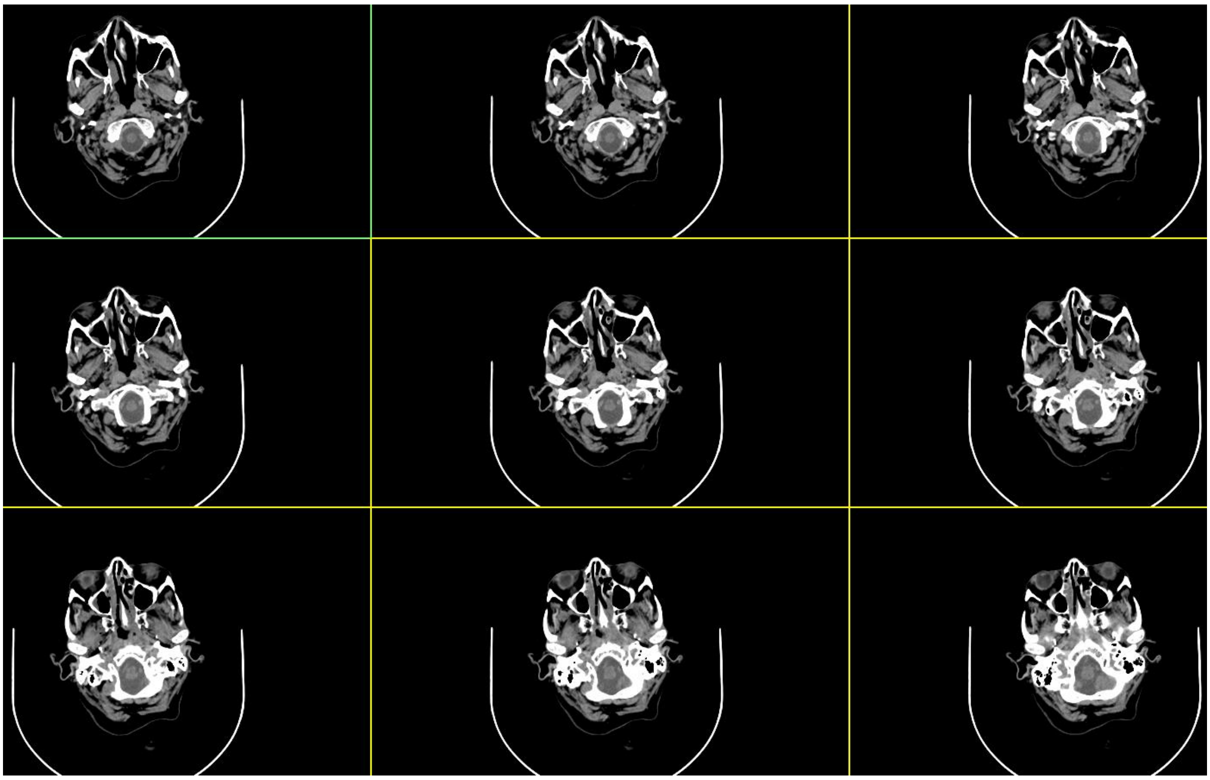

Figure C1 Brain Plain Computer Tomography Images, (3x3 display), Region of Interest is Globe

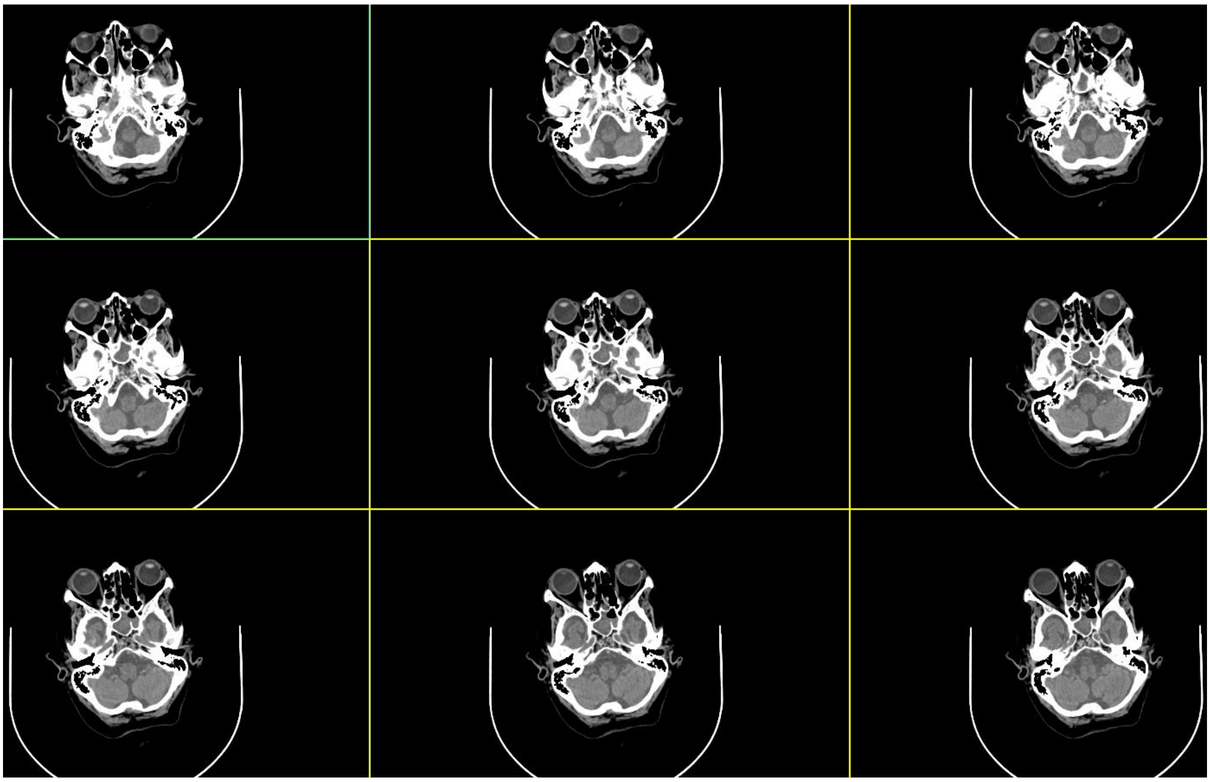

Figure C2 Brain Plain Computer Tomography Images, (3x3 display), Region of Interest is Globe and Lens

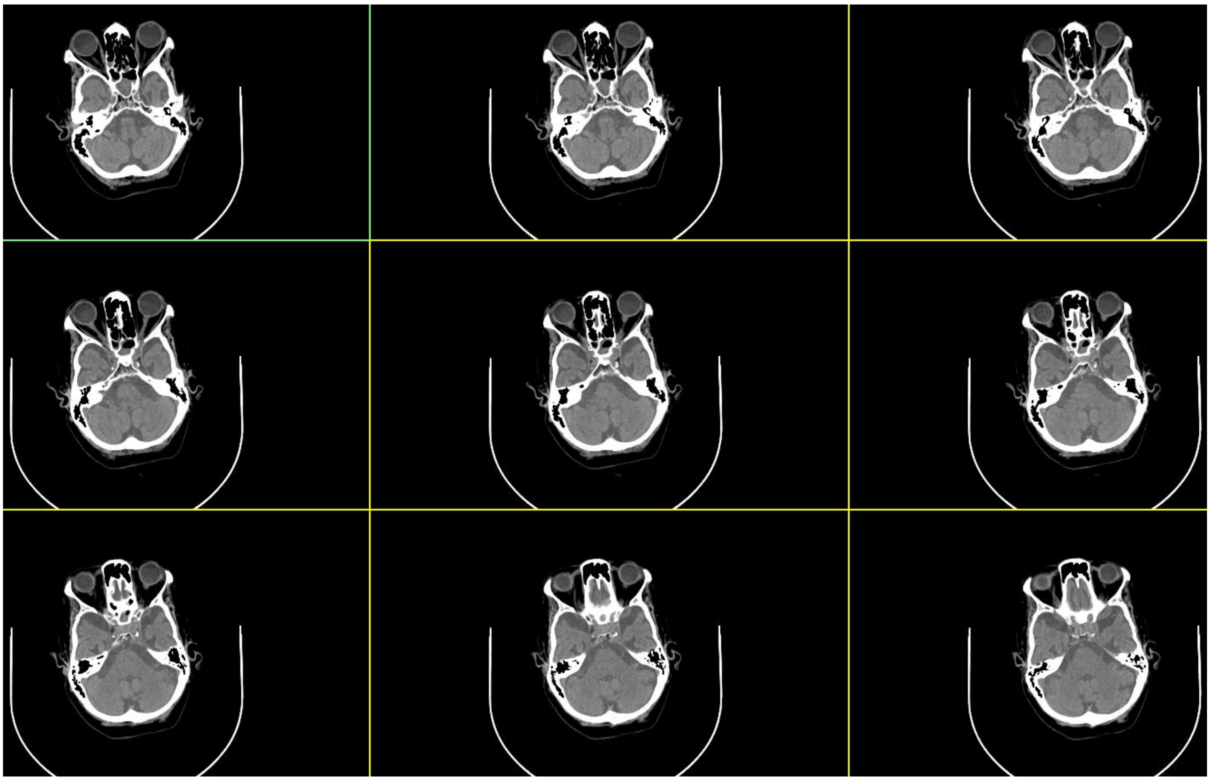

Figure C3 Brain Plain Computer Tomography Images, (3x3 display), Region of Interest is Globe and Lens

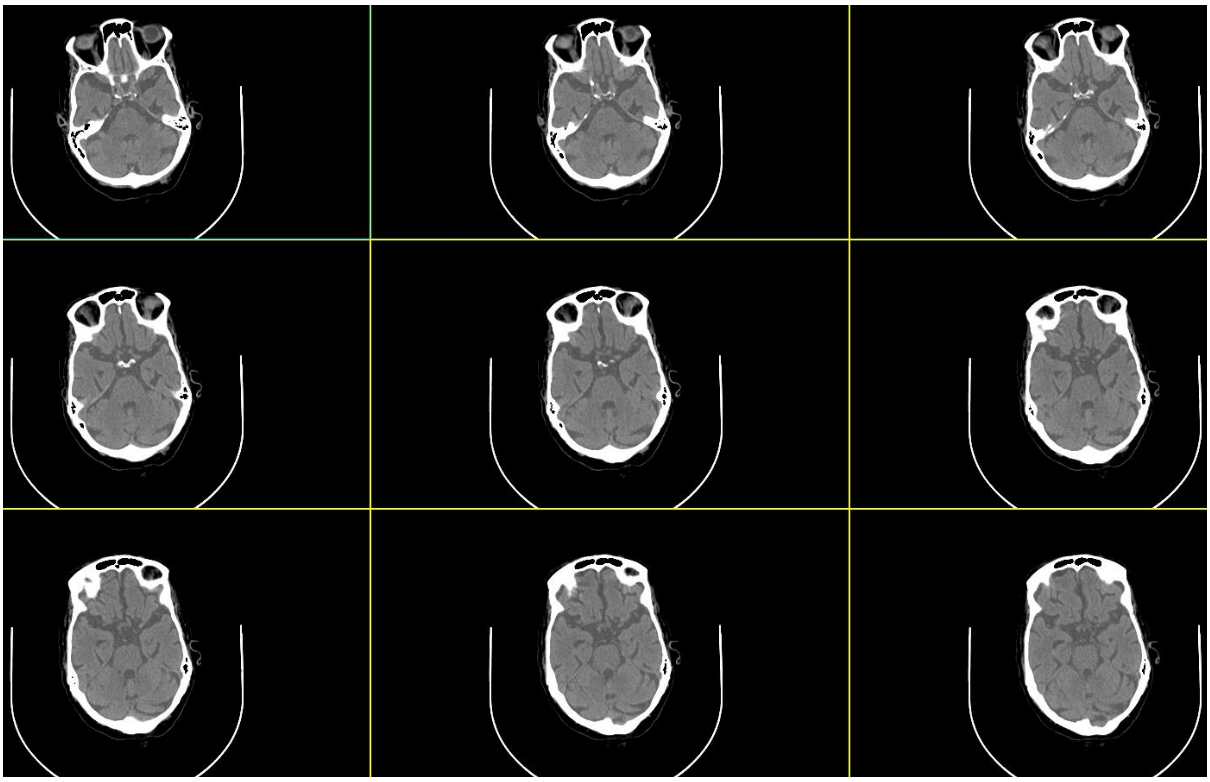

Figure C4 Brain Plain Computer Tomography Images, (3x3 display), Region of Interest is Globe, Lens and Ventricles

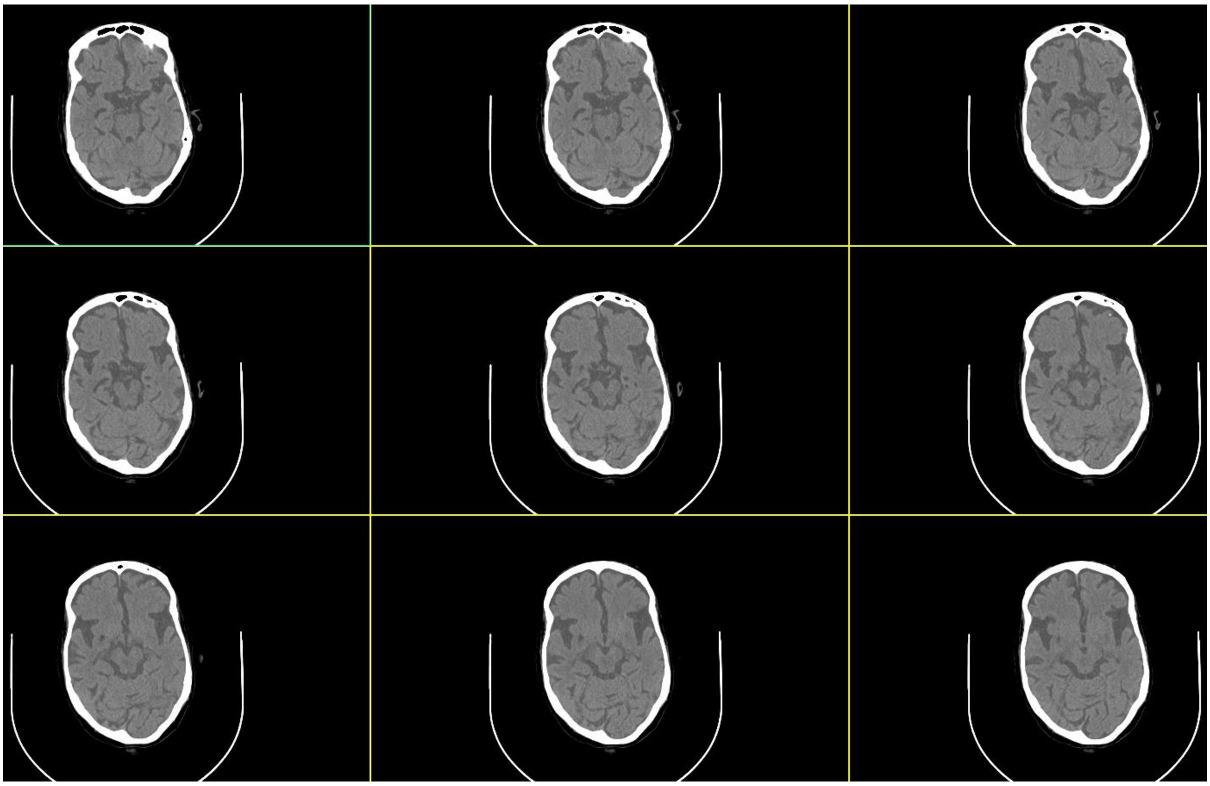

Figure C5 Brain Plain Computer Tomography Images, (3x3 display), Region of Interest is Ventricles

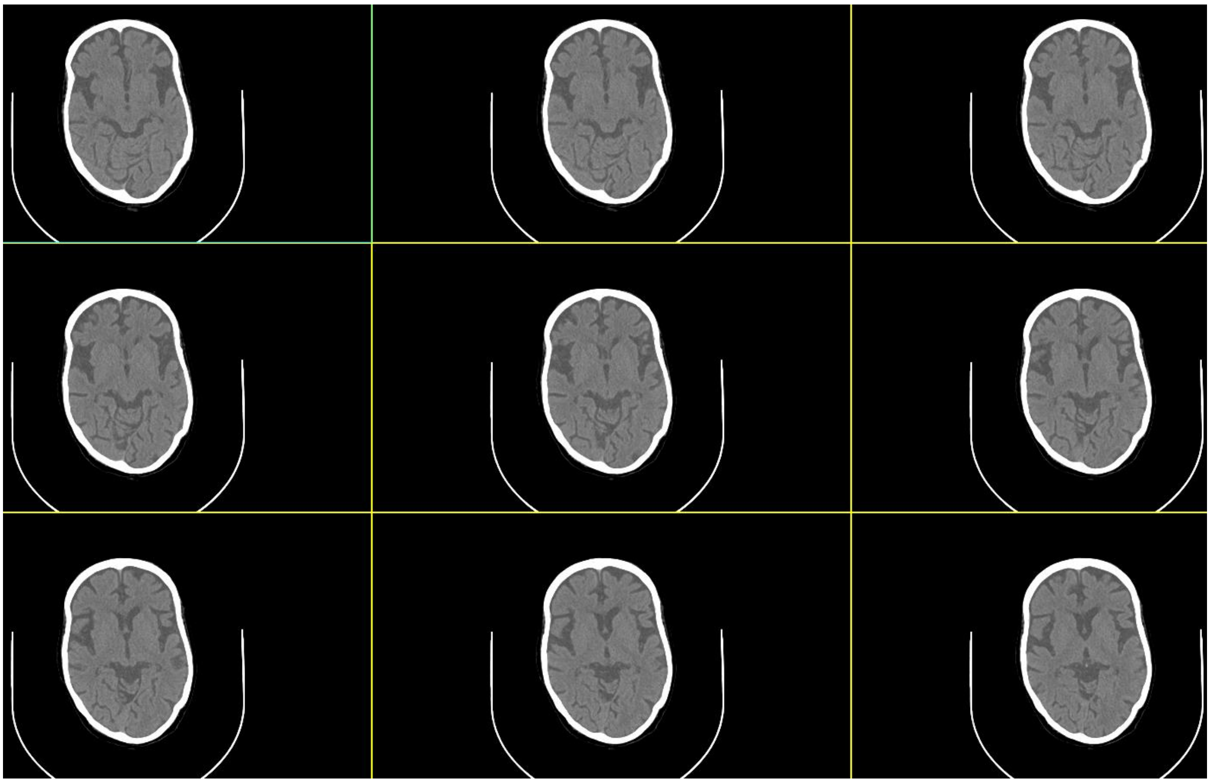

Figure C6 Brain Plain Computer Tomography Images, (3x3 display), Region of Interest is Ventricles

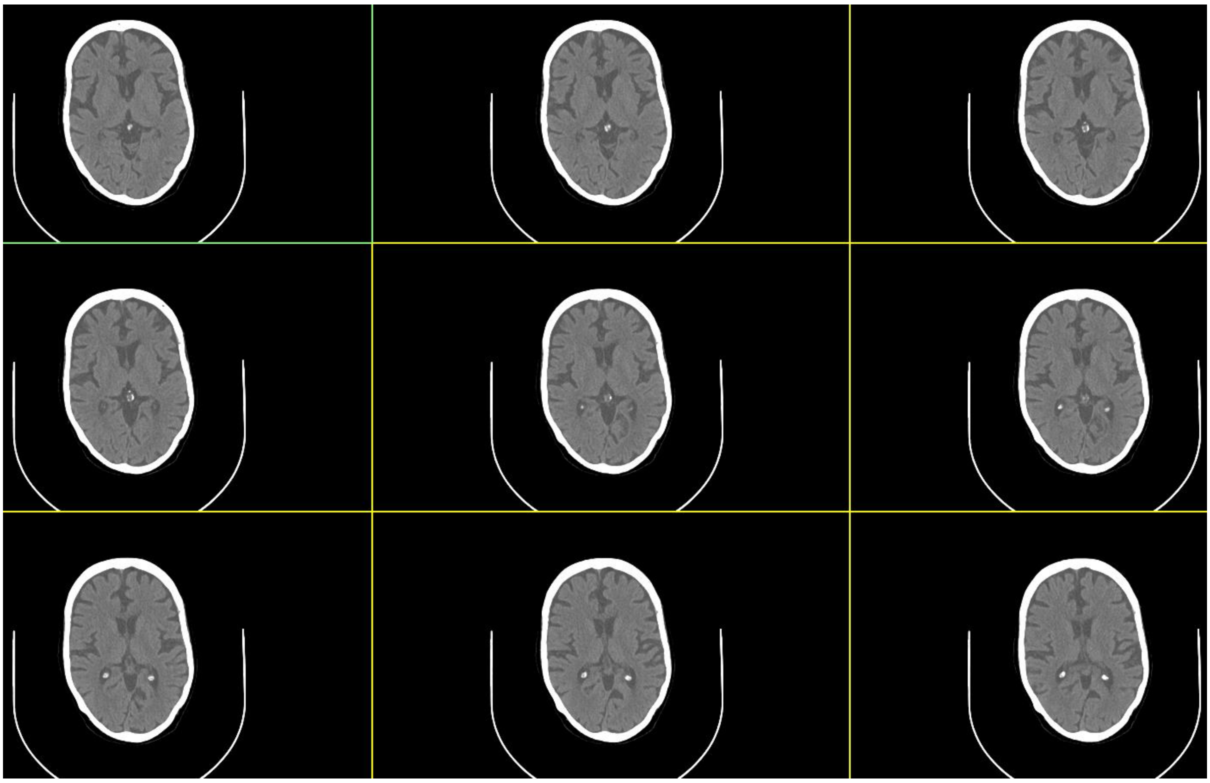

Figure C7 Brain Plain Computer Tomography Images, (3x3 display), Region of Interest is Brain tissues and Ventricles

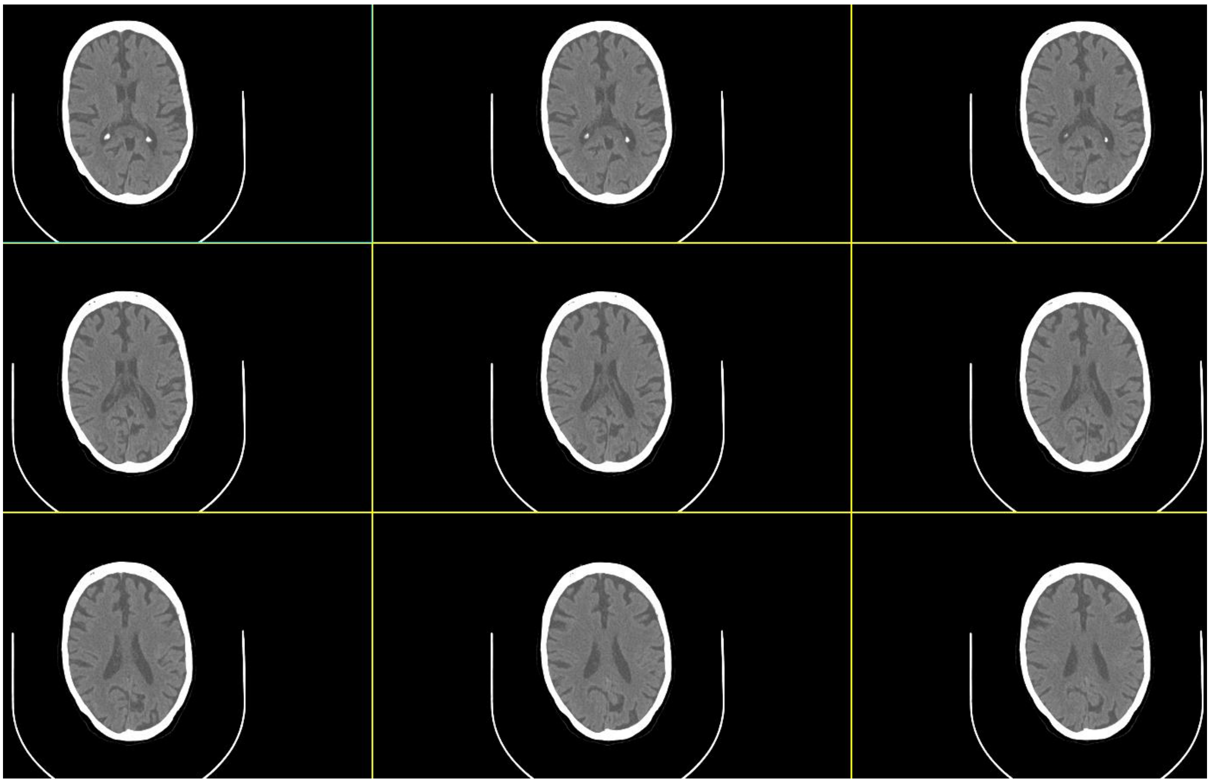

Figure C8 Brain Plain Computer Tomography Images, (3x3 display), Region of Interest is Arteries and Ventricles

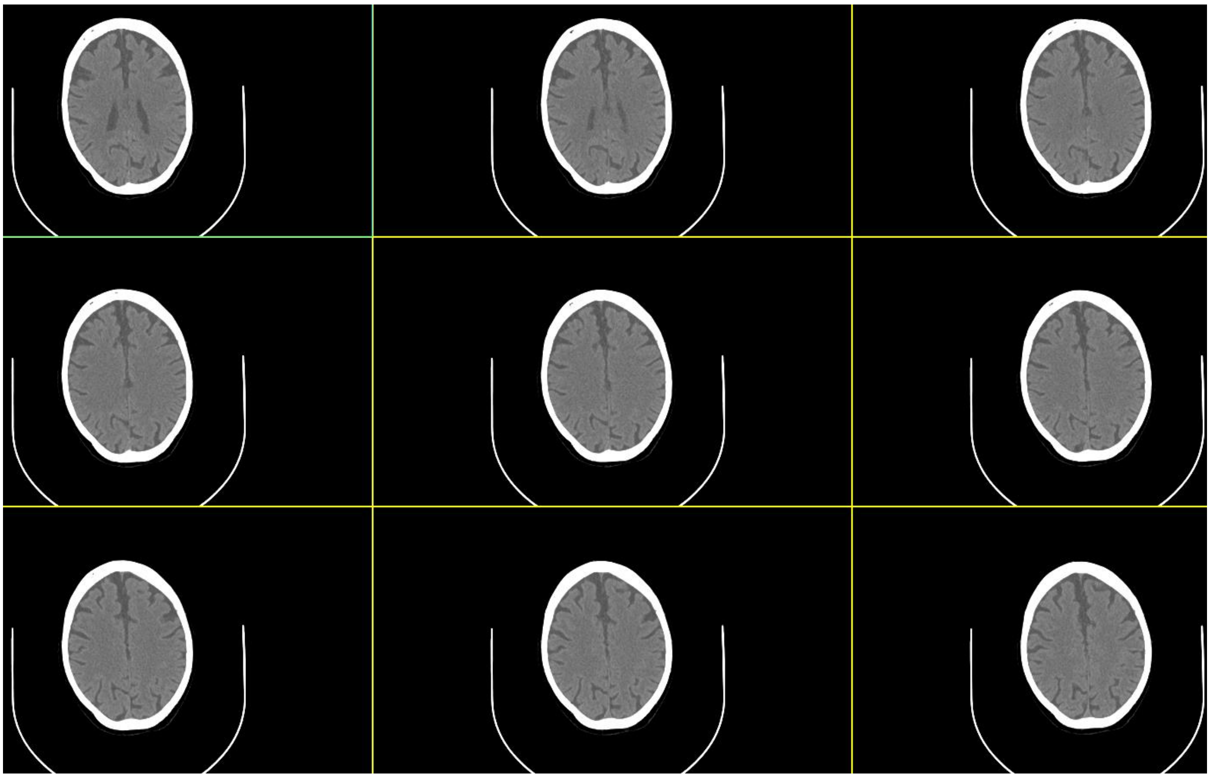

Figure C9 Brain Plain Computer Tomography Images, (3x3 display), Region of Interest is Brain tissues and Ventricles

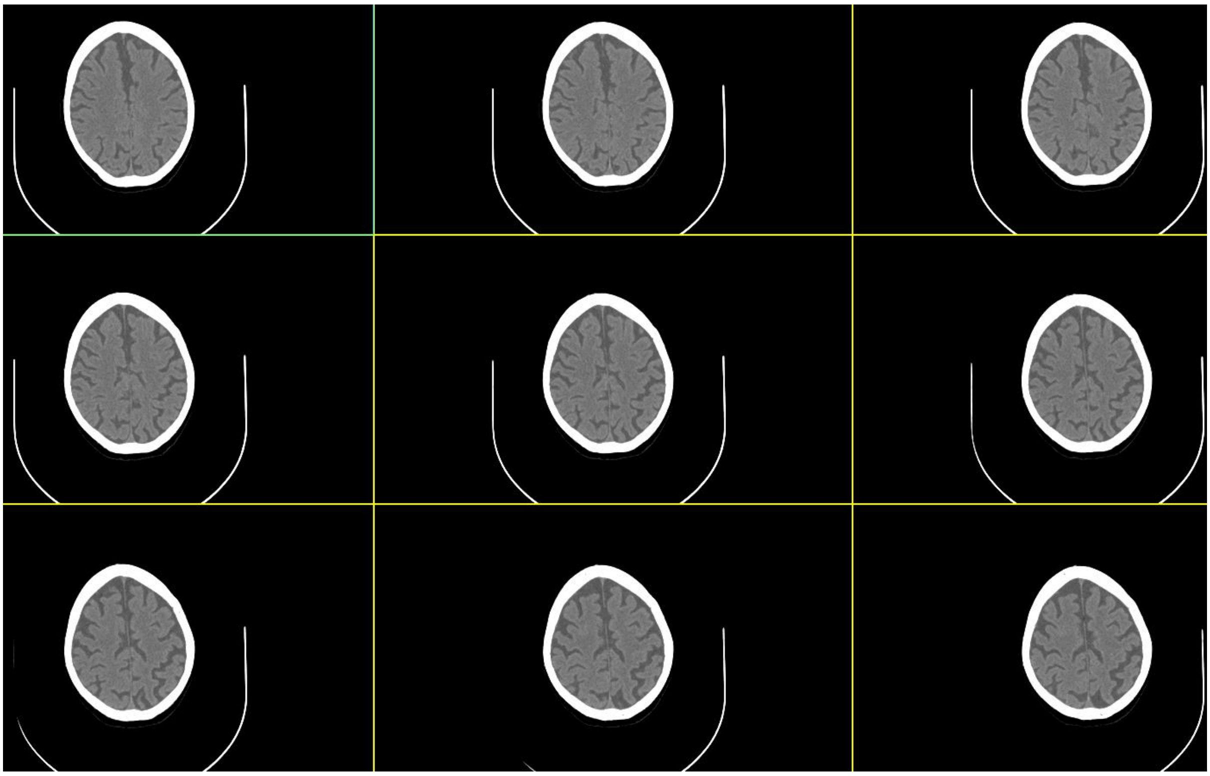

Figure C10 Brain Plain Computer Tomography Images, (3x3 display), Region of Interest is Brain tissues and Ventricles

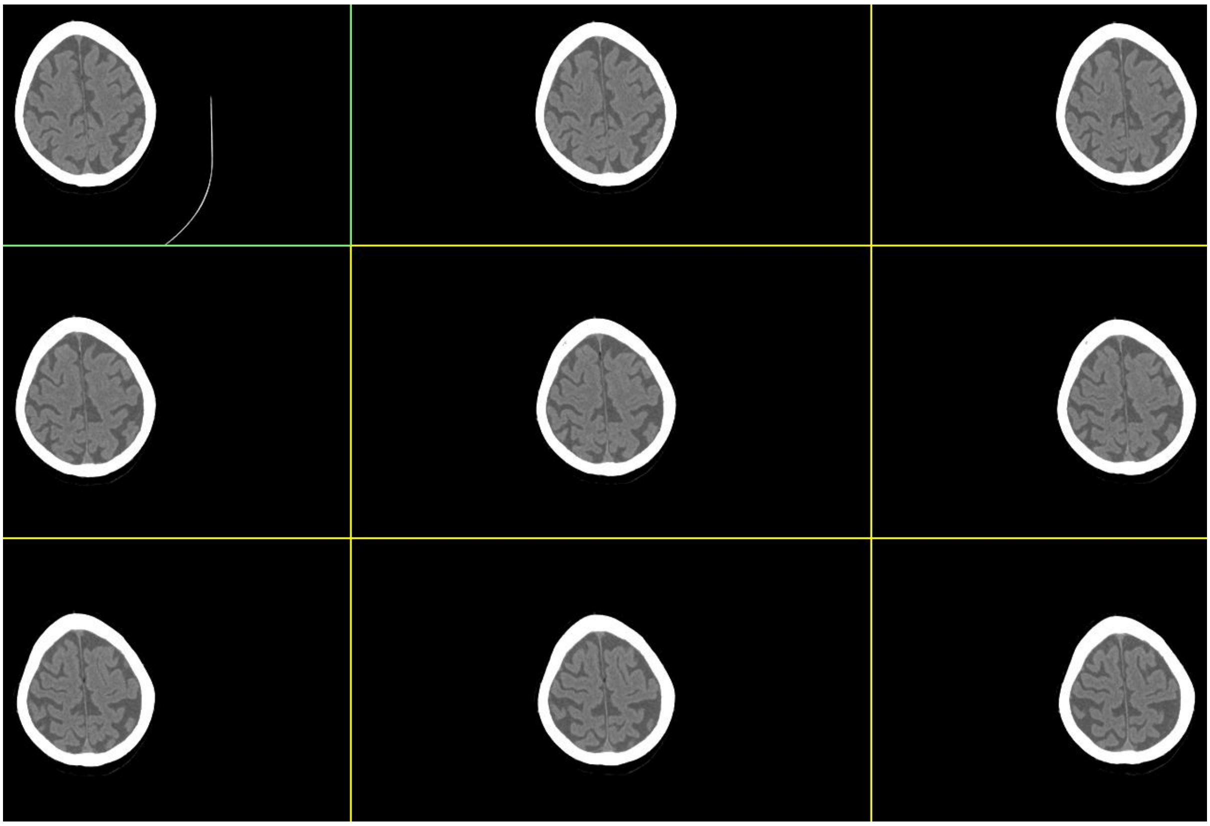

Figure C11 Brain Plain Computer Tomography Images, (3x3 display), Region of Interest is Brain tissues and Ventricles

All the above figures of Thoracic cavity (figure C1-C11), are Brain Plain CT images acquired at improved low contrast resolution for better image impression

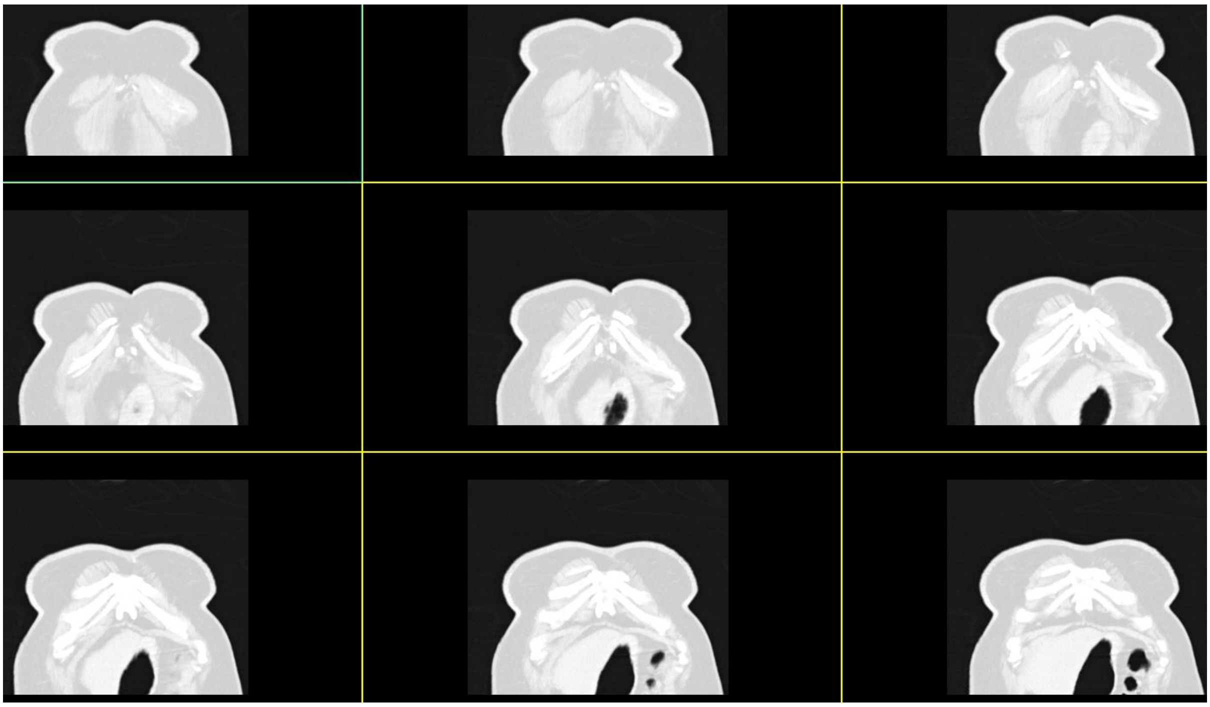

Figure D1 High Resolution Computer Tomography Thorax region, CORONAL Plane of 3 mm thickness in (3x3 display) mode

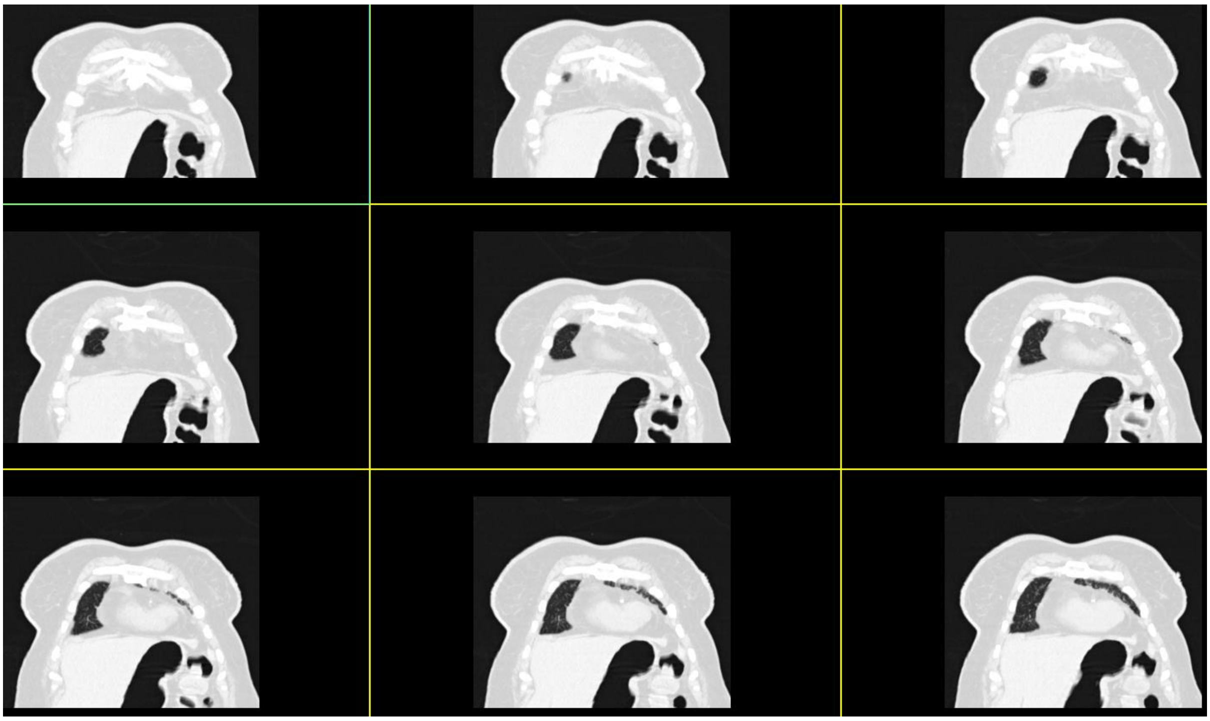

Figure D2 High Resolution Computer Tomography Thorax region, CORONAL Plane of 3 mm thickness in (3x3 display) mode

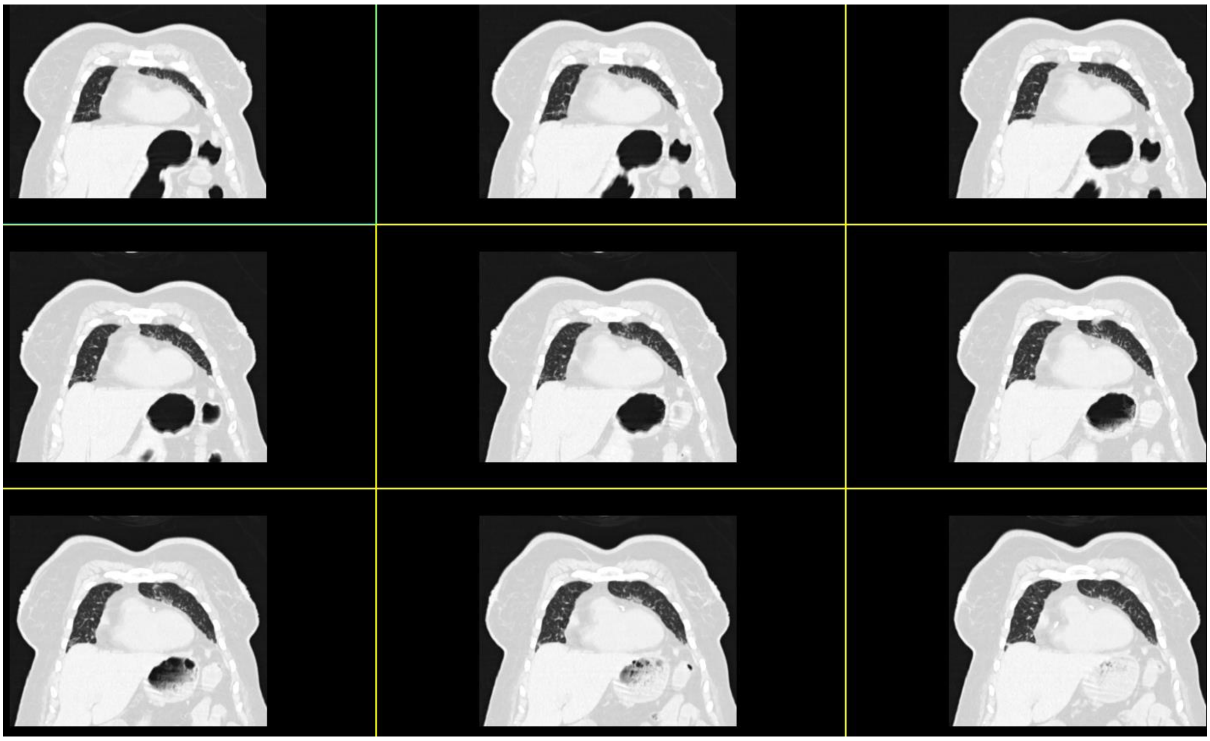

Figure D3 High Resolution Computer Tomography Thoracic Cavity, CORONAL Plane of 3 mm thickness, in (3x3 display) mode, Region of Interest is Lungs

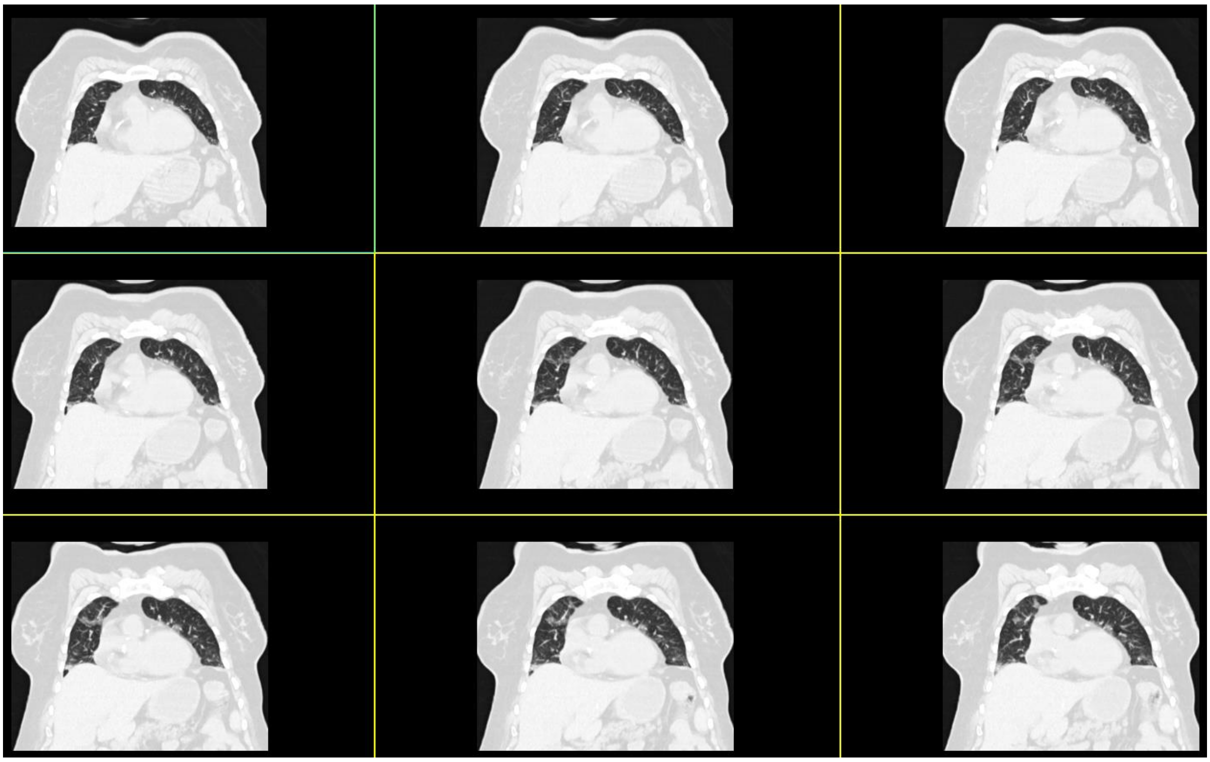

Figure D4 High Resolution Computer Tomography Thoracic Cavity, CORONAL Plane of 3 mm thickness, in (3x3 display) mode, Region of Interest is Lungs

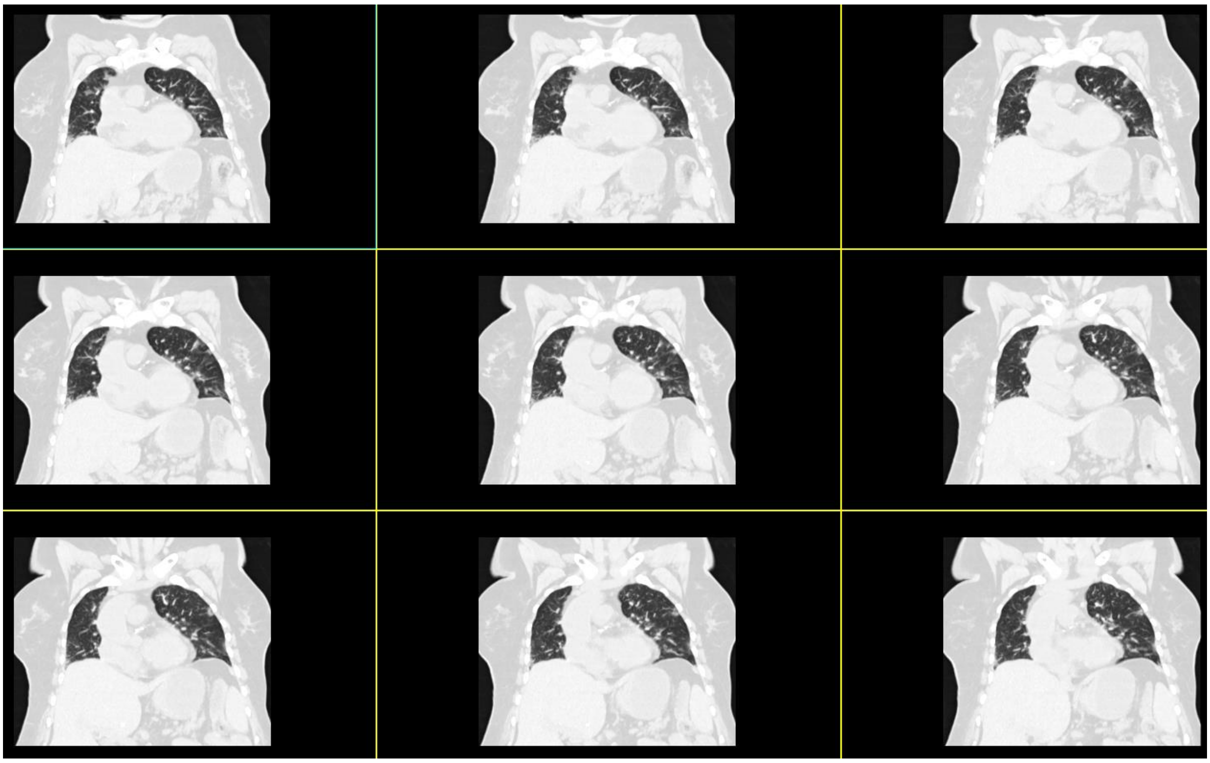

Figure D5 High Resolution Computer Tomography Thoracic Cavity, CORONAL Plane of 3 mm thickness, in (3x3 display) mode, Region of Interest is Lungs

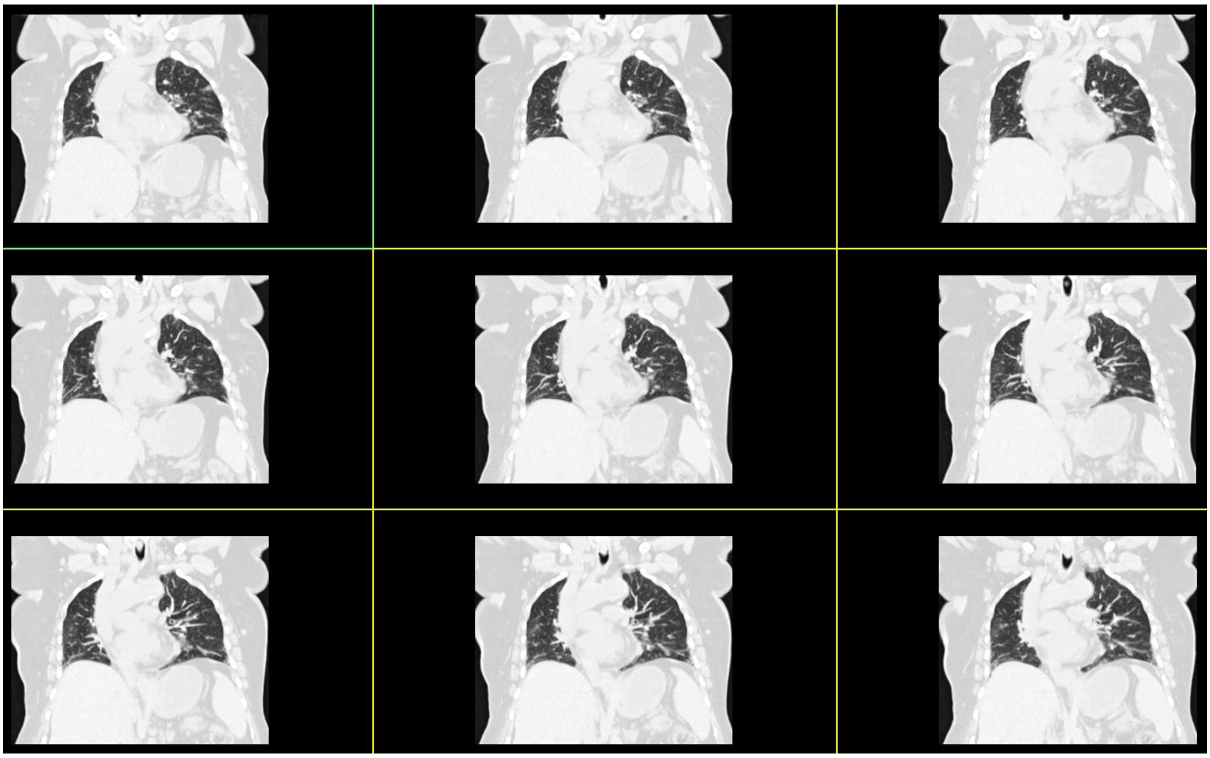

Figure D6 High Resolution Computer Tomography Thoracic Cavity, CORONAL Plane of 3 mm thickness, in (3x3 display) mode, Region of Interest is Lungs

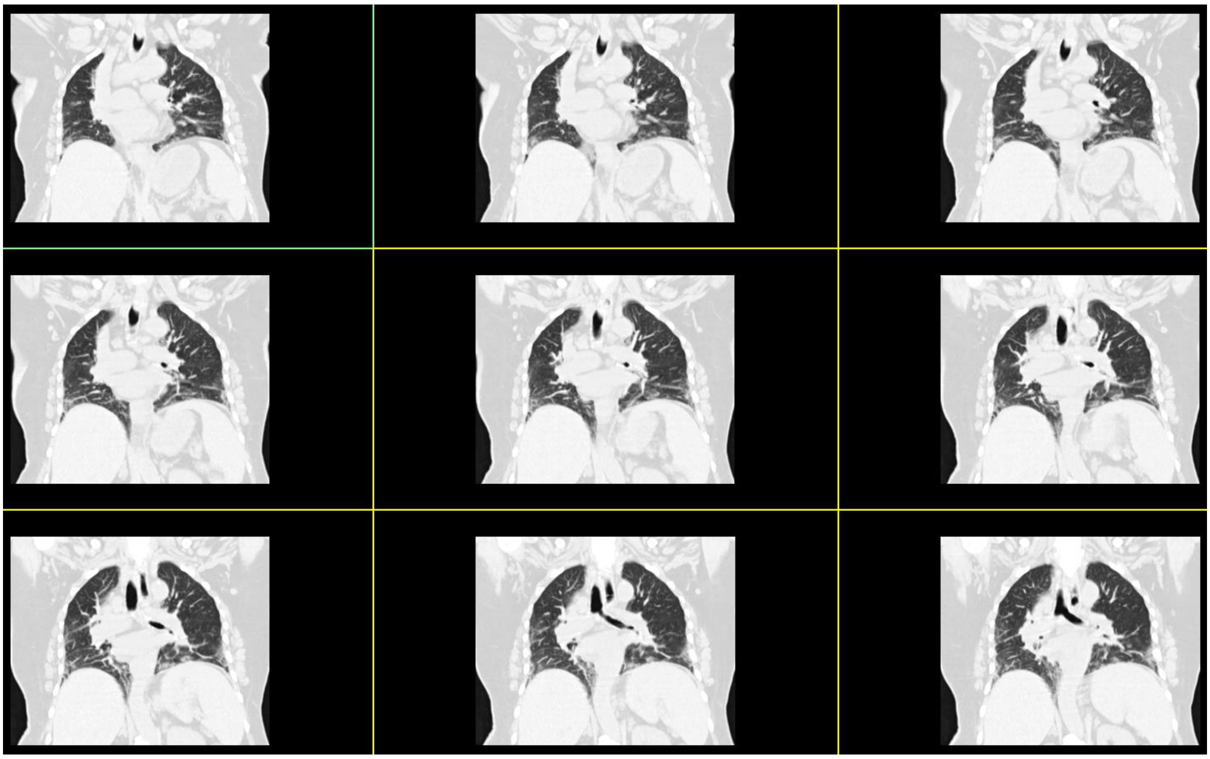

Figure D7 High Resolution Computer Tomography Thoracic Cavity, CORONAL Plane of 3 mm thickness, in (3x3 display) mode, Region of Interest is Lungs

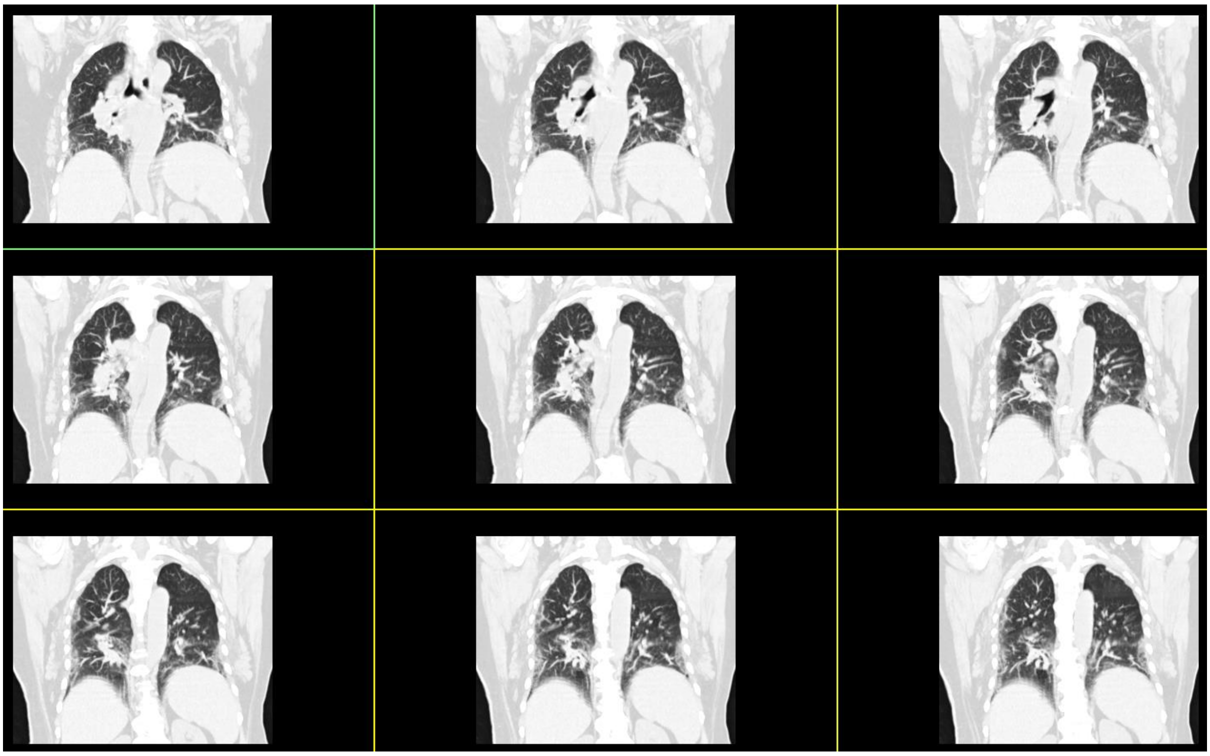

Figure D8 High Resolution Computer Tomography Thoracic Cavity, CORONAL Plane of 3 mm thickness, in (3x3 display) mode, Region of Interest is Lungs

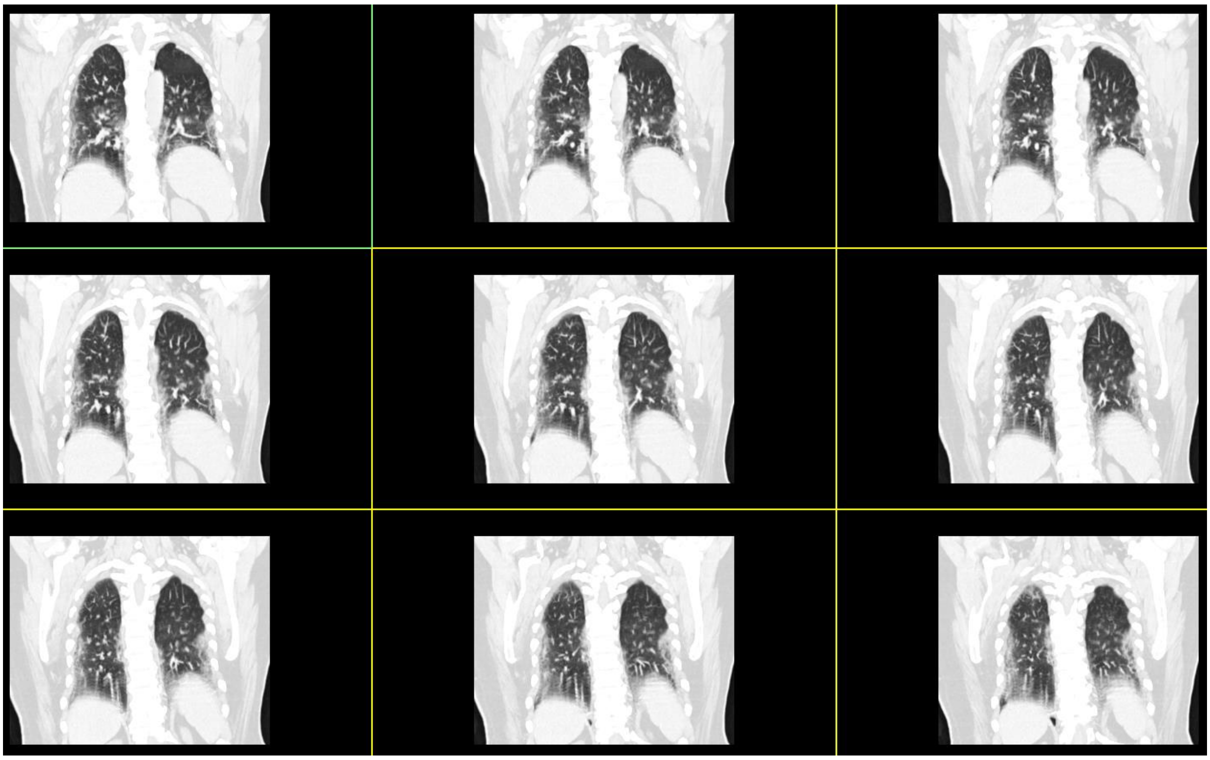

Figure D9 High Resolution Computer Tomography Thoracic Cavity, CORONAL Plane of 3 mm thickness, in (3x3 display) mode, Region of Interest is Lungs

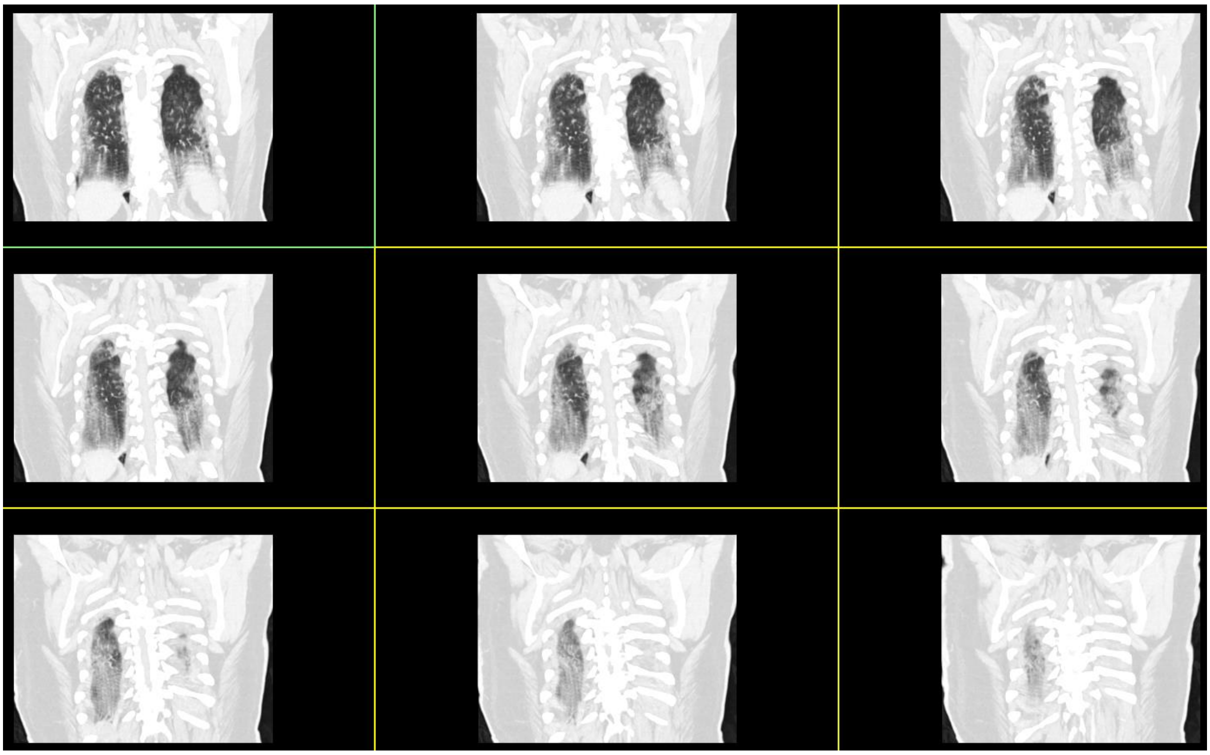

Figure D10 High Resolution Computer Tomography Thoracic Cavity, CORONAL Plane of 3 mm thickness, in (3x3 display) mode, Region of Interest is Lungs and rib cage

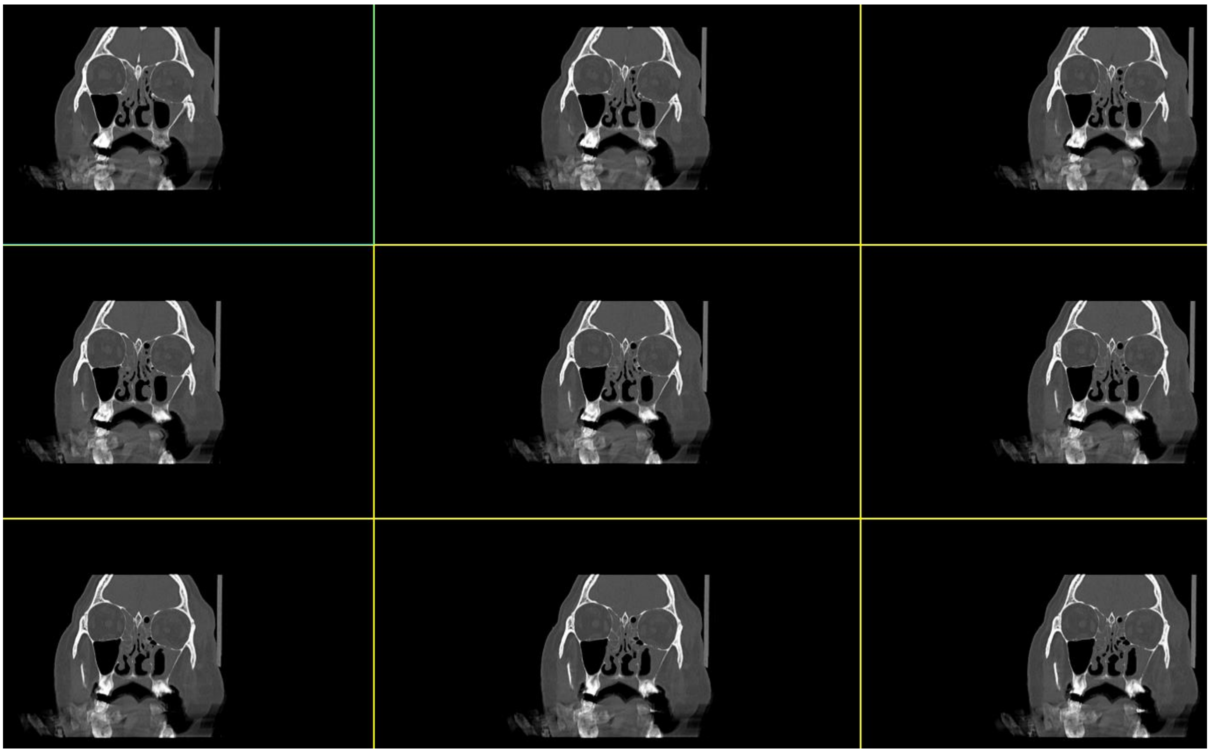

Figure E1 Computer Tomography Multiplanar Reconstructed Images of 0.500 mm thickness in (3x3 display) mode

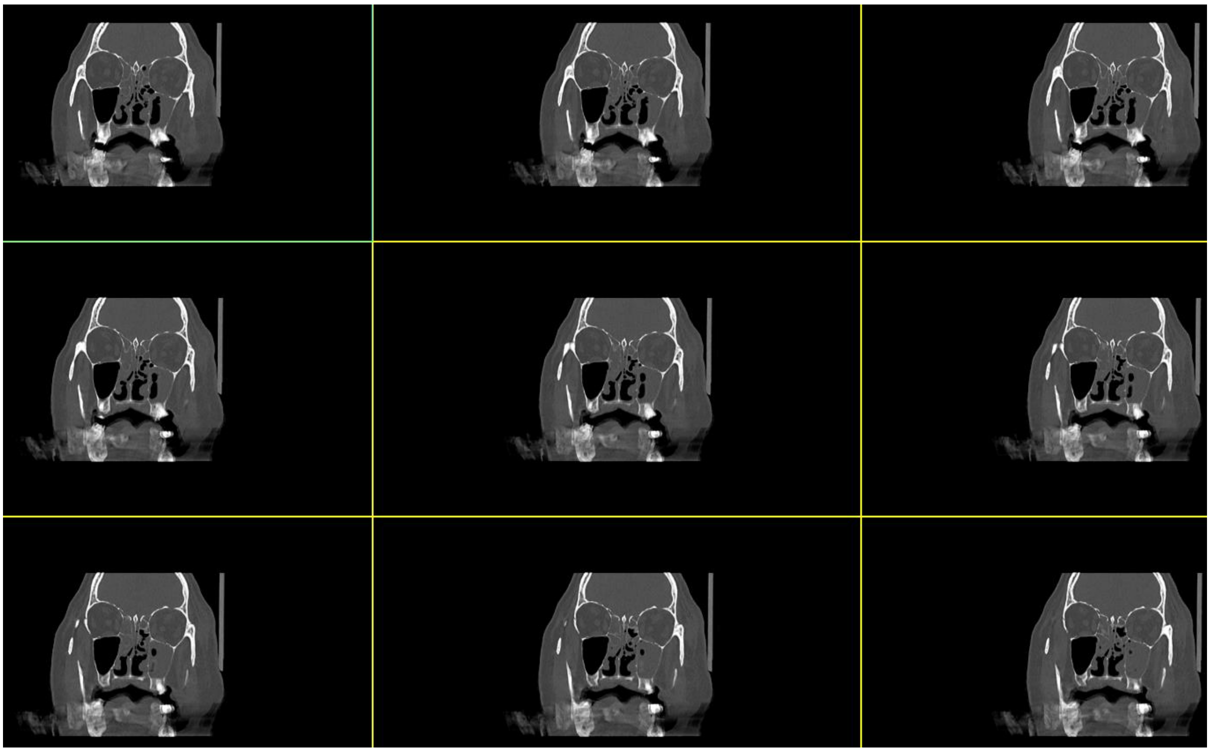

Figure E2 Computer Tomography Multiplanar Reconstructed Images of 0.500 mm thickness in (3x3 display) mode

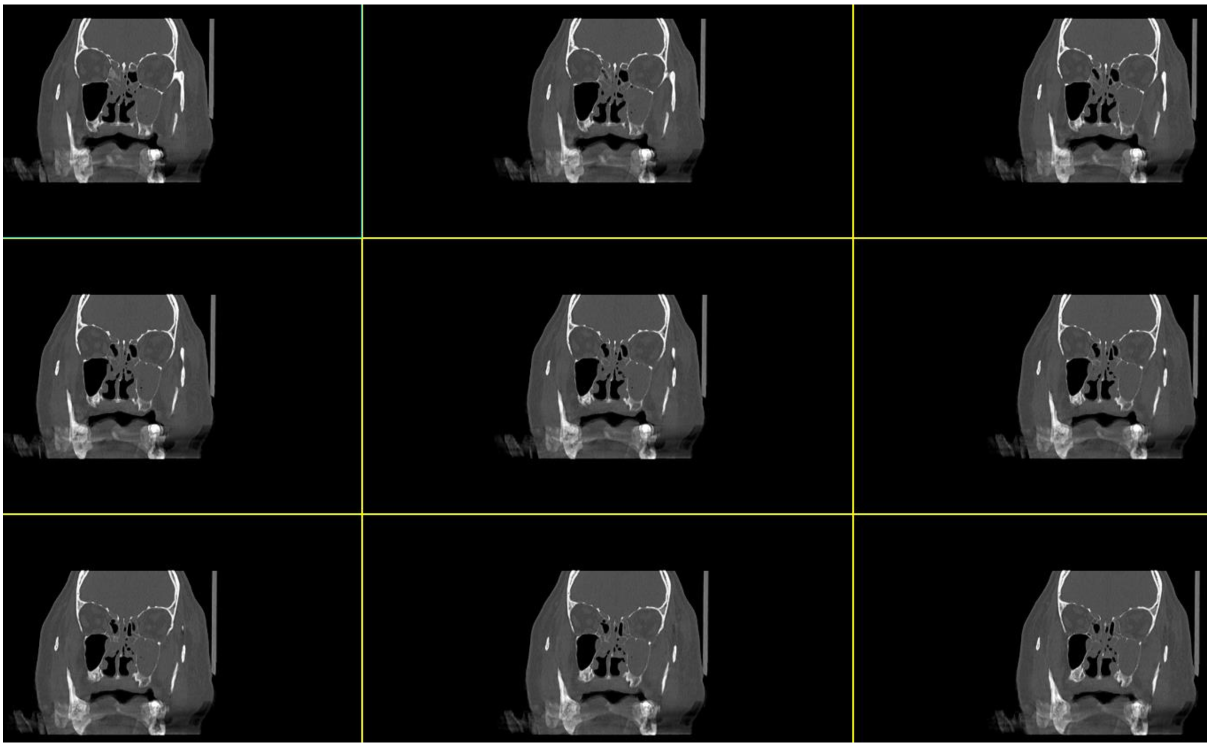

Figure E3 Computer Tomography Multiplanar Reconstructed Images of 0.500 mm thickness in (3x3 display) mode

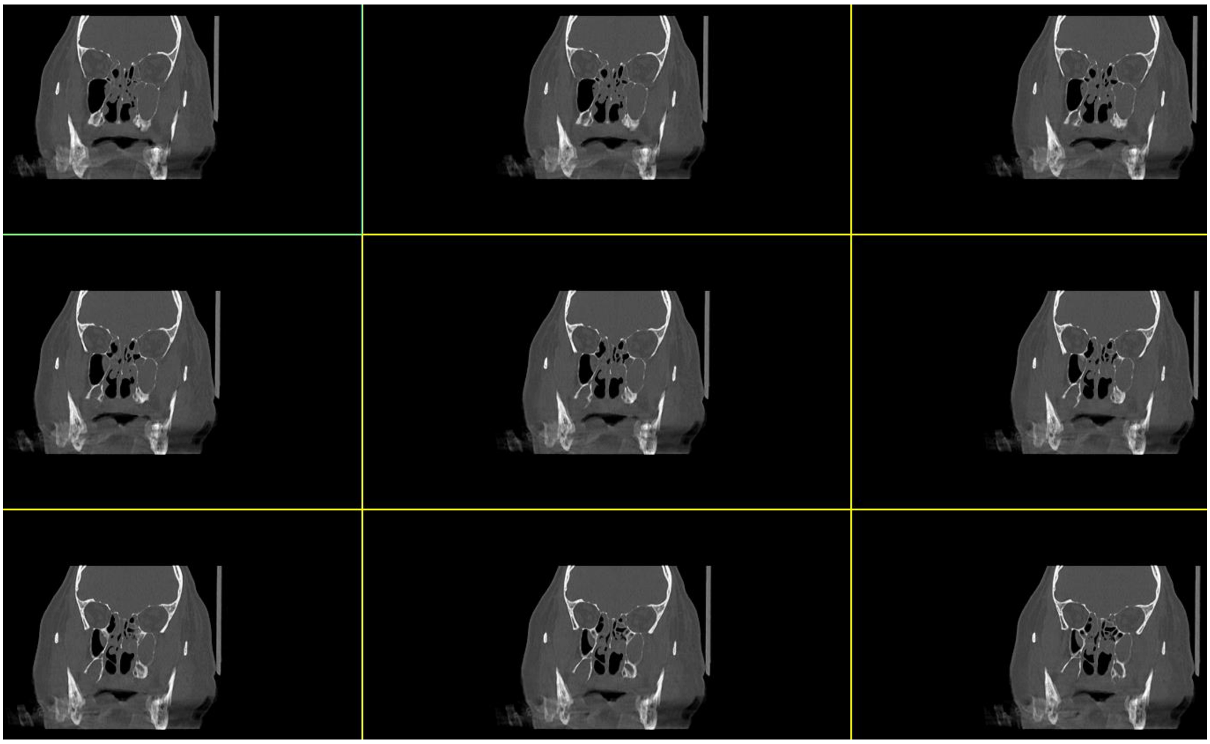

Figure E4 Computer Tomography Multiplanar Reconstructed Images of 0.500 mm thickness in (3x3 display) mode

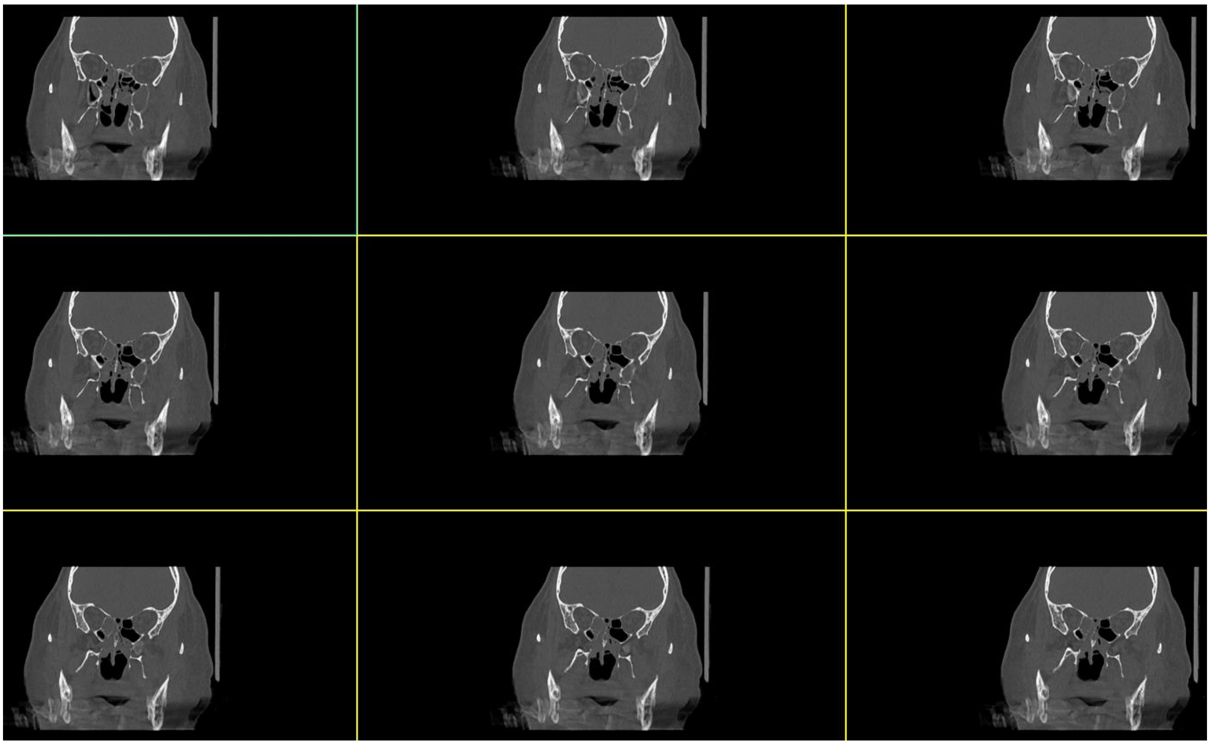

Figure E5 Computer Tomography Multiplanar Reconstructed Images of 0.500 mm thickness in (3x3 display) mode

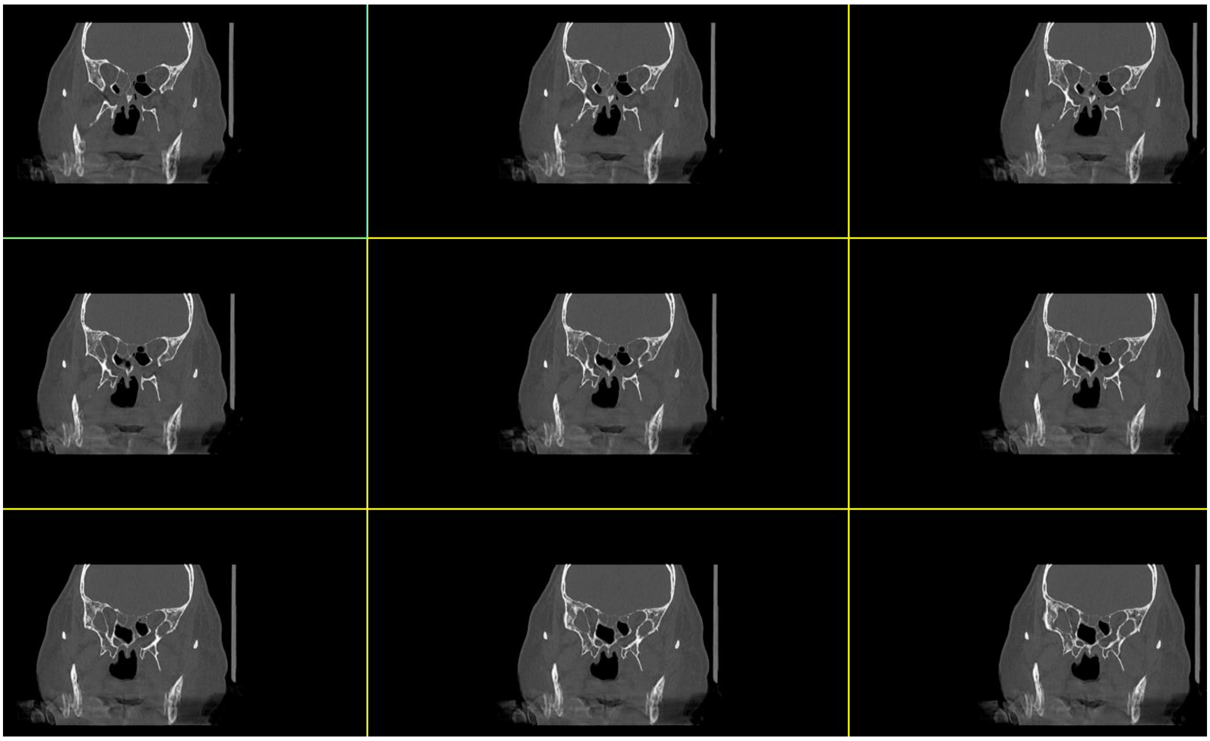

Figure E6 Computer Tomography Multiplanar Reconstructed Images of 0.500 mm thickness in (3x3 display) mode

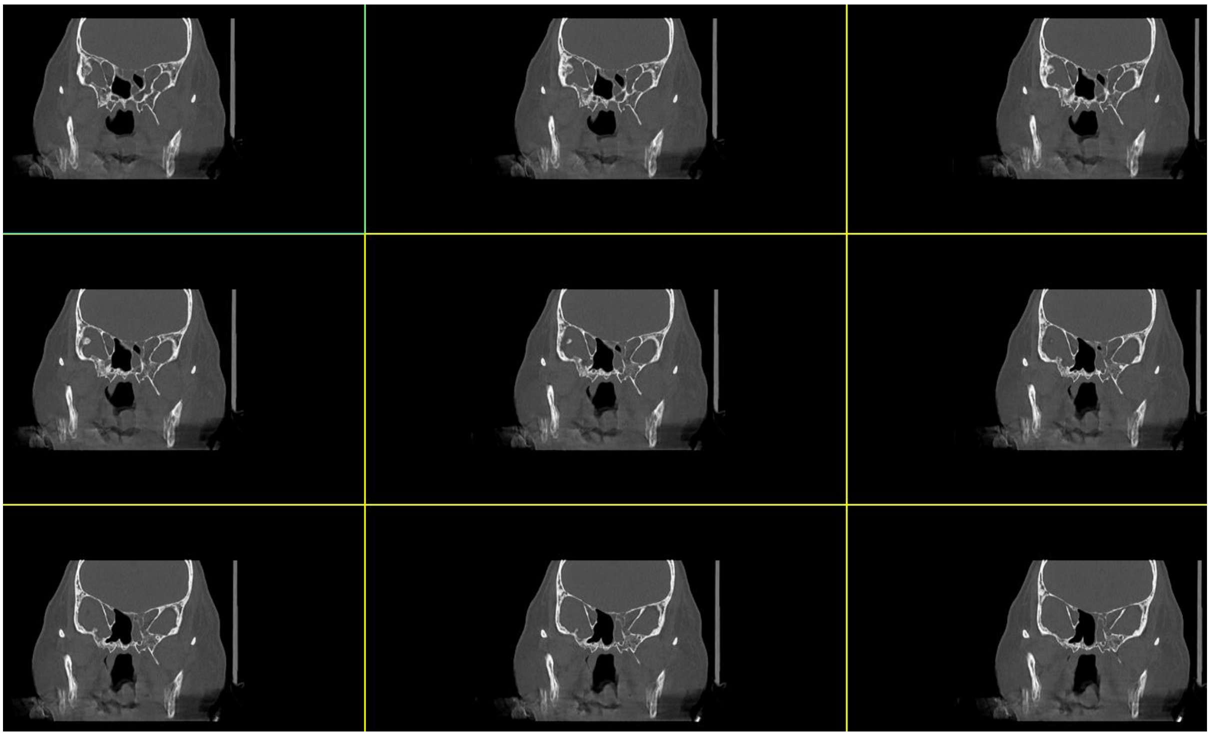

Figure E7 Computer Tomography Multiplanar Reconstructed Images of 0.500 mm thickness in (3x3 display) mode

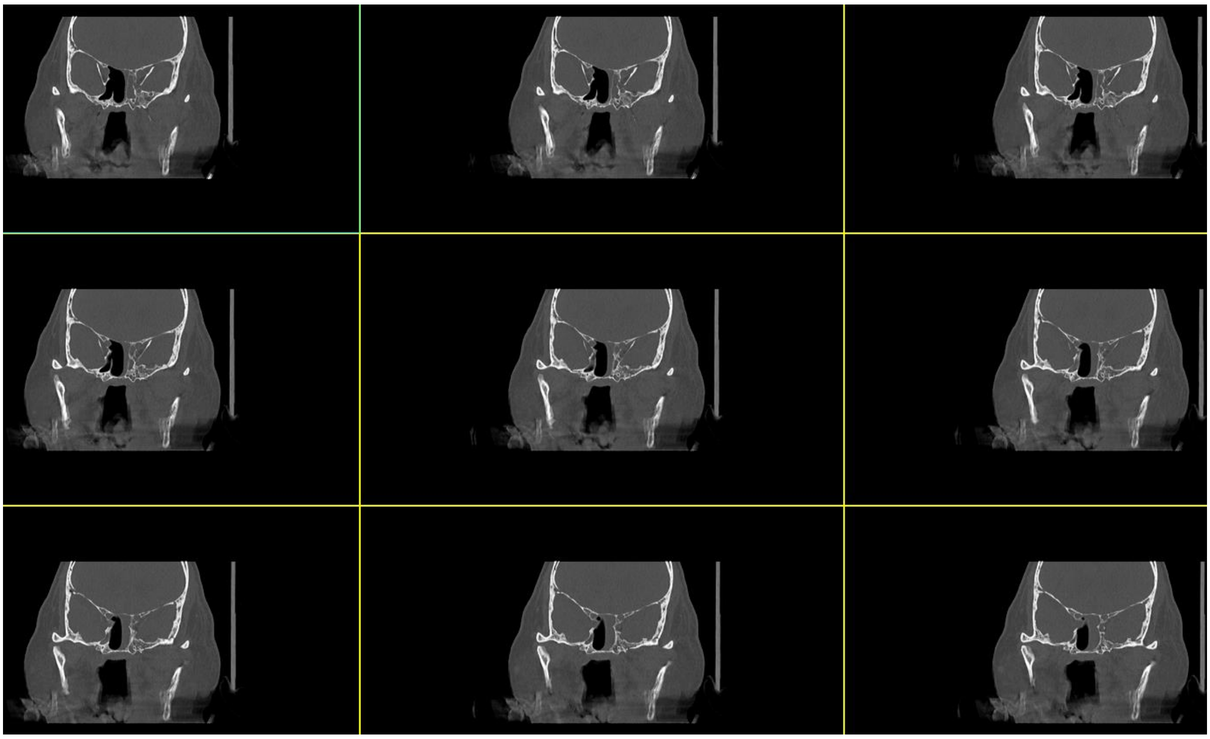

Figure E8 Computer Tomography Multiplanar Reconstructed Images of 0.500 mm thickness in (3x3 display) mode

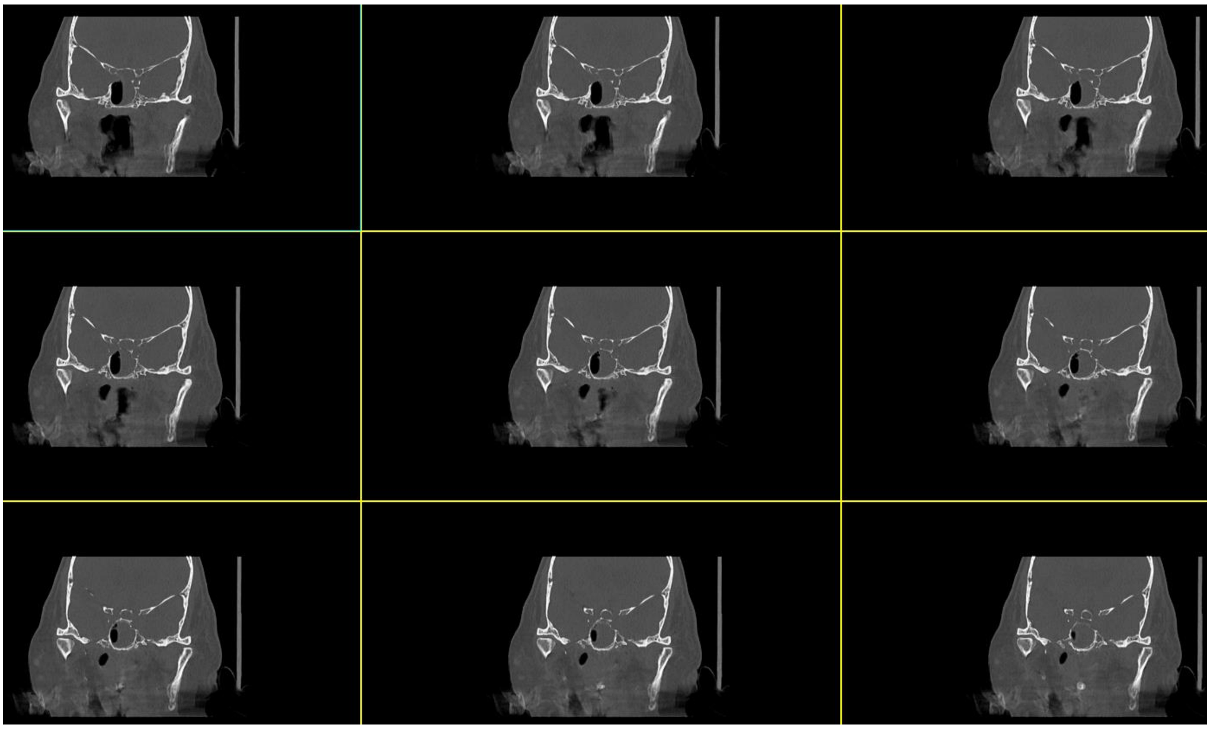

Figure E9 Computer Tomography Multiplanar Reconstructed Images of 0.500 mm thickness in (3x3 display) mode

All the above figure (E1-E9), are obtained at a tilt angle of 0.0 degrees

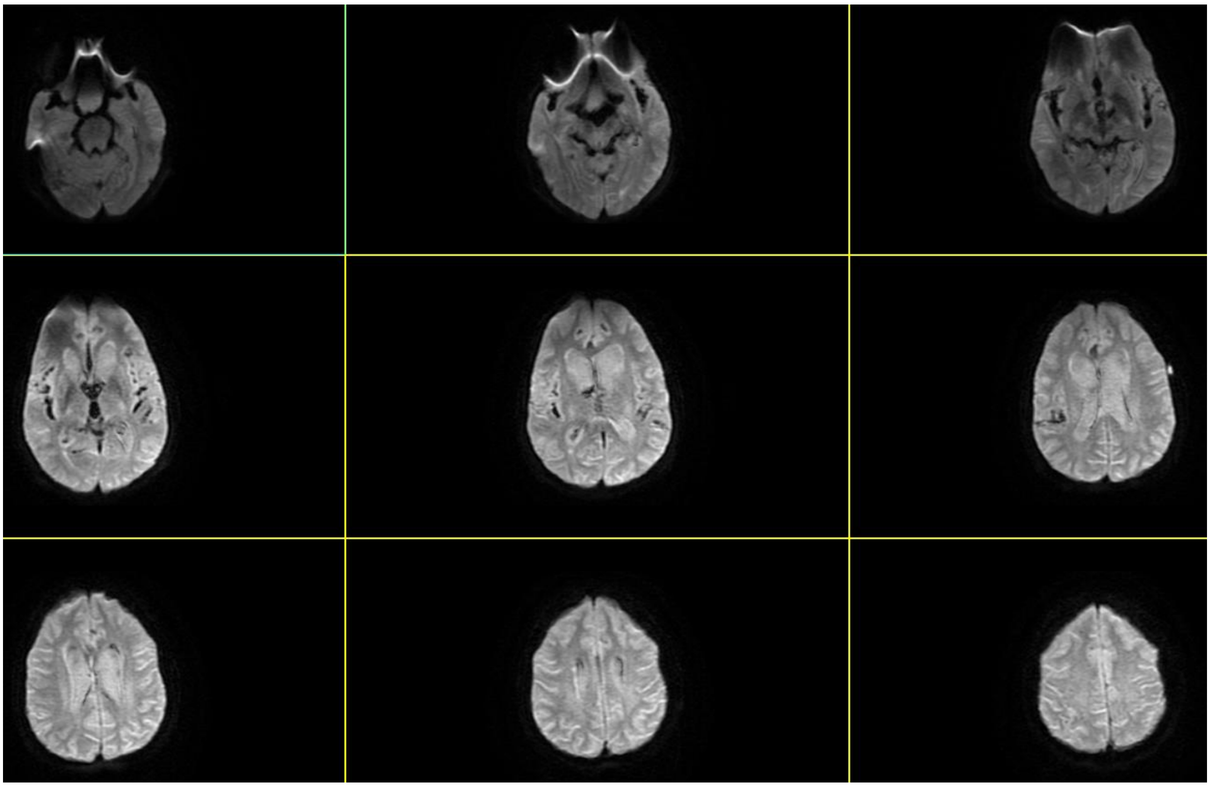

Figure F1 Diffusion Weighed Magnetic Resonance Images of Brain in (3x3 display), Region of Interest is tissues of Brain

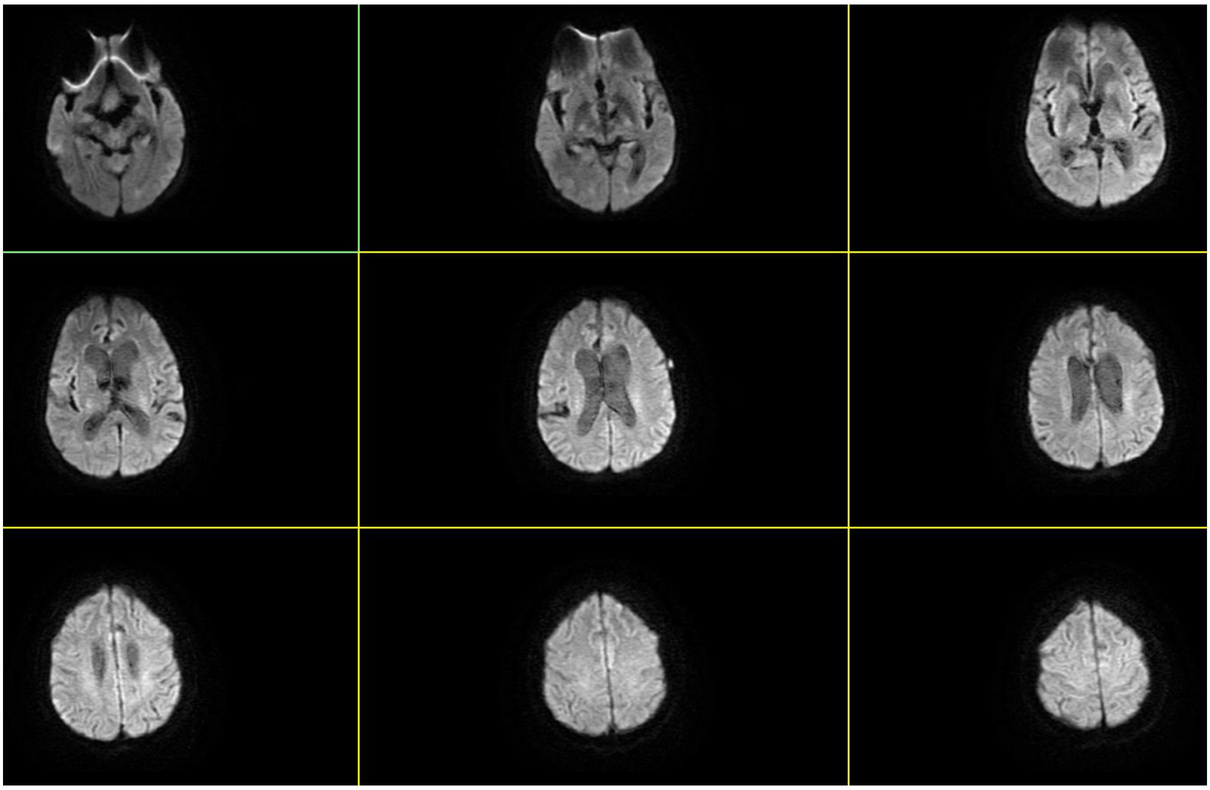

Figure F2 Diffusion Weighed Magnetic Resonance Images of Brain in (3x3 display), Region of Interest is tissues of Brain and arteries of Brain

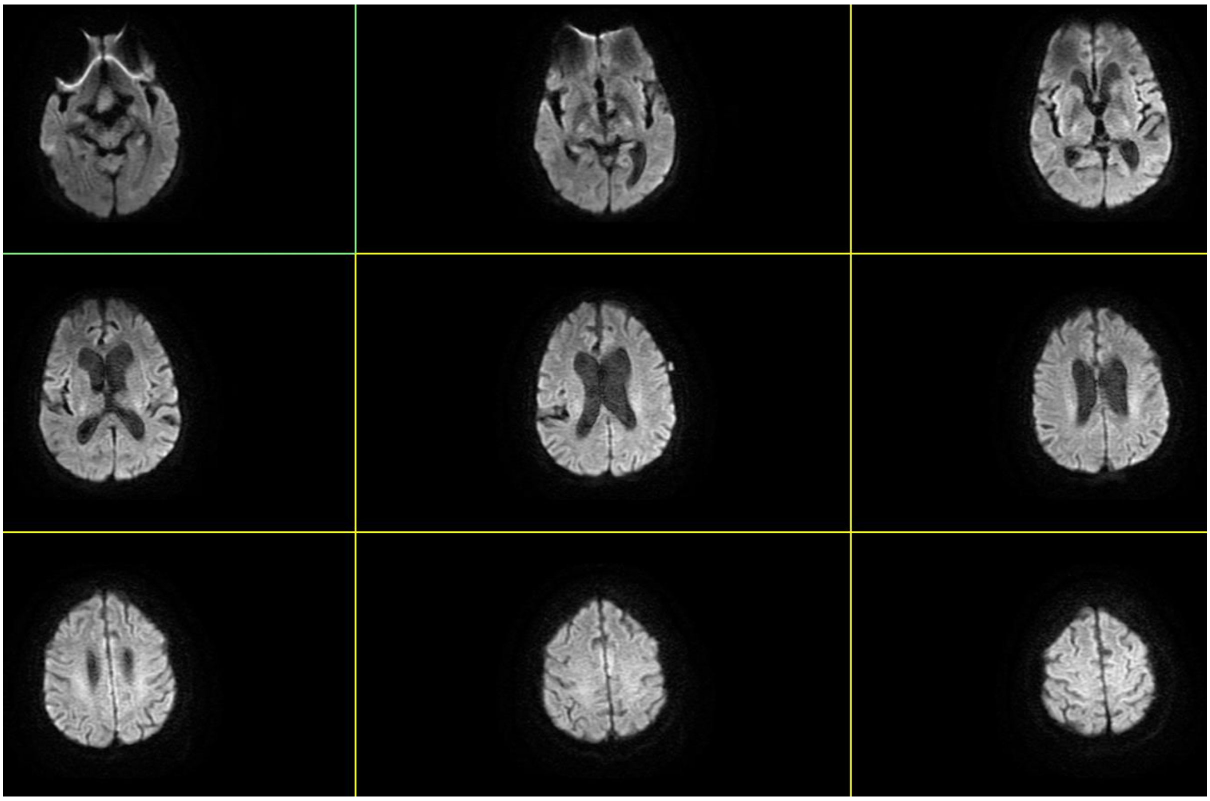

Figure F3 Diffusion Weighed Magnetic Resonance Images of Brain in (3x3 display), Region of Interest is Middle Cerebral Artery and tissues of Brain

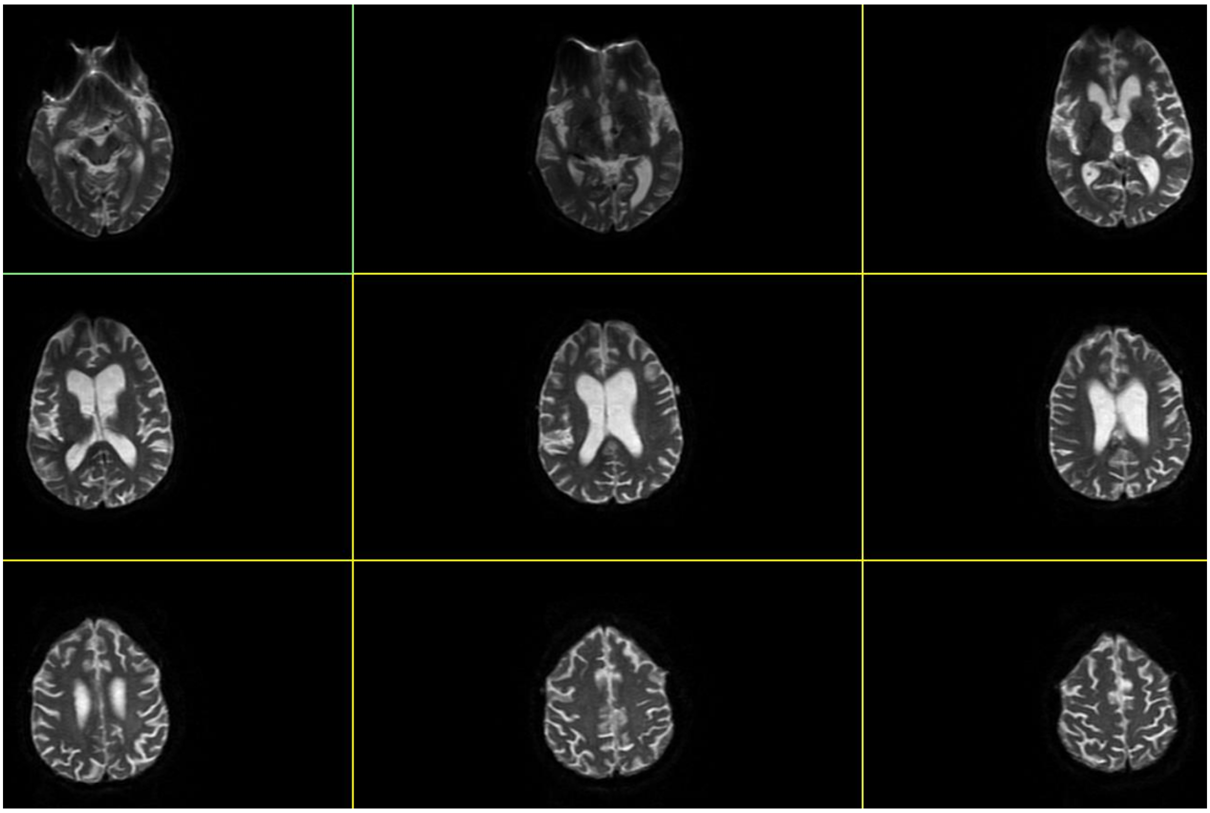

Figure F4 Diffusion Weighed Magnetic Resonance Images of Brain in (3x3 display), Region of Interest is Middle Cerebral Artery and tissues of Brain

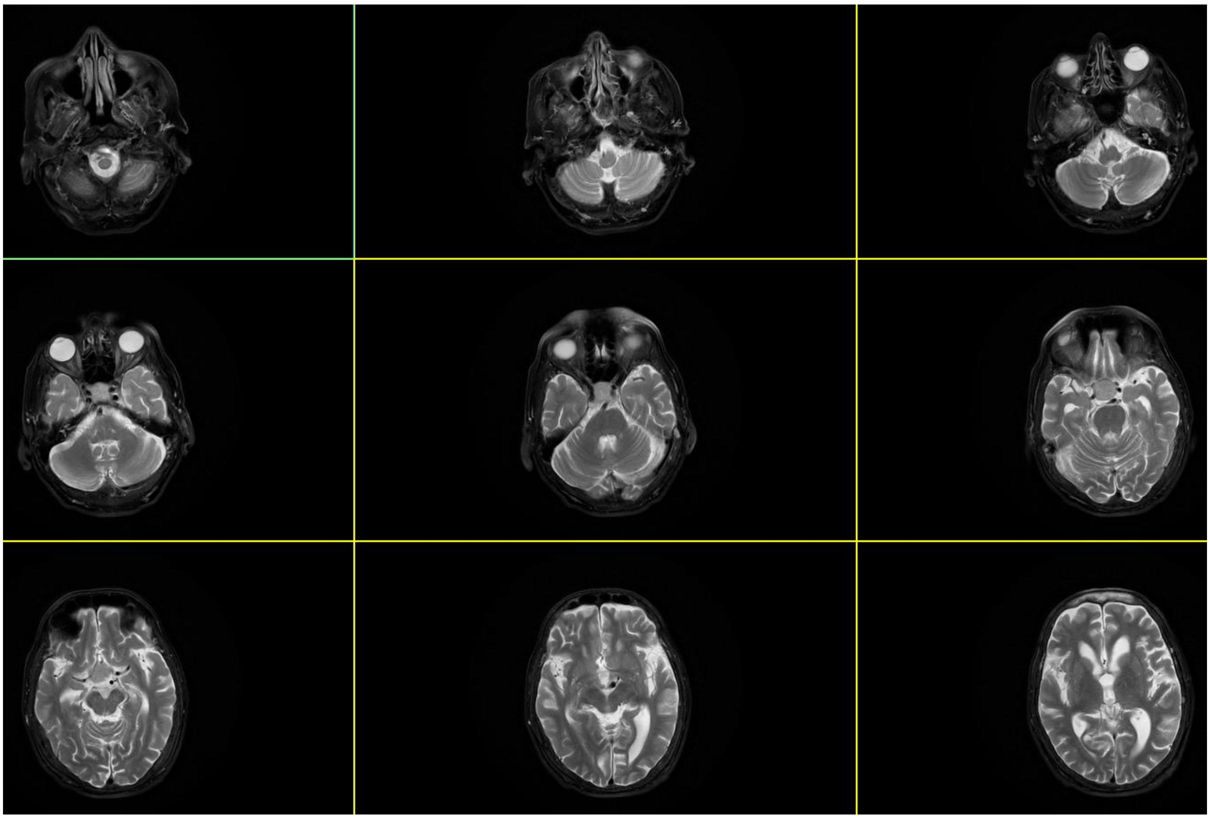

Figure G1 PROPELLER MR images of brain (3x3 display), Region of Interest is Globe, Lens and arteries of brain

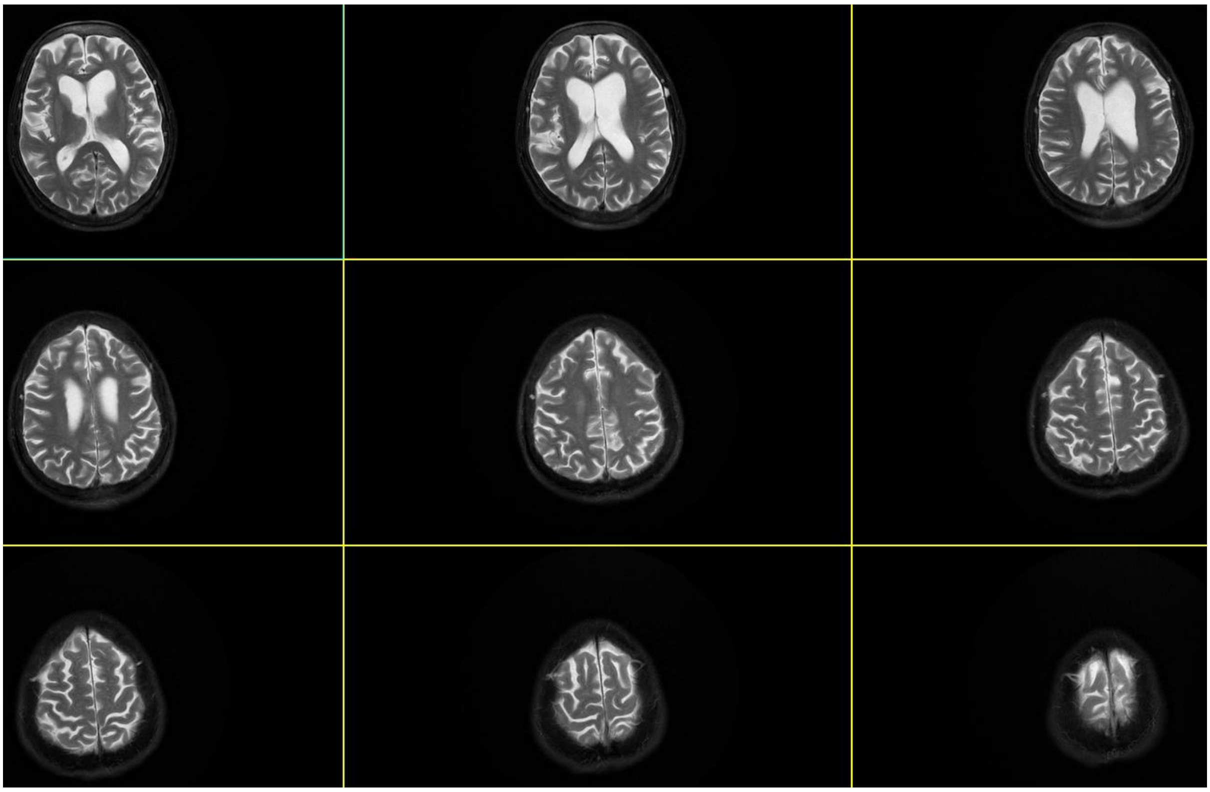

Figure G2 PROPELLER MR images of brain (3x3 display), Region of Interest is Middle Cerebral Artery and tissues of brain

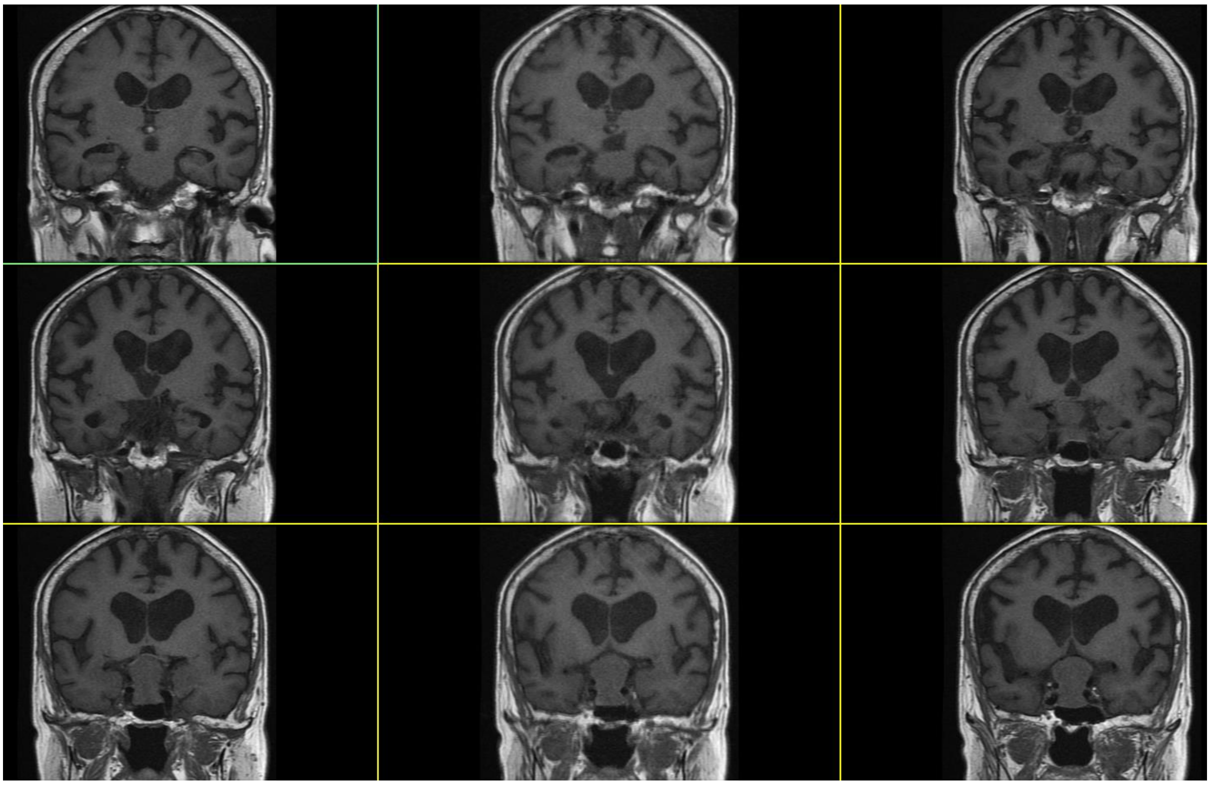

Figure H1 T1 Weighed Magnetic Resonance Images in Coronal plane (3x3 display), Region of Interest is arteries and tissues of brain

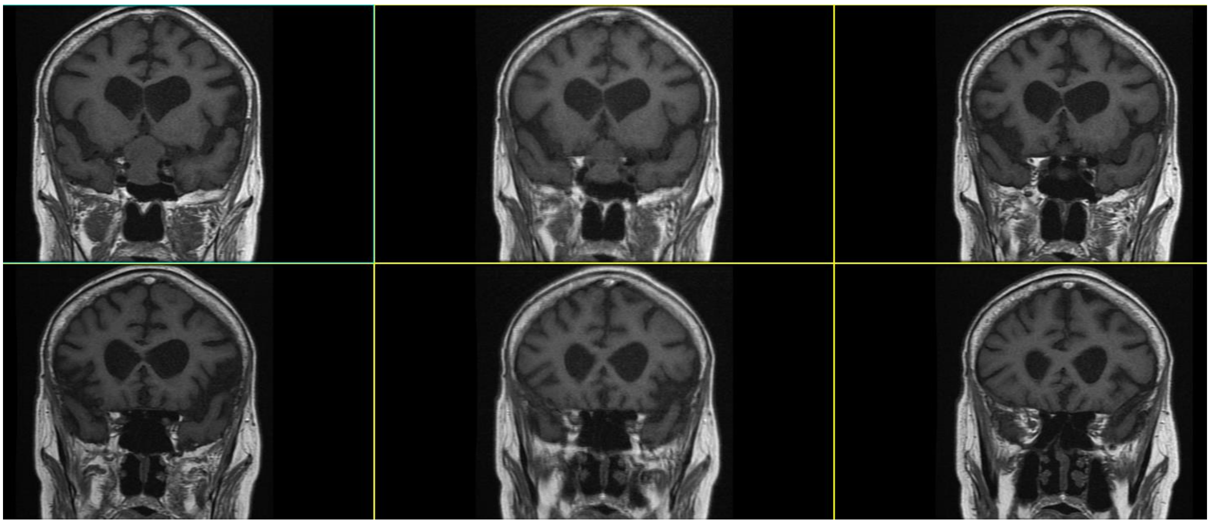

Figure H2 T1 Weighed Magnetic Resonance Images in Coronal plane (2x3 display), Region of Interest is arteries and tissues of brain

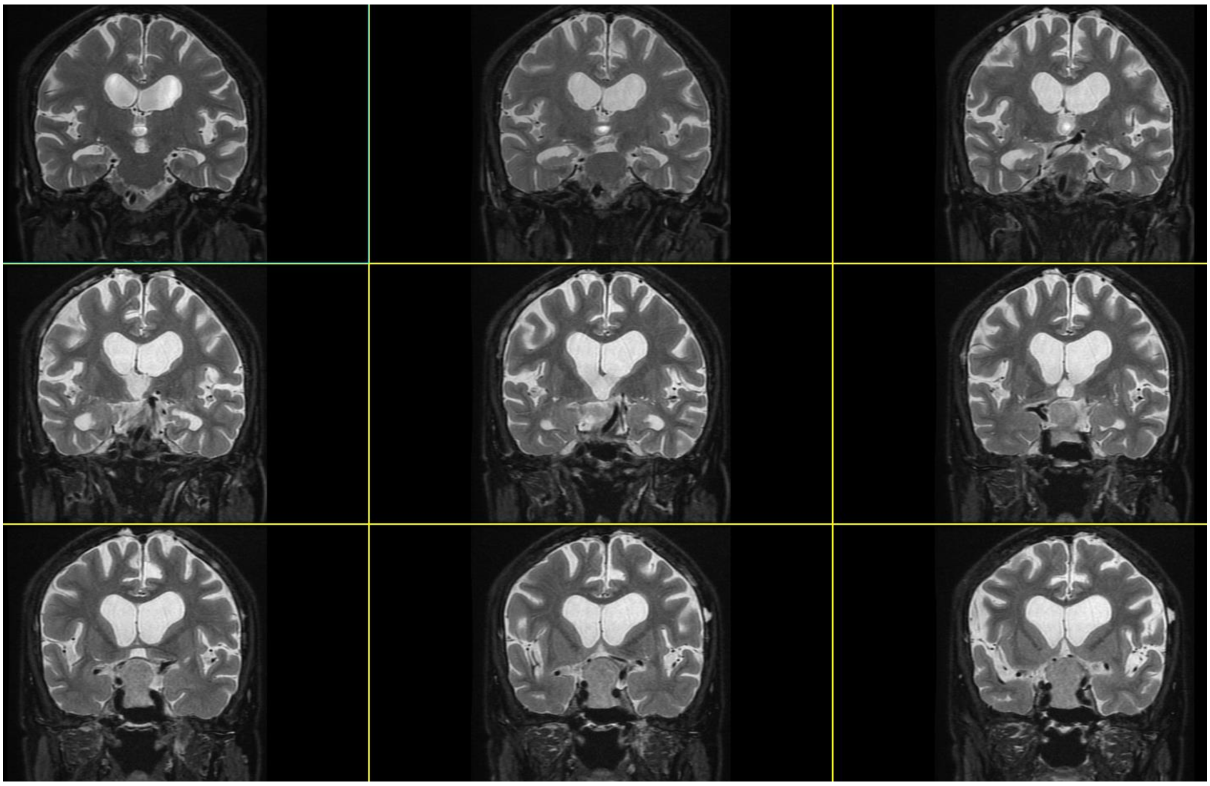

Figure I1 T2 Weighed Magnetic Resonance Images in Coronal plane (3x3 display), Region of Interest is arteries and tissues of brain

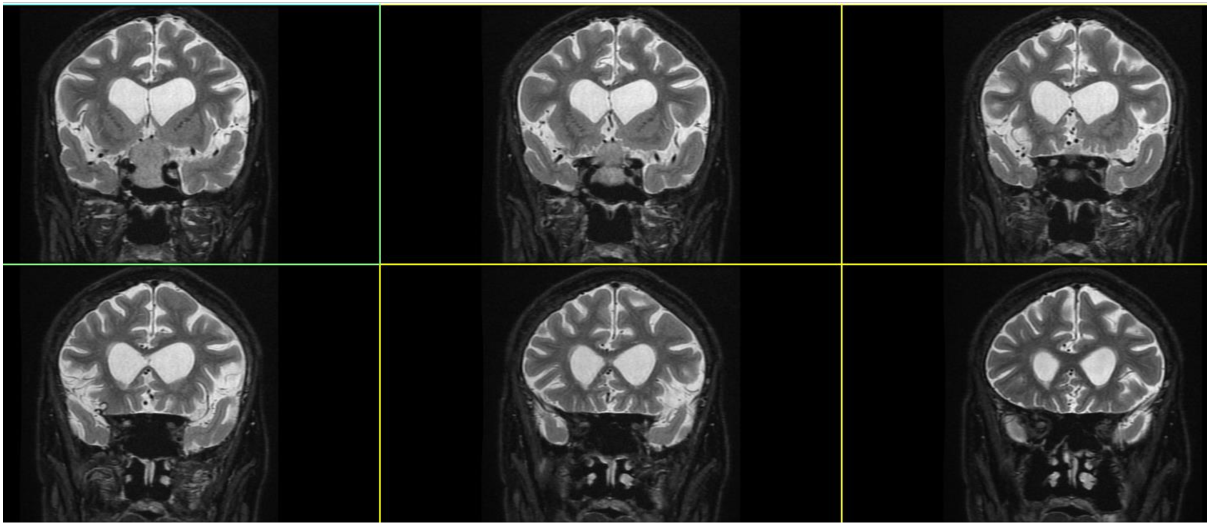

Figure I2 T2 Weighed Magnetic Resonance Images in Coronal plane (2x3 display), Region of Interest is arteries and tissues of brain

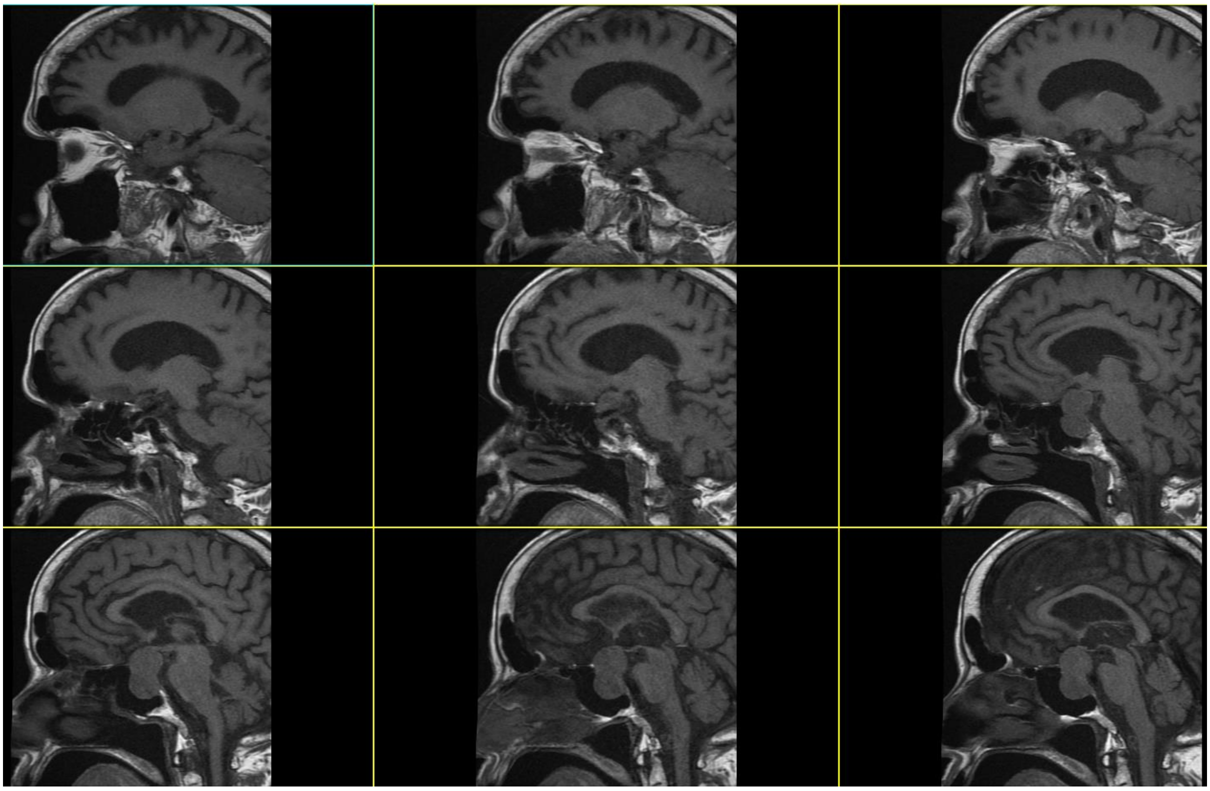

Figure J1 T1 Weighed Magnetic Resonance Images in Sagittal plane (3x3 display), Region of Interest is arteries and tissues of brain

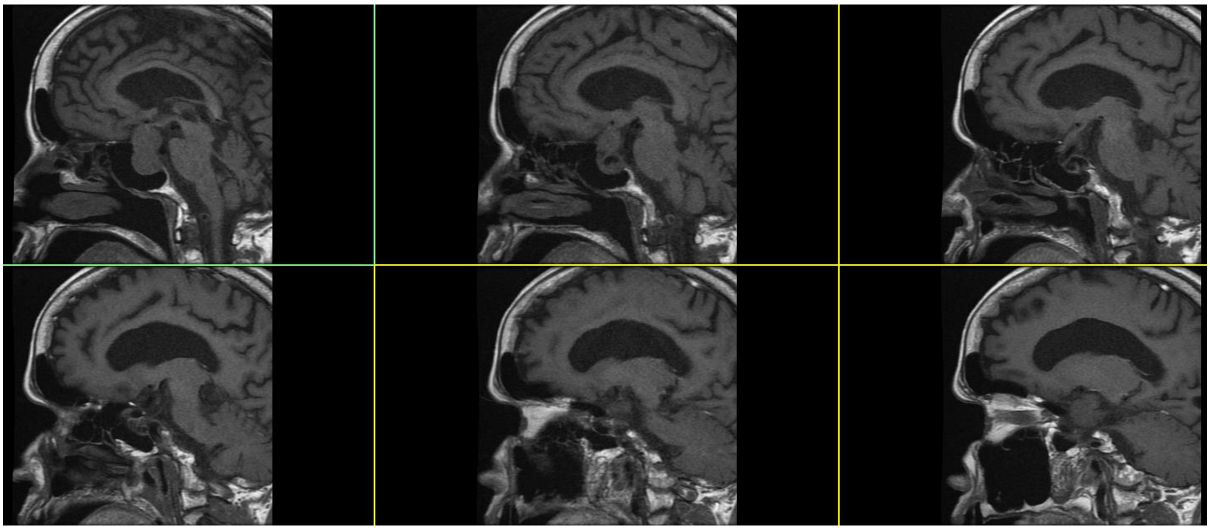

Figure J2 T1 Weighed Magnetic Resonance Images in Sagittal plane (2x3 display), Region of Interest is arteries and tissues of brain

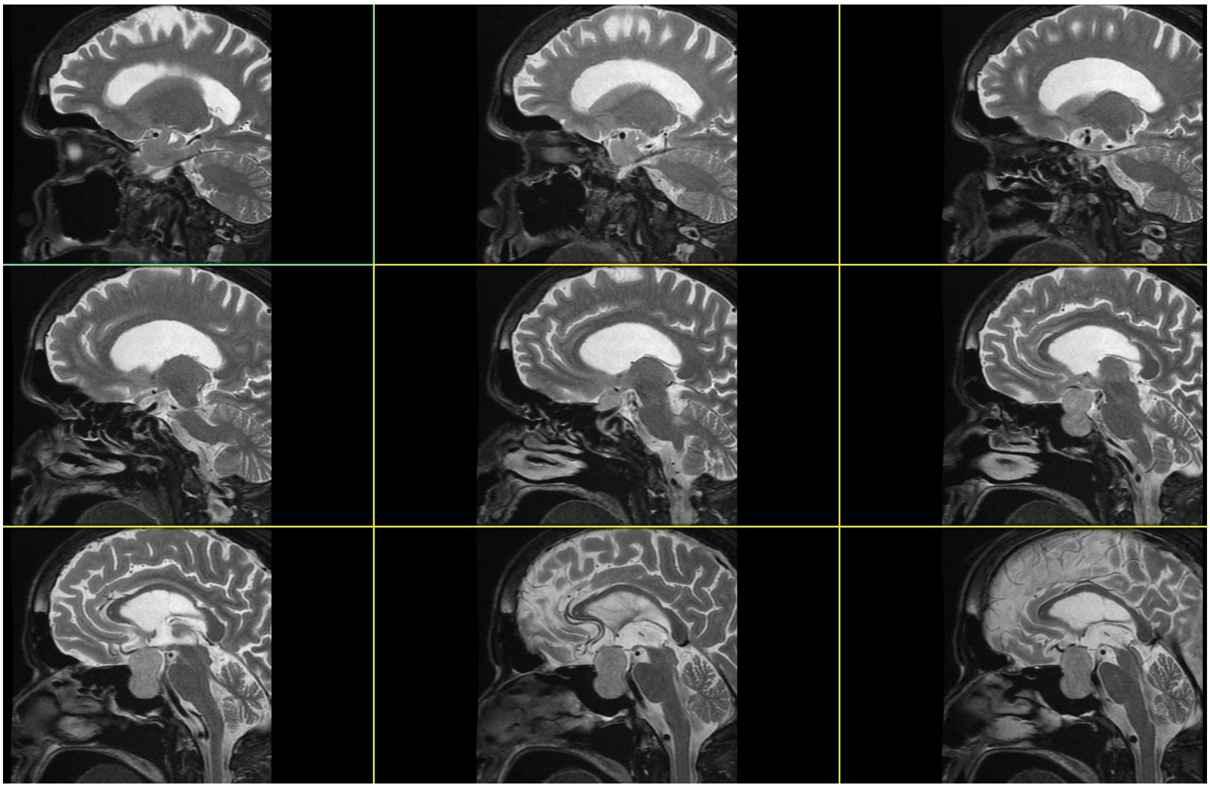

Figure K1 T2 Weighed Magnetic Resonance Images in Sagittal plane (3x3 display), Region of Interest is arteries and tissues of brain

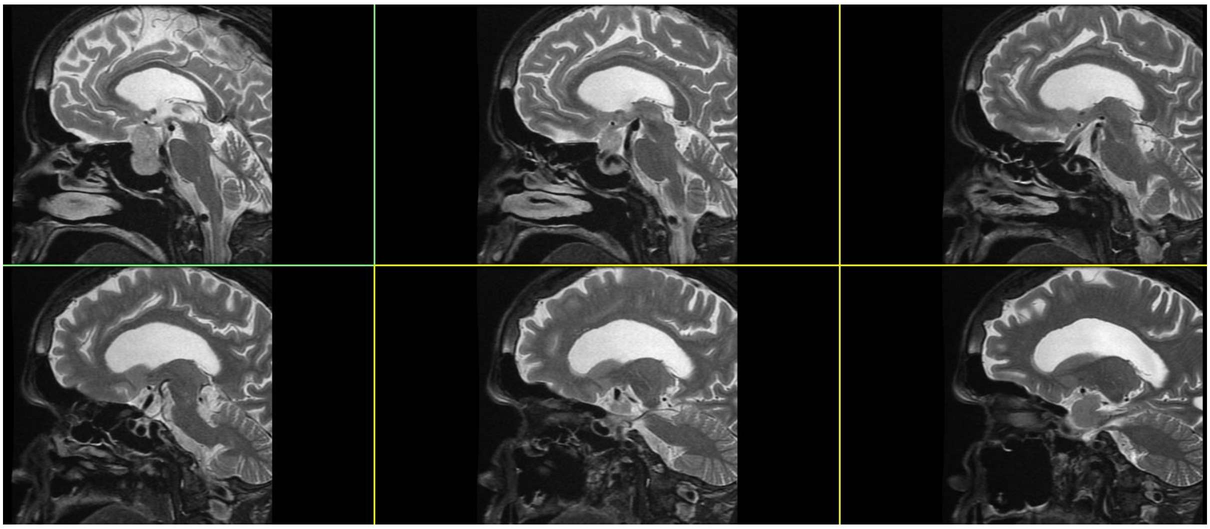

Figure K2 T2 Weighed Magnetic Resonance Images in Sagittal plane (2x3 display), Region of Interest is arteries and tissues of brain

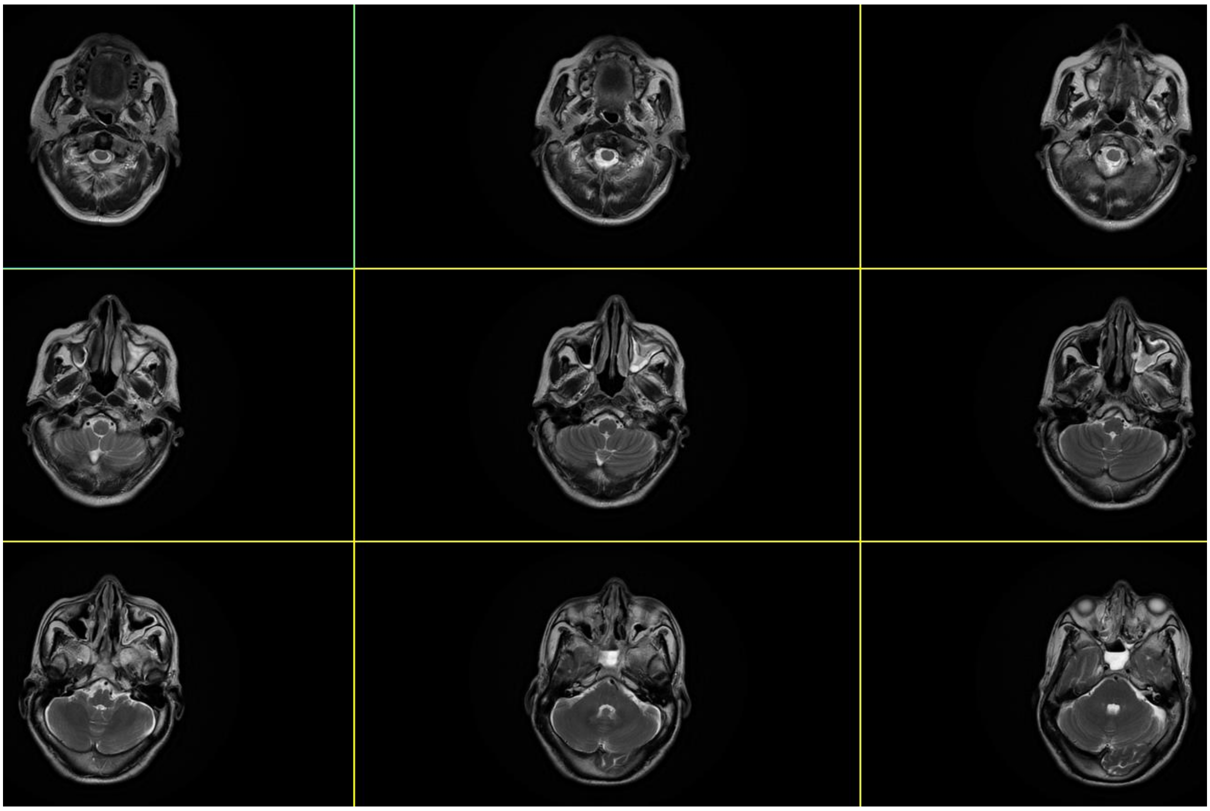

Figure L1 PROPELLER T2 weighed MR images of brain (3x3 display), Region of Interest is Globe, Lens and arteries of brain

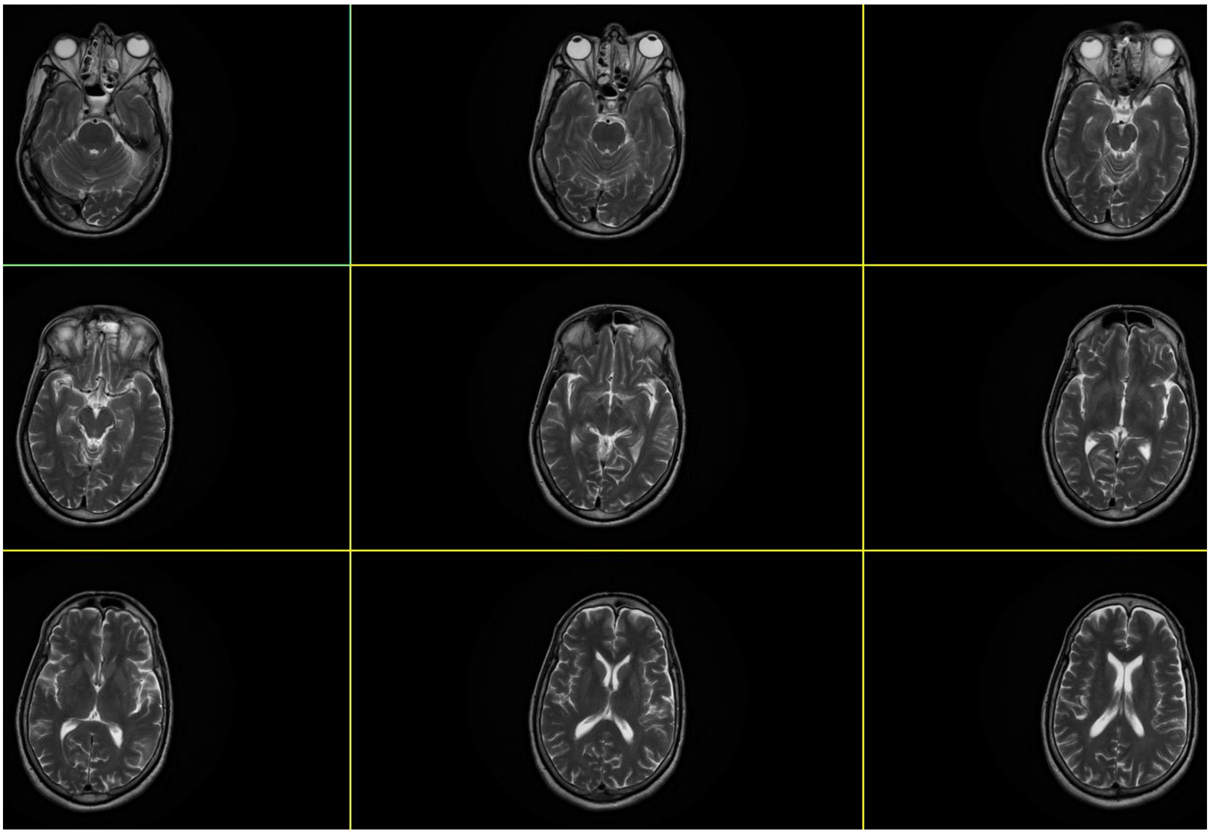

Figure L2 PROPELLER T2 weighed MR images of brain (3x3 display), Region of Interest is Globe, Lens, Middle Cerebral Artery and tissues of brain

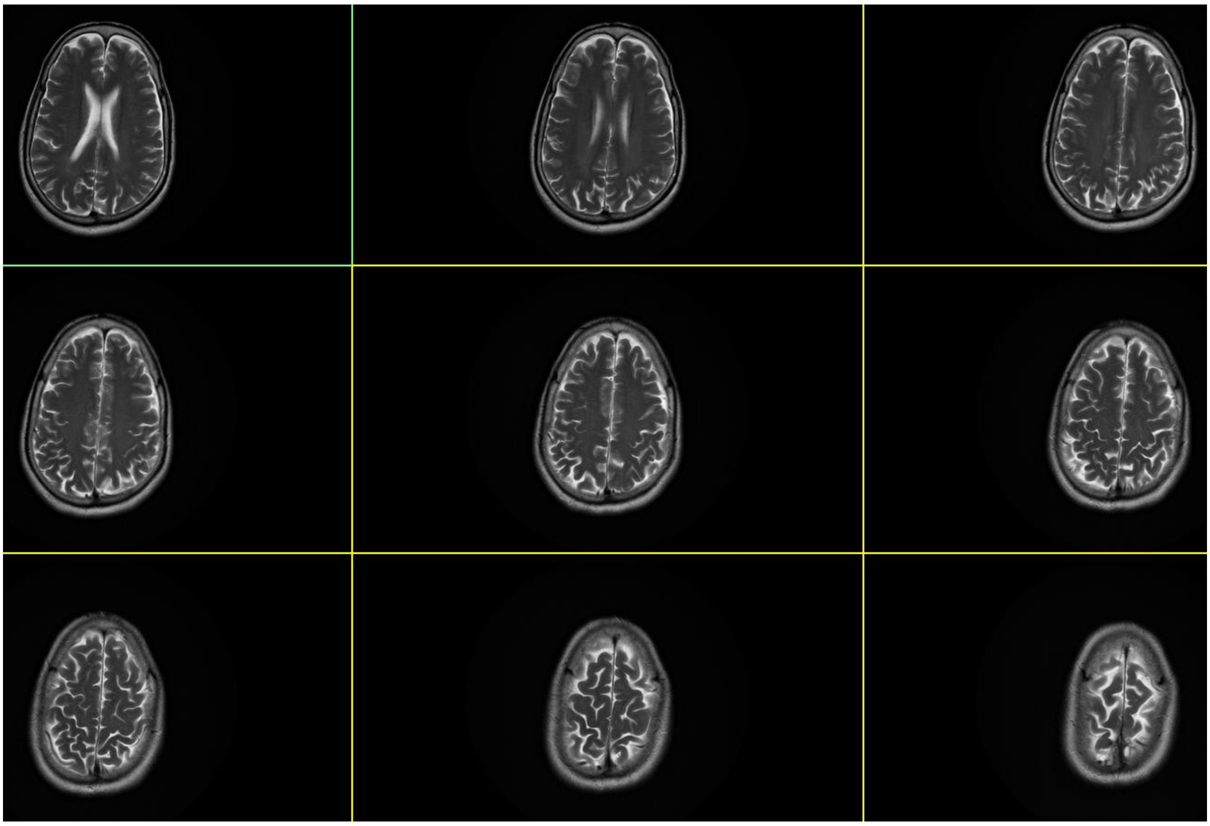

Figure L3 PROPELLER T2 weighed MR images of brain (3x3 display), Region of Interest is Globe, Lens, Middle Cerebral Artery and tissues of brain

All the above three images (figures L1-L3), are acquired in axial plane

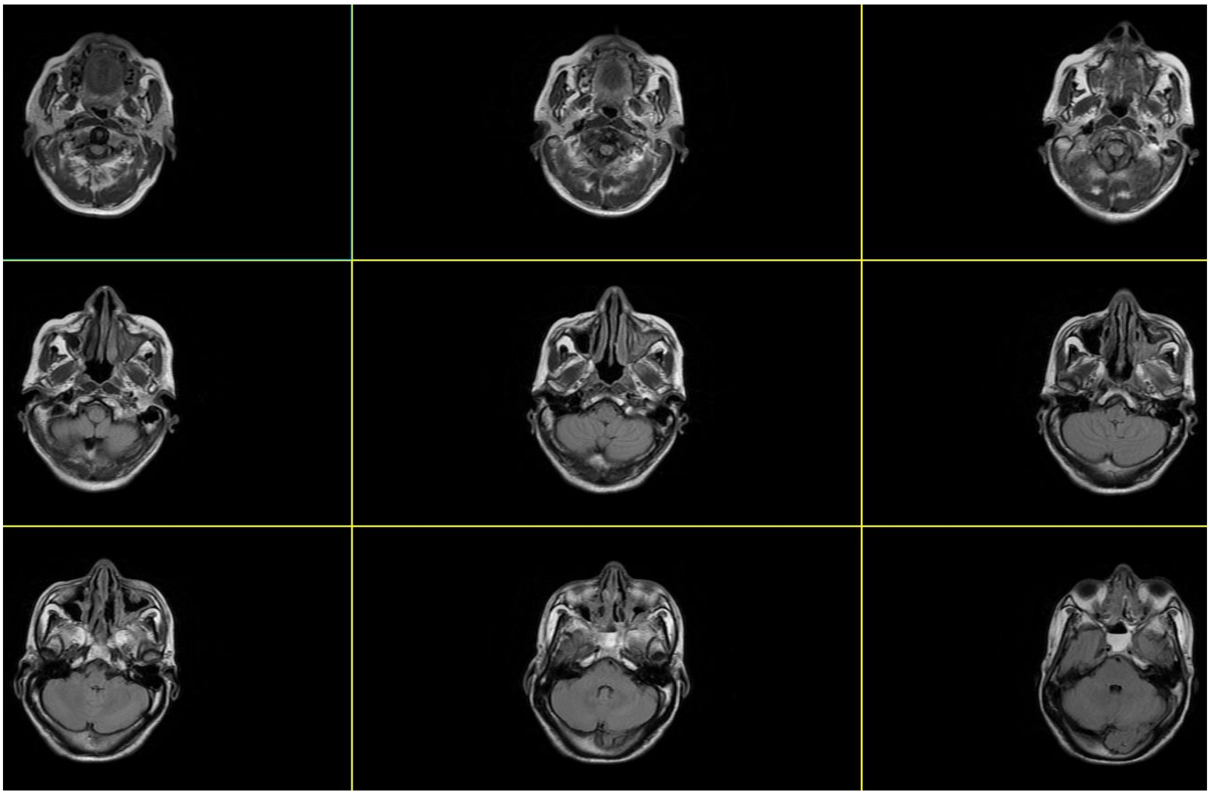

Figure M1 FLAIR T2 weighed MR images of brain (3x3 display), Region of Interest is Globe, Lens, and tissues of brain

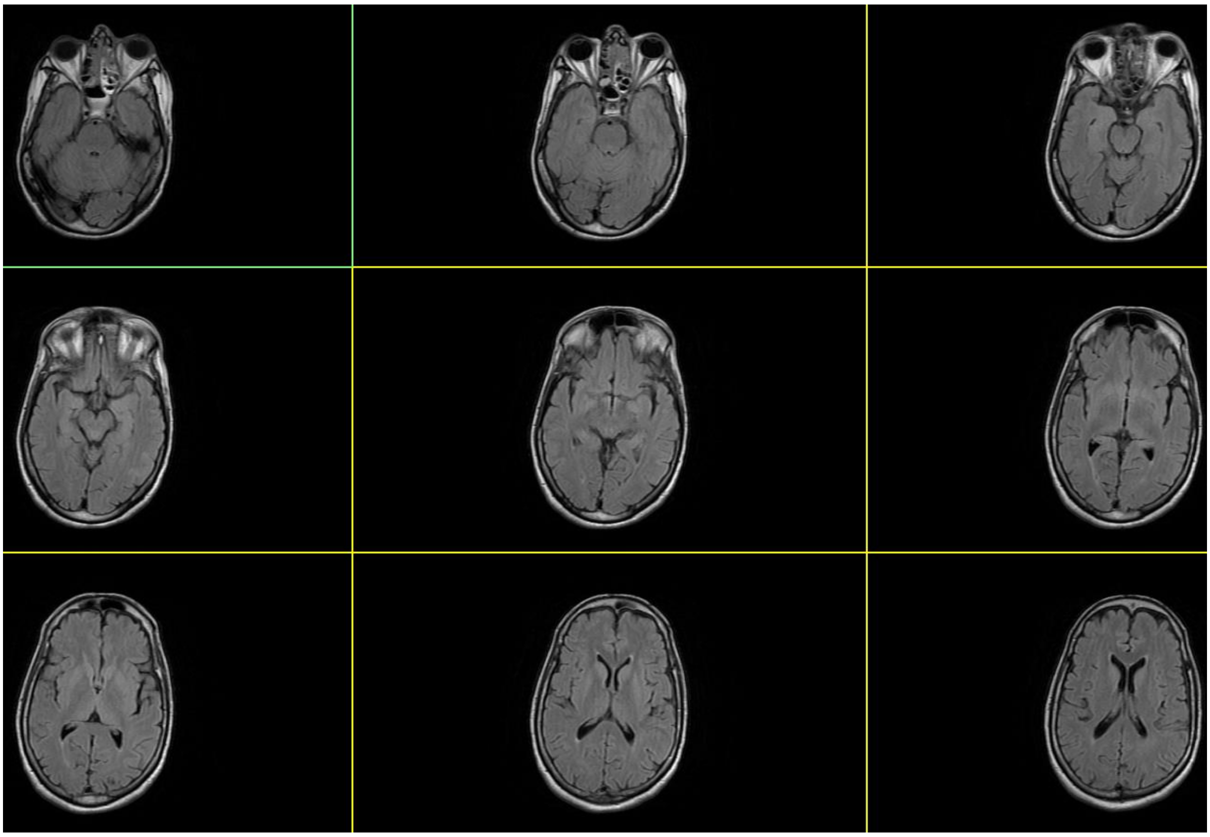

Figure M2 FLAIR T2 weighed MR images of brain (3x3 display), Region of Interest is Globe, Lens, and Middle Cerebral Artery of Brian

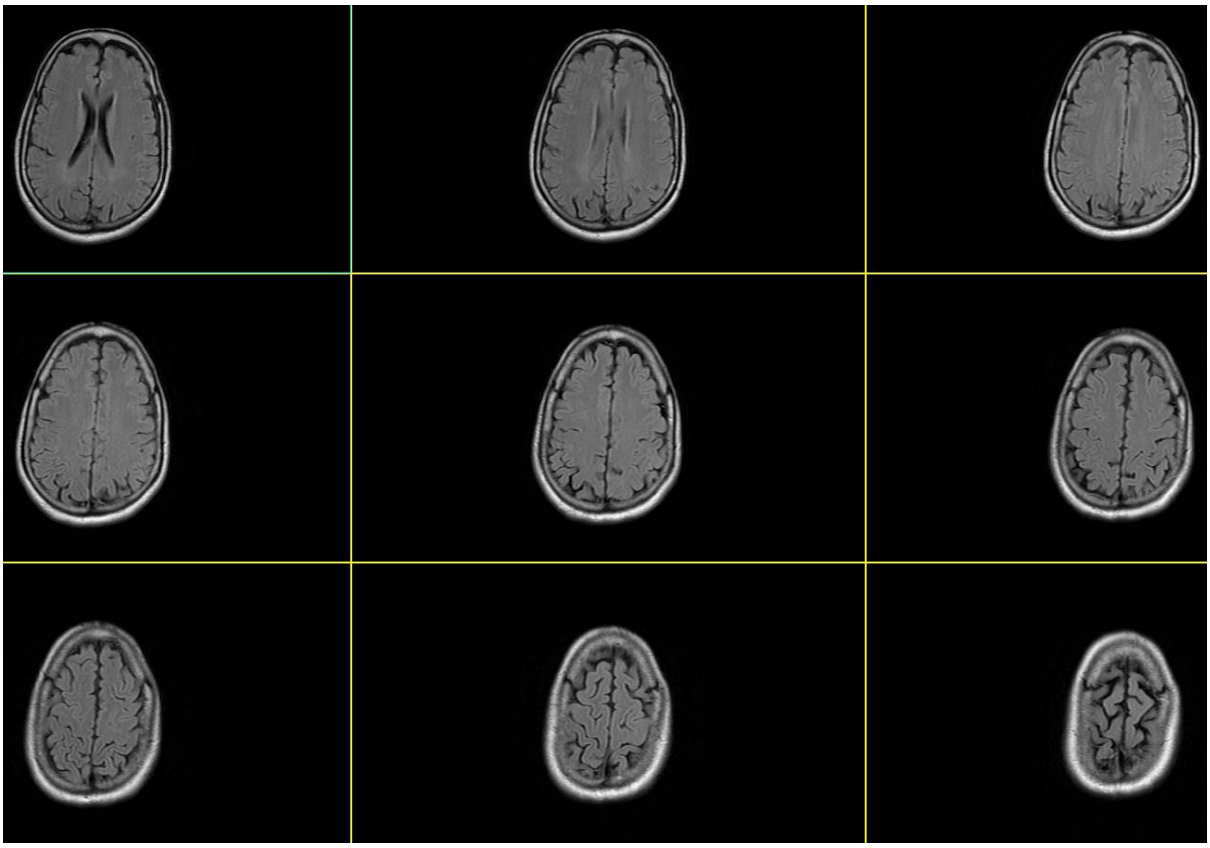

Figure M3 FLAIR T2 weighed MR images of brain (3x3 display), Region of Interest is Middle Cerebral Artery and tissues of Brian

All of the above images (figure M1-M3), are obtained at axial plane

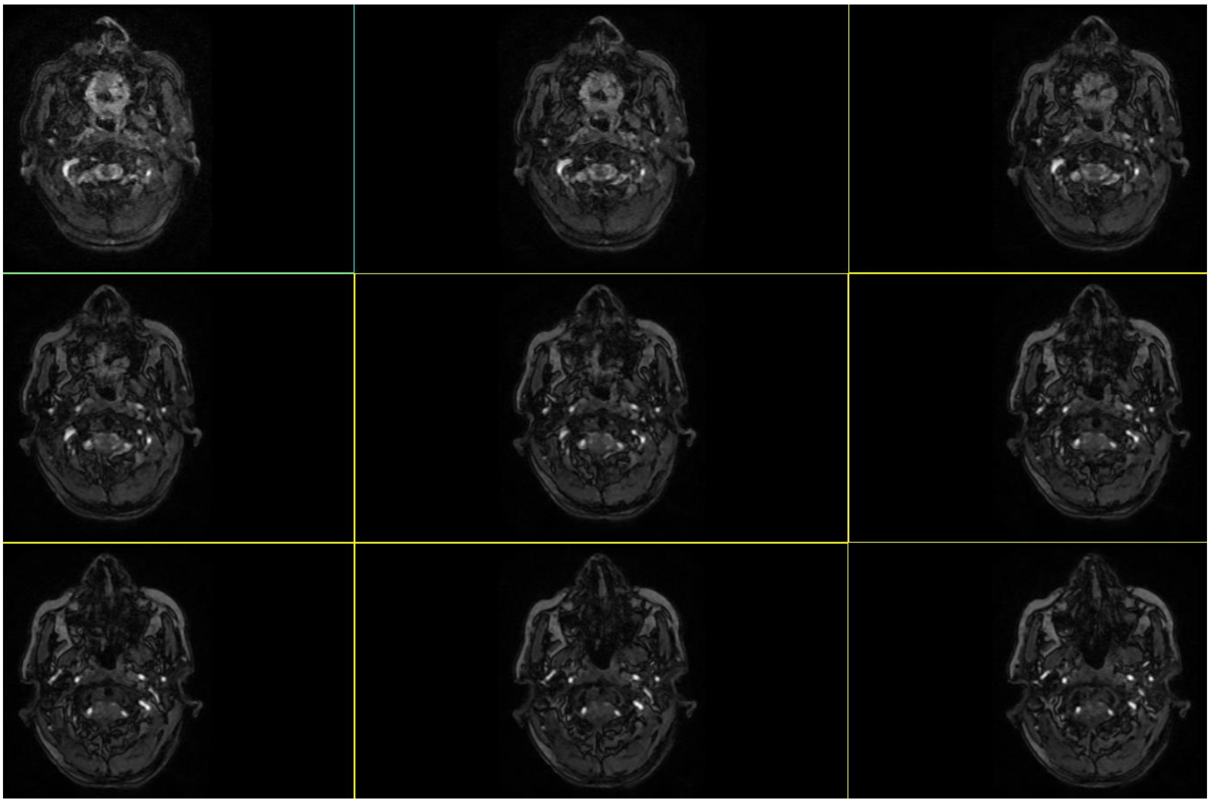

Figure N1 Magnetic Resonance Angiography image of brain (3x3 display), Region of Interest is Cerebral Arteries and tissues of Brian

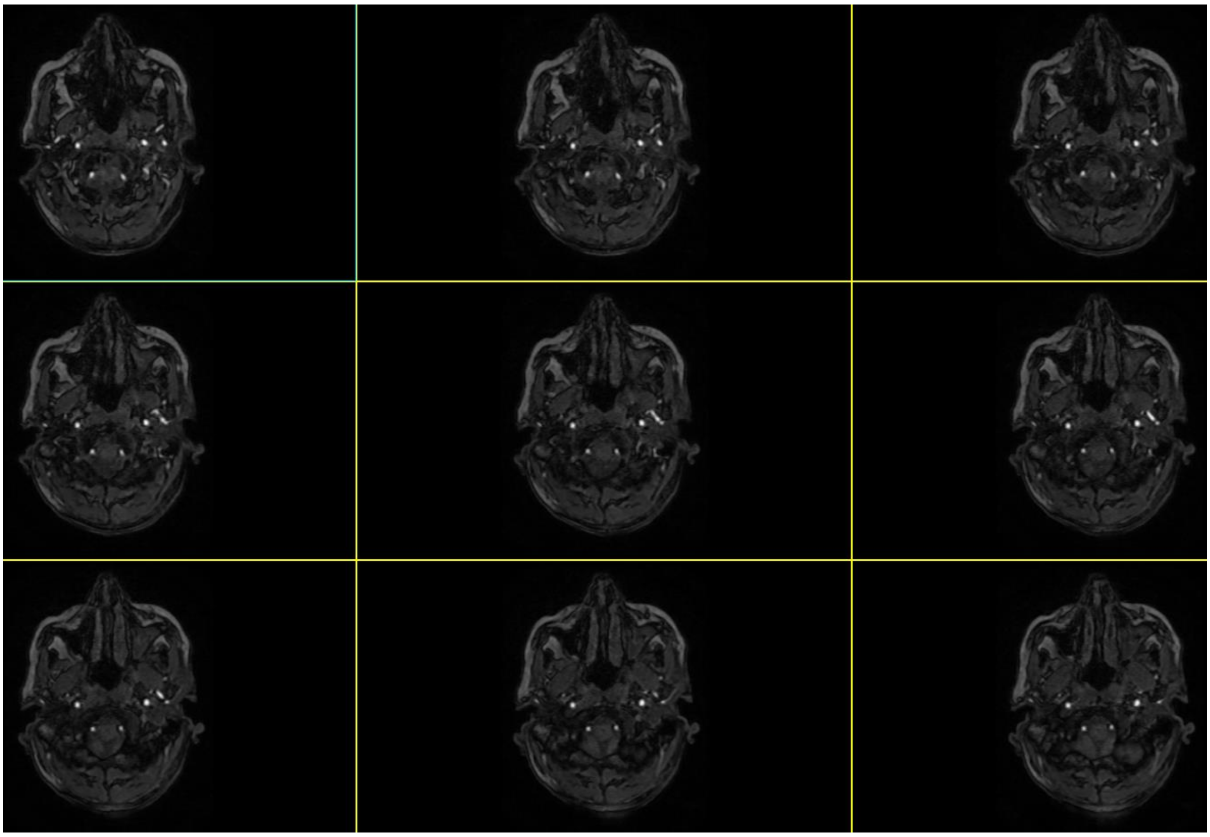

Figure N2 Magnetic Resonance Angiography image of brain (3x3 display), Region of Interest is Cerebral Arteries and tissues of Brian

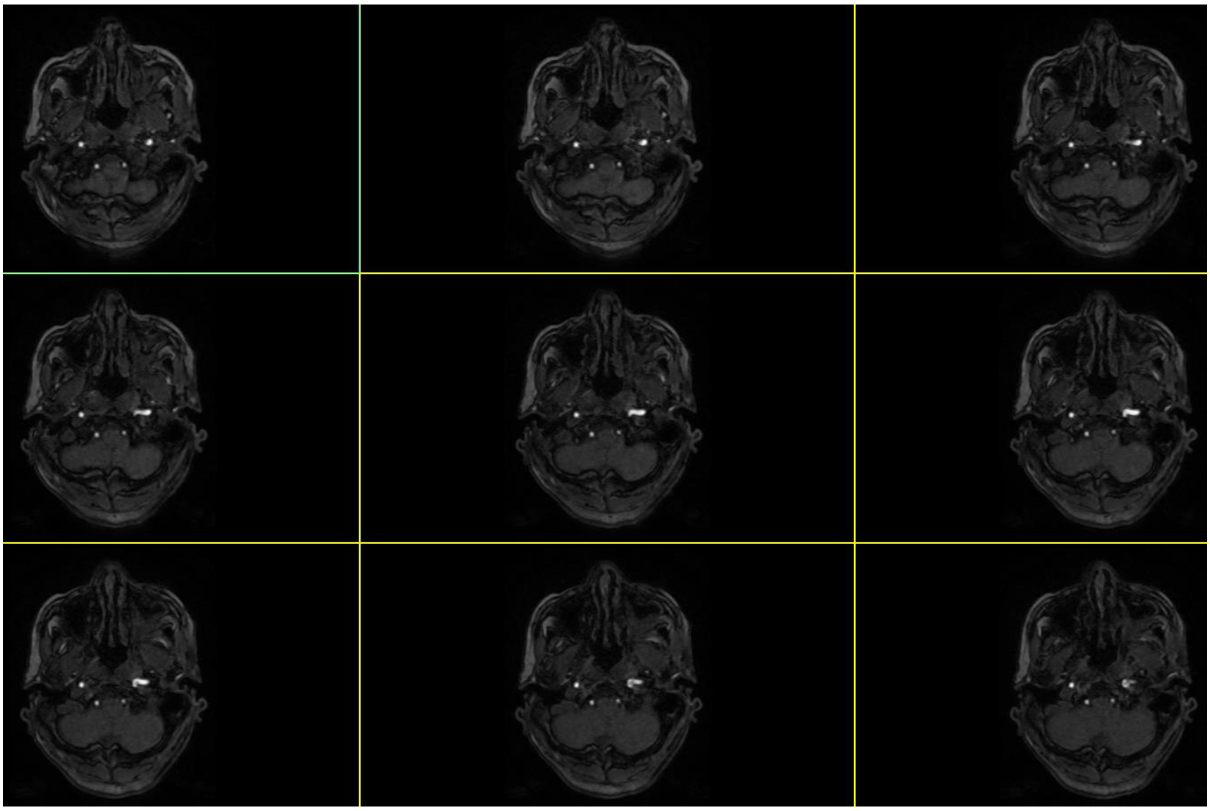

Figure N3 Magnetic Resonance Angiography image of brain (3x3 display), Region of Interest is Cerebral Arteries and tissues of Brian

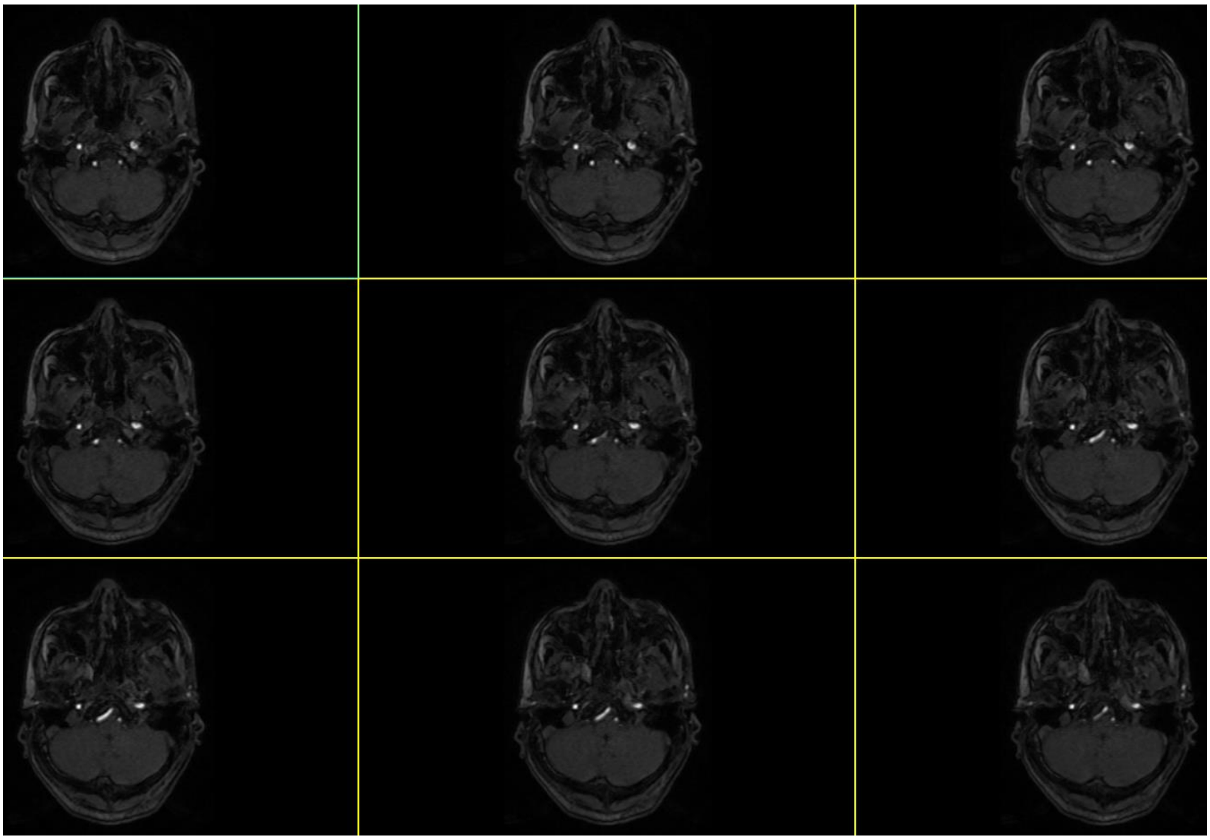

Figure N4 Magnetic Resonance Angiography image of brain (3x3 display), Region of Interest is Cerebral Arteries and tissues of Brian

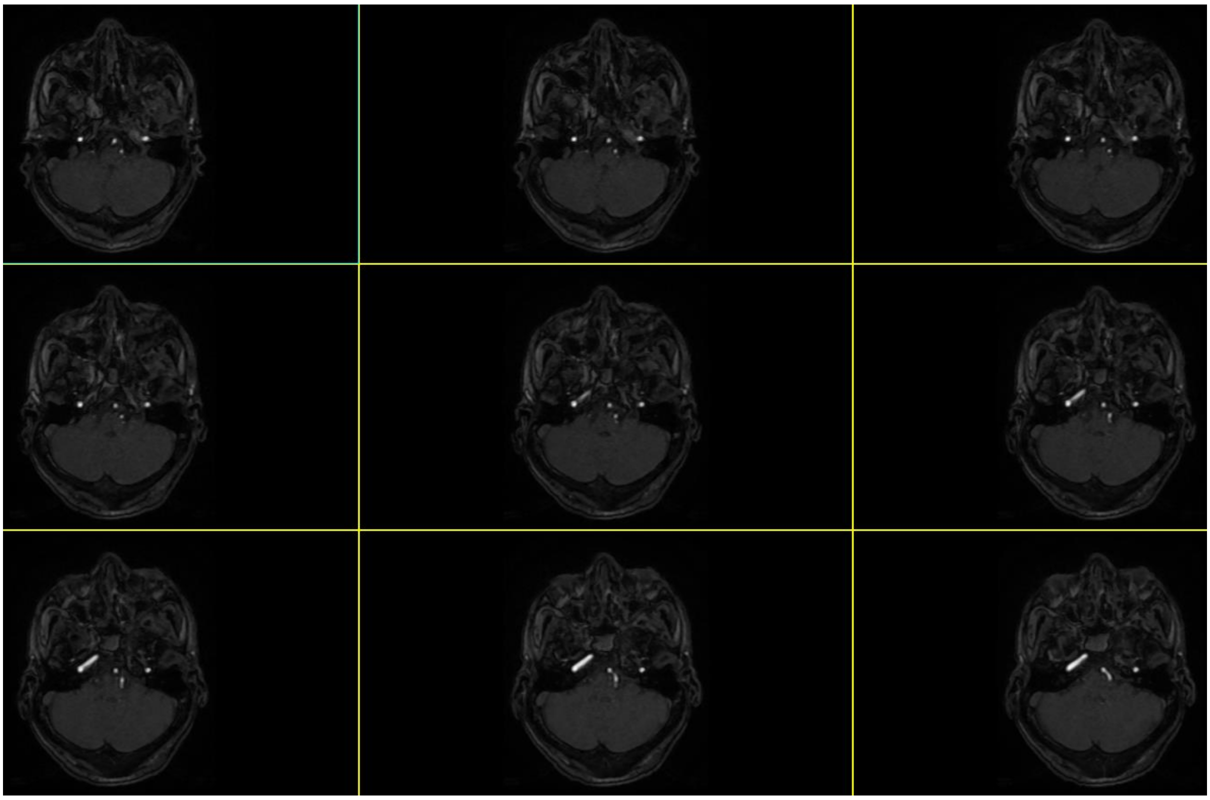

Figure N5 Magnetic Resonance Angiography image of brain (3x3 display), Region of Interest is Cerebral blood vessels and tissues of Brian

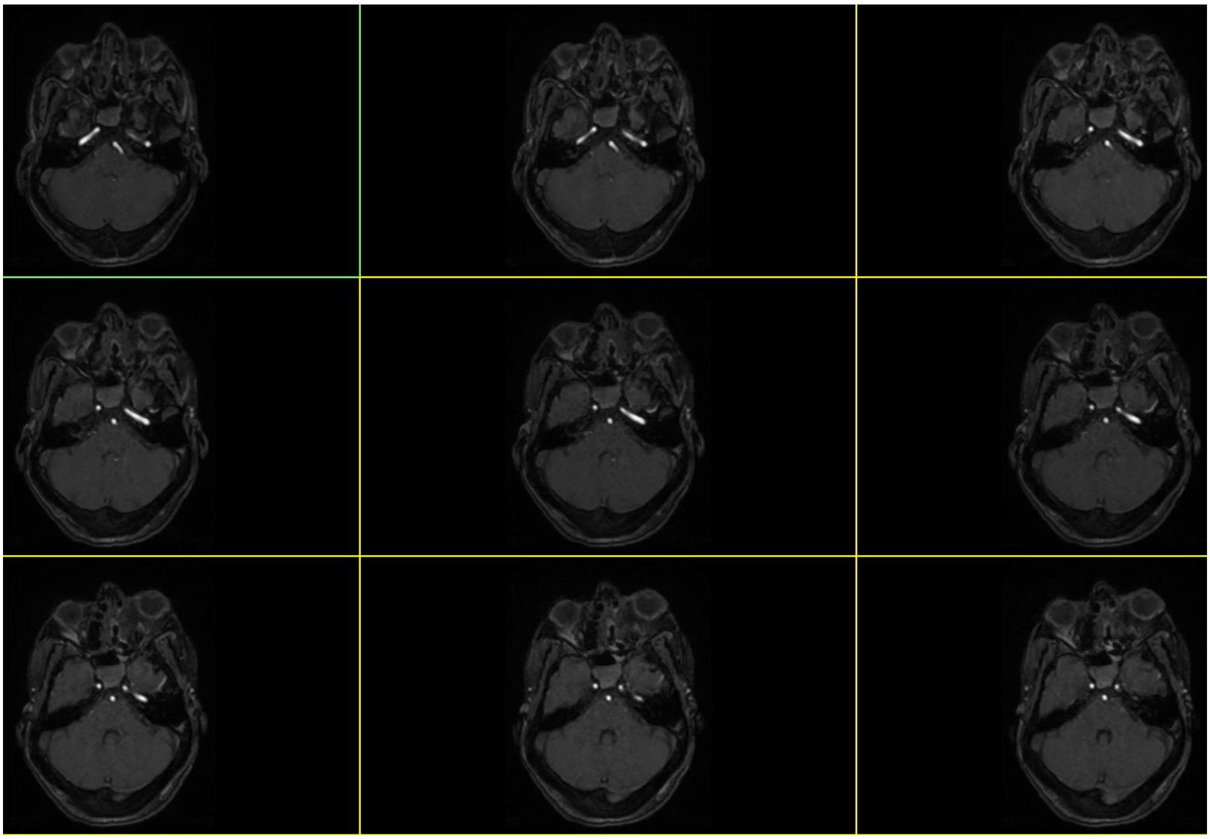

Figure N6 Magnetic Resonance Angiography image of brain (3x3 display), Region of Interest is Globe, Cerebral blood vessels and tissues of Brian

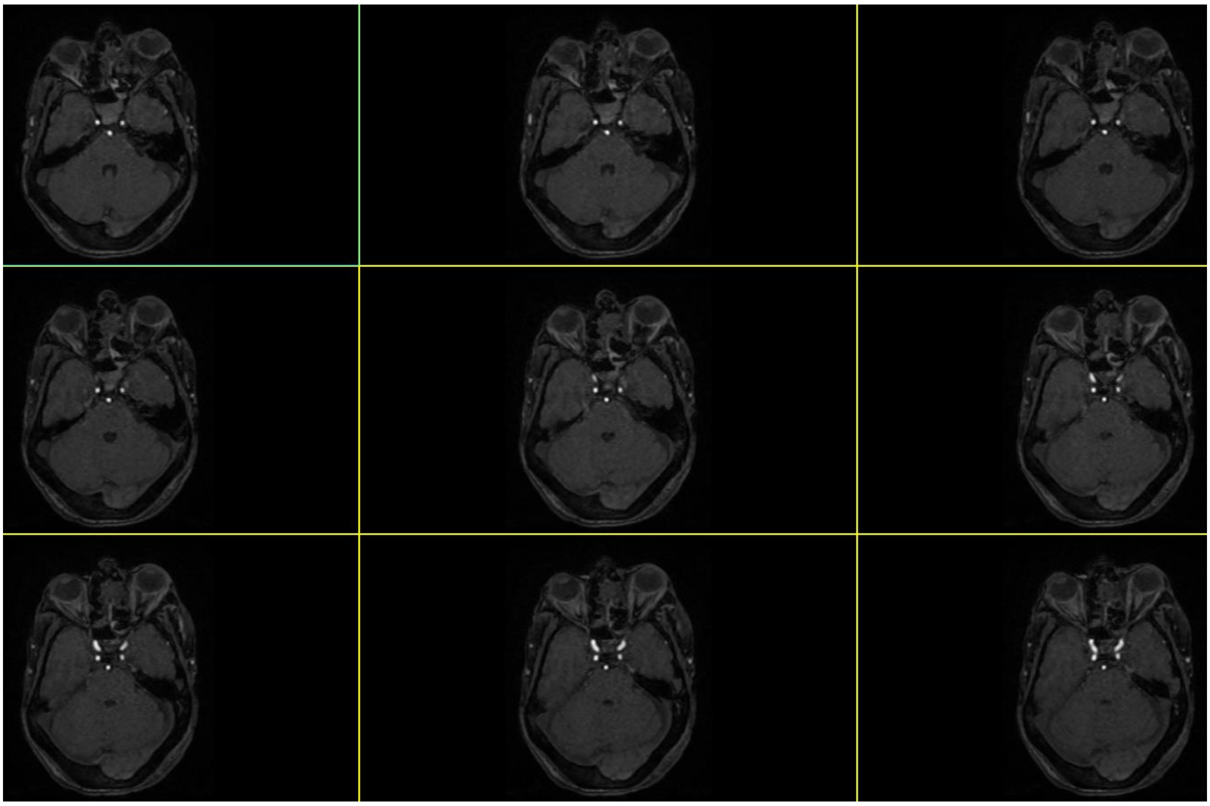

Figure N7 Magnetic Resonance Angiography image of brain (3x3 display), Region of Interest is Globe and Cerebral blood vessels

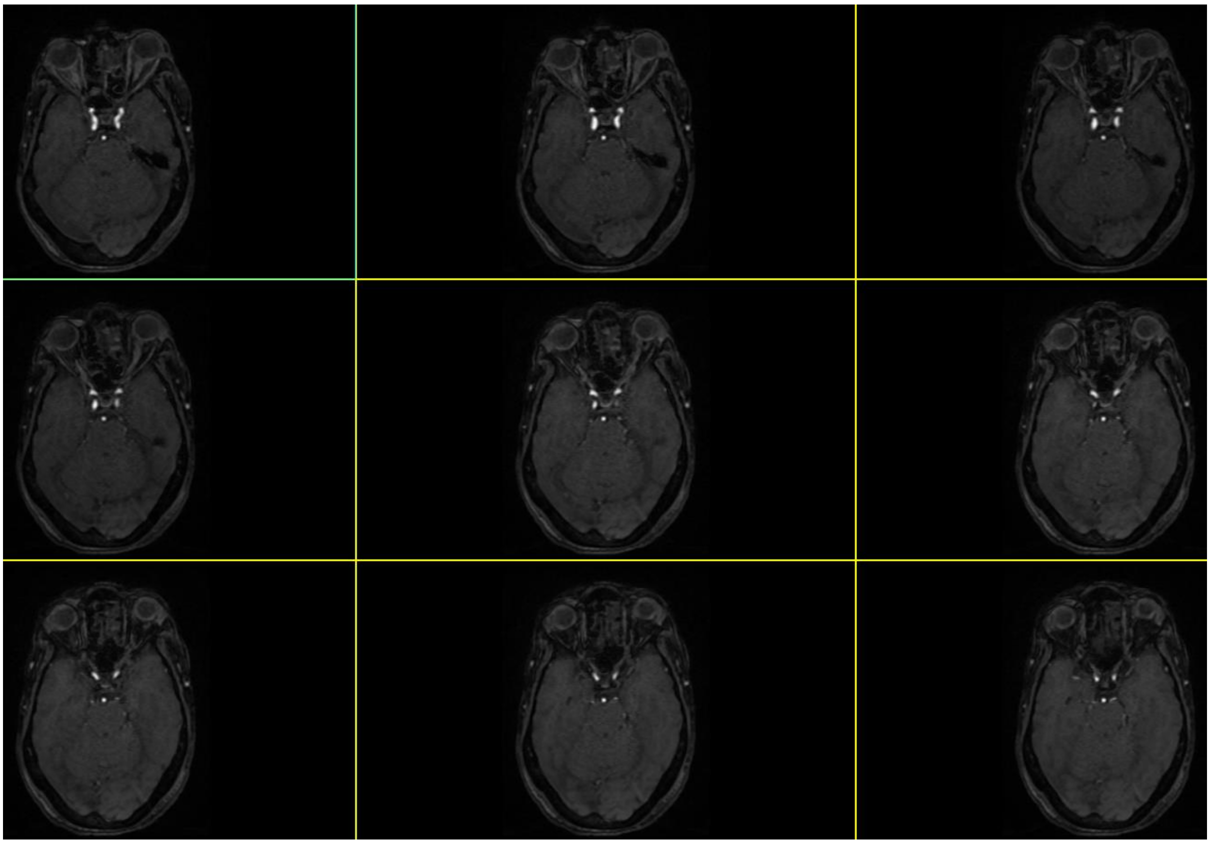

Figure N8 Magnetic Resonance Angiography image of brain (3x3 display), Region of Interest is Globe and Cerebral blood vessels

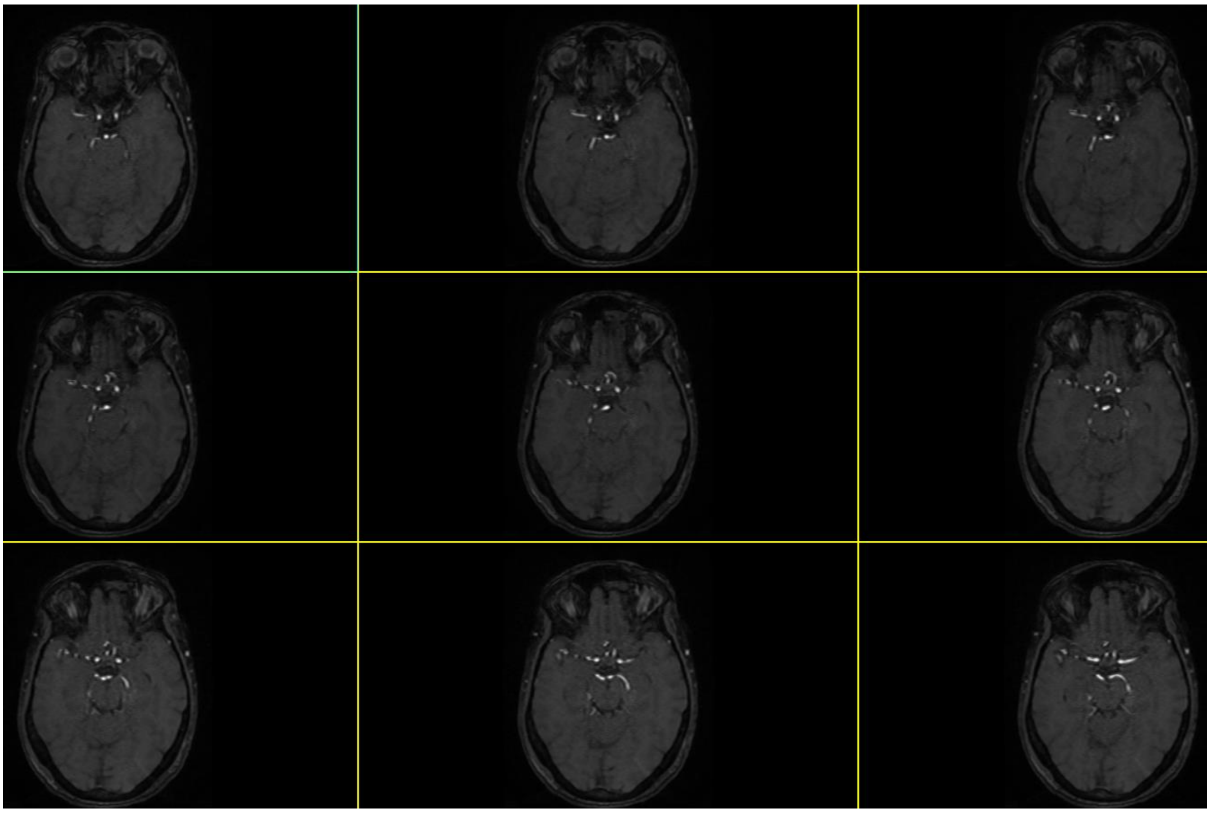

Figure N9 Magnetic Resonance Angiography image of brain (3x3 display), Region of Interest is Globe, Cerebral blood vessels and Cisterns in cerebral regions

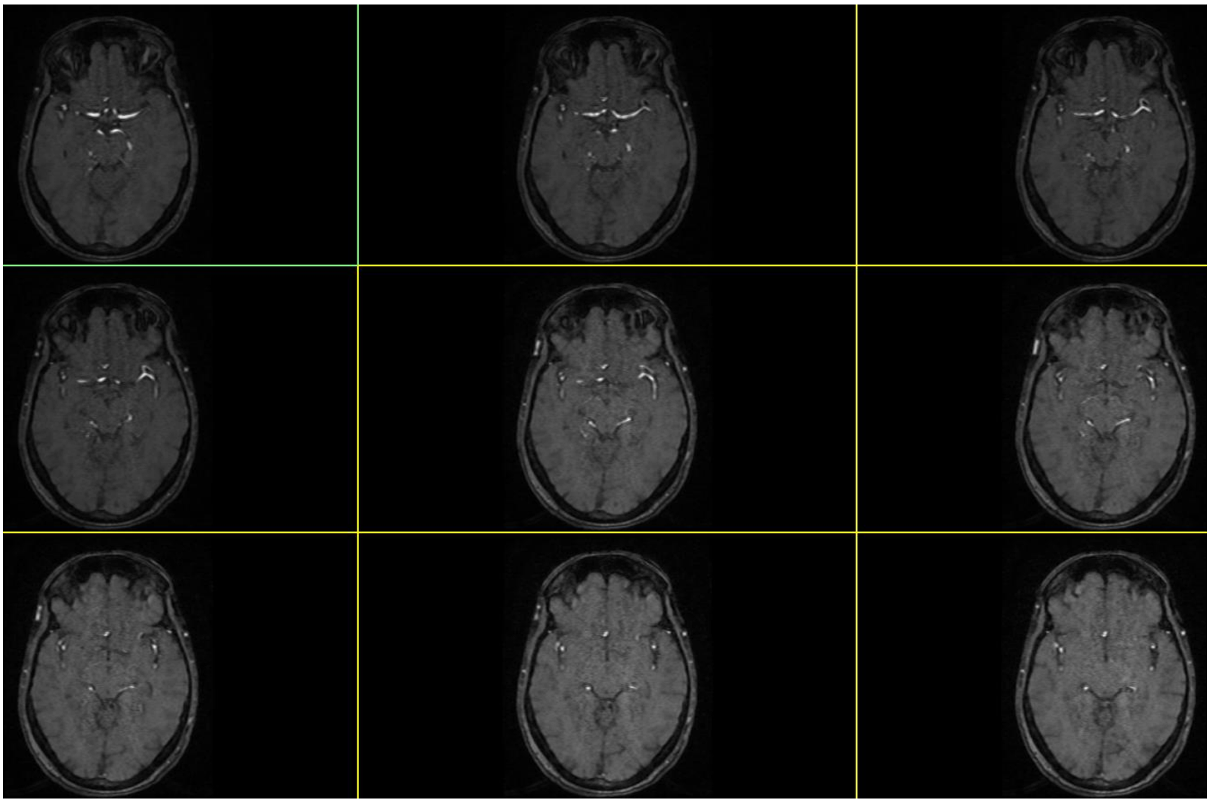

Figure N10 Magnetic Resonance Angiography image of brain (3x3 display), Region of Interest is Globe, Cerebral blood vessels and Cisterns in cerebral regions

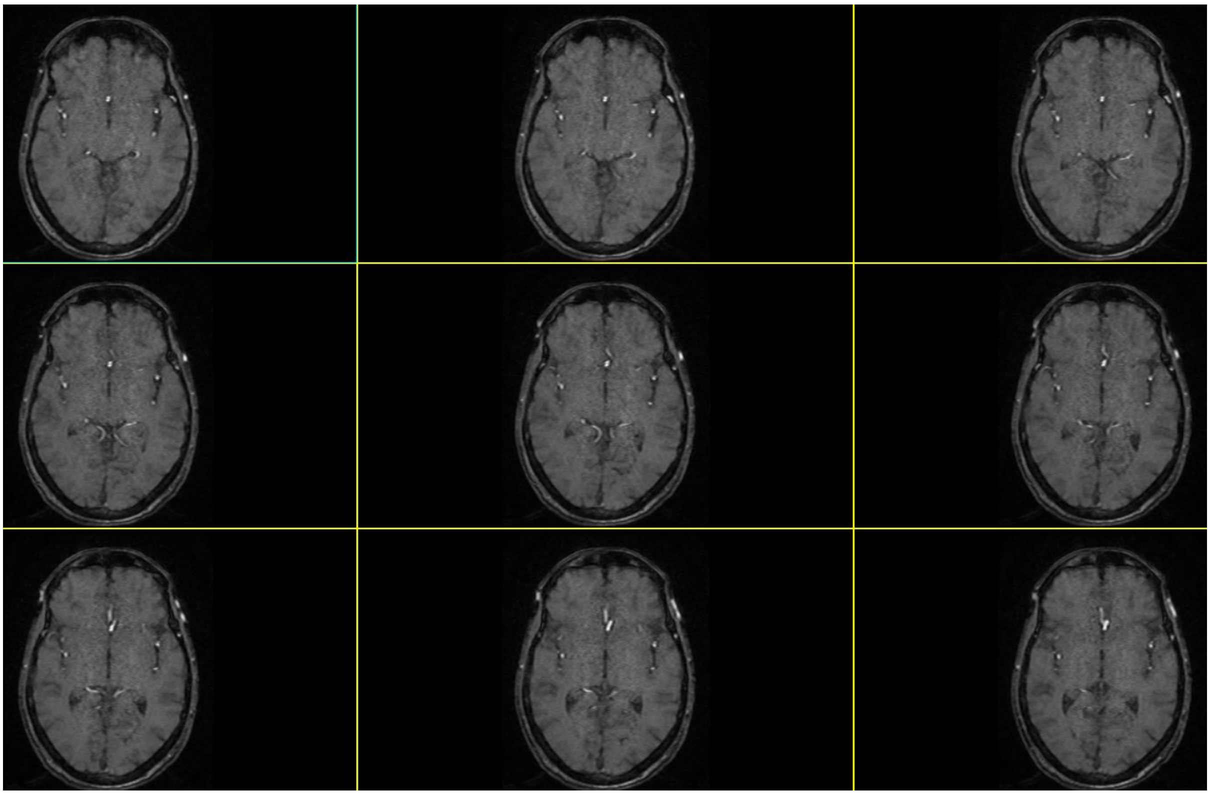

Figure N11 Magnetic Resonance Angiography image of brain (3x3 display), Region of Interest is arteries and Cisterns in cerebral regions

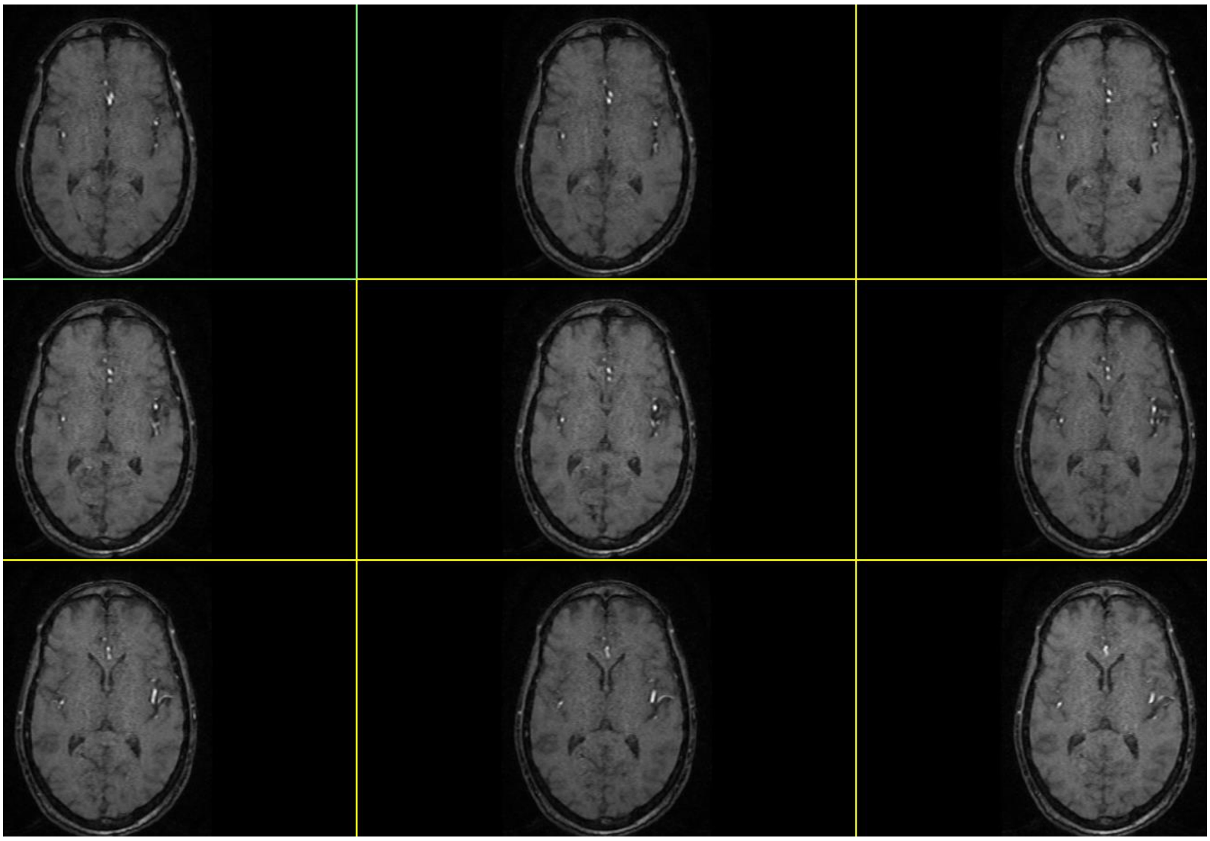

Figure N12 Magnetic Resonance Angiography image of brain (3x3 display), Region of Interest is Middle Cerebral Artery and Cisterns in cerebral regions

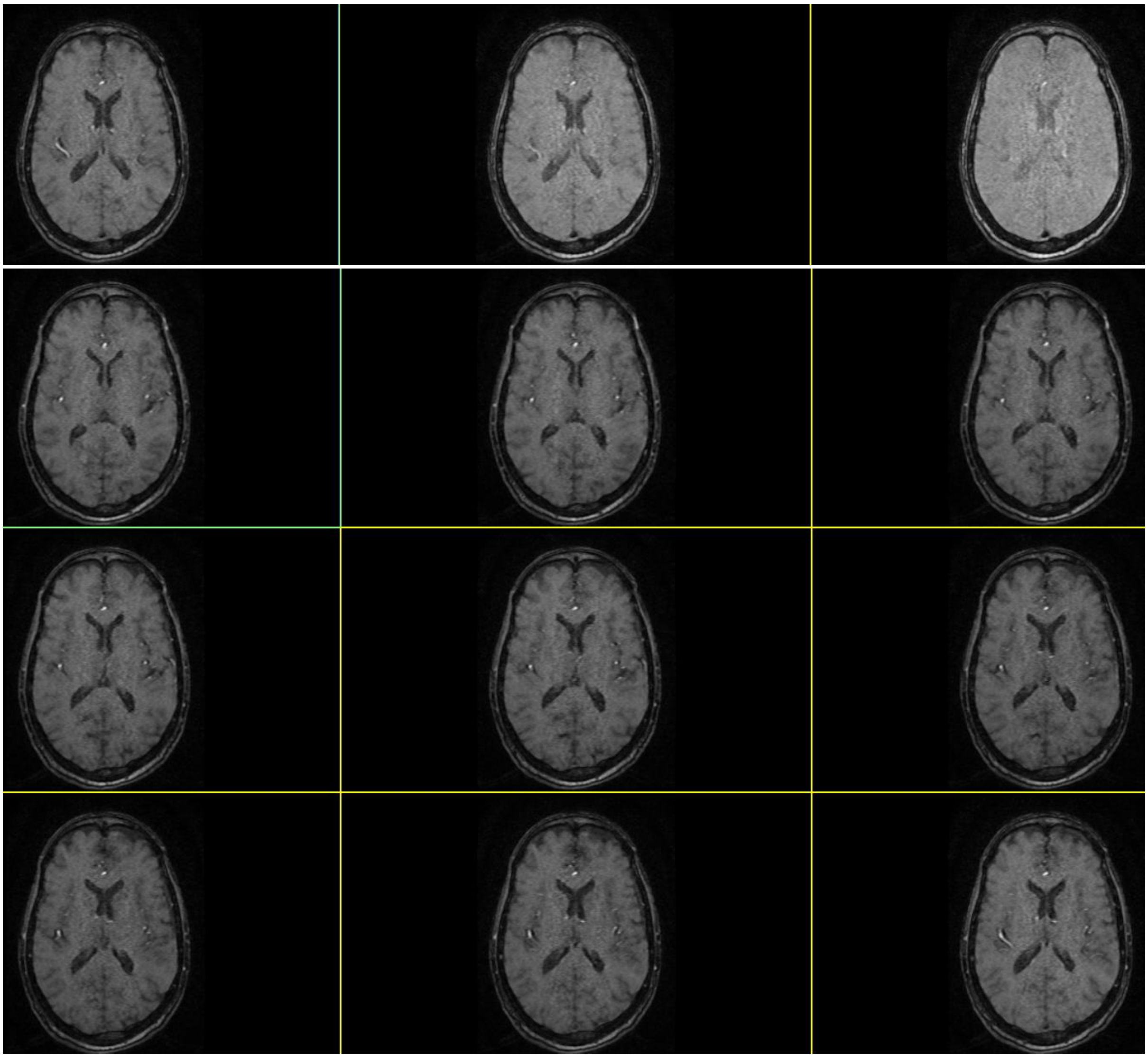

Figure N13 Magnetic Resonance Angiography image of brain (4x3 display), Region of Interest is Middle Cerebral Artery and tissues in cerebral regions

All of the above images (figure N1-N13) are obtained in the axial plane. This Magnetic Resonance Angiography MRA images of brain is mainly used for analyzation of blood vessels and tissues of brain. This is considered the best sequence for detection of any defects in structures of blood vessels of cerebral regions.

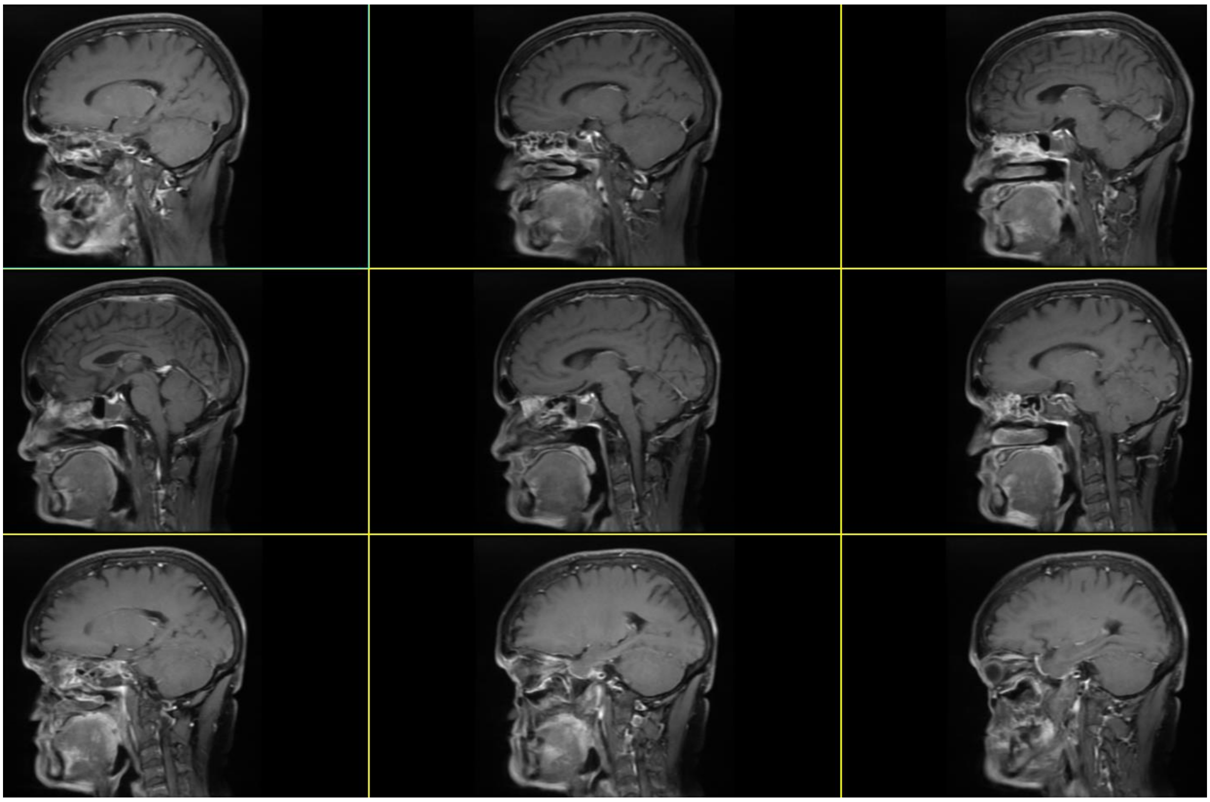

Figure O1 Multiplanar reconstructed Magnetic Resonance Images of Head in Sagittal plane (3x3 display), Region of Interest is Globe, cervical spine, arteries and tissues of brain

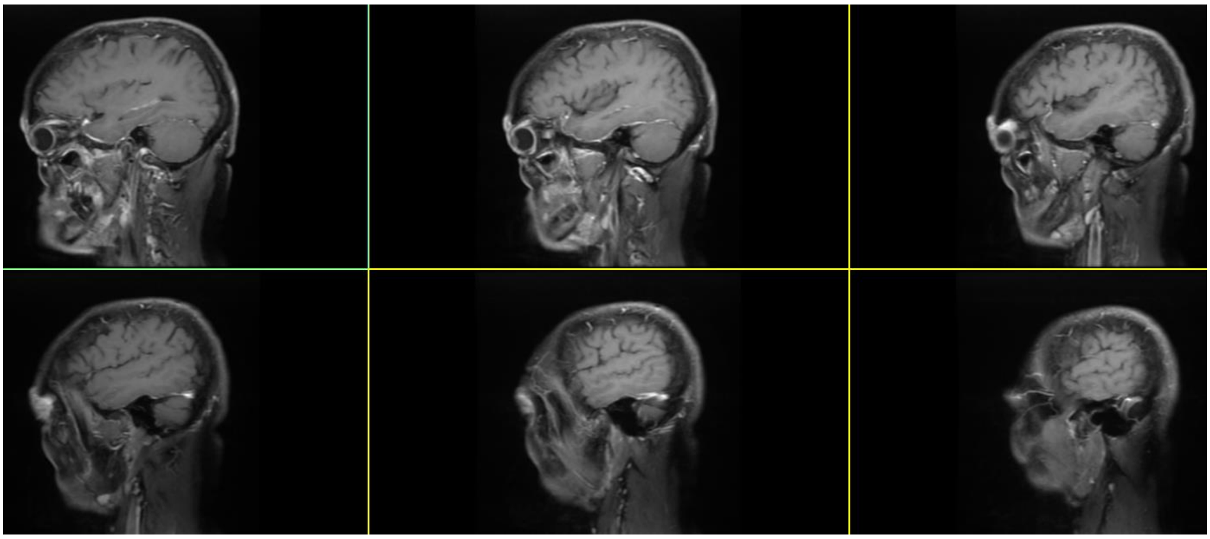

Figure O2 Multiplanar reconstructed Magnetic Resonance Images of Head in Sagittal plane (2x3 display), Region of Interest is Globe, arteries and tissues of brain

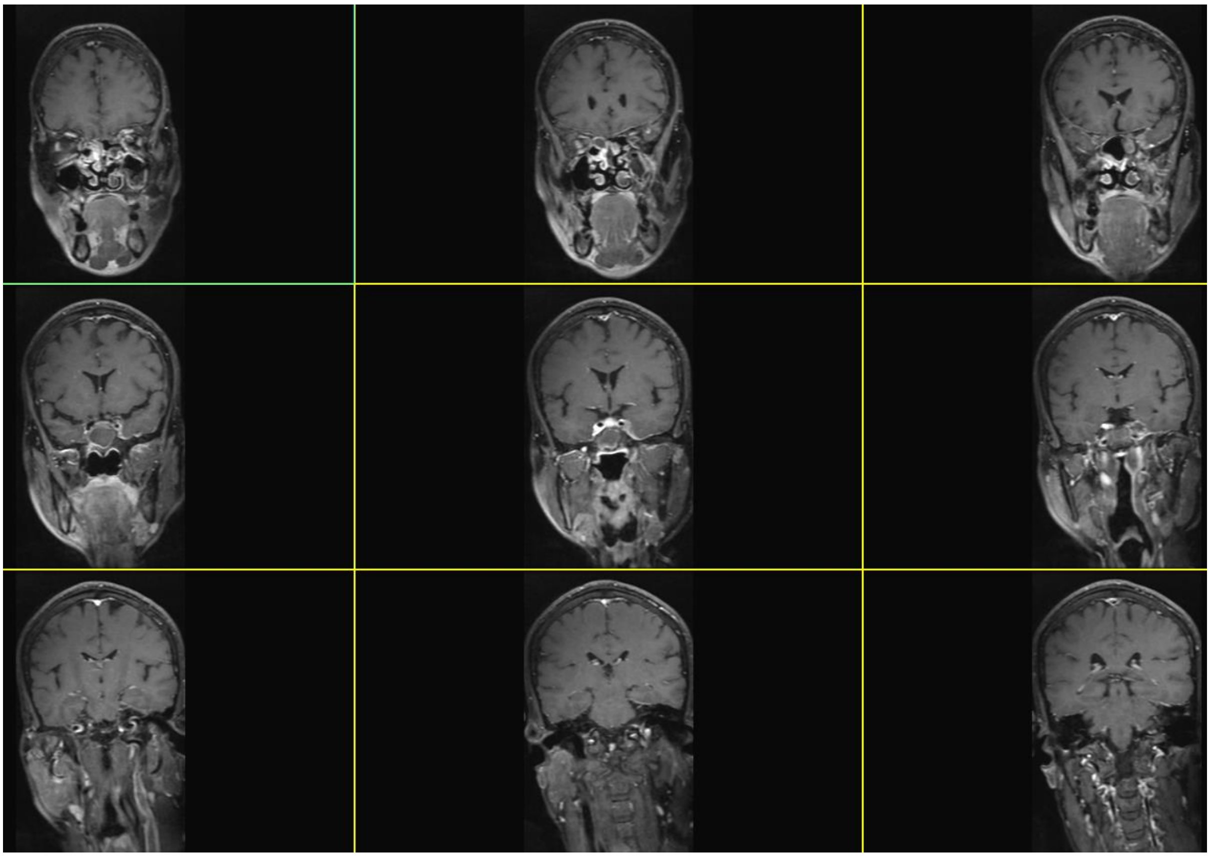

Figure P1 Magnetic Resonance CUBE processed Image in (3x3 display), Region of Interest is arteries, cisterns and tissues of cerebral region

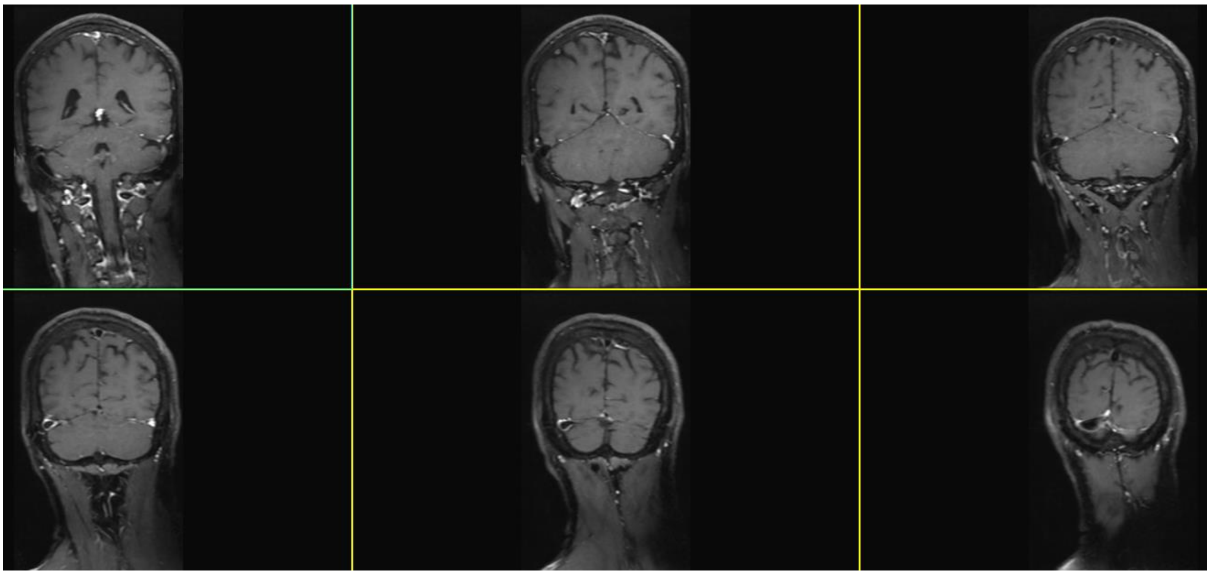

Figure P2 Magnetic Resonance CUBE processed Image in (2x3 display), Region of Interest is arteries, cisterns and tissues of cerebral region

Advantage of the above MRI images (figure P1-P2), are it is a contiguous, sub-millimetre, isotropic three-dimensional data sets of cerebral regions. This is possible due to CUBE technology which allows for volume isotropic turbo spin echo acquisition of MR images.

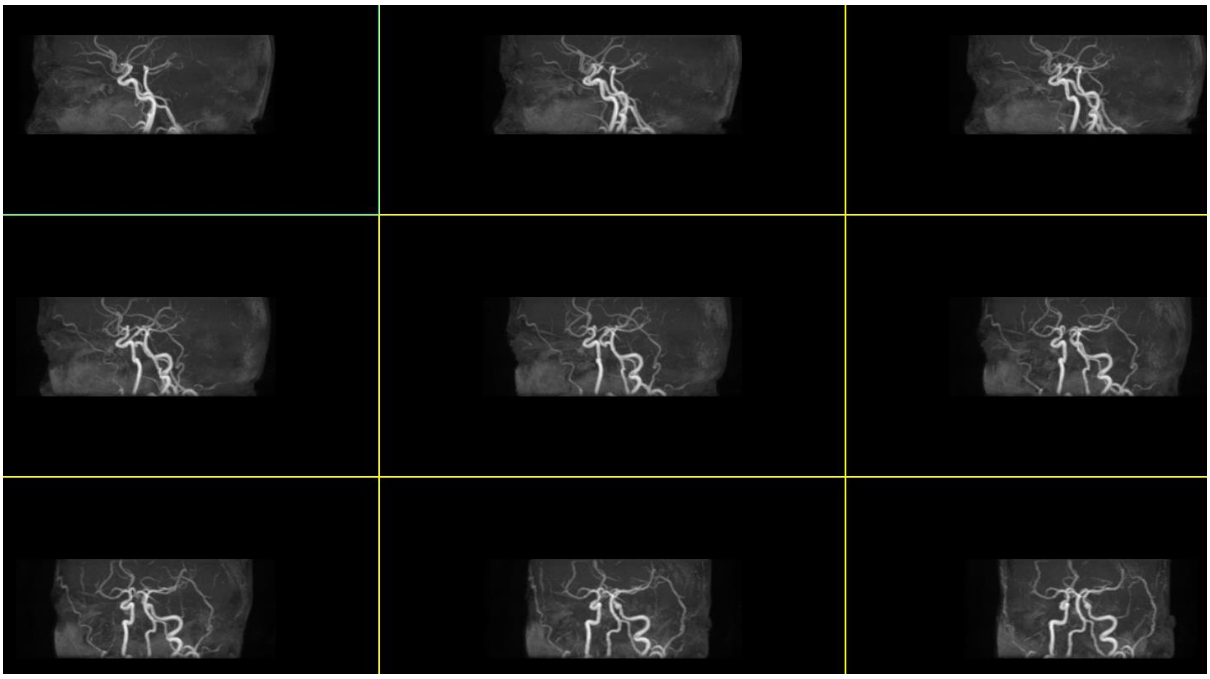

Figure Q1 Magnetic Resonance Venography image of brain (3x3 display)

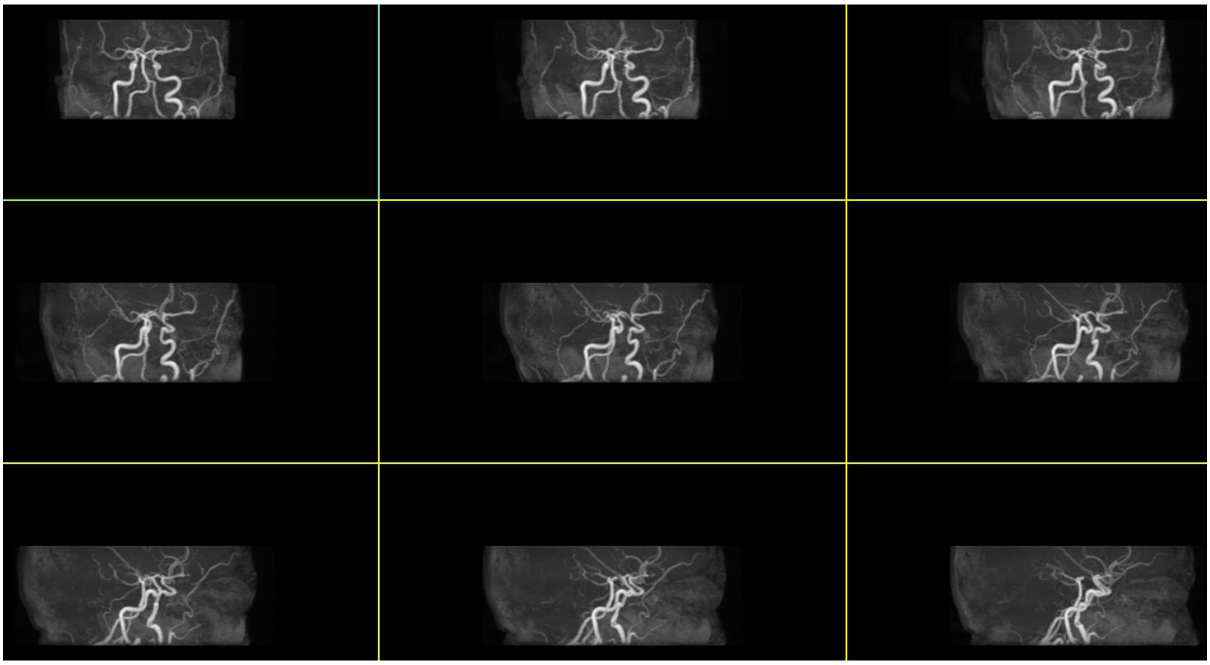

Figure Q2 Magnetic Resonance Venography image of brain (3x3 display)

The above MR Venography images of brain are mainly used for study the abnormalities if any in the Dural venous sinuses region in the brain and also in regions of cerebral veins
