# Supplementary material for: Continuum topological derivative - a novel application tool for denoising CT and MRI medical images
Source: BMC Med Imaging. 2024 Jul 24;24:182. doi: 10.1186/s12880-024-01341-1 (PMC11267933; doi:10.1186/s12880-024-01341-1)
Supplement: Supplementary file 12 — Supplementary Material 12. [file 12880_2024_1341_MOESM12_ESM.docx]

**Table AS1** Quality Metrics for extensive intracranial hemorrhage

| Metrics | Continuum TD | Kuan Filter | Frost Filter | PMAD Filter(15 itrs) | Haar Wavelet | Ordinary Filter Min | Median Filter | Wiener Filter | Average Filter 7x7 | Gaussian Filter | Laplacian Filter | Laplacian Filter Sharp |
| --- | --- | --- | --- | --- | --- | --- | --- | --- | --- | --- | --- | --- |
| AD | 0.0927 | 0.8763 | 0.4787 | 3.54 | 1.04 | 8.41 | 0.3578 | 1.24 | 3.60 | 2.26 | 2.18 | 5.55 |
| MSE | 0.1090 | 2.95 | 1.21 | 33.55 | 3.84 | 76.70 | 1.11 | 5.90 | 29.19 | 18.99 | 17.37 | 49.80 |
| RMSE | 0.3301 | 1.72 | 1.10 | 5.79 | 1.96 | 8.75 | 1.05 | 2.43 | 5.40 | 4.35 | 4.16 | 7.05 |
| PSNR | 57.76 | 43.42 | 47.30 | 32.87 | 42.28 | 29.28 | 47.65 | 40.41 | 33.48 | 35.34 | 35.73 | 31.15 |
| MD | 6 | 101 | 13 | 55 | 11 | 103 | 19 | 22 | 88 | 44 | 47 | 114 |
| NAE | 0.0013 | 0.0124 | 0.0068 | 0.0502 | 0.0147 | 0.1191 | 0.0051 | 0.0176 | 0.0511 | 0.032 | 0.0310 | 0.0786 |
| NMSE | 4.82e-04 | 0.0147 | 0.0060 | 0.1622 | 0.0186 | 0.3760 | 0.0055 | 0.0284 | 0.1469 | 0.0925 | 0.0851 | 0.2429 |
| SC | 1 | 0.99 | 0.99 | 0.98 | 1 | 1.06 | 1 | 0.99 | 1.01 | 0.98 | 1.03 | 1.08 |
| CC | 1 | 0.99 | 1 | 0.98 | 1 | 0.9838 | 1 | 0.99 | 0.98 | 0.99 | 0.99 | 0.98 |
| NCC | 1 | 0.99 | 1 | 1 | 1 | 0.9651 | 1 | 1 | 0.99 | 1 | 0.97 | 0.93 |
| IQI | 1 | 0.9770 | 0.9881 | 1.04 | 0.9470 | 0.8948 | 0.99 | 0.9706 | 0.90 | 0.92 | 0.98 | 0.92 |
| SSIM | 1 | 0.9758 | 0.99 | 0.8107 | 0.9653 | 0.8850 | 0.99 | 0.9552 | 0.87 | 0.90 | 0.96 | 0.84 |
| CNR | 1.04e-06 | 0.0025 | 0.0017 | 0.0044 | 1.83e-04 | 0.1178 | 2.03e-05 | 0.0012 | 0.02 | 1.73e-05 | 0.03 | 0.0748 |
| NI | 1.12e-05 | 1.11e-05 | 1.11e-05 | 1.06e-05 | 1.11e-05 | 1.20e-05 | 1.11e-05 | 1.10e-05 | 1.13e-05 | 1.07e-05 | 1.16e-05 | 1.24e-05 |
| ASNR | 8.90e+04 | 8.95e+04 | 8.99e+04 | 9.36e+04 | 8.95e+04 | 8.31e+04 | 8.93e+04 | 9.03e+04 | 8.82e+04 | 9.26e+04 | 8.56e+04 | 8.05e+04 |
| IV | 2.70e+03 | 2.66e+03 | 2.65e+03 | 2.42e+03 | 2.67e+03 | 2.40e+03 | 2.67e+03 | 2.61e+03 | 2.62e+03 | 2.49e+03 | 2.73e+03 | 2.80e+03 |
| NSD | 3.27e+08 | 3.25e+08 | 3.27e+08 | 3.23e+08 | 3.26e+08 | 2.53e+08 | 3.26e+08 | 3.25e+08 | 3.11e+08 | 3.27e+08 | 3.06e+08 | 2.77e+08 |
| ENL | 4.67e-14 | 4.69e-14 | 4.65e-14 | 4.71e-14 | 4.70e-14 | 6.01e-14 | 4.67e-14 | 4.68e-14 | 4.89e-14 | 4.67e-14 | 4.97e-14 | 5.50e-14 |
