# Supplementary material for: Continuum topological derivative - a novel application tool for denoising CT and MRI medical images
Source: BMC Med Imaging. 2024 Jul 24;24:182. doi: 10.1186/s12880-024-01341-1 (PMC11267933; doi:10.1186/s12880-024-01341-1)
Supplement: Supplementary file 13 — Supplementary Material 13. [file 12880_2024_1341_MOESM13_ESM.docx]

**Table BS1** Quality Metrics for fractured left knee

| Metrics | Continuum TD | Kuan Filter | Frost Filter | PMAD Filter(15 itrs) | Haar Wavelet | Ordinary Filter Min | Median Filter | Wiener Filter | Average Filter 7x7 | Gaussian Filter | Laplacian Filter | Laplacian Filter Sharp |
| --- | --- | --- | --- | --- | --- | --- | --- | --- | --- | --- | --- | --- |
| AD | 0.063 | 1.19 | 0.7204 | 2.67 | 0.8762 | 6.90 | 0.6247 | 1.17 | 2.73 | 1.98 | 2.99 | 7.07 |
| MSE | 0.1000 | 6.70 | 3.55 | 23.05 | 3.34 | 55.65 | 3.18 | 6.55 | 21.36 | 15.28 | 26.26 | 56.52 |
| RMSE | 0.3162 | 2.59 | 1.88 | 4.80 | 1.82 | 7.45 | 1.78 | 2.56 | 4.62 | 3.90 | 5.12 | 7.51 |
| PSNR | 58.13 | 39.86 | 42.62 | 34.50 | 42.89 | 30.67 | 43.09 | 39.96 | 34.83 | 36.28 | 33.93 | 30.60 |
| MD | 10 | 73 | 38 | 77 | 11 | 147 | 41 | 25 | 88 | 90 | 100 | 157 |
| NAE | 0.0012 | 0.0220 | 0.0132 | 0.0491 | 0.0161 | 0.1267 | 0.0115 | 0.0216 | 0.0502 | 0.0364 | 0.0549 | 0.1300 |
| NMSE | 5.62e-04 | 0.0381 | 0.0210 | 0.1273 | 0.0185 | 0.3099 | 0.0187 | 0.0372 | 0.1220 | 0.0869 | 0.1501 | 0.3188 |
| SC | 1 | 1 | 1 | 0.98 | 1 | 1.03 | 1 | 0.99 | 1 | 0.98 | 1.02 | 1.08 |
| CC | 1 | 1 | 1 | 0.98 | 1 | 0.97 | 1 | 1 | 0.98 | 0.98 | 1 | 0.9564 |
| NCC | 1 | 0.99 | 1 | 1 | 1 | 0.98 | 1 | 1 | 1 | 1 | 0.98 | 0.9421 |
| IQI | 1 | 0.98 | 0.98 | 0.94 | 0.95 | 0.91 | 0.98 | 1 | 0.96 | 0.96 | 0.96 | 0.9228 |
| SSIM | 1 | 0.95 | 0.97 | 0.82 | 0.96 | 0.86 | 0.97 | 0.92 | 0.89 | 0.87 | 0.91 | 0.7439 |
| CNR | 6.65e-07 | 0.0040 | 0.0014 | 0.0055 | 1.39e-04 | 0.1052 | 0.0012 | 4.89e-04 | 0.0183 | 5.76e-05 | 0.0436 | 0.1032 |
| NI | 1.36e-05 | 1.35e-05 | 1.34e-05 | 1.31e-05 | 1.35e-05 | 1.41e-05 | 1.35e-05 | 1.34e-05 | 1.37e-05 | 1.31e-05 | 1.42e-05 | 1.55e-05 |
| ASNR | 7.33e+04 | 7.37e+04 | 7.42e+04 | 7.62e+04 | 7.37e+04 | 7.06e+04 | 7.37e+04 | 7.41e+04 | 7.31e+04 | 7.62e+04 | 6.99e+04 | 6.42e+04 |
| IV | 2.36e+03 | 2.31e+03 | 2.31e+03 | 2.16e+03 | 2.34e+03 | 1.94e+03 | 2.33e+03 | 2.31e+03 | 2.27e+03 | 2.18e+03 | 2.32e+03 | 2.33e+03 |
| NSD | 1.94e+08 | 1.92e+08 | 1.95e+08 | 1.91e+08 | 1.94e+08 | 1.48e+08 | 1.93e+08 | 1.94e+08 | 1.85e+08 | 1.94e+08 | 1.73e+08 | 1.47e+08 |
| ENL | 7.85e-14 | 7.93e-14 | 7.82e-14 | 7.96e-14 | 7.85e-14 | 1.03e-13 | 7.87e-14 | 7.86e-14 | 8.22e-14 | 7.85e-14 | 8.79e-14 | 1.03e-13 |
