# Supplementary material for: Continuum topological derivative - a novel application tool for denoising CT and MRI medical images
Source: BMC Med Imaging. 2024 Jul 24;24:182. doi: 10.1186/s12880-024-01341-1 (PMC11267933; doi:10.1186/s12880-024-01341-1)
Supplement: Supplementary file 14 — Supplementary Material 14. [file 12880_2024_1341_MOESM14_ESM.docx]

**Table CS1** Quality Metrics for complex cranial base axial

| Metrics | Continuum TD | Kuan Filter | Frost Filter | PMAD Filter(15 itrs) | Haar Wavelet | Ordinary Filter Min | Median Filter | Wiener Filter | Average Filter 7x7 | Gaussian Filter | Laplacian Filter | Laplacian Filter Sharp |
| --- | --- | --- | --- | --- | --- | --- | --- | --- | --- | --- | --- | --- |
| AD | 0.0051 | 2.75 | 2.24 | 5.37 | 0.5119 | 18.97 | 1.11 | 2.95 | 8.35 | 7.11 | 4.69 | 8.61 |
| MSE | 0.0172 | 24.67 | 20.17 | 48.86 | 1.26 | 89.17 | 8.57 | 30.76 | 47.39 | 48.17 | 33.21 | 42.05 |
| RMSE | 0.1311 | 4.96 | 4.49 | 6.99 | 1.12 | 9.44 | 2.92 | 5.54 | 6.88 | 6.94 | 5.76 | 6.48 |
| PSNR | 65.77 | 34.20 | 35.08 | 31.24 | 47.09 | 28.62 | 38.79 | 33.25 | 31.37 | 31.30 | 32.91 | 31.89 |
| MD | 19 | 107 | 92 | 119 | 5 | 234 | 136 | 105 | 173 | 175 | 170 | 209 |
| NAE | 6.31e-05 | 0.0344 | 0.0281 | 0.0672 | 0.0064 | 0.2374 | 0.0138 | 0.0370 | 0.1046 | 0.0889 | 0.0587 | 0.1077 |
| NMSE | 1.40e-04 | 0.1791 | 0.1472 | 0.3550 | 0.0091 | 0.6505 | 0.0606 | 0.2208 | 0.3496 | 0.3502 | 0.2464 | 0.3152 |
| SC | 1 | 0.9371 | 0.94 | 0.83 | 1 | 1.19 | 1 | 0.89 | 0.90 | 0.84 | 1.09 | 1.17 |
| CC | 1 | 0.99 | 0.99 | 0.98 | 1 | 0.92 | 1 | 0.99 | 0.95 | 0.96 | 0.98 | 0.96 |
| NCC | 1 | 1.01 | 1.01 | 1.02 | 1 | 0.88 | 0.99 | 1.01 | 1.01 | 1.02 | 0.92 | 0.86 |
| IQI | 1 | 0.96 | 0.96 | 0.90 | 0.96 | 0.75 | 1 | 0.90 | 0.91 | 0.8770 | 0.95 | 0.94 |
| SSIM | 1 | 0.94 | 0.96 | 0.75 | 0.99 | 0.66 | 0.97 | 0.88 | 0.73 | 0.7318 | 0.93 | 0.85 |
| CNR | 6.86e-07 | 2.94e-05 | 4.41e-04 | 3.12e-04 | 6.86e-05 | 0.1499 | 0.0013 | 8.57e-04 | 0.0142 | 1.86e-04 | 0.0352 | 0.0645 |
| NI | 1.79e-05 | 1.73e-05 | 1.75e-05 | 1.69e-05 | 1.79e-05 | 2.11e-05 | 1.79e-05 | 1.73e-05 | 1.71e-05 | 1.63e-05 | 1.91e-05 | 2.02e-05 |
| ASNR | 5.55e+04 | 5.74e+04 | 5.71e+04 | 5.89e+04 | 5.57e+04 | 4.73e+04 | 5.55e+04 | 5.75e+04 | 5.82e+04 | 6.10e+04 | 5.22e+04 | 4.94e+04 |
| IV | 8.89e+03 | 8.30e+03 | 8.42e+03 | 7.89e+03 | 8.83e+03 | 7.13e+03 | 8.84e+03 | 8.29e+03 | 7.71e+03 | 7.34e+03 | 8.91e+03 | 8.94e+03 |
| NSD | 4.18e+08 | 4.18e+08 | 4.19e+08 | 4.18e+08 | 4.18e+08 | 2.43e+08 | 4.17e+08 | 4.20e+08 | 3.99e+08 | 4.18e+08 | 3.71e+08 | 3.33e+08 |
| ENL | 3.64e-14 | 3.64e-14 | 3.63e-14 | 3.64e-14 | 3.64e-14 | 6.26e-14 | 3.65e-14 | 3.63e-14 | 3.81e-14 | 3.64e-14 | 4.11e-14 | 4.57e-14 |
